# Longitudinal Profiling of Fasting Plasma Metabolome in Response to Weight-Loss Interventions in Patients with Morbid Obesity

**DOI:** 10.3390/metabo14020116

**Published:** 2024-02-10

**Authors:** Mingjing Chen, Guanhong Miao, Zhiguang Huo, Hao Peng, Xiaoxiao Wen, Stephen Anton, Dachuan Zhang, Gang Hu, Ricky Brock, Phillip J. Brantley, Jinying Zhao

**Affiliations:** 1 Department of Epidemiology, College of Public Health & Health Professions, University of Florida, Gainesville, FL 32603, USA; mingjingchen@ufl.edu (M.C.); xiaoxiao.wen@ufl.edu (X.W.); 2 Department of Biostatistics, College of Public Health & Health Professions, University of Florida, Gainesville, FL 32603, USA; zhuo@ufl.edu; 3 Department of Epidemiology and Biostatistics, School of Public Health, Medical College, Soochow University, Suzhou 215123, China; penghao@suda.edu.cn; 4 Department of Aging and Geriatric Research, University of Florida, Gainesville, FL 32603, USA; santon@ufl.edu; 5 Department of Biostatistics, Pennington Biomedical Research Center, Louisiana State University System, Baton Rouge, LA 70808, USA; dachuan.zhang@pbrc.edu; 6 Chronic Disease Epidemiology Laboratory, Pennington Biomedical Research Center, Louisiana State University System, Baton Rouge, LA 70808, USA; gang.hu@pbrc.edu; 7 Behavioral Medicine Laboratory, Pennington Biomedical Research Center, Louisiana State University System, Baton Rouge, LA 70808, USA; ricky.brock@pbrc.edu (R.B.); phil.brantley@pbrc.edu (P.J.B.)

**Keywords:** obesity, bariatric surgery, metabolomics, glycemic outcomes

## Abstract

It is well recognized that patients with severe obesity exhibit remarkable heterogeneity in response to different types of weight-loss interventions. Those who undergo Roux-en-Y gastric bypass (RYGB) usually exhibit more favorable glycemic outcomes than those who receive adjustable gastric banding (BAND) or intensive medical intervention (IMI). The molecular mechanisms behind these observations, however, remain largely unknown. To identify the plasma metabolites associated with differential glycemic outcomes induced by weight-loss intervention, we studied 75 patients with severe obesity (25 each in RYGB, BAND, or IMI). Using untargeted metabolomics, we repeatedly measured 364 metabolites in plasma samples at baseline and 1-year after intervention. Linear regression was used to examine whether baseline metabolites or changes in metabolites are associated with differential glycemic outcomes in response to different types of weight-loss intervention, adjusting for sex, baseline age, and BMI as well as weight loss. Network analyses were performed to identify differential metabolic pathways involved in the observed associations. After correction for multiple testing (q < 0.05), 33 (RYGB vs. IMI) and 28 (RYGB vs. BAND) baseline metabolites were associated with changes in fasting plasma glucose (FPG) or glycated hemoglobin (HbA1c). Longitudinal changes in 38 (RYGB vs. IMI) and 38 metabolites (RYGB vs. BAND) were significantly associated with changes in FPG or HbA1c. The identified metabolites are enriched in pathways involved in the biosynthesis of aminoacyl-tRNA and branched-chain amino acids. Weight-loss intervention evokes extensive changes in plasma metabolites, and the altered metabolome may underlie the differential glycemic outcomes in response to different types of weight-loss intervention, independent of weight loss itself.

## 1. Introduction

Morbid obesity, defined as a body mass index (BMI) ≥ 40 kg/m^2^ or BMI ≥ 35 kg/m^2^ with comorbidities (e.g., diabetes), is a serious health problem that impedes normal daily life and activities [1]. Patients with morbid obesity suffer from an increased risk of type-2 diabetes [2], hypertension [3], heart disease [4], and cancer [4]. Although non-surgical interventions, such as calorie restriction, physical activity, and medication management, are effective in achieving short-term weight loss [5], bariatric surgery has proven to be most effective in achieving sustainable weight loss and long-term beneficial glycemic outcomes [6,7]. Of the several bariatric surgical procedures, such as adjustable gastric banding (BAND) and Roux-en-Y gastric bypass (RYGB), RYGB typically results in more weight loss and a higher rate of diabetes remission [8,9]. However, patients undergoing bariatric surgery exhibit remarkable heterogeneity in response to different types of surgical procedures as well as post-operative outcomes. Although several mechanisms such as genetics [10], epigenetics [11], and gut hormone [12] have been postulated to explain the beneficial effects of bariatric surgery, the precise mechanisms through which bariatric surgery induces favorable and heterogeneous glycemic outcomes remain largely unknown and unexplored. A better understanding of such mechanisms may lead to the discovery of novel mechanistic markers that can be used to identify patients who may benefit from a specific type of weight-loss intervention, thereby achieving the precise treatment of obesity.

Metabolomics is an emerging high-throughput biochemical technique that can identify and quantify numerous small metabolites (<1500 Da) in a biological sample. There is evidence that bariatric surgery can rewire cellular metabolism and results in extensive metabolic changes [13,14]. As an individual’s metabolic profile provides the functional readout of his/her current metabolic state that is responsive to both intrinsic and extrinsic perturbations [15,16], metabolomics provides a powerful tool to characterize the entire “metabolome” (all metabolites in a biological sample) and discover previously undescribed metabolic disturbances evoked by weight-loss intervention. In fact, changes in several metabolites such as branched-chain amino acids (BCAAs) [17,18], glycerophospholipids [13,19], and bile acids [20], have been shown to be differentially responsive to surgical versus lifestyle/behavioral interventions. However, several research gaps exist in this field. First, previous metabolomic studies on bariatric surgery were largely cross-sectional, which did not examine the relationship between a change in metabolites and a change in glycemic outcomes. Second, most previous studies employed a targeted approach by focusing on a preselected list of metabolites, resulting in a low coverage of the metabolome [21,22]. Third, to the best of our knowledge, very few previous metabolomic studies on bariatric surgery examined whether alterations in the blood metabolome underlie the differential glycemic outcomes in response to different types of weight-loss intervention. Fourth, most existing metabolomic studies on bariatric surgery did not control for weight loss, making it hard to interpret whether the observed post-operative metabolic improvements are due to bariatric surgery itself or subsequent weight loss. Our hypothesis is that metabolomic alterations underline the differential glycemic outcomes in response to different types of weight-loss intervention, independent of weight loss. Disentangling the weight-loss-independent mechanism is important because recent evidence [23,24] suggests that there are additional important mechanisms through which bariatric surgery can result in the improvement of glycemic outcomes, independent of weight loss. 

Using a longitudinal study design and an untargeted metabolomic approach, the goals of this study are to (1) identify baseline plasma metabolites associated with the differential glycemic outcomes in response to different types of weight-loss interventions, independent of known clinical factors and weight loss; and (2) examine the longitudinal association between a change in plasma metabolome and a change in glycemic indices (before and after weight-loss intervention), independent of baseline clinical factors and weight loss. 

## 2. Materials and Methods

### 2.1. Study Participants

The HeadsUp Study (July 2011–June 2016), funded by the State of Louisiana, was a longitudinal study designed for obesity management among patients with obesity (aged 21–70 years, 83% women, 29% black) in the State of Louisiana Office of Group Benefits (OGB). A detailed description of the study design and the inclusion and exclusion criteria has been previously reported [25]. Briefly, the HeadsUp Study was designed to examine the effectiveness of bariatric surgery and non-surgical intervention on short-term (6 months) and long-term (≥1 year) health outcomes, thereby providing guidance for future treatment options for patients with severe obesity [25]. Eligibility criteria for the surgical intervention included BMI > 40 kg/m^2^ or BMI > 35 kg/m^2^ with type-2 diabetes, and BMI ≥ 33 kg/m^2^ to be eligible for the intensive medical intervention (IMI) program. Exclusion criteria of the HeadsUp Study included (1) individuals who failed to complete the initial web-based or follow-up telephone screenings; (2) individuals who did not meet the BMI thresholds (BMI < 33 kg/m^2^ for non-surgical interventions and BMI < 35 kg/m^2^ without type-2 diabetes or <40 kg/m^2^ for surgical interventions); (3) individuals who did not achieve the required pre-screening weight loss of at least 4 pounds; (4) women who were pregnant or intended to become pregnant within 3 years; (5) individuals with significant medical conditions or psychiatric disorders; and (6) individuals who underwent prior bariatric surgeries or those who were unable to comply with the study protocol.

A total of 75 participants were enrolled into three equal-sized groups based on their clinical characteristics or personal preferences: 25 participants underwent Roux-en-Y gastric bypass (RYGB), 25 participants underwent adjustable gastric banding (BAND), and 25 participants underwent intensive medical intervention (IMI). All participants met the following criteria: aged 21–70 years; had available biospecimen (fasting plasma collected at baseline and at 1-year follow-up); and had completed clinical data at both baseline and 1-year follow-up. All participants provided informed consent. The HeadsUp Study protocols were approved by the Pennington Biomedical Research Center Institutional Review Boards.

### 2.2. Anthropometric and Clinical Measures

Demographic (age, gender, and race) and clinical (e.g., body weight, waist circumference, blood pressure, fasting plasma glucose (FPG), glycated hemoglobin (HbA1c), and cholesterol) information were collected at baseline and at 1-year follow-up. In brief, body weight, height, and waist circumference were measured at all study visits using a standardized protocol in which participants wore light clothes and no shoes. Body mass index (BMI) was calculated as the body weight in kilograms divided by the square of the height in meters. Blood pressure was measured by trained study staff based on standard procedures [26]. All patients in the surgical groups discontinued their diabetes medications on the day of surgery. Over time, participants in IMI group either discontinued their diabetes medications or reduced the dosage, taking only low doses of metformin after the weight-loss intervention. Samples were collected at both baseline and 1 year after intervention. Diabetes was defined as either fasting blood glucose ≥ 126 mg/dL and/or HbA1c ≥ 6.5% or receiving hypoglycemic medications based on the American Diabetes Association criteria [27]. 

### 2.3. Study Outcomes

Our primary glycemic outcomes include percent (%) change in fasting plasma glucose or HbA1c (i.e., the difference between follow-up and baseline, divided by baseline value) before and 1 year after weight-loss intervention. 

### 2.4. Metabolomic Data Acquisition, Pre-Processing, and Quality Control 

Fasting plasma samples (EDTA-treated) were shipped on dry ice overnight to the West Coast Metabolomics Center and immediately stored at −80 °C until analysis. Methods for metabolites data acquisition, processing, and normalization have been described previously [28,29]. Briefly, relative abundance of plasma metabolites was quantified by gas chromatography time-of-flight mass spectrometry. Results were exported and further processed using the metabolomics BinBase database, a relational database system employed for automated metabolite annotation [30]. Each entry in BinBase was matched against the Fiehn mass spectral library of 1200 authentic metabolite spectra using retention index and mass spectrum information, or against the NIST05 commercial library. Metabolites were reported only if present within at least 50% of each study group [31]. Peak heights of quantifier ions defined for each metabolite in BinBase were normalized to the sum intensities of all known metabolites and were used for statistical analysis. Batch effect was removed by SERRF [32], a quality-control-based sample normalization method for large-scale untargeted metabolomics data. After pre-processing and quality control, a total of 364 plasma metabolites (153 known) were identified in 150 plasma samples from 75 participants at both baseline and 1-year follow-up. No batch effect was observed in our metabolomic data (Figure S1).

### 2.5. Statistical Analysis 

Prior to analysis, all continuous variables including metabolites were standardized to zero mean and unit variance. All statistical analyses were performed using R version 4.1.1. Changes in clinical characteristics were calculated as the measurement at 1-year follow-up minus the baseline value. Differences in the changes were tested by paired *t*-test or Wilcoxon rank test for statistical significance. Figure S2 describes the analytic plan of this study.

#### 2.5.1. Prospective Association Analysis

We constructed linear regression models to identify baseline plasma metabolites associated with the differential glycemic outcomes in response to different types of weight-loss intervention. In the model, % change in fasting plasma glucose or % change in HbA1c was the dependent variable, and baseline metabolite, intervention groups (RYGB, BAND, IMI), and their interactions (metabolite × types of intervention) were the independent variables. The model adjusted for sex, baseline age, and BMI as well as weight loss. Here, we are especially interested in testing the interaction term. A significant interaction indicates that baseline plasma metabolite is associated with differential glycemic outcomes induced by different types of intervention, independent of covariates including weight loss. Multiple testing was controlled by false discovery rate and Storey’s q-value (q) < 0.05 was considered significant [33].

#### 2.5.2. Repeated Measurement Analysis

We constructed linear regression models to examine the association between longitudinal changes in plasma metabolites and changes in glycemic outcomes (before and 1 year after intervention) in response to different types of weight-loss intervention. In the model, the dependent variable was % change in fasting plasma glucose or % change in HbA1c, and % change in metabolite, intervention groups (RYGB, BAND, IMI), and their interactions (i.e., % change in metabolite × types of intervention) were the independent variables, adjusting for sex, baseline age, and BMI as well as weight loss. Storey’s q-value (q) < 0.05 was considered significant [33].

#### 2.5.3. Differential Metabolic Networks

Given the high correlations between plasma metabolites, we performed the Weighted Gene Correlation Network Analysis (WGCNA) [34] to identify differential metabolic networks (i.e., clusters of correlated metabolites) associated with different types of weight-loss intervention. To control for potential confounding, we first regressed out the effects of clinical variables from each metabolite, including age, sex, BMI, and weight loss, and used the residuals in the network analysis. In this analysis, correlated metabolites were hierarchically clustered, and those with a high topological overlap similarity were grouped into the same module. Modular differential connectivity analysis [35] was performed to examine the difference in module connectivity between different intervention groups, e.g., RYGB vs. IMI, RYGB vs. BAND. The statistical significance of the modular differential connectivity analysis was assessed by 1000 permutations [36]. Gain of connectivity (GOC) was defined if the correlation among metabolites in one group (e.g., RYGB) was significantly higher than that in another group (e.g., BAND or IMI). Loss of connectivity (LOC) was similarly defined.

#### 2.5.4. Pathway-Enrichment Analysis

To examine whether the identified metabolites are involved in biological pathways, we submitted the metabolites identified in the network analysis to the program MetaboAnalyst 5.0 [37]. The background information of this analysis included 153 known metabolites detected in all samples from all participants. The statistical significance of each pathway was evaluated using the Fisher’s exact test. The importance of each pathway was tested by topological analysis [38]. A pathway impact score was defined as the sum of the importance measures for the identified metabolites divided by the total sum of importance measures of all metabolites in the pathway. 

## 3. Results

**Figure 1:**
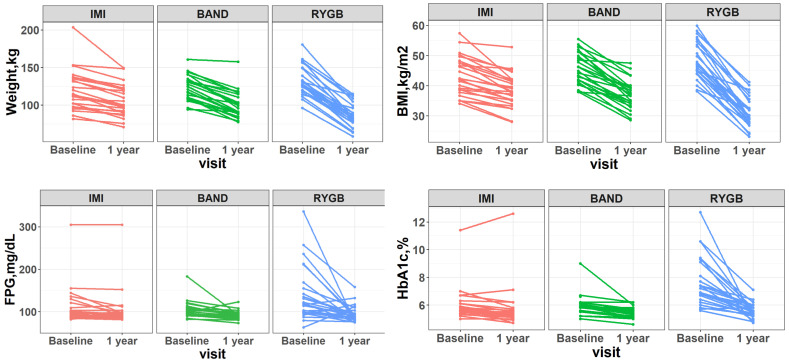
Changes in body weight, BMI, fasting plasma glucose (FPG), and HbA1c (before and 1 year after intervention) in response to different types of weight-loss intervention. IMI: intensive medical intervention. BAND: adjustable gastric banding. RYGB: Roux-en-Y gastric bypass surgery.

The clinical characteristics of participants are shown in Table 1. The mean age of participants was 51 ± 8 years, and the mean BMI at baseline was 45.8 kg/m^2^ (range: 34.1 to 59.9). While all three types of intervention resulted in significant reduction in body weight, waist circumference, blood pressure, fasting plasma glucose, and HbA1c (all *p*-values < 0.05), participants who received RYGB exhibited the largest weight loss and the highest diabetes remission rate (90%), followed by those who received BAND or IMI (Figure 1). 

### 3.1. Baseline Plasma Metabolites Associated with Changes in Glycemic Outcomes, Independent of Weight Loss

After correction for multiple testing (q < 0.05), baseline levels of 33 (13 known) metabolites were significantly associated with changes in fasting plasma glucose or HbA1c when comparing RYGB to IMI. In the comparison between RYGB and BAND, baseline levels of 28 (14 known) metabolites were significantly associated with changes in fasting plasma glucose or HbA1c (Table 2 and Table 3). 

Of note, baseline levels of nine known metabolites, including 3-aminoisobutyric acid, behenic acid, butane-2,3-diol, creatinine, gluconic acid, hydrocinnamic acid, methionine, pyrophosphate, and 1,5-anhydroglucitol, were significantly associated with changes in fasting plasma glucose or HbA1c when comparing RYGB to IMI or BAND. These associations remained largely unchanged after further adjusting for use of hypoglycemic drugs (including insulin use) prior to intervention. A full list of baseline metabolites associated with changes in fasting plasma glucose or HbA1c across different types of intervention is shown in Tables S1 and S2.

### 3.2. Longitudinal Changes in Plasma Metabolites Associated with Changes in Glycemic Outcomes, Independent of Weight Loss

**Figure 2:**
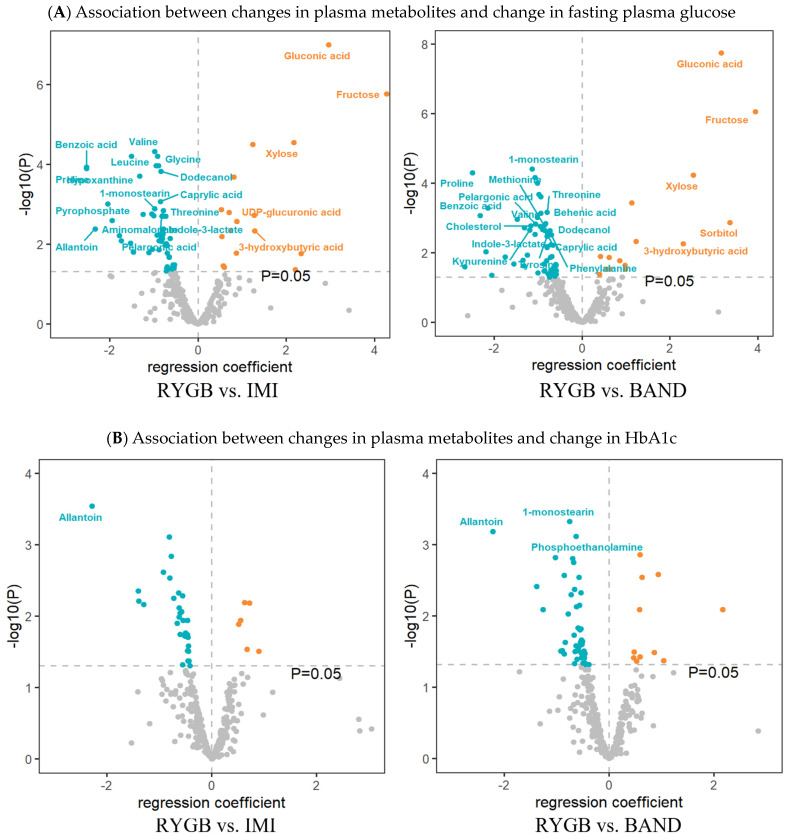
Volcano plots displaying the longitudinal association between changes in plasma metabolites and changes in fasting plasma glucose (FPG) or changes in HbA1c in response to different types of weight-loss interventions. Regression coefficients (*x*-axis) were obtained from the linear regression model, in which % change in FPG or % change in HbA1c (follow-up value minus baseline, divided by baseline value) was the dependent variable, and % change in metabolite, intervention groups (1 = IMI, 2 = BAND, 3 = RYGB, with IMI being the reference), and their interaction (% change in each metabolite × types of intervention) were the independent variables, adjusting for age, sex, baseline BMI, and weight loss. Only known metabolites with *p* < 0.05 are shown. Metabolites with q < 0.05 are labeled.

After correction for multiple testing (q < 0.05), longitudinal changes in 38 metabolites (20 known) were significantly associated with changes in fasting plasma glucose, and changes in 4 metabolites (1 known) were significantly associated with changes in HbA1c (Figure 2A and Figure S3) when comparing RYGB to IMI. When comparing RYGB to BAND, longitudinal changes in 36 metabolites (20 known) were significantly associated with changes in fasting plasma glucose, and changes in 6 metabolites (3 known) were significantly associated with changes in HbA1c (Figure 2B). 

Of note, longitudinal change in one metabolite (i.e., allantoin) was inversely associated with changes in both fasting plasma glucose and HbA1c when comparing RYGB to IMI or BAND. The longitudinal association between changes in all measured metabolites and changes in fasting plasma glucose or HbA1c across different types of intervention is shown in Tables S3 and S4.

### 3.3. Differential Metabolic Networks Associated with Different Types of Weight-Loss Intervention

**Figure 3:**
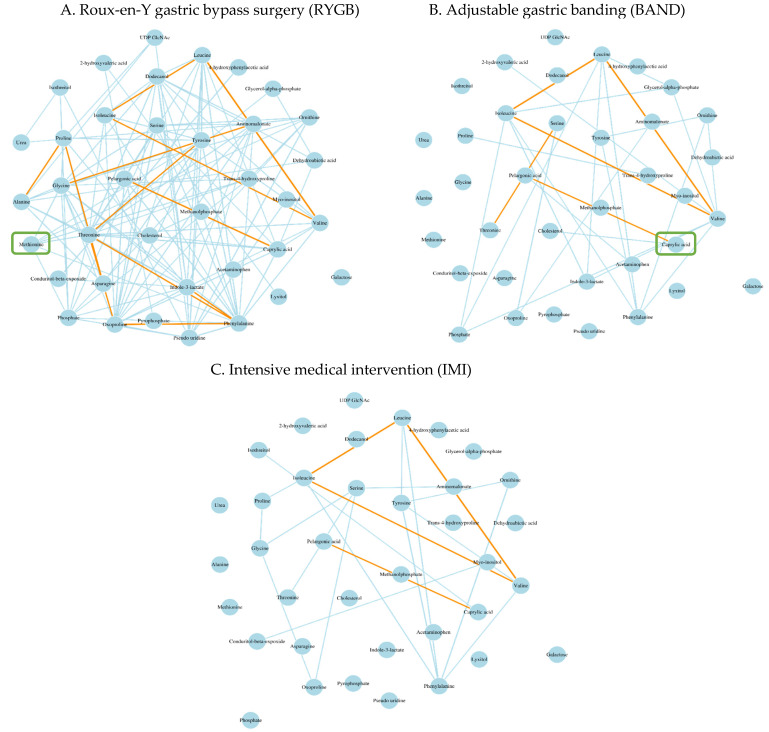
Differential metabolites networks associated with different types of weight-loss intervention. Gain of connectivity in the blue module was observed among participants who underwent RYGB (**A**) compared to those who underwent IMI (**B**) (modular differential connectivity [RYGB vs. IMI] = 82.7, *p* = 0.001) or those who underwent BAND (**C**) (modular differential connectivity [RYGB vs. BAND] = 89.7, *p* = 0.013). The edge colors reflect the strength of correlation between metabolites with orange color showing the strongest correlation, followed by the light blue color. Hub metabolite in each module was labelled by a green square.

Our network analysis identified five metabolic modules in the RYGB group and three modules in each of the IMI and the BAND group (Tables S5–S7, Figure S4). Of these, two modules showed differential co-regulation across the three intervention groups. Specifically, metabolites in one module (the blue module) exhibited a significant gain of connectivity (GOC) in comparing RYGB to IMI (modular differential connectivity = 82.7, *p* = 0.001). When comparing RYGB to BAND, two modules showed significant differential co-regulation, with one showing gain of connectivity (blue module, modular differential connectivity = 89.7, *p* = 0.013, Figure 3) and the other showing loss of connectivity (turquoise module, modular differential connectivity = −15.94, *p* = 0.011, Figure S5).

Pathway enrichment analysis indicated that, among patients receiving RYGB, the 37 metabolites identified in the blue module (34 metabolites matched to Human Metabolome Database or Kyoto Encyclopedia of Genes and Genomes database) were enriched in the biosynthesis of aminoacyl-tRNA and branched-chain amino acids (valine, leucine, and isoleucine) at *p* < 0.05 (Figure S6). More details for the pathway enrichment and network analyses can be found in Tables S8–S12.

## 4. Discussion

Using an untargeted metabolomic approach and a longitudinal study design, we found that (1) baseline metabolites and their longitudinal changes are associated with post-operative glycemic outcomes, independent of known clinical variables and weight loss; (2) alterations in plasma metabolites may underlie the differential glycemic outcomes in response to different types of weight-loss intervention, independent of weight loss; and (3) RYGB evokes the most extensive metabolic changes compared to BAND or IMI. 

In comparing RYGB to non-surgical intervention (IMI) or another surgical intervention (BAND), we found that higher baseline levels of 1,5-anhydroglucitol and 3-aminoisobutyric acid were positively associated with changes in HbA1c and changes in fasting plasma glucose, respectively, before and after intervention. To our knowledge, few previous studies have reported the associations of these metabolites with glycemic outcomes among patients who underwent RYGB compared to those who underwent other types of weight-loss interventions. The metabolite 1,5-anhydroglucitol is a six-carbon monosaccharide mainly derived from dietary intake. Circulating 1,5-anhydroglucitol has been consistently reported to be reduced in patients with type 2 diabetes and was inversely correlated with fasting plasma glucose and HbA1c in previous studies [39,40]. Moreover, circulating 1,5-anhydroglucitol was associated with the loss of functional β cells in a mouse model of diabetes [41] and human subjects [39]. 3-aminoisobutyric acid, also known as β-aminoisobutyric acid, is a non-protein amino acid produced by skeletal muscle during physical activity [42]. Previous research has shown a that higher level of plasma 3-aminoisobutyric acid is associated with decreased levels of glucose, insulin, triglyceride, and total cholesterol in Caucasians and African Americans [43]. It is possible that 3-aminoisobutyric acid may affect glycemic outcomes by increasing fatty acid oxidation and glucose homeostasis through the peroxisome proliferator-activated receptor gamma coactivator 1-alpha (PGC-1α)-dependent pathway [43]. 

Our repeated measurement analyses showed that changes in plasma metabolites are associated with changes in glycemic indices, independent of baseline clinical factors and weight loss. For instance, longitudinal changes in 51 metabolites (28 known), largely amino acids, lipids, fatty acids and ketone bodies, were significantly associated with changes in fasting plasma glucose, HbA1c, or both during the 1-year follow-up after intervention. Specifically, longitudinal changes in branched-chain amino acids (e.g., valine, leucine) and other amino acids (e.g., threonine, proline, tyrosine and glycine) were inversely associated with glycemic outcomes. The observed associations between amino acids, especially branched-chain amino acids, and glycemic outcomes following bariatric surgery, appeared to be in agreement with previous studies showing that reduced branched-chain amino acids levels were associated with improved post-operative glycemic outcomes [44,45]. These results also corroborated a multi-center randomized controlled trial showing that branched-chain amino acids and tyrosine may serve as potential biomarkers for glycemic improvement following weight-loss interventions [46]. In addition, we found that longitudinal changes in 3-hydroxybutyric acid were positively associated with changes in glycemic outcomes in the comparisons between RYGB and IMI or between RYGB and BAND. The metabolite 3-hydroxybutyric acid, also known as the ketone body β-hydroxybutyric acid, is a natural compound produced in the process of fat metabolism. It has been shown that 3-hydroxybutyric acid may serve as an early metabolic marker for the long-term prognosis of bariatric surgery [44]. It is likely that 3-hydroxybutyric acid may influence glycemic outcomes by mediating the homeostasis of the intestinal stem [47] or by inducing the expression of antioxidative factors [48]. Importantly, we observed that, compared to patients who underwent IMI or BAND, those who received RYGB exhibited the most extensive metabolic changes before and after intervention. This finding may explain, at least in part, why patients receiving RYGB usually exhibit the most favorable post-operative metabolic outcomes (e.g., larger weight loss, rapid diabetes remission) than those receiving BAND or non-surgical weight-loss intervention (IMI). 

In this study, we controlled for weight loss in all statistical analyses. Despite that weight loss induced by bariatric surgery has profound beneficial effects on the metabolic abnormalities involved in obesity and type-2 diabetes (i.e., a weight loss-dependent mechanism), bariatric surgery has important weight loss-independent mechanisms [24] that are currently poorly understood. Although a number of hypotheses [49] (e.g., insulin sensitivity, incretin response, gut microbiome, bile acid metabolism, intestinal glucose metabolism, etc.) have been proposed to explain the beneficial glycemic outcomes after bariatric surgery, none can fully explain the potential unique beneficial effects of bariatric surgery. Thus, it is crucial to disentangle the mechanisms through which bariatric surgery can lead to the substantial improvement of glycemic outcomes, independent of weight loss. Such results not only deepen our understanding of the mechanisms underlying the beneficial effects of bariatric surgery but also help develop weight loss-independent therapeutic strategies.

Our network analysis revealed differential metabolic networks in response to different types of weight-loss interventions. Specifically, of the 37 metabolites clustered into the blue module in the RYGB group, 18 metabolites, including 15 amino acids (e.g., branched-chain amino acids, aromatic amino acids, and methionine, etc.) and three lipids (e.g., caprylic acid, cholesterol, and dodecanol), were clustered in this group but not in the BAND or the IMI group. These findings appeared to be consistent with previous studies [21,50] showing that patients who experienced post-operative diabetes remission exhibited differential metabolic profiles for branched-chain amino acids, aromatic amino acids, and lipids compared to those who did not. In addition, we found that methionine was highly correlated with other metabolites in the RYGB group, but not the other two groups (BAND, IMI). In line with this finding, previous research has shown that methionine could serve as a potential biomarker for oxidative stress [51], a mechanism known to be involved in obesity [52] and diabetes [53]. Another study reported that bariatric surgery resulted in a reduction of blood methionine [54]. Together, these findings lend support to our hypothesis that altered metabolites may underlie the differential glycemic outcomes induced by different types of weight-loss interventions.

The present study has several strengths. First, the repeated measurement analysis of both pre- and post-operative blood metabolome represents the first of its kind in this field. Such a longitudinal design allows us to examine the temporal relationship between changes in plasma metabolome and changes in glycemic outcomes evoked by different types of weight-loss intervention. Second, by using an untargeted metabolomic approach, our study has a relatively high coverage of the blood metabolome, which allows for the identification of novel metabolites that will not be uncovered otherwise. Finally, as weight-loss is known to be related to favorable glycemic outcomes, the adjustment of weight-loss in all statistical analyses allows us to identify metabolites’ effects that are beyond weight loss itself.

Some limitations should also be noted. First, the number of participants in each intervention group was small; thus, we might have missed some important metabolites. In addition, due to ethical consideration, participants were enrolled into different intervention groups based on their clinical characteristics or personal preferences rather than being randomly assigned, which resulted in baseline differences across the groups. However, we have adjusted for these clinical factors in all statistical models. Second, we were unable to include participants undergoing gastric sleeve in the current analysis because the sleeve procedure was introduced to HeadsUp after the current project was initiated. Third, despite the relatively high coverage of our metabolomic analysis, we cannot detect all metabolites in the sample. This is due to the complexity of the biochemical properties and the vast range of concentrations of blood metabolites. To date, no single analytical platform can detect all metabolites in a biological sample. Future studies should consider using multiple complementary platforms to increase the coverage of the blood metabolome. Finally, due to the lack of available cohorts with a similar study design and longitudinal metabolomic data, we were unable to validate our results in an independent sample. Further investigation and replication of our findings is warranted. 

## 5. Conclusions

In summary, we found that baseline blood metabolites and their longitudinal changes are associated with differential glycemic outcomes in response to different types of weight-loss intervention, independent of known clinical factors and weight loss. Moreover, weight-loss intervention, especially RYGB, evokes extensive changes in plasma metabolites, and the altered metabolome may underlie the differential glycemic outcomes in response to different types of weight-loss intervention. Our findings may help explain why RYGB often results in better glycemic outcomes than other types of weight-loss intervention. If validated, the identified metabolites may serve as biomarkers for the precise treatment of obesity and diabetes.

## Figures and Tables

**Table 1:** Clinical characteristics of participants before and 1 year after weight-loss intervention.

Characteristics	Intensive Medical Intervention (*n* = 25)				Adjustable Gastric Banding (*n* = 25)				Roux-en-Y Gastric Bypass Surgery (*n* = 25)			
	Baseline	1 Year	Change	*p*-Value	Baseline	1 Year	Change	*p*-Value	Baseline	1 Year	Change	*p*-Value
Age, (year)	51 ± 8	-	-	-	50 ± 8	-	-	-	51 ± 9	-	-	-
Female, n (%)	21 (84)	-	-	-	21 (84)	-	-	-	21 (84)	-	-	
White, n (%)	17 (68)	-	-	-	19 (76)	-	-	-	18 (72)	-	-	-
BMI, kg/m^2^	43.3 ± 6.5	38.3 ± 5.7	−5.0 ± 3.3	5.69 × 10^−8^	45.7 ± 5.3	36.9 ± 5.0	−8.8 ± 3.9	3.13 × 10^−11^	48.5 ± 6.2	31.2 ± 5.0	−17.2 ± 4.8	2.43 × 10^−15^
Weight, kg	120 ± 26	106 ± 21	−14 ± 11	5.69 × 10^−7^	123 ± 16	100 ± 18	−24 ± 10	1.43 × 10^−11^	133 ± 20	86 ± 16	−47 ± 14	4.91 × 10^−15^
WC, cm	123 ± 15	115 ± 13	−7 ± 8	1.69 × 10^−4^	130 ± 10	110 ± 12	−20 ± 9.3	1.39 × 10^−10^	140 ± 11	101 ± 13	−38 ± 12	6.51 × 10^−14^
SBP, mmHg	126 ± 12	120 ± 17	−6 ± 12	3.27 × 10^−2^	129 ± 15	115 ± 13	−14 ± 19	1.05 × 10^−3^	129 ± 14	120 ± 19	−10 ± 20	2.13 × 10^−2^
DBP, mmHg	82 ± 9	80 ± 8	−2 ± 8	1.82 × 10^−1^	81 ± 8	73 ± 7	−8 ± 8	1.26 × 10^−5^	78 ± 11	73 ± 9	−5 ± 12	4.84 × 10^−2^
Total cholesterol, mg/dL	188.4 ± 28.8	188.8 ± 28.9	0.4 ± 30	0.95	185.4 ± 39.9	183.3 ± 40.8	1.1 ± 26	0.83	180 ± 40	180 ± 29	−6.8 ± 36	0.36
FPG, mg/dL	98 [90, 109]	93 [90, 98]	− 3 [−7, 2]	3.18 × 10^−2^	103 [94, 110]	92 [88, 96]	−11 [−15, −4]	6.33 × 10^−4^	118 [98, 155]	92 [84, 96]	− 27 [−58, −6]	6.98 × 10^−4^
HbA1c, %	5.7 [5.5, 6.1]	5.3 [5.2, 5.6]	−0.4 [−0.5, −0.2]	1.53 × 10^−3^	5.9 [5.8, 6.1]	5.4 [5.2, 5.6]	−0.5 [−0.6, −0.3]	4.10 × 10^−5^	7.2 [6.4, 9.1]	5.5 [5.3, 5.8]	−1.3 [−3.1, −0.8]	1.45 × 10^−5^

Continuous variables were expressed as mean ± standard deviation, except FPG and HbA1c as median [Q25, Q75]. Qualitative variables were expressed as n (%). Changes in clinical characteristics were calculated as the measurement at 1-year follow -up minus the baseline value. *p*-values were obtained by paired *t*-test or Wilcoxon rank test if the variables did not follow normal distribution. BMI: body mass index; WC: waist circumference; SBP: systolic blood pressure; DBP: diastolic blood pressure; FPG: fasting plasma glucose; HbA1c: glycated hemoglobin.

**Table 2:** Association between baseline plasma metabolites and change in fasting plasma glucose (FPG) before and 1 year after intervention. Only known metabolites with q < 0.05 are shown.

Metabolites	IMI	BAND	RYGB	RYGB vs. IMI		RYGB vs. BAND		BAND vs. IMI	
	β* (SE)	β* (SE)	β* (SE)	β^†^ (SE)	*p* Value	β^†^ (SE)	*p* Value	β^†^ (SE)	*p* Value
Pyrophosphate	1.74 (3.15)	0.01 (0.09)	17.38 (2.63)	15.64 (4.16)	**3.76** ** × 10^−4^**	17.37 (2.63)	**9.50** ** × 10^−9^**	−1.73 (3.15)	5.85 × 10^−1^
Behenic acid	0.11 (0.12)	−0.46 (0.22)	1.78 (0.37)	1.67 (0.38)	**4.70** ** × 10^−5^**	2.24 (0.43)	**2.36** ** × 10^−6^**	−0.57 (0.25)	2.79 × 10^−2^
3-aminoisobutyric acid	0.13 (0.16)	0.02 (0.14)	2.09 (0.45)	1.96 (0.48)	**1.35** ** × 10^−4^**	2.07 (0.47)	**4.63** ** × 10^−5^**	−0.11 (0.22)	6.09 × 10^−1^
Hydrocinnamic acid	0.09 (0.27)	−0.12 (0.15)	0.88 (0.44)	1.79 (0.51)	**8.37** ** × 10^−4^**	2.00 (0.46)	**4.92** ** × 10^−5^**	−0.21 (0.31)	5.00 × 10^−1^
Gluconic acid	0.11 (0.27)	0.14 (0.19)	−0.89 (0.14)	−1.00 (0.30)	**1.48** ** × 10^−3^**	−1.02 (0.24)	**5.27** ** × 10^−5^**	0.03 (0.32)	9.34 × 10^−1^
Butane-2,3-diol	0.00 (0.16)	−0.33 (0.18)	5.21 (1.28)	5.21 (1.28)	**1.30** ** × 10^−4^**	5.53 (1.29)	**5.97** ** × 10^−5^**	−0.32 (0.24)	1.80 × 10^−1^
Methionine	0.01 (0.20)	−0.05 (0.22)	1.76 (0.45)	1.75 (0.49)	**7.16** ** × 10^−4^**	1.81 (0.50)	**6.16** ** × 10^−4^**	−0.06 (0.30)	8.51 × 10^−1^
Creatinine	0.09 (0.17)	−0.01 (0.18)	1.15 (0.31)	1.06 (0.34)	**2.92** ** × 10^−3^**	1.16 (0.35)	**1.37** ** × 10^−3^**	−0.10 (0.23)	6.73 × 10^−1^
Glucose	−0.09 (0.16)	−0.13 (0.28)	−0.86 (0.14)	−0.78 (0.21)	**4.83** ** × 10^−4^**	−0.73 (0.32)	2.38 × 10^−2^	−0.05 (0.33)	8.83 × 10^−1^
Alanine	−0.10 (0.19)	0.15 (0.20)	0.75 (0.17)	0.85 (0.26)	**1.55** ** × 10^−3^**	0.60 (0.27)	2.67 × 10^−2^	0.25 (0.27)	3.65 × 10^−1^
Allantoin	1.39 (2.19)	0.11 (0.17)	0.95 (0.21)	−0.44 (2.20)	8.43 × 10^−1^	0.84 (0.27)	**2.66** ** × 10^−3^**	−1.27 (2.20)	5.64 × 10^−1^
Galactonic acid	−0.01 (0.26)	0.13 (0.20)	−0.79 (0.15)	−0.78 (0.30)	1.03 × 10^−2^	−0.92 (0.25)	**4.01** ** × 10^−4^**	−0.14 (0.32)	6.68 × 10^−1^
Glutamine	0.05 (0.17)	−0.08 (0.20)	0.93 (0.24)	0.88 (0.30)	4.54 × 10^−3^	1.01 (0.32)	**2.38** ** × 10^−3^**	0.13 (0.25)	6.08 × 10^−1^
Mannose	−0.09 (0.21)	0.12 (0.22)	−0.76 (0.16)	−0.67 (0.26)	1.23 × 10^−2^	−0.88 (0.27)	**1.94** ** × 10^−3^**	−0.21 (0.29)	4.81 × 10^−1^
N-methylalanine	0.04 (0.17)	−0.23 (0.20)	0.75 (0.24)	0.71 (0.29)	1.71 × 10^−2^	0.98 (0.31)	**2.83** ** × 10^−3^**	−0.27 (0.26)	3.12 × 10^−1^

Regression coefficient β* (standard error (SE)) denotes the association between baseline level of each metabolite and change in FPG in each intervention group (IMI, BAND or RYGB). β^†^ (SE) denotes the association between baseline level of each metabolite and change in FPG induced by different types of weight-loss interventions (BAND vs. IMI, RYGB vs. IMI, and RYGB vs. BAND), and was obtained from the interaction term (metabolite × types of intervention) in a linear regression model. In this model, % change in FPG (follow-up value minus baseline value, divided by baseline value) was the dependent variable, baseline level of each metabolite, intervention groups (1 = IMI, 2 = BAND, 3 = RYGB, with IMI being the reference), and an interaction term (metabolite × types of intervention) were the independent variables, adjusting for baseline age, sex, BMI, as well as weight loss. Abbreviations: IMI: intensive medical intervention. BAND: adjustable gastric banding. RYGB: Roux-en-Y gastric bypass surgery. Bold numbers: metabolites remained to be significant at q < 0.05.

**Table 3:** Association between baseline plasma metabolites and change in HbA1c before and 1 year after intervention. Only known metabolites with q < 0.05 are shown.

Metabolites	IMI	BAND	RYGB	RYGB vs. IMI		RYGB vs. BAND		BAND vs. IMI	
	β* (SE)	β* (SE)	β* (SE)	β^†^ (SE)	*p* Value	β^†^ (SE)	*p* Value	β^†^ (SE)	*p* Value
1,5-anhydroglucitol	−0.14 (0.15)	0.17 (0.13)	0.83 (0.14)	0.97 (0.21)	**1.44** ** × 10^−5^**	0.66 (0.19)	**9.77** ** × 10^−4^**	0.31 (0.20)	1.19 × 10^−1^
Hydroxylamine	0.02 (0.14)	0.10 (0.13)	0.83 (0.19)	0.81 (0.23)	**6.37** ** × 10^−4^**	0.73 (0.22)	1.70 × 10^−3^	0.08 (0.18)	6.58 × 10^−1^
Nicotinic acid	0.02 (0.12)	0.13 (0.14)	0.93 (0.23)	0.90 (0.26)	**7.76** ** × 10^−4^**	0.80 (0.27)	4.07 × 10^−3^	0.11 (0.18)	5.50 × 10^−1^
Phosphoethanolamine	−0.02 (0.13)	0.09 (0.15)	0.67 (0.17)	0.69 (0.20)	**1.16** ** × 10^−3^**	0.58 (0.22)	1.04 × 10^−2^	0.11 (0.19)	5.51 × 10^−1^
Allantoin	−0.63 (1.63)	−0.05 (0.13)	0.77 (0.15)	1.41 (1.64)	3.94 × 10^−1^	0.83 (0.20)	**9.99** ** × 10^−5^**	0.58 (1.64)	7.24 × 10^−1^
Pyrophosphate	−0.02 (2.76)	−0.01 (0.08)	9.58 (2.31)	9.59 (3.65)	1.08 × 10^−2^	9.59 (2.31)	**1.00** ** × 10^−4^**	0.00 (2.77)	9.99 × 10^−1^

Regression coefficient β* (standard error (SE)) denotes the association between baseline level of each metabolite and change in HbA1c in each intervention group (IMI, BAND or RYGB). β^†^ (SE) denotes the association between baseline level of each metabolite and change in HbA1c induced by different types of weight-loss interventions (BAND vs. IMI, RYGB vs. IMI, and RYGB vs. BAND), and was obtained from the interaction term (metabolite × types of intervention) in a linear regression model. In this model, % change in HbA1c (follow-up value minus baseline value, divided by baseline value) was the dependent variable, baseline level of each metabolite, intervention groups (1 = IMI, 2 = BAND, 3 = RYGB, with IMI being the reference), and an interaction term (metabolite × types of intervention) were the independent variables, adjusting for baseline age, sex, BMI, as well as weight loss. Abbreviations: IMI: intensive medical intervention. BAND: adjustable gastric banding. RYGB: Roux-en-Y gastric bypass surgery. Bold numbers: metabolites remained to be at q < 0.05.

## Data Availability

Clinical data used in this study can be requested through the HEADSUP Study (PI: Dr. Phillip J. Brantley). Metabolomic data used in this study can be requested from the corresponding author upon a reasonable request.

## Supplementary Materials

The following supporting information can be downloaded at: https://www.mdpi.com/article/10.3390/metabo14020116/s1, Figure S1: Principal component analysis shows that there are no clear batches in our metabolomic data; Figure S2: Statistical Analysis flowchart; Figure S3: Heatmap showing the longitudinal association between changes in plasma metabolites and changes in fasting plasma glucose in response to different weight-loss interventions; Figure S4: Membership of co-regulated metabolites modules identified by WGCNA in the RYGB group (A), the BAND group (B) and the IMI group (C); Figure S5: Differential metabolites networks (modules) associated with different types of weight-loss interventions; Figure S6: Enrichment analysis showing that metabolites identified in the differential network analysis are enriched in metabolic pathways. Table S1: A full list of baseline plasma metabolites associated with change in FPG before and 1-yr after intervention; Table S2: A full list of baseline plasma metabolites associated with change in HbA1c before and 1-yr after intervention; Table S3: Differential association between changes in metabolites and change in FPG before and 1-yr after intervention; Table S4: Differential association between changes in metabolites and change in HbA1c before and 1-yr after intervention; Table S5: Metabolite modules identified by WGCNA among participants who received RYGB (n = 25); Table S6: Metabolite modules identified by WGCNA among participants who received IMI (n = 25); Table S7: Metabolite modules identified by WGCNA among participants who received BAND (n = 25); Table S8: Module differentially connectivity analysis (RYGB vs. IMI); Table S9: Module differentially connectivity analysis (RYGB vs. BAND); Table S10: List of metabolites included in the enrichment analysis; Table S11: List of background metabolites included in the enrichment analysis; Table S12: Pathway enrichment for metabolites in the blue module (RYGB vs. BAND).
