# Supplementary material for: Longitudinal Profiling of Fasting Plasma Metabolome in Response to Weight-Loss Interventions in Patients with Morbid Obesity
Source: Metabolites. 2024 Feb 10;14(2):116. doi: 10.3390/metabo14020116 (PMC10890440; doi:10.3390/metabo14020116)
Supplement: Supplementary file 1 [file metabolites-14-00116-s001.zip › metabolites-2839646-supplementary.pdf]

Supplementary Tables

Table S1. A full list of baseline plasma metabolites associated with change in FPG before and 1-yr after intervention

Table S2. A full list of baseline plasma metabolites associated with change in HbA1c before and 1-yr after intervention

Table S3. Differential association between changes in metabolites and change in FPG before and 1-yr after intervention

Table S4. Differential association between changes in metabolites and change in HbA1c before and 1-yr after intervention

Table S5. Metabolite modules identified by WGCNA among participants who received RYGB (n=25)

Table S6. Metabolite modules identified by WGCNA among participants who received IMI (n=25)

Table S7. Metabolite modules identified by WGCNA among participants who received BAND (n=25)

Table S8. Module differentially connectivity analysis (MDC) (RYGB vs IMI)

Table S9. Module differentially connectivity analysis (MDC) (RYGB vs BAND)

Table S10. List of metabolites included in the enrichment analysis

Table S11. List of background metabolites included in the enrichment analysis

Table S12. Pathway enrichment for metabolites in the blue module (RYGB vs BAND)

Table S1. A full list of baseline plasma metabolites associated with change in FPG before and 1-yr after intervention

	β*(SE)			β*(SE)			p-value			q-value		
**Metabolites**	**IMI**	**BAND**	**RYGB**	**BAND *vs.* IMI**	**RYGB *vs.* IMI**	**RYGB *vs.* BAND**	**BAND *vs.* IMI**	**RYGB *vs.* IMI**	**RYGB *vs.* BAND**	**BAND *vs.* IMI**	**RYGB *vs.* IMI**	**RYGB *vs.*BAND**
**Known**												
Behenic acid	0.11(0.12)	-0.46(0.22)	1.78(0.37)	-0.57(0.25)	1.67(0.38)	2.24(0.43)	2.79E-02	4.70E-05	2.36E-06	9.98E-01	4.43E-03	3.00E-04
Butane-2,3-diol NIST	0.00(0.16)	-0.33(0.18)	5.21(1.28)	-0.32(0.24)	5.21(1.28)	5.53(1.29)	1.80E-01	1.30E-04	5.97E-05	9.98E-01	6.36E-03	3.22E-03
3-aminoisobutyric acid	0.13(0.16)	0.02(0.14)	2.09(0.45)	-0.11(0.22)	1.96(0.48)	2.07(0.47)	6.09E-01	1.35E-04	4.63E-05	9.98E-01	6.36E-03	3.22E-03
Pyrophosphate	1.74(3.15)	0.01(0.09)	17.38(2.63)	-1.73(3.15)	15.64(4.16)	17.37(2.63)	5.85E-01	3.76E-04	9.50E-09	9.98E-01	1.29E-02	3.58E-06
Glucose	-0.09(0.16)	-0.13(0.28)	-0.86(0.14)	-0.05(0.33)	-0.78(0.21)	-0.73(0.32)	8.83E-01	4.83E-04	2.38E-02	9.98E-01	1.52E-02	1.49E-01
Methionine	0.01(0.20)	-0.05(0.22)	1.76(0.45)	-0.06(0.30)	1.75(0.49)	1.81(0.50)	8.51E-01	7.16E-04	6.16E-04	9.98E-01	2.08E-02	1.66E-02
Hydrocinnamic acid	0.09(0.27)	-0.12(0.15)	1.88(0.44)	-0.21(0.31)	1.79(0.51)	2.00(0.46)	5.00E-01	8.37E-04	4.92E-05	9.98E-01	2.25E-02	3.22E-03
Gluconic acid	0.11(0.27)	0.14(0.19)	-0.89(0.14)	0.03(0.32)	-1.00(0.30)	-1.02(0.24)	9.34E-01	1.48E-03	5.27E-05	9.98E-01	2.92E-02	3.22E-03
Alanine	-0.10(0.19)	0.15(0.20)	0.75(0.17)	0.25(0.27)	0.85(0.26)	0.60(0.27)	3.65E-01	1.55E-03	2.67E-02	9.98E-01	2.92E-02	1.60E-01
Creatinine	0.09(0.17)	-0.01(0.18)	1.15(0.31)	-0.10(0.23)	1.06(0.34)	1.16(0.35)	6.73E-01	2.92E-03	1.37E-03	9.98E-01	4.24E-02	3.44E-02
Arachidic acid	-0.21(0.20)	0.24(0.22)	0.60(0.17)	0.45(0.30)	0.81(0.27)	0.35(0.28)	1.41E-01	3.73E-03	2.14E-01	9.98E-01	5.02E-02	6.33E-01
Glutamine	0.05(0.17)	-0.08(0.20)	0.93(0.24)	-0.13(0.25)	0.88(0.30)	1.01(0.32)	6.08E-01	4.54E-03	2.38E-03	9.98E-01	5.55E-02	4.09E-02
Valine	-0.05(0.21)	0.11(0.18)	0.80(0.21)	0.16(0.28)	0.85(0.30)	0.69(0.28)	5.66E-01	5.66E-03	1.60E-02	9.98E-01	6.28E-02	1.24E-01
UDP GlcNAc	0.10(0.16)	-0.01(0.21)	0.87(0.25)	-0.11(0.27)	0.77(0.29)	0.88(0.33)	6.89E-01	9.11E-03	1.03E-02	9.98E-01	8.80E-02	9.87E-02
Galactonic acid	-0.01(0.26)	0.13(0.20)	-0.79(0.15)	0.14(0.32)	-0.78(0.30)	-0.92(0.25)	6.68E-01	1.03E-02	4.01E-04	9.98E-01	9.21E-02	1.37E-02
Glycine	0.16(0.16)	0.11(0.23)	0.78(0.17)	-0.04(0.28)	0.62(0.24)	0.66(0.28)	8.83E-01	1.18E-02	1.96E-02	9.98E-01	1.03E-01	1.33E-01
Mannose	-0.09(0.21)	0.12(0.22)	-0.76(0.16)	0.21(0.29)	-0.67(0.26)	-0.88(0.27)	4.81E-01	1.23E-02	1.94E-03	9.98E-01	1.05E-01	4.07E-02
N-methylalanine	0.04(0.17)	-0.23(0.20)	0.75(0.24)	-0.27(0.26)	0.71(0.29)	0.98(0.31)	3.12E-01	1.71E-02	2.83E-03	9.98E-01	1.29E-01	4.45E-02
Salicylic acid	-0.08(0.56)	0.01(0.12)	4.44(1.77)	0.09(0.57)	4.52(1.85)	4.44(1.77)	8.80E-01	1.71E-02	1.49E-02	9.98E-01	1.29E-01	1.22E-01
Histidine	0.09(0.17)	-0.20(0.23)	0.80(0.24)	-0.29(0.27)	0.71(0.29)	0.99(0.33)	2.98E-01	1.84E-02	3.84E-03	9.98E-01	1.36E-01	5.36E-02
Glycerol-alpha-phosphate	0.07(0.19)	0.15(0.19)	0.71(0.21)	0.08(0.26)	0.65(0.27)	0.56(0.28)	7.50E-01	2.11E-02	4.83E-02	9.98E-01	1.52E-01	2.48E-01

2-hydroxypyrazinyl-2-propenoic acid ethyl ester N	0.10(0.16)	0.10(0.22)	0.71(0.21)	-0.01(0.27)	0.60(0.27)	0.61(0.31)	9.80E-01	2.78E-02	5.10E-02	9.98E-01	1.77E-01	2.50E-01
Phosphate	0.10(0.17)	0.32(0.20)	0.72(0.24)	0.22(0.25)	0.62(0.29)	0.40(0.30)	3.81E-01	3.41E-02	1.89E-01	9.98E-01	2.11E-01	6.18E-01
Proline	0.04(0.22)	0.00(0.18)	0.66(0.21)	-0.05(0.28)	0.61(0.30)	0.66(0.28)	8.59E-01	4.68E-02	2.04E-02	9.98E-01	2.59E-01	1.35E-01
Fructose	0.16(0.37)	0.20(0.32)	-0.62(0.16)	0.03(0.47)	-0.79(0.40)	-0.82(0.37)	9.42E-01	5.33E-02	2.84E-02	9.98E-01	2.87E-01	1.62E-01
Hippuric acid	0.31(0.39)	0.13(0.13)	-0.71(0.36)	-0.18(0.42)	-1.02(0.52)	-0.84(0.38)	6.77E-01	5.70E-02	3.13E-02	9.98E-01	2.96E-01	1.76E-01
Pinitol	0.27(0.27)	0.91(0.44)	1.43(0.54)	0.64(0.51)	1.16(0.60)	0.52(0.70)	2.18E-01	5.80E-02	4.57E-01	9.98E-01	2.96E-01	8.50E-01
Uridine	0.00(0.15)	0.38(0.27)	0.67(0.32)	0.38(0.32)	0.67(0.35)	0.29(0.43)	2.37E-01	5.98E-02	4.96E-01	9.98E-01	3.00E-01	8.74E-01
Pelargonic acid	0.14(0.19)	0.10(0.21)	0.66(0.21)	-0.04(0.28)	0.52(0.28)	0.56(0.30)	8.84E-01	7.03E-02	6.44E-02	9.98E-01	3.40E-01	2.89E-01
1-monostearin	0.08(0.15)	-0.14(0.26)	0.70(0.31)	-0.22(0.30)	0.62(0.35)	0.84(0.41)	4.65E-01	7.80E-02	4.29E-02	9.98E-01	3.72E-01	2.28E-01
Hypoxanthine	-0.01(0.24)	0.00(0.19)	0.55(0.21)	0.01(0.30)	0.57(0.32)	0.56(0.28)	9.74E-01	8.26E-02	5.09E-02	9.98E-01	3.80E-01	2.50E-01
Aconitic acid	0.01(0.25)	0.26(0.23)	-0.50(0.15)	0.25(0.34)	-0.51(0.29)	-0.75(0.27)	4.69E-01	8.81E-02	8.03E-03	9.98E-01	3.95E-01	9.17E-02
Leucine	-0.06(0.24)	0.25(0.19)	0.44(0.22)	0.32(0.30)	0.50(0.32)	0.19(0.28)	2.89E-01	1.15E-01	5.10E-01	9.98E-01	4.83E-01	8.85E-01
Isoleucine	-0.08(0.26)	0.23(0.19)	0.45(0.22)	0.31(0.32)	0.53(0.34)	0.22(0.28)	3.26E-01	1.20E-01	4.43E-01	9.98E-01	4.98E-01	8.48E-01
Adenosine-5-monophosphate	0.06(0.18)	0.02(0.20)	0.57(0.27)	-0.05(0.26)	0.50(0.32)	0.55(0.33)	8.60E-01	1.23E-01	1.03E-01	9.98E-01	4.98E-01	4.16E-01
N-acetylornithine	0.07(0.31)	0.19(0.18)	-0.51(0.23)	0.12(0.35)	-0.58(0.38)	-0.70(0.28)	7.35E-01	1.27E-01	1.56E-02	9.98E-01	5.09E-01	1.24E-01
Dodecanol	0.05(0.17)	0.21(0.21)	0.48(0.23)	0.15(0.27)	0.43(0.29)	0.27(0.31)	5.70E-01	1.42E-01	3.80E-01	9.98E-01	5.49E-01	8.10E-01
Phenylethylamine	-0.13(0.24)	0.10(0.21)	0.31(0.18)	0.24(0.32)	0.44(0.30)	0.20(0.27)	4.57E-01	1.43E-01	4.55E-01	9.98E-01	5.49E-01	8.50E-01
Linoleic acid	0.02(0.22)	0.11(0.19)	-0.43(0.22)	0.10(0.29)	-0.45(0.31)	-0.55(0.29)	7.39E-01	1.45E-01	6.39E-02	9.98E-01	5.53E-01	2.89E-01
Ketohexose	-0.03(0.12)	0.59(0.85)	-1.35(0.94)	0.62(0.86)	-1.32(0.95)	-1.94(1.26)	4.72E-01	1.67E-01	1.28E-01	9.98E-01	6.05E-01	4.73E-01
Hydroxylamine	0.12(0.20)	0.15(0.18)	0.56(0.27)	0.03(0.27)	0.44(0.33)	0.41(0.32)	9.21E-01	1.85E-01	2.06E-01	9.98E-01	6.57E-01	6.33E-01
Lactulose	0.34(0.47)	0.05(0.32)	-0.30(0.14)	-0.28(0.56)	-0.64(0.48)	-0.35(0.34)	6.15E-01	1.94E-01	3.07E-01	9.98E-01	6.65E-01	7.41E-01
Beta-alanine	-0.04(0.18)	0.26(0.20)	0.35(0.24)	0.30(0.27)	0.39(0.30)	0.09(0.31)	2.67E-01	1.99E-01	7.78E-01	9.98E-01	6.69E-01	9.32E-01
N-acetylmannosamine	0.03(0.29)	0.00(0.16)	-0.47(0.28)	-0.03(0.33)	-0.51(0.39)	-0.47(0.32)	9.15E-01	2.03E-01	1.46E-01	9.98E-01	6.76E-01	5.20E-01
Conduritol-beta-expoxide	-0.05(0.22)	-0.01(0.18)	0.34(0.22)	0.05(0.29)	0.39(0.31)	0.34(0.28)	8.76E-01	2.11E-01	2.30E-01	9.98E-01	6.78E-01	6.54E-01
Threonine	0.23(0.19)	0.15(0.20)	0.59(0.22)	-0.08(0.27)	0.35(0.29)	0.43(0.30)	7.71E-01	2.28E-01	1.51E-01	9.98E-01	7.22E-01	5.30E-01
Galactose	-0.15(0.20)	-0.33(0.21)	-0.47(0.18)	-0.18(0.29)	-0.32(0.27)	-0.14(0.27)	5.39E-01	2.39E-01	6.15E-01	9.98E-01	7.43E-01	9.16E-01

Benzoic acid	0.32(0.49)	0.16(0.18)	0.96(0.26)	-0.15(0.52)	0.64(0.54)	0.80(0.31)	7.67E-01	2.40E-01	1.32E-02	9.98E-01	7.43E-01	1.16E-01
Methionine sulfoxide	0.20(0.24)	0.13(0.19)	0.56(0.21)	-0.07(0.28)	0.36(0.32)	0.43(0.28)	8.05E-01	2.69E-01	1.32E-01	9.98E-01	8.00E-01	4.84E-01
Oxamic acid	0.12(0.23)	0.06(0.17)	-0.28(0.29)	-0.06(0.28)	-0.40(0.36)	-0.34(0.33)	8.30E-01	2.71E-01	3.09E-01	9.98E-01	8.00E-01	7.41E-01
3-hydroxybutyric acid	0.06(0.27)	0.01(0.20)	-0.30(0.19)	-0.05(0.34)	-0.35(0.32)	-0.30(0.28)	8.76E-01	2.78E-01	2.81E-01	9.98E-01	8.00E-01	7.20E-01
Sucrose	2.91(2.69)	0.04(0.27)	-0.04(0.13)	-2.87(2.71)	-2.94(2.69)	-0.08(0.31)	2.94E-01	2.78E-01	8.06E-01	9.98E-01	8.00E-01	9.43E-01
Phenylalanine	0.05(0.21)	0.21(0.22)	0.35(0.20)	0.15(0.30)	0.30(0.29)	0.15(0.29)	6.16E-01	3.01E-01	6.12E-01	9.98E-01	8.33E-01	9.16E-01
Cysteine-glycine	0.29(0.31)	0.27(0.23)	-0.05(0.16)	-0.02(0.38)	-0.34(0.34)	-0.32(0.27)	9.52E-01	3.19E-01	2.45E-01	9.98E-01	8.52E-01	6.68E-01
Nonadecanoic acid	0.05(0.14)	-0.31(0.45)	-0.24(0.27)	-0.36(0.47)	-0.29(0.30)	0.07(0.52)	4.49E-01	3.34E-01	8.99E-01	9.98E-01	8.75E-01	9.77E-01
Oleic acid	0.02(0.27)	0.05(0.19)	-0.31(0.20)	0.03(0.32)	-0.32(0.34)	-0.36(0.27)	9.20E-01	3.37E-01	1.95E-01	9.98E-01	8.75E-01	6.28E-01
Indole-3-acetate	-0.10(0.22)	-0.03(0.18)	0.46(0.55)	0.06(0.29)	0.56(0.59)	0.49(0.58)	8.22E-01	3.47E-01	3.97E-01	9.98E-01	8.75E-01	8.18E-01
Pyruvic acid	0.03(0.23)	0.19(0.21)	0.31(0.18)	0.16(0.31)	0.27(0.29)	0.12(0.28)	6.14E-01	3.54E-01	6.81E-01	9.98E-01	8.75E-01	9.29E-01
Indole-3-lactate	0.03(0.22)	0.14(0.27)	0.28(0.16)	0.11(0.35)	0.25(0.27)	0.14(0.32)	7.64E-01	3.64E-01	6.50E-01	9.98E-01	8.75E-01	9.22E-01
Tagatose	0.01(0.39)	0.09(0.39)	-0.35(0.15)	0.08(0.55)	-0.37(0.42)	-0.45(0.42)	8.84E-01	3.83E-01	2.90E-01	9.98E-01	9.08E-01	7.23E-01
Lauric acid	0.05(0.25)	0.21(0.18)	0.36(0.26)	0.16(0.31)	0.32(0.36)	0.15(0.32)	6.01E-01	3.89E-01	6.34E-01	9.98E-01	9.11E-01	9.17E-01
2-piperidinobenzonitrile NIST	0.11(0.21)	0.07(0.18)	-0.20(0.31)	-0.04(0.27)	-0.31(0.36)	-0.27(0.36)	8.94E-01	4.03E-01	4.52E-01	9.98E-01	9.26E-01	8.50E-01
2-hydroxyglutaric acid	-0.08(0.22)	0.23(0.22)	0.15(0.18)	0.31(0.31)	0.23(0.28)	-0.08(0.28)	3.25E-01	4.16E-01	7.86E-01	9.98E-01	9.31E-01	9.32E-01
Tyrosine	0.07(0.23)	0.07(0.24)	0.30(0.17)	-0.00(0.33)	0.23(0.29)	0.23(0.29)	9.96E-01	4.30E-01	4.36E-01	9.98E-01	9.31E-01	8.47E-01
Isocitric acid	0.06(0.17)	0.15(0.20)	-0.24(0.34)	0.09(0.26)	-0.29(0.37)	-0.38(0.39)	7.25E-01	4.36E-01	3.25E-01	9.98E-01	9.31E-01	7.43E-01
Deoxycholic acid	-0.03(0.17)	0.15(0.29)	0.19(0.22)	0.17(0.32)	0.21(0.27)	0.04(0.36)	5.90E-01	4.40E-01	9.15E-01	9.98E-01	9.31E-01	9.83E-01
N-acetylglycine NIST	0.09(0.20)	0.03(0.20)	0.32(0.21)	-0.07(0.28)	0.23(0.29)	0.29(0.29)	8.14E-01	4.40E-01	3.18E-01	9.98E-01	9.31E-01	7.41E-01
Cysteine	0.03(0.22)	0.06(0.33)	0.24(0.16)	0.03(0.38)	0.21(0.27)	0.18(0.36)	9.35E-01	4.43E-01	6.19E-01	9.98E-01	9.31E-01	9.16E-01
Kynurenine	0.10(0.14)	0.22(0.36)	0.35(0.29)	0.12(0.39)	0.25(0.32)	0.13(0.48)	7.70E-01	4.44E-01	7.81E-01	9.98E-01	9.31E-01	9.32E-01
Tryptophan	0.01(0.21)	-0.06(0.22)	0.26(0.26)	-0.07(0.29)	0.25(0.33)	0.32(0.34)	8.24E-01	4.46E-01	3.46E-01	9.98E-01	9.31E-01	7.67E-01
N-acetylglutamate	0.11(0.22)	0.08(0.18)	-0.15(0.26)	-0.03(0.28)	-0.26(0.34)	-0.23(0.32)	9.22E-01	4.52E-01	4.71E-01	9.98E-01	9.31E-01	8.54E-01
Xylose	-0.13(0.51)	0.37(0.25)	-0.53(0.20)	0.49(0.56)	-0.41(0.55)	-0.90(0.33)	3.87E-01	4.62E-01	7.84E-03	9.98E-01	9.31E-01	9.17E-02
Palmitoleic acid	0.09(0.37)	0.08(0.17)	-0.22(0.22)	-0.01(0.40)	-0.31(0.43)	-0.30(0.28)	9.72E-01	4.63E-01	2.83E-01	9.98E-01	9.31E-01	7.20E-01

Cystine	-0.02(0.23)	-0.15(0.21)	0.21(0.23)	-0.12(0.31)	0.23(0.32)	0.36(0.32)	6.88E-01	4.75E-01	2.63E-01	9.98E-01	9.31E-01	6.88E-01
1,5-anhydroglucitol	0.17(0.24)	0.24(0.21)	0.40(0.22)	0.07(0.31)	0.23(0.33)	0.17(0.30)	8.32E-01	4.77E-01	5.81E-01	9.98E-01	9.31E-01	9.04E-01
Asparagine	0.17(0.25)	0.06(0.19)	0.38(0.18)	-0.11(0.31)	0.22(0.30)	0.32(0.26)	7.32E-01	4.77E-01	2.18E-01	9.98E-01	9.31E-01	6.33E-01
Myristic acid	0.21(0.25)	0.15(0.17)	0.44(0.23)	-0.06(0.30)	0.23(0.33)	0.30(0.28)	8.31E-01	4.85E-01	3.00E-01	9.98E-01	9.31E-01	7.39E-01
Shikimic acid	-0.03(0.19)	-0.33(0.16)	0.39(0.56)	-0.31(0.24)	0.41(0.61)	0.72(0.59)	2.14E-01	5.01E-01	2.29E-01	9.98E-01	9.31E-01	6.54E-01
Erythritol	0.12(0.30)	0.19(0.28)	-0.10(0.15)	0.07(0.41)	-0.23(0.34)	-0.29(0.31)	8.69E-01	5.04E-01	3.54E-01	9.98E-01	9.31E-01	7.70E-01
Phosphoethanolamine	0.15(0.19)	0.20(0.21)	0.35(0.24)	0.05(0.28)	0.20(0.29)	0.15(0.32)	8.69E-01	5.04E-01	6.33E-01	9.98E-01	9.31E-01	9.17E-01
Hydroxyproline dipeptide NIST	0.06(0.17)	0.13(0.25)	-0.15(0.26)	0.07(0.30)	-0.20(0.31)	-0.28(0.36)	8.08E-01	5.12E-01	4.42E-01	9.98E-01	9.31E-01	8.48E-01
Dehydroabietic acid	0.07(0.22)	0.60(0.43)	0.29(0.25)	0.53(0.48)	0.21(0.33)	-0.32(0.49)	2.75E-01	5.19E-01	5.24E-01	9.98E-01	9.31E-01	8.92E-01
Isothreonic acid	0.03(0.36)	0.40(0.27)	-0.22(0.14)	0.37(0.45)	-0.25(0.40)	-0.62(0.31)	4.14E-01	5.25E-01	4.94E-02	9.98E-01	9.31E-01	2.48E-01
Oxalic acid	0.06(0.20)	0.06(0.18)	-0.19(0.35)	-0.00(0.26)	-0.25(0.39)	-0.24(0.39)	9.98E-01	5.28E-01	5.28E-01	9.98E-01	9.31E-01	8.92E-01
Methanolphosphate	-0.02(0.17)	0.10(0.22)	-0.22(0.27)	0.11(0.28)	-0.20(0.32)	-0.31(0.36)	6.81E-01	5.31E-01	3.82E-01	9.98E-01	9.31E-01	8.10E-01
Threonic acid	0.00(0.31)	0.08(0.17)	-0.23(0.24)	0.08(0.35)	-0.23(0.37)	-0.31(0.29)	8.24E-01	5.34E-01	2.93E-01	9.98E-01	9.31E-01	7.27E-01
Caprylic acid	0.13(0.28)	0.22(0.24)	0.33(0.16)	0.08(0.36)	0.19(0.31)	0.11(0.29)	8.17E-01	5.35E-01	7.00E-01	9.98E-01	9.31E-01	9.29E-01
Citric acid	0.12(0.18)	0.18(0.20)	0.33(0.30)	0.06(0.26)	0.21(0.34)	0.15(0.35)	8.25E-01	5.37E-01	6.67E-01	9.98E-01	9.31E-01	9.22E-01
Oxoproline	0.16(0.22)	0.23(0.21)	0.33(0.19)	0.07(0.29)	0.17(0.28)	0.10(0.28)	8.02E-01	5.39E-01	7.21E-01	9.98E-01	9.31E-01	9.29E-01
2,4-diaminobutyric acid	0.12(0.22)	0.01(0.22)	0.29(0.19)	-0.11(0.30)	0.17(0.28)	0.28(0.29)	7.15E-01	5.53E-01	3.40E-01	9.98E-01	9.31E-01	7.58E-01
Naproxen	0.03(0.18)	0.37(1.46)	-0.12(0.16)	0.34(1.47)	-0.14(0.24)	-0.49(1.47)	8.15E-01	5.55E-01	7.42E-01	9.98E-01	9.31E-01	9.29E-01
Xylitol	0.25(0.21)	0.07(0.17)	0.03(0.30)	-0.17(0.26)	-0.21(0.36)	-0.04(0.33)	5.15E-01	5.56E-01	9.07E-01	9.98E-01	9.31E-01	9.77E-01
Fucose	-0.01(0.26)	0.18(0.23)	-0.20(0.17)	0.19(0.35)	-0.19(0.31)	-0.38(0.28)	5.89E-01	5.58E-01	1.91E-01	9.98E-01	9.31E-01	6.22E-01
4-hydroxyphenylacetic acid	0.18(0.28)	0.10(0.29)	0.00(0.15)	-0.08(0.40)	-0.18(0.32)	-0.11(0.33)	8.44E-01	5.64E-01	7.51E-01	9.98E-01	9.31E-01	9.30E-01
Lysine	0.09(0.20)	0.08(0.24)	0.27(0.24)	-0.00(0.31)	0.18(0.31)	0.18(0.34)	9.88E-01	5.67E-01	5.88E-01	9.98E-01	9.31E-01	9.04E-01
2,3-dihydroxybutanoic acid NIST	0.12(0.17)	0.06(0.26)	-0.04(0.22)	-0.06(0.31)	-0.16(0.28)	-0.10(0.35)	8.52E-01	5.72E-01	7.70E-01	9.98E-01	9.31E-01	9.32E-01
Pyrrole-2-carboxylic acid	0.10(0.19)	0.07(0.19)	-0.10(0.30)	-0.03(0.27)	-0.20(0.35)	-0.17(0.35)	9.02E-01	5.73E-01	6.40E-01	9.98E-01	9.31E-01	9.17E-01
Ornithine	0.11(0.22)	0.07(0.21)	0.28(0.19)	-0.04(0.30)	0.16(0.29)	0.20(0.28)	8.95E-01	5.76E-01	4.62E-01	9.98E-01	9.31E-01	8.50E-01

Glyceric acid	-0.04(0.17)	0.19(0.23)	0.12(0.25)	0.23(0.29)	0.16(0.30)	-0.06(0.33)	4.27E-01	5.85E-01	8.46E-01	9.98E-01	9.42E-01	9.46E-01
Palmitic acid	0.09(0.21)	0.07(0.23)	-0.06(0.20)	-0.02(0.31)	-0.16(0.29)	-0.14(0.31)	9.50E-01	5.90E-01	6.62E-01	9.98E-01	9.46E-01	9.22E-01
Hexitol	-0.12(0.21)	-0.06(0.16)	-0.32(0.32)	0.06(0.27)	-0.20(0.37)	-0.26(0.36)	8.10E-01	5.97E-01	4.62E-01	9.98E-01	9.50E-01	8.50E-01
Lactic acid	-0.16(0.29)	-0.07(0.33)	0.02(0.16)	0.09(0.45)	0.17(0.34)	0.08(0.36)	8.49E-01	6.12E-01	8.16E-01	9.98E-01	9.56E-01	9.44E-01
Arachidonic acid	0.05(0.23)	0.00(0.23)	-0.10(0.18)	-0.05(0.31)	-0.15(0.29)	-0.10(0.29)	8.81E-01	6.13E-01	7.32E-01	9.98E-01	9.56E-01	9.29E-01
Malic acid	0.37(0.49)	0.31(0.64)	0.11(0.12)	-0.06(0.80)	-0.25(0.50)	-0.20(0.65)	9.45E-01	6.15E-01	7.63E-01	9.98E-01	9.56E-01	9.32E-01
3,6-anhydro-D-galactose	-0.09(0.37)	0.11(0.28)	-0.30(0.15)	0.20(0.47)	-0.21(0.41)	-0.41(0.31)	6.75E-01	6.16E-01	1.99E-01	9.98E-01	9.56E-01	6.33E-01
Acetaminophen	0.37(0.48)	0.11(0.19)	0.14(0.17)	-0.25(0.52)	-0.22(0.51)	0.03(0.25)	6.28E-01	6.64E-01	9.04E-01	9.98E-01	9.74E-01	9.77E-01
Ethanolamine	0.14(0.22)	0.39(0.25)	0.02(0.18)	0.25(0.33)	-0.12(0.28)	-0.37(0.31)	4.53E-01	6.65E-01	2.36E-01	9.98E-01	9.74E-01	6.59E-01
1-monopalmitin	0.13(0.23)	0.09(0.17)	0.28(0.24)	-0.04(0.28)	0.14(0.33)	0.18(0.29)	8.78E-01	6.67E-01	5.31E-01	9.98E-01	9.74E-01	8.92E-01
Tocopherol gamma-	0.18(0.22)	0.18(0.20)	0.31(0.26)	0.01(0.29)	0.13(0.33)	0.13(0.32)	9.81E-01	6.91E-01	7.00E-01	9.98E-01	9.74E-01	9.29E-01
Serine	0.22(0.19)	0.16(0.23)	0.33(0.21)	-0.07(0.30)	0.11(0.28)	0.18(0.31)	8.18E-01	6.97E-01	5.69E-01	9.98E-01	9.74E-01	9.04E-01
Parabanic acid NIST	0.11(0.24)	0.04(0.17)	-0.03(0.27)	-0.07(0.28)	-0.14(0.35)	-0.07(0.32)	8.06E-01	7.02E-01	8.36E-01	9.98E-01	9.74E-01	9.46E-01
Tocopherol alpha-	-0.03(0.21)	0.14(0.20)	0.12(0.33)	0.17(0.29)	0.14(0.38)	-0.02(0.38)	5.60E-01	7.06E-01	9.50E-01	9.98E-01	9.74E-01	9.94E-01
2-deoxyisotetronic acid NIST	-0.01(0.20)	0.10(0.25)	-0.11(0.20)	0.10(0.32)	-0.11(0.29)	-0.21(0.32)	7.46E-01	7.09E-01	5.03E-01	9.98E-01	9.74E-01	8.77E-01
Alloxanoic acid NIST	0.14(0.28)	0.05(0.16)	-0.02(0.34)	-0.09(0.31)	-0.16(0.42)	-0.07(0.37)	7.76E-01	7.12E-01	8.55E-01	9.98E-01	9.74E-01	9.51E-01
Pseudo uridine	0.09(0.24)	0.12(0.23)	0.20(0.17)	0.03(0.33)	0.11(0.30)	0.08(0.29)	9.36E-01	7.19E-01	7.79E-01	9.98E-01	9.74E-01	9.32E-01
Succinic acid	0.12(0.18)	0.34(0.28)	0.03(0.21)	0.22(0.32)	-0.09(0.27)	-0.31(0.34)	5.07E-01	7.25E-01	3.67E-01	9.98E-01	9.74E-01	7.92E-01
Quinic acid	0.24(0.22)	0.16(0.19)	0.36(0.25)	-0.08(0.30)	0.12(0.33)	0.20(0.31)	7.87E-01	7.25E-01	5.30E-01	9.98E-01	9.74E-01	8.92E-01
Arabitol	0.20(0.29)	0.06(0.32)	0.08(0.15)	-0.13(0.43)	-0.11(0.33)	0.02(0.35)	7.59E-01	7.29E-01	9.57E-01	9.98E-01	9.74E-01	9.95E-01
Cellobiose minor	0.07(0.18)	0.01(0.24)	0.17(0.23)	-0.07(0.29)	0.10(0.29)	0.16(0.33)	8.25E-01	7.32E-01	6.17E-01	9.98E-01	9.74E-01	9.16E-01
Mannitol	0.07(0.37)	-0.91(0.57)	-0.06(0.13)	-0.98(0.67)	-0.13(0.39)	0.85(0.59)	1.47E-01	7.43E-01	1.52E-01	9.98E-01	9.80E-01	5.30E-01
Pipecolinic acid	0.25(0.27)	-0.07(0.24)	0.15(0.16)	-0.32(0.36)	-0.10(0.31)	0.22(0.28)	3.78E-01	7.53E-01	4.43E-01	9.98E-01	9.82E-01	8.48E-01
Pentadecanoic acid	-0.29(0.23)	-0.04(0.25)	-0.20(0.17)	0.25(0.34)	0.09(0.28)	-0.16(0.30)	4.67E-01	7.65E-01	5.83E-01	9.98E-01	9.92E-01	9.04E-01
Trans-4-hydroxyproline	0.19(0.25)	-0.02(0.24)	0.28(0.17)	-0.21(0.34)	0.09(0.30)	0.30(0.29)	5.37E-01	7.73E-01	3.10E-01	9.98E-01	9.94E-01	7.41E-01
Glutamic acid	-0.02(0.20)	-0.17(0.25)	-0.10(0.19)	-0.15(0.31)	-0.08(0.27)	0.07(0.31)	6.40E-01	7.83E-01	8.16E-01	9.98E-01	9.94E-01	9.44E-01
Sorbitol	0.02(0.41)	-0.11(4.31)	-0.09(0.13)	-0.13(4.32)	-0.12(0.43)	0.01(4.31)	9.76E-01	7.86E-01	9.97E-01	9.98E-01	9.94E-01	9.98E-01

Cholesterol	0.17(0.35)	0.20(0.15)	0.29(0.29)	0.04(0.38)	0.12(0.45)	0.08(0.33)	9.21E-01	7.91E-01	8.08E-01	9.98E-01	9.94E-01	9.43E-01
Myo-inositol	0.13(0.28)	0.20(0.28)	0.05(0.16)	0.07(0.39)	-0.08(0.32)	-0.15(0.32)	8.64E-01	7.96E-01	6.44E-01	9.98E-01	9.94E-01	9.20E-01
Capric acid	0.30(0.42)	0.27(0.23)	0.18(0.17)	-0.03(0.48)	-0.11(0.45)	-0.08(0.28)	9.48E-01	7.98E-01	7.70E-01	9.98E-01	9.94E-01	9.32E-01
2-deoxytetronic acid	0.26(0.21)	0.34(0.24)	0.33(0.20)	0.08(0.31)	0.07(0.29)	-0.01(0.31)	7.86E-01	8.02E-01	9.72E-01	9.98E-01	9.94E-01	9.95E-01
Allantoic acid	-0.04(0.20)	-0.22(0.27)	0.09(0.51)	-0.18(0.34)	0.13(0.55)	0.32(0.58)	5.90E-01	8.10E-01	5.86E-01	9.98E-01	9.94E-01	9.04E-01
2-hydroxyvaleric acid	0.09(0.37)	-0.10(0.21)	-0.01(0.22)	-0.19(0.43)	-0.10(0.43)	0.09(0.30)	6.59E-01	8.24E-01	7.55E-01	9.98E-01	9.94E-01	9.30E-01
Glycolic acid	0.03(0.16)	0.05(0.30)	0.11(0.30)	0.02(0.33)	0.07(0.34)	0.05(0.43)	9.48E-01	8.29E-01	9.04E-01	9.98E-01	9.94E-01	9.77E-01
UDP-glucuronic acid	0.31(0.70)	-0.02(0.38)	0.17(0.13)	-0.33(0.80)	-0.14(0.72)	0.19(0.40)	6.82E-01	8.43E-01	6.37E-01	9.98E-01	9.94E-01	9.17E-01
Allantoin	1.39(2.19)	0.11(0.17)	0.95(0.21)	-1.27(2.20)	-0.44(2.20)	0.84(0.27)	5.64E-01	8.43E-01	2.66E-03	9.98E-01	9.94E-01	4.36E-02
Maltose	0.04(0.17)	0.10(0.25)	0.10(0.22)	0.06(0.30)	0.06(0.28)	-0.01(0.34)	8.32E-01	8.44E-01	9.82E-01	9.98E-01	9.94E-01	9.95E-01
Maltotriose	0.14(0.26)	0.05(0.19)	0.20(0.20)	-0.09(0.31)	0.06(0.32)	0.15(0.27)	7.83E-01	8.50E-01	5.91E-01	9.98E-01	9.94E-01	9.06E-01
2-hydroxybutanoic acid	-0.33(0.26)	-0.14(0.21)	-0.38(0.17)	0.19(0.33)	-0.06(0.31)	-0.24(0.28)	5.79E-01	8.56E-01	3.90E-01	9.98E-01	9.94E-01	8.12E-01
Aspartic acid	0.12(0.24)	0.16(0.18)	0.06(0.24)	0.04(0.30)	-0.06(0.34)	-0.11(0.30)	8.82E-01	8.57E-01	7.23E-01	9.98E-01	9.94E-01	9.29E-01
Levoglucosan	0.24(0.35)	0.02(0.20)	0.17(0.16)	-0.22(0.40)	-0.07(0.39)	0.15(0.26)	5.91E-01	8.61E-01	5.63E-01	9.98E-01	9.94E-01	9.04E-01
Urea	0.29(0.26)	0.05(0.16)	0.23(0.23)	-0.24(0.31)	-0.06(0.35)	0.18(0.28)	4.44E-01	8.68E-01	5.22E-01	9.98E-01	9.94E-01	8.92E-01
Ribonic acid	0.02(0.50)	0.17(0.20)	-0.07(0.21)	0.15(0.54)	-0.09(0.54)	-0.24(0.29)	7.78E-01	8.69E-01	4.09E-01	9.98E-01	9.94E-01	8.25E-01
Lyxitol	0.07(0.21)	-0.05(0.24)	0.11(0.19)	-0.12(0.32)	0.04(0.28)	0.16(0.31)	7.04E-01	8.88E-01	6.07E-01	9.98E-01	9.94E-01	9.16E-01
Nicotinic acid	0.06(0.18)	0.12(0.21)	0.11(0.34)	0.05(0.26)	0.04(0.38)	-0.01(0.39)	8.47E-01	9.12E-01	9.81E-01	9.98E-01	9.94E-01	9.95E-01
3-(1-pyrazolyl)-L-alanine	0.13(0.40)	0.11(0.72)	0.09(0.13)	-0.01(0.81)	-0.04(0.41)	-0.02(0.73)	9.85E-01	9.28E-01	9.75E-01	9.98E-01	9.94E-01	9.95E-01
Fumaric acid	0.08(0.19)	0.18(0.21)	0.06(0.27)	0.09(0.28)	-0.03(0.32)	-0.12(0.33)	7.33E-01	9.38E-01	7.22E-01	9.98E-01	9.94E-01	9.29E-01
Heptadecanoic acid	0.07(0.20)	-0.04(0.25)	0.09(0.19)	-0.11(0.33)	0.02(0.28)	0.13(0.32)	7.40E-01	9.40E-01	6.86E-01	9.98E-01	9.94E-01	9.29E-01
Uric acid	-0.07(0.23)	0.18(0.27)	-0.05(0.20)	0.25(0.35)	0.02(0.31)	-0.23(0.33)	4.80E-01	9.53E-01	4.89E-01	9.98E-01	9.94E-01	8.70E-01
Taurine	-0.02(0.27)	0.19(0.20)	-0.04(0.19)	0.21(0.33)	-0.01(0.33)	-0.22(0.27)	5.32E-01	9.69E-01	4.21E-01	9.98E-01	9.94E-01	8.40E-01
Aminomalonate	0.11(0.18)	0.27(0.25)	0.12(0.21)	0.16(0.30)	0.01(0.28)	-0.15(0.32)	5.93E-01	9.79E-01	6.33E-01	9.98E-01	9.94E-01	9.17E-01
Glycerol	0.11(0.20)	0.17(0.20)	0.11(0.26)	0.06(0.27)	-0.01(0.31)	-0.07(0.31)	8.23E-01	9.86E-01	8.30E-01	9.98E-01	9.94E-01	9.46E-01
Stearic acid	0.13(0.21)	-0.02(0.25)	0.14(0.20)	-0.15(0.33)	0.00(0.28)	0.16(0.32)	6.37E-01	9.88E-01	6.21E-01	9.98E-01	9.94E-01	9.16E-01
Hexuronic acid	0.00(0.43)	0.25(0.31)	-0.01(0.16)	0.26(0.53)	-0.01(0.45)	-0.26(0.35)	6.32E-01	9.89E-01	4.54E-01	9.98E-01	9.94E-01	8.50E-01
Isothreitol	0.16(0.32)	0.39(0.29)	0.17(0.14)	0.23(0.43)	0.00(0.34)	-0.22(0.32)	6.01E-01	9.94E-01	4.86E-01	9.98E-01	9.94E-01	8.70E-01

**Unknown**												
135869	0.02(0.15)	-0.25(0.34)	-1.05(0.14)	-0.27(0.37)	-1.07(0.21)	-0.80(0.37)	4.64E-01	3.82E-06	3.71E-02	9.98E-01	1.44E-03	2.06E-01
41689	0.02(0.14)	0.04(0.33)	-1.15(0.20)	0.02(0.36)	-1.17(0.25)	-1.19(0.39)	9.53E-01	1.13E-05	3.36E-03	9.98E-01	2.12E-03	5.00E-02
6646	-0.01(0.16)	-0.37(0.36)	-0.95(0.13)	-0.36(0.39)	-0.94(0.21)	-0.58(0.39)	3.66E-01	2.38E-05	1.42E-01	9.98E-01	3.00E-03	5.11E-01
6066	0.04(0.18)	-0.10(0.16)	1.02(0.15)	-0.14(0.24)	0.98(0.23)	1.12(0.22)	5.66E-01	8.46E-05	2.39E-06	9.98E-01	6.36E-03	3.00E-04
87756	0.05(0.14)	0.01(0.21)	1.41(0.30)	-0.04(0.24)	1.36(0.33)	1.41(0.36)	8.59E-01	1.05E-04	2.33E-04	9.98E-01	6.36E-03	9.78E-03
88583	-0.06(0.16)	-0.12(0.19)	0.88(0.18)	-0.06(0.25)	0.94(0.24)	1.00(0.26)	8.16E-01	2.06E-04	2.71E-04	9.98E-01	8.63E-03	1.02E-02
64546	-0.01(0.17)	-0.06(0.30)	-0.83(0.14)	-0.04(0.35)	-0.82(0.22)	-0.78(0.33)	8.98E-01	3.69E-04	2.15E-02	9.98E-01	1.29E-02	1.40E-01
16550	0.09(0.15)	0.10(0.19)	1.38(0.34)	0.01(0.24)	1.29(0.37)	1.28(0.39)	9.59E-01	9.38E-04	1.68E-03	9.98E-01	2.36E-02	3.73E-02
127277	-0.01(0.16)	-0.33(0.18)	4.43(1.29)	-0.32(0.24)	4.43(1.29)	4.75(1.30)	1.92E-01	1.03E-03	5.08E-04	9.98E-01	2.43E-02	1.47E-02
41683	-0.04(0.17)	-0.04(0.40)	-0.82(0.15)	-0.01(0.44)	-0.78(0.23)	-0.78(0.43)	9.89E-01	1.24E-03	7.72E-02	9.98E-01	2.74E-02	3.27E-01
132379	0.08(0.19)	0.21(0.19)	0.96(0.19)	0.13(0.27)	0.88(0.27)	0.75(0.27)	6.13E-01	1.55E-03	7.36E-03	9.98E-01	2.92E-02	9.17E-02
135260	0.16(0.16)	0.31(0.20)	0.90(0.16)	0.15(0.25)	0.74(0.23)	0.59(0.26)	5.61E-01	1.79E-03	2.52E-02	9.98E-01	3.22E-02	1.56E-01
62	0.04(0.19)	0.02(0.20)	0.81(0.15)	-0.02(0.27)	0.77(0.24)	0.79(0.25)	9.44E-01	2.30E-03	2.18E-03	9.98E-01	3.94E-02	4.09E-02
131620	0.02(0.18)	-0.01(0.17)	0.89(0.21)	-0.02(0.25)	0.87(0.28)	0.89(0.27)	9.29E-01	2.58E-03	1.64E-03	9.98E-01	4.15E-02	3.73E-02
112487	0.09(0.17)	0.16(0.23)	0.83(0.16)	0.07(0.28)	0.74(0.24)	0.67(0.28)	8.00E-01	2.70E-03	1.83E-02	9.98E-01	4.15E-02	1.30E-01
135610	0.03(0.19)	-0.01(0.27)	-0.73(0.15)	-0.04(0.34)	-0.76(0.24)	-0.72(0.32)	9.13E-01	2.75E-03	2.59E-02	9.98E-01	4.15E-02	1.58E-01
95550	0.02(0.17)	0.25(0.24)	0.75(0.17)	0.23(0.30)	0.72(0.24)	0.49(0.30)	4.45E-01	3.33E-03	1.05E-01	9.98E-01	4.65E-02	4.23E-01
7718	0.01(0.13)	-0.03(0.31)	1.52(0.50)	-0.03(0.33)	1.51(0.51)	1.54(0.58)	9.22E-01	4.34E-03	1.01E-02	9.98E-01	5.55E-02	9.87E-02
2001	-0.08(0.23)	-0.15(0.17)	0.77(0.18)	-0.07(0.28)	0.85(0.29)	0.91(0.25)	8.14E-01	4.56E-03	4.50E-04	9.98E-01	5.55E-02	1.41E-02
6115	0.06(0.17)	0.03(0.20)	-0.90(0.29)	-0.03(0.26)	-0.96(0.33)	-0.93(0.35)	9.05E-01	5.26E-03	9.29E-03	9.98E-01	6.19E-02	9.73E-02
1709	0.02(0.17)	0.03(0.22)	0.74(0.19)	0.01(0.28)	0.72(0.25)	0.71(0.29)	9.58E-01	5.65E-03	1.82E-02	9.98E-01	6.28E-02	1.30E-01
5990	0.02(0.18)	0.16(0.16)	1.11(0.34)	0.13(0.24)	1.09(0.39)	0.95(0.38)	5.80E-01	6.69E-03	1.47E-02	9.98E-01	7.21E-02	1.22E-01
16792	-0.01(0.16)	-0.36(0.23)	-0.81(0.24)	-0.36(0.28)	-0.81(0.29)	-0.45(0.33)	2.09E-01	7.32E-03	1.77E-01	9.98E-01	7.67E-02	6.02E-01
159824	0.05(0.20)	0.13(0.18)	0.79(0.18)	0.08(0.27)	0.73(0.27)	0.65(0.25)	7.69E-01	8.50E-03	1.19E-02	9.98E-01	8.44E-02	1.07E-01
62433	0.11(0.18)	0.24(0.19)	0.80(0.18)	0.13(0.26)	0.68(0.26)	0.55(0.26)	6.20E-01	9.43E-03	3.76E-02	9.98E-01	8.89E-02	2.06E-01
146957	0.03(0.19)	0.05(0.17)	0.79(0.21)	0.02(0.26)	0.76(0.29)	0.74(0.27)	9.26E-01	9.77E-03	7.87E-03	9.98E-01	8.98E-02	9.17E-02
6802	0.07(0.22)	0.08(0.16)	-1.06(0.40)	-0.00(0.26)	-1.13(0.45)	-1.13(0.43)	9.92E-01	1.38E-02	1.02E-02	9.98E-01	1.16E-01	9.87E-02
132976	-0.16(0.20)	-0.16(0.22)	0.51(0.17)	-0.01(0.30)	0.67(0.26)	0.67(0.28)	9.80E-01	1.45E-02	1.86E-02	9.98E-01	1.19E-01	1.30E-01

21513	0.10(0.19)	0.10(0.19)	-0.87(0.35)	-0.00(0.25)	-0.97(0.39)	-0.97(0.40)	9.86E-01	1.50E-02	1.82E-02	9.98E-01	1.20E-01	1.30E-01
47	0.23(0.19)	0.15(0.20)	0.83(0.16)	-0.09(0.26)	0.59(0.24)	0.68(0.25)	7.43E-01	1.62E-02	8.79E-03	9.98E-01	1.27E-01	9.47E-02
21666	-0.03(0.21)	-0.12(0.19)	0.62(0.18)	-0.09(0.28)	0.65(0.28)	0.74(0.26)	7.53E-01	2.14E-02	5.73E-03	9.98E-01	1.52E-01	7.72E-02
2039	-0.04(0.18)	-0.09(0.21)	0.65(0.23)	-0.06(0.28)	0.69(0.30)	0.75(0.30)	8.32E-01	2.31E-02	1.45E-02	9.98E-01	1.61E-01	1.22E-01
20903	0.10(0.35)	0.01(0.13)	1.03(0.22)	-0.09(0.38)	0.94(0.41)	1.03(0.26)	8.12E-01	2.43E-02	1.84E-04	9.98E-01	1.67E-01	8.67E-03
2061	0.07(0.21)	0.10(0.19)	0.70(0.17)	0.03(0.28)	0.62(0.28)	0.60(0.26)	9.24E-01	2.67E-02	2.35E-02	9.98E-01	1.77E-01	1.49E-01
443	0.13(0.21)	-0.01(0.17)	0.76(0.19)	-0.13(0.27)	0.63(0.28)	0.77(0.25)	6.22E-01	2.75E-02	3.45E-03	9.98E-01	1.77E-01	5.00E-02
109423	-0.10(0.15)	-0.89(1.40)	0.48(0.23)	-0.80(1.40)	0.58(0.27)	1.37(1.40)	5.73E-01	3.53E-02	3.31E-01	9.98E-01	2.15E-01	7.48E-01
1981	0.11(0.13)	0.43(0.46)	0.69(0.23)	0.32(0.47)	0.57(0.27)	0.25(0.51)	5.04E-01	3.67E-02	6.22E-01	9.98E-01	2.19E-01	9.16E-01
1912	0.06(0.18)	-0.14(0.20)	0.64(0.22)	-0.20(0.28)	0.58(0.28)	0.78(0.30)	4.68E-01	4.02E-02	1.11E-02	9.98E-01	2.33E-01	1.02E-01
1941	0.01(0.20)	0.02(0.19)	0.60(0.21)	0.01(0.27)	0.60(0.29)	0.59(0.29)	9.66E-01	4.22E-02	4.53E-02	9.98E-01	2.41E-01	2.37E-01
1029	0.22(0.24)	0.08(0.17)	0.81(0.16)	-0.14(0.29)	0.59(0.29)	0.73(0.23)	6.29E-01	4.60E-02	2.28E-03	9.98E-01	2.59E-01	4.09E-02
109387	0.06(0.19)	0.08(0.25)	-0.48(0.20)	0.02(0.30)	-0.53(0.27)	-0.55(0.32)	9.51E-01	5.09E-02	8.54E-02	9.98E-01	2.78E-01	3.58E-01
5930	0.14(0.16)	0.24(0.24)	0.95(0.38)	0.09(0.29)	0.80(0.41)	0.71(0.46)	7.46E-01	5.72E-02	1.26E-01	9.98E-01	2.96E-01	4.71E-01
257	-0.10(0.23)	-0.04(0.18)	0.50(0.21)	0.06(0.28)	0.60(0.31)	0.54(0.27)	8.42E-01	5.79E-02	5.23E-02	9.98E-01	2.96E-01	2.50E-01
3465	0.06(0.17)	-0.15(0.24)	-0.51(0.24)	-0.21(0.29)	-0.57(0.30)	-0.36(0.34)	4.76E-01	6.07E-02	2.86E-01	9.98E-01	3.00E-01	7.20E-01
14509	0.11(0.17)	0.07(0.20)	0.83(0.37)	-0.04(0.26)	0.72(0.41)	0.76(0.42)	8.77E-01	8.19E-02	7.53E-02	9.98E-01	3.80E-01	3.26E-01
132414	0.08(0.23)	0.04(0.15)	-0.63(0.35)	-0.04(0.27)	-0.71(0.40)	-0.67(0.37)	8.72E-01	8.24E-02	7.68E-02	9.98E-01	3.80E-01	3.27E-01
1725	0.07(0.17)	0.00(0.24)	0.55(0.22)	-0.07(0.29)	0.47(0.27)	0.55(0.32)	7.99E-01	8.81E-02	8.86E-02	9.98E-01	3.95E-01	3.67E-01
4956	0.16(0.22)	0.07(0.17)	-0.49(0.31)	-0.10(0.28)	-0.66(0.38)	-0.56(0.35)	7.31E-01	9.30E-02	1.18E-01	9.98E-01	4.12E-01	4.59E-01
469	0.17(0.20)	-0.08(0.19)	0.64(0.19)	-0.25(0.27)	0.47(0.28)	0.72(0.26)	3.54E-01	9.61E-02	8.39E-03	9.98E-01	4.13E-01	9.31E-02
134336	0.16(0.17)	0.36(0.26)	0.60(0.21)	0.20(0.30)	0.43(0.26)	0.23(0.32)	5.05E-01	9.62E-02	4.68E-01	9.98E-01	4.13E-01	8.52E-01
117141	0.09(0.21)	0.00(0.21)	0.56(0.18)	-0.09(0.30)	0.47(0.28)	0.56(0.27)	7.50E-01	9.64E-02	4.17E-02	9.98E-01	4.13E-01	2.24E-01
103102	0.21(0.20)	0.29(0.21)	0.62(0.17)	0.09(0.28)	0.41(0.26)	0.32(0.26)	7.53E-01	1.14E-01	2.17E-01	9.98E-01	4.82E-01	6.33E-01
5346	0.15(0.17)	0.31(0.24)	0.55(0.19)	0.17(0.30)	0.41(0.26)	0.24(0.30)	5.74E-01	1.23E-01	4.34E-01	9.98E-01	4.98E-01	8.47E-01
4942	0.06(0.23)	0.02(0.16)	-0.54(0.33)	-0.04(0.27)	-0.60(0.39)	-0.56(0.36)	8.85E-01	1.29E-01	1.25E-01	9.98E-01	5.14E-01	4.71E-01
17068	0.12(0.19)	-0.03(0.20)	0.54(0.22)	-0.15(0.28)	0.42(0.28)	0.57(0.30)	5.93E-01	1.33E-01	5.74E-02	9.98E-01	5.23E-01	2.67E-01
160313	0.72(0.93)	0.15(0.20)	-0.69(0.25)	-0.57(0.94)	-1.41(0.97)	-0.84(0.32)	5.48E-01	1.48E-01	1.05E-02	9.98E-01	5.56E-01	9.87E-02
4948	-0.02(0.26)	0.03(0.15)	-0.59(0.30)	0.06(0.29)	-0.57(0.39)	-0.63(0.33)	8.50E-01	1.49E-01	6.09E-02	9.98E-01	5.56E-01	2.80E-01

6526	0.18(0.33)	0.02(0.14)	-0.48(0.35)	-0.16(0.35)	-0.66(0.46)	-0.51(0.38)	6.58E-01	1.54E-01	1.81E-01	9.98E-01	5.71E-01	6.02E-01
8607	-0.14(0.24)	0.06(0.28)	-0.53(0.14)	0.20(0.37)	-0.39(0.28)	-0.59(0.32)	5.97E-01	1.57E-01	7.15E-02	9.98E-01	5.73E-01	3.13E-01
7053	-0.01(0.20)	-0.15(0.25)	0.39(0.23)	-0.14(0.31)	0.40(0.30)	0.55(0.34)	6.43E-01	1.90E-01	1.09E-01	9.98E-01	6.65E-01	4.34E-01
4577	0.14(0.22)	0.07(0.19)	0.53(0.21)	-0.06(0.29)	0.40(0.30)	0.46(0.29)	8.28E-01	1.92E-01	1.15E-01	9.98E-01	6.65E-01	4.53E-01
4981	0.19(0.37)	0.03(0.14)	-0.47(0.37)	-0.17(0.39)	-0.67(0.51)	-0.50(0.40)	6.65E-01	1.96E-01	2.13E-01	9.98E-01	6.65E-01	6.33E-01
5483	0.04(0.19)	0.05(0.18)	-0.46(0.33)	0.01(0.26)	-0.49(0.38)	-0.51(0.37)	9.58E-01	1.96E-01	1.82E-01	9.98E-01	6.65E-01	6.02E-01
134	0.07(0.16)	-0.14(0.25)	-0.35(0.29)	-0.20(0.30)	-0.42(0.33)	-0.22(0.37)	4.94E-01	2.06E-01	5.65E-01	9.98E-01	6.78E-01	9.04E-01
4927	0.06(0.17)	0.03(0.24)	-2.61(2.10)	-0.03(0.30)	-2.67(2.10)	-2.65(2.10)	9.28E-01	2.07E-01	2.12E-01	9.98E-01	6.78E-01	6.33E-01
9320	0.03(0.19)	0.14(0.28)	0.36(0.18)	0.11(0.34)	0.33(0.26)	0.23(0.33)	7.58E-01	2.10E-01	4.98E-01	9.98E-01	6.78E-01	8.74E-01
8270	-0.13(0.19)	-0.25(0.30)	-0.45(0.18)	-0.13(0.35)	-0.32(0.26)	-0.20(0.35)	7.23E-01	2.21E-01	5.66E-01	9.98E-01	7.05E-01	9.04E-01
4863	-0.01(0.18)	0.10(0.30)	0.29(0.18)	0.11(0.35)	0.30(0.26)	0.19(0.36)	7.59E-01	2.48E-01	5.94E-01	9.98E-01	7.60E-01	9.07E-01
6580	0.02(0.20)	0.08(0.18)	-0.40(0.33)	0.06(0.26)	-0.42(0.37)	-0.48(0.37)	8.32E-01	2.63E-01	2.06E-01	9.98E-01	8.00E-01	6.33E-01
4941	0.07(0.22)	0.05(0.18)	-0.39(0.37)	-0.02(0.28)	-0.46(0.42)	-0.44(0.41)	9.40E-01	2.77E-01	2.85E-01	9.98E-01	8.00E-01	7.20E-01
3329	0.09(0.18)	0.07(0.20)	-0.46(0.48)	-0.02(0.26)	-0.54(0.50)	-0.52(0.52)	9.30E-01	2.78E-01	3.18E-01	9.98E-01	8.00E-01	7.41E-01
18488	0.11(0.24)	0.11(0.17)	-0.26(0.25)	0.01(0.29)	-0.37(0.34)	-0.37(0.30)	9.78E-01	2.84E-01	2.17E-01	9.98E-01	8.08E-01	6.33E-01
4925	0.05(0.22)	0.08(0.18)	-0.34(0.29)	0.04(0.27)	-0.39(0.36)	-0.42(0.34)	8.93E-01	2.85E-01	2.11E-01	9.98E-01	8.08E-01	6.33E-01
4971	0.06(0.21)	0.04(0.19)	-0.27(0.25)	-0.02(0.28)	-0.33(0.32)	-0.32(0.31)	9.53E-01	2.97E-01	3.20E-01	9.98E-01	8.33E-01	7.41E-01
41708	0.11(0.29)	0.08(0.16)	-0.30(0.28)	-0.03(0.32)	-0.41(0.39)	-0.38(0.32)	9.24E-01	2.99E-01	2.43E-01	9.98E-01	8.33E-01	6.68E-01
4937	0.15(0.19)	0.08(0.19)	0.56(0.37)	-0.07(0.27)	0.41(0.40)	0.48(0.41)	7.99E-01	3.04E-01	2.48E-01	9.98E-01	8.35E-01	6.72E-01
4938	0.07(0.25)	0.04(0.17)	-0.42(0.43)	-0.04(0.29)	-0.49(0.48)	-0.46(0.46)	9.05E-01	3.06E-01	3.22E-01	9.98E-01	8.36E-01	7.41E-01
54	0.06(0.20)	0.16(0.18)	-0.31(0.30)	0.10(0.27)	-0.37(0.36)	-0.47(0.35)	7.06E-01	3.09E-01	1.82E-01	9.98E-01	8.39E-01	6.02E-01
3258	0.02(0.16)	0.02(0.27)	0.40(0.35)	0.01(0.30)	0.38(0.38)	0.37(0.43)	9.80E-01	3.19E-01	3.86E-01	9.98E-01	8.52E-01	8.10E-01
573	0.03(0.17)	0.44(0.34)	0.30(0.22)	0.40(0.38)	0.27(0.27)	-0.14(0.40)	2.91E-01	3.27E-01	7.31E-01	9.98E-01	8.67E-01	9.29E-01
4943	0.07(0.22)	0.03(0.17)	-0.31(0.33)	-0.04(0.27)	-0.38(0.39)	-0.34(0.36)	8.95E-01	3.36E-01	3.49E-01	9.98E-01	8.75E-01	7.70E-01
47170	0.12(0.22)	0.16(0.20)	0.40(0.20)	0.05(0.29)	0.28(0.29)	0.23(0.28)	8.77E-01	3.47E-01	4.13E-01	9.98E-01	8.75E-01	8.28E-01
6579	0.10(0.16)	0.07(0.21)	-0.43(0.56)	-0.03(0.26)	-0.53(0.57)	-0.50(0.59)	9.13E-01	3.55E-01	3.96E-01	9.98E-01	8.75E-01	8.18E-01
17651	0.05(0.19)	0.21(0.23)	-0.22(0.25)	0.16(0.31)	-0.28(0.30)	-0.43(0.34)	6.13E-01	3.57E-01	2.12E-01	9.98E-01	8.75E-01	6.33E-01
100253	0.12(0.30)	0.04(0.16)	0.47(0.22)	-0.09(0.34)	0.34(0.37)	0.43(0.28)	8.02E-01	3.58E-01	1.25E-01	9.98E-01	8.75E-01	4.71E-01
64551	0.15(0.25)	0.16(0.19)	-0.15(0.20)	0.01(0.31)	-0.30(0.32)	-0.31(0.28)	9.69E-01	3.59E-01	2.75E-01	9.98E-01	8.75E-01	7.11E-01

153	0.07(0.20)	0.08(0.24)	0.33(0.20)	0.01(0.31)	0.26(0.28)	0.25(0.32)	9.77E-01	3.60E-01	4.33E-01	9.98E-01	8.75E-01	8.47E-01
892	-0.11(0.25)	0.07(0.15)	-0.53(0.38)	0.18(0.29)	-0.41(0.45)	-0.59(0.41)	5.36E-01	3.62E-01	1.53E-01	9.98E-01	8.75E-01	5.30E-01
87710	-0.09(0.17)	0.09(0.28)	-0.36(0.23)	0.18(0.34)	-0.27(0.29)	-0.45(0.37)	5.90E-01	3.63E-01	2.32E-01	9.98E-01	8.75E-01	6.54E-01
1875	0.18(0.20)	0.33(0.18)	0.47(0.27)	0.16(0.26)	0.29(0.32)	0.14(0.31)	5.49E-01	3.68E-01	6.61E-01	9.98E-01	8.79E-01	9.22E-01
4871	0.07(0.16)	-0.16(0.42)	-0.15(0.21)	-0.23(0.44)	-0.23(0.26)	0.01(0.46)	6.00E-01	3.85E-01	9.88E-01	9.98E-01	9.08E-01	9.97E-01
4887	0.04(0.24)	0.32(0.23)	0.30(0.20)	0.28(0.32)	0.26(0.31)	-0.02(0.30)	3.93E-01	4.02E-01	9.49E-01	9.98E-01	9.26E-01	9.94E-01
41697	0.14(0.23)	0.07(0.17)	-0.19(0.33)	-0.07(0.28)	-0.33(0.39)	-0.25(0.36)	7.97E-01	4.03E-01	4.87E-01	9.98E-01	9.26E-01	8.70E-01
91421	0.04(0.20)	0.23(0.24)	-0.23(0.25)	0.19(0.31)	-0.26(0.32)	-0.46(0.33)	5.36E-01	4.09E-01	1.76E-01	9.98E-01	9.31E-01	6.02E-01
1878	0.19(0.18)	0.30(0.21)	0.43(0.25)	0.12(0.27)	0.24(0.29)	0.12(0.32)	6.58E-01	4.15E-01	7.01E-01	9.98E-01	9.31E-01	9.29E-01
31357	0.08(0.16)	0.22(0.28)	-0.15(0.23)	0.14(0.32)	-0.22(0.28)	-0.37(0.37)	6.61E-01	4.20E-01	3.20E-01	9.98E-01	9.31E-01	7.41E-01
5288	0.11(0.23)	0.06(0.16)	0.41(0.32)	-0.05(0.27)	0.30(0.38)	0.34(0.35)	8.66E-01	4.30E-01	3.31E-01	9.98E-01	9.31E-01	7.48E-01
117171	0.04(0.19)	0.07(0.26)	0.26(0.21)	0.03(0.32)	0.22(0.28)	0.19(0.33)	9.22E-01	4.32E-01	5.77E-01	9.98E-01	9.31E-01	9.04E-01
4945	0.03(0.22)	0.05(0.18)	0.28(0.26)	0.01(0.28)	0.25(0.32)	0.24(0.31)	9.67E-01	4.42E-01	4.50E-01	9.98E-01	9.31E-01	8.50E-01
46292	0.26(0.25)	0.18(0.17)	0.52(0.26)	-0.08(0.30)	0.26(0.35)	0.34(0.31)	7.90E-01	4.62E-01	2.76E-01	9.98E-01	9.31E-01	7.11E-01
41682	0.07(0.19)	0.04(0.30)	-0.13(0.19)	-0.03(0.36)	-0.20(0.27)	-0.17(0.35)	9.36E-01	4.70E-01	6.36E-01	9.98E-01	9.31E-01	9.17E-01
139467	0.24(0.27)	0.05(0.16)	-0.03(0.30)	-0.19(0.30)	-0.26(0.38)	-0.08(0.33)	5.32E-01	4.84E-01	8.21E-01	9.98E-01	9.31E-01	9.46E-01
4922	0.22(0.60)	0.16(0.19)	-0.22(0.22)	-0.07(0.62)	-0.44(0.63)	-0.37(0.30)	9.17E-01	4.87E-01	2.12E-01	9.98E-01	9.31E-01	6.33E-01
119167	-0.05(0.28)	0.00(0.27)	-0.27(0.15)	0.05(0.40)	-0.22(0.32)	-0.27(0.31)	8.94E-01	4.96E-01	3.83E-01	9.98E-01	9.31E-01	8.10E-01
5482	0.17(0.26)	0.07(0.16)	-0.12(0.36)	-0.10(0.29)	-0.29(0.43)	-0.20(0.39)	7.39E-01	4.96E-01	6.19E-01	9.98E-01	9.31E-01	9.16E-01
160543	0.17(0.36)	0.14(0.24)	0.43(0.17)	-0.03(0.41)	0.26(0.39)	0.29(0.29)	9.41E-01	5.04E-01	3.12E-01	9.98E-01	9.31E-01	7.41E-01
6864	0.11(0.24)	0.07(0.17)	-0.15(0.34)	-0.04(0.28)	-0.26(0.39)	-0.22(0.37)	8.74E-01	5.05E-01	5.56E-01	9.98E-01	9.31E-01	9.04E-01
7403	-0.08(1.79)	0.11(0.18)	1.14(0.27)	0.19(1.80)	1.21(1.81)	1.03(0.32)	9.18E-01	5.06E-01	2.17E-03	9.98E-01	9.31E-01	4.09E-02
2936	0.19(0.25)	0.10(0.25)	0.39(0.16)	-0.09(0.35)	0.19(0.30)	0.29(0.30)	7.96E-01	5.20E-01	3.39E-01	9.98E-01	9.31E-01	7.58E-01
160	0.02(0.32)	-0.08(0.22)	0.26(0.21)	-0.10(0.38)	0.24(0.37)	0.34(0.30)	7.82E-01	5.23E-01	2.57E-01	9.98E-01	9.31E-01	6.82E-01
4932	0.12(0.23)	-0.18(0.20)	-0.08(0.20)	-0.30(0.30)	-0.20(0.31)	0.10(0.28)	3.28E-01	5.28E-01	7.14E-01	9.98E-01	9.31E-01	9.29E-01
473	0.15(0.23)	0.05(0.16)	-0.10(0.34)	-0.10(0.27)	-0.25(0.40)	-0.15(0.37)	7.11E-01	5.32E-01	6.93E-01	9.98E-01	9.31E-01	9.29E-01
160463	-0.07(0.23)	-0.05(0.19)	0.12(0.21)	0.03(0.29)	0.19(0.31)	0.17(0.28)	9.26E-01	5.34E-01	5.56E-01	9.98E-01	9.31E-01	9.04E-01
4928	0.35(1.28)	0.16(0.19)	-0.46(0.25)	-0.19(1.29)	-0.80(1.30)	-0.62(0.31)	8.86E-01	5.38E-01	5.21E-02	9.98E-01	9.31E-01	2.50E-01
1064	-0.02(0.16)	-0.11(0.29)	-0.40(0.59)	-0.10(0.33)	-0.38(0.61)	-0.28(0.67)	7.72E-01	5.40E-01	6.73E-01	9.98E-01	9.31E-01	9.27E-01

6331	0.07(0.14)	0.17(0.52)	-0.31(0.63)	0.10(0.53)	-0.39(0.64)	-0.48(0.80)	8.55E-01	5.48E-01	5.47E-01	9.98E-01	9.31E-01	9.04E-01
41691	0.20(0.24)	0.05(0.17)	0.40(0.26)	-0.14(0.29)	0.21(0.35)	0.35(0.30)	6.23E-01	5.48E-01	2.50E-01	9.98E-01	9.31E-01	6.72E-01
4976	-0.02(0.14)	0.07(0.33)	0.22(0.38)	0.08(0.35)	0.24(0.40)	0.15(0.49)	8.13E-01	5.51E-01	7.52E-01	9.98E-01	9.31E-01	9.30E-01
41808	0.12(0.18)	0.14(0.24)	-0.07(0.27)	0.02(0.30)	-0.19(0.32)	-0.21(0.37)	9.43E-01	5.57E-01	5.76E-01	9.98E-01	9.31E-01	9.04E-01
87769	0.11(0.19)	0.09(0.19)	-0.10(0.31)	-0.02(0.27)	-0.21(0.36)	-0.19(0.36)	9.34E-01	5.63E-01	6.03E-01	9.98E-01	9.31E-01	9.16E-01
4546	-0.34(0.40)	-0.06(0.24)	-0.59(0.16)	0.27(0.47)	-0.25(0.44)	-0.53(0.28)	5.65E-01	5.66E-01	6.91E-02	9.98E-01	9.31E-01	3.06E-01
490	0.10(0.21)	0.04(0.18)	-0.12(0.33)	-0.05(0.27)	-0.22(0.38)	-0.16(0.36)	8.38E-01	5.69E-01	6.59E-01	9.98E-01	9.31E-01	9.22E-01
2806	-1.26(2.06)	0.30(0.22)	-0.08(0.20)	1.56(2.08)	1.18(2.07)	-0.38(0.30)	4.57E-01	5.70E-01	2.18E-01	9.98E-01	9.31E-01	6.33E-01
106629	0.13(0.19)	0.18(0.20)	0.33(0.30)	0.04(0.27)	0.20(0.35)	0.16(0.36)	8.78E-01	5.72E-01	6.63E-01	9.98E-01	9.31E-01	9.22E-01
4926	-0.05(0.79)	0.03(0.20)	-0.49(0.18)	0.09(0.82)	-0.44(0.81)	-0.52(0.27)	9.16E-01	5.92E-01	5.52E-02	9.98E-01	9.46E-01	2.60E-01
4975	0.01(0.18)	0.30(0.24)	0.16(0.22)	0.29(0.29)	0.14(0.28)	-0.15(0.32)	3.28E-01	6.07E-01	6.52E-01	9.98E-01	9.56E-01	9.22E-01
119129	2.39(4.43)	0.02(0.21)	0.11(0.15)	-2.37(4.44)	-2.28(4.43)	0.09(0.25)	5.96E-01	6.08E-01	7.34E-01	9.98E-01	9.56E-01	9.29E-01
1148	0.28(0.36)	-0.09(0.19)	0.47(0.16)	-0.37(0.40)	0.19(0.39)	0.56(0.25)	3.62E-01	6.25E-01	2.83E-02	9.98E-01	9.66E-01	1.62E-01
41699	0.11(0.26)	0.06(0.17)	0.28(0.26)	-0.05(0.30)	0.17(0.36)	0.23(0.32)	8.63E-01	6.30E-01	4.76E-01	9.98E-01	9.67E-01	8.59E-01
126397	0.08(0.18)	0.06(0.20)	-0.10(0.34)	-0.02(0.26)	-0.18(0.37)	-0.16(0.39)	9.37E-01	6.32E-01	6.83E-01	9.98E-01	9.67E-01	9.29E-01
91	0.09(0.26)	0.04(0.20)	-0.07(0.19)	-0.05(0.33)	-0.16(0.33)	-0.10(0.27)	8.73E-01	6.34E-01	7.04E-01	9.98E-01	9.67E-01	9.29E-01
4600	0.12(0.27)	0.14(0.32)	0.26(0.15)	0.02(0.43)	0.14(0.30)	0.12(0.36)	9.62E-01	6.41E-01	7.38E-01	9.98E-01	9.73E-01	9.29E-01
110	0.29(0.34)	0.17(0.20)	0.47(0.22)	-0.12(0.39)	0.18(0.40)	0.30(0.29)	7.67E-01	6.43E-01	3.06E-01	9.98E-01	9.73E-01	7.41E-01
98027	0.03(0.19)	-0.31(0.31)	0.28(0.51)	-0.34(0.37)	0.25(0.55)	0.60(0.60)	3.62E-01	6.45E-01	3.22E-01	9.98E-01	9.73E-01	7.41E-01
120562	0.10(0.22)	0.07(0.19)	-0.05(0.28)	-0.04(0.28)	-0.16(0.34)	-0.12(0.33)	8.92E-01	6.52E-01	7.22E-01	9.98E-01	9.74E-01	9.29E-01
42357	0.03(0.15)	-0.08(0.29)	0.21(0.38)	-0.10(0.32)	0.18(0.41)	0.29(0.48)	7.49E-01	6.55E-01	5.56E-01	9.98E-01	9.74E-01	9.04E-01
4986	0.10(0.30)	0.16(0.22)	0.25(0.19)	0.06(0.37)	0.15(0.36)	0.10(0.29)	8.75E-01	6.65E-01	7.38E-01	9.98E-01	9.74E-01	9.29E-01
479	0.11(0.23)	0.07(0.19)	-0.04(0.28)	-0.04(0.28)	-0.15(0.34)	-0.11(0.33)	8.95E-01	6.65E-01	7.33E-01	9.98E-01	9.74E-01	9.29E-01
111162	2.33(5.44)	0.09(0.22)	-0.01(0.19)	-2.24(5.43)	-2.34(5.44)	-0.10(0.29)	6.81E-01	6.68E-01	7.36E-01	9.98E-01	9.74E-01	9.29E-01
145494	0.12(0.23)	0.05(0.16)	-0.06(0.36)	-0.07(0.28)	-0.18(0.42)	-0.11(0.39)	8.14E-01	6.78E-01	7.77E-01	9.98E-01	9.74E-01	9.32E-01
4898	0.04(0.19)	0.18(0.26)	0.15(0.20)	0.14(0.31)	0.11(0.27)	-0.03(0.33)	6.60E-01	6.80E-01	9.36E-01	9.98E-01	9.74E-01	9.94E-01
3286	0.12(0.20)	0.06(0.18)	-0.07(0.43)	-0.05(0.26)	-0.19(0.47)	-0.14(0.46)	8.41E-01	6.84E-01	7.65E-01	9.98E-01	9.74E-01	9.32E-01
592	0.09(0.28)	0.11(0.20)	-0.05(0.20)	0.02(0.34)	-0.14(0.34)	-0.16(0.29)	9.60E-01	6.86E-01	5.84E-01	9.98E-01	9.74E-01	9.04E-01
3232	0.10(0.19)	-0.05(0.22)	0.21(0.21)	-0.15(0.29)	0.11(0.28)	0.26(0.30)	6.01E-01	6.90E-01	3.87E-01	9.98E-01	9.74E-01	8.10E-01

119066	0.21(0.19)	-0.05(0.27)	0.10(0.20)	-0.26(0.34)	-0.11(0.28)	0.15(0.34)	4.47E-01	6.94E-01	6.64E-01	9.98E-01	9.74E-01	9.22E-01
4983	0.13(0.25)	0.05(0.16)	-0.02(0.31)	-0.09(0.29)	-0.16(0.40)	-0.07(0.36)	7.69E-01	6.95E-01	8.42E-01	9.98E-01	9.74E-01	9.46E-01
6353	0.19(0.32)	-0.04(0.18)	0.04(0.24)	-0.23(0.35)	-0.15(0.39)	0.08(0.29)	5.12E-01	6.96E-01	7.85E-01	9.98E-01	9.74E-01	9.32E-01
9392	0.06(0.21)	-0.12(0.24)	0.18(0.25)	-0.18(0.31)	0.12(0.33)	0.30(0.36)	5.63E-01	7.17E-01	4.03E-01	9.98E-01	9.74E-01	8.20E-01
448	0.06(0.19)	0.05(0.19)	-0.08(0.35)	-0.02(0.26)	-0.14(0.40)	-0.13(0.39)	9.47E-01	7.18E-01	7.51E-01	9.98E-01	9.74E-01	9.30E-01
4933	0.02(0.15)	-0.17(0.34)	-0.10(0.29)	-0.18(0.38)	-0.12(0.33)	0.07(0.44)	6.27E-01	7.20E-01	8.82E-01	9.98E-01	9.74E-01	9.66E-01
7402	-0.08(1.05)	0.10(0.22)	0.31(0.25)	0.18(1.07)	0.38(1.08)	0.21(0.33)	8.70E-01	7.23E-01	5.33E-01	9.98E-01	9.74E-01	8.92E-01
915	-0.83(3.11)	0.21(0.22)	0.27(0.22)	1.04(3.11)	1.09(3.11)	0.05(0.31)	7.39E-01	7.26E-01	8.70E-01	9.98E-01	9.74E-01	9.56E-01
17453	0.05(0.17)	0.08(0.25)	0.15(0.22)	0.03(0.30)	0.10(0.29)	0.07(0.33)	9.13E-01	7.26E-01	8.39E-01	9.98E-01	9.74E-01	9.46E-01
1675	0.07(0.15)	-0.27(1.00)	-0.02(0.21)	-0.34(1.01)	-0.09(0.26)	0.25(1.02)	7.39E-01	7.34E-01	8.08E-01	9.98E-01	9.74E-01	9.43E-01
17263	0.07(0.19)	0.08(0.21)	-0.06(0.36)	0.01(0.28)	-0.13(0.40)	-0.14(0.41)	9.71E-01	7.44E-01	7.35E-01	9.98E-01	9.80E-01	9.29E-01
1872	0.14(0.19)	0.29(0.25)	0.05(0.21)	0.15(0.30)	-0.09(0.27)	-0.23(0.32)	6.33E-01	7.49E-01	4.64E-01	9.98E-01	9.82E-01	8.50E-01
6538	0.12(0.20)	-0.05(0.23)	0.21(0.23)	-0.17(0.30)	0.10(0.30)	0.27(0.32)	5.65E-01	7.50E-01	4.04E-01	9.98E-01	9.82E-01	8.20E-01
130396	0.09(0.24)	0.10(0.17)	-0.01(0.25)	-0.00(0.29)	-0.10(0.33)	-0.11(0.30)	9.92E-01	7.61E-01	7.24E-01	9.98E-01	9.89E-01	9.29E-01
5900	0.13(0.21)	0.10(0.17)	0.23(0.30)	-0.03(0.27)	0.10(0.36)	0.13(0.35)	9.06E-01	7.85E-01	7.08E-01	9.98E-01	9.94E-01	9.29E-01
120526	0.04(0.21)	0.06(0.21)	-0.05(0.28)	0.02(0.28)	-0.09(0.33)	-0.11(0.34)	9.35E-01	7.89E-01	7.39E-01	9.98E-01	9.94E-01	9.29E-01
5085	-0.38(0.41)	0.12(0.27)	-0.26(0.17)	0.50(0.50)	0.12(0.44)	-0.38(0.32)	3.22E-01	7.91E-01	2.41E-01	9.98E-01	9.94E-01	6.67E-01
119023	-0.03(0.40)	0.03(0.20)	0.08(0.16)	0.06(0.45)	0.11(0.43)	0.05(0.26)	9.01E-01	8.01E-01	8.41E-01	9.98E-01	9.94E-01	9.46E-01
87720	0.40(0.23)	0.14(0.18)	0.32(0.21)	-0.26(0.29)	-0.08(0.31)	0.18(0.27)	3.77E-01	8.07E-01	5.13E-01	9.98E-01	9.94E-01	8.87E-01
26916	0.05(0.12)	0.37(1.55)	-3.94(16.31)	0.32(1.56)	-3.99(16.30)	-4.31(16.35)	8.38E-01	8.07E-01	7.93E-01	9.98E-01	9.94E-01	9.37E-01
4929	0.25(0.36)	0.26(0.19)	0.16(0.20)	0.01(0.40)	-0.09(0.40)	-0.10(0.27)	9.72E-01	8.25E-01	7.06E-01	9.98E-01	9.94E-01	9.29E-01
137	0.02(0.22)	0.24(0.22)	-0.04(0.20)	0.22(0.31)	-0.07(0.30)	-0.28(0.30)	4.91E-01	8.26E-01	3.53E-01	9.98E-01	9.94E-01	7.70E-01
1996	0.24(0.24)	0.10(0.19)	0.30(0.21)	-0.14(0.30)	0.07(0.31)	0.21(0.28)	6.39E-01	8.30E-01	4.64E-01	9.98E-01	9.94E-01	8.50E-01
32247	-0.04(0.24)	0.07(0.20)	0.03(0.20)	0.11(0.31)	0.07(0.32)	-0.04(0.28)	7.36E-01	8.36E-01	8.86E-01	9.98E-01	9.94E-01	9.66E-01
61	0.07(0.25)	0.21(0.35)	0.13(0.15)	0.13(0.43)	0.06(0.29)	-0.08(0.38)	7.56E-01	8.41E-01	8.45E-01	9.98E-01	9.94E-01	9.46E-01
26918	0.05(0.12)	0.32(1.57)	2.24(11.04)	0.27(1.57)	2.18(11.04)	1.91(11.11)	8.63E-01	8.44E-01	8.64E-01	9.98E-01	9.94E-01	9.52E-01
1704	0.19(0.25)	0.12(0.18)	0.12(0.22)	-0.07(0.30)	-0.06(0.33)	0.00(0.28)	8.27E-01	8.50E-01	9.89E-01	9.98E-01	9.94E-01	9.97E-01
160542	0.71(1.51)	0.20(0.32)	0.42(0.13)	-0.51(1.51)	-0.29(1.51)	0.22(0.35)	7.38E-01	8.51E-01	5.20E-01	9.98E-01	9.94E-01	8.92E-01

4609	0.23(0.86)	0.19(0.23)	0.39(0.23)	-0.04(0.89)	0.16(0.89)	0.20(0.33)	9.64E-01	8.55E-01	5.36E-01	9.98E-01	9.94E-01	8.94E-01
4543	0.14(0.39)	0.12(0.20)	0.06(0.21)	-0.02(0.44)	-0.08(0.44)	-0.06(0.29)	9.68E-01	8.58E-01	8.35E-01	9.98E-01	9.94E-01	9.46E-01
106936	0.15(0.23)	0.04(0.19)	0.09(0.24)	-0.11(0.28)	-0.06(0.33)	0.05(0.30)	7.05E-01	8.65E-01	8.64E-01	9.98E-01	9.94E-01	9.52E-01
139436	0.29(0.59)	0.33(0.31)	0.38(0.15)	0.04(0.66)	0.09(0.61)	0.06(0.33)	9.57E-01	8.77E-01	8.62E-01	9.98E-01	9.94E-01	9.52E-01
135889	-0.20(0.26)	-0.25(0.26)	-0.95(4.92)	-0.05(0.36)	-0.76(4.95)	-0.70(4.93)	8.83E-01	8.79E-01	8.87E-01	9.98E-01	9.94E-01	9.66E-01
160464	0.80(2.86)	0.19(0.23)	0.37(0.19)	-0.61(2.87)	-0.44(2.86)	0.18(0.30)	8.32E-01	8.79E-01	5.54E-01	9.98E-01	9.94E-01	9.04E-01
34075	0.22(0.21)	-0.25(0.20)	0.18(0.21)	-0.48(0.29)	-0.04(0.30)	0.44(0.29)	1.07E-01	8.88E-01	1.37E-01	9.98E-01	9.94E-01	4.95E-01
31559	-0.01(0.32)	0.12(0.89)	-0.06(0.13)	0.13(0.93)	-0.05(0.35)	-0.18(0.89)	8.91E-01	8.89E-01	8.44E-01	9.98E-01	9.94E-01	9.46E-01
121002	0.09(0.21)	0.11(0.22)	0.13(0.23)	0.02(0.30)	0.04(0.29)	0.02(0.32)	9.43E-01	8.91E-01	9.54E-01	9.98E-01	9.94E-01	9.95E-01
39	0.11(0.19)	0.16(0.23)	0.15(0.27)	0.05(0.30)	0.04(0.31)	0.00(0.35)	8.78E-01	8.93E-01	9.93E-01	9.98E-01	9.94E-01	9.98E-01
2011	0.04(0.21)	0.19(0.22)	0.00(0.21)	0.15(0.30)	-0.04(0.29)	-0.19(0.31)	6.15E-01	8.98E-01	5.46E-01	9.98E-01	9.94E-01	9.04E-01
145492	0.29(0.30)	0.01(0.20)	0.34(0.19)	-0.28(0.36)	0.04(0.35)	0.33(0.27)	4.29E-01	8.99E-01	2.32E-01	9.98E-01	9.94E-01	6.54E-01
106634	0.08(0.17)	0.06(0.21)	0.02(0.44)	-0.02(0.26)	-0.06(0.47)	-0.04(0.48)	9.43E-01	8.99E-01	9.34E-01	9.98E-01	9.94E-01	9.94E-01
17253	0.56(0.66)	0.14(0.23)	0.48(0.18)	-0.42(0.70)	-0.08(0.69)	0.34(0.30)	5.51E-01	9.07E-01	2.57E-01	9.98E-01	9.94E-01	6.82E-01
453	-0.02(0.19)	0.15(0.21)	0.02(0.30)	0.17(0.28)	0.04(0.35)	-0.13(0.36)	5.54E-01	9.08E-01	7.28E-01	9.98E-01	9.94E-01	9.29E-01
135619	-0.04(0.28)	-0.10(0.26)	-0.08(0.17)	-0.06(0.40)	-0.04(0.34)	0.02(0.30)	8.86E-01	9.13E-01	9.47E-01	9.98E-01	9.94E-01	9.94E-01
657	0.07(0.24)	0.03(0.17)	0.04(0.24)	-0.05(0.29)	-0.03(0.34)	0.02(0.29)	8.71E-01	9.24E-01	9.58E-01	9.98E-01	9.94E-01	9.95E-01
122151	0.02(0.21)	0.08(0.21)	-0.01(0.28)	0.05(0.28)	-0.03(0.34)	-0.09(0.34)	8.46E-01	9.26E-01	7.99E-01	9.98E-01	9.94E-01	9.41E-01
4936	0.08(0.19)	0.06(0.21)	0.19(1.29)	-0.02(0.28)	0.11(1.30)	0.13(1.29)	9.45E-01	9.32E-01	9.20E-01	9.98E-01	9.94E-01	9.85E-01
7440	1.00(2.30)	0.12(0.17)	0.81(0.22)	-0.88(2.31)	-0.20(2.31)	0.69(0.28)	7.04E-01	9.33E-01	1.61E-02	9.98E-01	9.94E-01	1.24E-01
93385	0.12(0.28)	0.10(0.20)	0.09(0.20)	-0.01(0.34)	-0.03(0.34)	-0.01(0.28)	9.70E-01	9.38E-01	9.61E-01	9.98E-01	9.94E-01	9.95E-01
3029	0.06(0.12)	0.28(0.67)	-0.05(1.33)	0.22(0.68)	-0.10(1.34)	-0.33(1.47)	7.42E-01	9.39E-01	8.25E-01	9.98E-01	9.94E-01	9.46E-01
4985	0.21(0.43)	0.20(0.21)	0.18(0.24)	-0.01(0.47)	-0.03(0.49)	-0.02(0.32)	9.80E-01	9.45E-01	9.45E-01	9.98E-01	9.94E-01	9.94E-01
17664	0.07(0.23)	0.07(0.18)	0.04(0.24)	-0.00(0.29)	-0.02(0.33)	-0.02(0.29)	9.98E-01	9.47E-01	9.42E-01	9.98E-01	9.94E-01	9.94E-01
307	0.08(0.21)	0.16(0.19)	0.10(0.25)	0.08(0.28)	0.02(0.32)	-0.06(0.32)	7.76E-01	9.53E-01	8.50E-01	9.98E-01	9.94E-01	9.48E-01
93947	0.08(0.32)	0.03(0.16)	0.10(0.25)	-0.05(0.35)	0.02(0.40)	0.07(0.29)	8.88E-01	9.64E-01	8.15E-01	9.98E-01	9.94E-01	9.44E-01
139478	-0.13(2.18)	0.12(0.21)	-0.03(0.29)	0.25(2.18)	0.10(2.20)	-0.15(0.35)	9.09E-01	9.64E-01	6.67E-01	9.98E-01	9.94E-01	9.22E-01
4533	0.15(0.33)	0.01(0.28)	0.16(0.16)	-0.14(0.43)	0.01(0.36)	0.15(0.32)	7.53E-01	9.68E-01	6.38E-01	9.98E-01	9.94E-01	9.17E-01
16788	-0.01(0.37)	0.09(0.24)	0.00(0.15)	0.10(0.43)	0.02(0.40)	-0.08(0.28)	8.20E-01	9.70E-01	7.66E-01	9.98E-01	9.94E-01	9.32E-01

13107	0.10(0.21)	0.11(0.18)	0.12(0.32)	-0.00(0.27)	0.01(0.38)	0.01(0.37)	9.94E-01	9.70E-01	9.74E-01	9.98E-01	9.94E-01	9.95E-01
121890	0.05(0.12)	0.05(0.81)	0.08(0.78)	-0.00(0.81)	0.03(0.79)	0.03(1.14)	9.96E-01	9.71E-01	9.77E-01	9.98E-01	9.94E-01	9.95E-01
2900	0.20(0.21)	-0.09(0.21)	0.18(0.27)	-0.28(0.31)	-0.01(0.34)	0.27(0.34)	3.67E-01	9.71E-01	4.33E-01	9.98E-01	9.94E-01	8.47E-01
133943	0.29(0.42)	0.11(0.21)	0.27(0.19)	-0.18(0.46)	-0.02(0.46)	0.17(0.29)	6.97E-01	9.73E-01	5.67E-01	9.98E-01	9.94E-01	9.04E-01
17537	0.08(0.22)	0.11(0.19)	0.09(0.27)	0.02(0.28)	0.01(0.34)	-0.01(0.32)	9.31E-01	9.74E-01	9.67E-01	9.98E-01	9.94E-01	9.95E-01
16561	0.08(0.20)	0.08(0.18)	0.09(0.38)	-0.00(0.26)	0.01(0.42)	0.01(0.42)	9.95E-01	9.74E-01	9.78E-01	9.98E-01	9.94E-01	9.95E-01
5419	0.19(0.52)	0.12(0.19)	0.20(0.18)	-0.07(0.54)	0.02(0.54)	0.08(0.26)	9.02E-01	9.76E-01	7.55E-01	9.98E-01	9.94E-01	9.30E-01
68	0.09(0.20)	0.17(0.25)	0.09(0.19)	0.08(0.31)	-0.01(0.27)	-0.09(0.32)	8.03E-01	9.77E-01	7.86E-01	9.98E-01	9.94E-01	9.32E-01
107960	0.22(0.24)	0.04(0.17)	0.23(0.31)	-0.18(0.28)	0.01(0.39)	0.19(0.35)	5.21E-01	9.82E-01	5.85E-01	9.98E-01	9.94E-01	9.04E-01
1700	0.06(0.39)	0.06(0.14)	0.06(0.72)	-0.01(0.41)	-0.01(0.81)	0.00(0.73)	9.86E-01	9.94E-01	9.98E-01	9.98E-01	9.94E-01	9.98E-01

Regression coefficient β* (standard error (SE)) denotes the association between baseline level of each metabolite and change in FPG in each intervention group (IMI, BAND or RYGB). β† (SE) denotes the association between baseline level of each metabolite and change in FPG induced by different types of weight-loss interventions (BAND vs. IMI, RYGB vs. IMI, and RYGB vs. BAND), and was obtained from the interaction term (metabolite × types of intervention) in a linear regression model. In this model, % change in FPG (follow-up value minus baseline value, divided by baseline value) was the dependent variable, baseline level of each metabolite, intervention groups (1=IMI, 2=BAND, 3=RYGB, with IMI being the reference), and an interaction term (metabolite × types of intervention) were the independent variables, adjusting for baseline age, sex, BMI as well as weight loss. Abbreviations: IMI: Intensive medical intervention. BAND: Laparoscopic adjustable banding. RYGB: Roux-en-Y gastric bypass surgery.

Table S2. A full list of baseline plasma metabolites associated with change in HbA1c before and 1-yr after intervention

	β^{*}(SE)			$\beta^{\dagger}$ (SE)			p-value			q-value		
**Metabolites**	**IMI**	**BAND**	**RYGB**	**BAND *vs.* IMI**	**RYGB *vs.* IMI**	**RYGB *vs.* BAND**	**BAND *vs.* IMI**	**RYGB vs. IMI**	**RYGB *vs.* BAND**	**BAND *vs.* IMI**	**RYGB *vs.* IMI**	**RYGB *vs.* BAND**
**Known**												
1,5-anhydroglucitol	-0.14(0.15)	0.17(0.13)	0.83(0.14)	0.31(0.20)	0.97(0.21)	0.66(0.19)	1.19E-01	1.44E-05	9.77E-04	9.98E-01	5.44E-03	4.60E-02
1-monopalmitin	0.06(0.17)	0.08(0.13)	0.37(0.18)	0.02(0.21)	0.31(0.24)	0.28(0.22)	9.18E-01	2.14E-01	1.97E-01	9.98E-01	4.79E-01	4.66E-01
1-monostearin	-0.09(0.11)	-0.07(0.20)	0.66(0.23)	0.01(0.23)	0.74(0.26)	0.73(0.30)	9.63E-01	5.28E-03	1.86E-02	9.98E-01	8.78E-02	1.75E-01
2,3-dihydroxybutanoic acid NIST	-0.04(0.13)	0.08(0.20)	-0.02(0.17)	0.11(0.24)	0.02(0.21)	-0.09(0.26)	6.33E-01	9.24E-01	7.27E-01	9.98E-01	9.73E-01	8.62E-01
2,4-diaminobutyric acid	0.00(0.16)	-0.07(0.16)	0.41(0.14)	-0.07(0.22)	0.42(0.21)	0.49(0.21)	7.46E-01	4.68E-02	2.38E-02	9.98E-01	2.13E-01	1.99E-01
2-deoxyisotetronic acid NIST	-0.06(0.15)	0.19(0.19)	-0.08(0.15)	0.24(0.24)	-0.03(0.22)	-0.27(0.24)	3.26E-01	9.05E-01	2.66E-01	9.98E-01	9.63E-01	5.22E-01
2-deoxytetronic acid	0.00(0.17)	0.12(0.19)	0.07(0.16)	0.12(0.24)	0.07(0.23)	-0.05(0.24)	6.13E-01	7.45E-01	8.43E-01	9.98E-01	9.21E-01	9.34E-01
2-hydroxybutanoic acid	0.07(0.18)	-0.11(0.15)	-0.53(0.12)	-0.18(0.23)	-0.60(0.22)	-0.42(0.19)	4.41E-01	6.75E-03	3.28E-02	9.98E-01	9.44E-02	2.34E-01
2-hydroxyglutaric acid	-0.01(0.15)	0.19(0.16)	0.46(0.13)	0.20(0.22)	0.47(0.20)	0.27(0.20)	3.66E-01	2.00E-02	1.73E-01	9.98E-01	1.57E-01	4.42E-01
2-hydroxypyrazinyl-2-propenoic acid ethyl ester NIST	0.08(0.12)	-0.10(0.16)	0.62(0.16)	-0.18(0.20)	0.54(0.20)	0.72(0.23)	3.76E-01	7.57E-03	2.13E-03	9.98E-01	9.52E-02	6.68E-02
2-hydroxyvaleric acid	0.02(0.28)	-0.11(0.16)	-0.09(0.16)	-0.14(0.32)	-0.11(0.32)	0.03(0.22)	6.74E-01	7.40E-01	9.02E-01	9.98E-01	9.21E-01	9.66E-01
2-piperidinobenzonitrile NIST	-0.05(0.16)	0.12(0.14)	0.42(0.23)	0.17(0.20)	0.47(0.27)	0.30(0.26)	4.04E-01	8.56E-02	2.57E-01	9.98E-01	2.99E-01	5.17E-01
3-(1-pyrazolyl)-L-alanine	-0.08(0.30)	-0.10(0.55)	-0.05(0.10)	-0.01(0.62)	0.04(0.32)	0.05(0.56)	9.82E-01	9.10E-01	9.29E-01	9.98E-01	9.66E-01	9.79E-01
3,6-anhydro-D-galactose	0.07(0.29)	0.08(0.22)	-0.04(0.12)	0.01(0.37)	-0.11(0.32)	-0.12(0.24)	9.80E-01	7.37E-01	6.32E-01	9.98E-01	9.21E-01	8.02E-01
3-aminoisobutyric acid	0.02(0.14)	0.08(0.12)	1.05(0.37)	0.07(0.18)	1.04(0.40)	0.97(0.39)	7.10E-01	1.16E-02	1.59E-02	9.98E-01	1.11E-01	1.65E-01
3-hydroxybutyric acid	0.11(0.20)	-0.08(0.15)	-0.21(0.14)	-0.19(0.26)	-0.31(0.25)	-0.13(0.21)	4.65E-01	2.05E-01	5.53E-01	9.98E-01	4.71E-01	7.64E-01
4-hydroxyphenylacetic acid	-0.05(0.21)	0.12(0.22)	-0.02(0.12)	0.17(0.30)	0.03(0.24)	-0.13(0.25)	5.85E-01	8.95E-01	5.97E-01	9.98E-01	9.63E-01	8.02E-01
Acetaminophen	-0.23(0.35)	0.14(0.14)	0.35(0.12)	0.36(0.37)	0.57(0.37)	0.21(0.18)	3.33E-01	1.24E-01	2.47E-01	9.98E-01	3.59E-01	5.11E-01

Aconitic acid	0.03(0.21)	0.09(0.19)	-0.02(0.12)	0.06(0.28)	-0.05(0.24)	-0.11(0.23)	8.23E-01	8.51E-01	6.35E-01	9.98E-01	9.48E-01	8.02E-01
Adenosine-5-monophosphate	-0.03(0.13)	0.08(0.14)	0.62(0.20)	0.11(0.19)	0.64(0.24)	0.54(0.24)	5.85E-01	7.98E-03	2.98E-02	9.98E-01	9.70E-02	2.29E-01
Alanine	0.08(0.17)	0.08(0.17)	0.17(0.14)	-0.01(0.24)	0.09(0.22)	0.10(0.23)	9.82E-01	6.84E-01	6.76E-01	9.98E-01	8.82E-01	8.21E-01
Allantoic acid	-0.13(0.15)	-0.05(0.20)	0.64(0.38)	0.08(0.25)	0.77(0.41)	0.69(0.43)	7.66E-01	6.55E-02	1.12E-01	9.98E-01	2.77E-01	3.60E-01
Allantoin	-0.63(1.63)	-0.05(0.13)	0.77(0.15)	0.58(1.64)	1.41(1.64)	0.83(0.20)	7.24E-01	3.94E-01	9.99E-05	9.98E-01	6.84E-01	1.26E-02
Alloxanoic acid NIST	0.01(0.20)	0.10(0.11)	0.69(0.24)	0.09(0.22)	0.68(0.30)	0.59(0.26)	6.84E-01	2.75E-02	2.83E-02	9.98E-01	1.76E-01	2.22E-01
Aminomalonate	0.07(0.13)	0.26(0.18)	0.42(0.15)	0.18(0.21)	0.35(0.20)	0.16(0.23)	3.98E-01	8.54E-02	4.79E-01	9.98E-01	2.99E-01	7.14E-01
Arabitol	-0.05(0.22)	0.11(0.24)	0.03(0.11)	0.16(0.33)	0.08(0.25)	-0.08(0.27)	6.20E-01	7.43E-01	7.61E-01	9.98E-01	9.21E-01	8.88E-01
Arachidic acid	-0.04(0.16)	0.10(0.18)	0.32(0.14)	0.14(0.25)	0.36(0.22)	0.22(0.23)	5.81E-01	1.01E-01	3.28E-01	9.98E-01	3.25E-01	5.80E-01
Arachidonic acid	0.14(0.16)	-0.09(0.17)	0.29(0.13)	-0.23(0.23)	0.15(0.21)	0.38(0.21)	3.16E-01	4.75E-01	7.68E-02	9.98E-01	7.26E-01	3.08E-01
Asparagine	0.13(0.20)	-0.01(0.15)	0.08(0.14)	-0.15(0.24)	-0.06(0.24)	0.09(0.20)	5.42E-01	8.08E-01	6.57E-01	9.98E-01	9.44E-01	8.12E-01
Aspartic acid	0.05(0.18)	0.19(0.14)	0.14(0.18)	0.13(0.23)	0.09(0.25)	-0.05(0.22)	5.54E-01	7.37E-01	8.27E-01	9.98E-01	9.21E-01	9.33E-01
Behenic acid	-0.03(0.10)	-0.47(0.18)	0.71(0.31)	-0.44(0.21)	0.74(0.32)	1.18(0.36)	4.35E-02	2.36E-02	1.77E-03	9.98E-01	1.63E-01	6.08E-02
Benzoic acid	-0.06(0.39)	0.04(0.14)	0.57(0.21)	0.10(0.41)	0.64(0.43)	0.54(0.25)	8.07E-01	1.46E-01	3.53E-02	9.98E-01	3.87E-01	2.34E-01
Beta-alanine	-0.03(0.14)	0.11(0.15)	0.23(0.19)	0.14(0.21)	0.26(0.23)	0.12(0.24)	5.12E-01	2.71E-01	6.21E-01	9.98E-01	5.63E-01	8.02E-01
Butane-2,3-diol NIST	-0.03(0.14)	-0.23(0.15)	-0.49(1.09)	-0.20(0.20)	-0.46(1.09)	-0.26(1.10)	3.37E-01	6.77E-01	8.14E-01	9.98E-01	8.80E-01	9.27E-01
Capric acid	0.00(0.29)	0.02(0.16)	0.49(0.12)	0.02(0.33)	0.49(0.31)	0.47(0.20)	9.51E-01	1.17E-01	1.95E-02	9.98E-01	3.42E-01	1.80E-01
Caprylic acid	0.06(0.20)	0.12(0.17)	0.39(0.12)	0.06(0.26)	0.33(0.23)	0.26(0.21)	8.09E-01	1.55E-01	2.11E-01	9.98E-01	3.97E-01	4.73E-01
Cellobiose minor	-0.03(0.13)	0.15(0.18)	0.32(0.17)	0.18(0.22)	0.35(0.21)	0.17(0.24)	4.03E-01	1.05E-01	4.96E-01	9.98E-01	3.27E-01	7.19E-01
Cholesterol	-0.05(0.26)	0.08(0.12)	0.30(0.23)	0.13(0.29)	0.34(0.34)	0.22(0.25)	6.64E-01	3.22E-01	4.01E-01	9.98E-01	6.04E-01	6.57E-01
Citric acid	0.09(0.13)	0.10(0.14)	0.79(0.21)	0.01(0.18)	0.70(0.24)	0.68(0.25)	9.38E-01	4.41E-03	7.16E-03	9.98E-01	7.92E-02	1.23E-01
Conduritol-beta-expoxide	0.07(0.16)	0.01(0.13)	0.52(0.16)	-0.06(0.21)	0.45(0.22)	0.51(0.20)	7.66E-01	4.49E-02	1.41E-02	9.98E-01	2.12E-01	1.53E-01
Creatinine	0.02(0.14)	-0.15(0.15)	-0.12(0.26)	-0.16(0.20)	-0.13(0.28)	0.03(0.29)	4.10E-01	6.40E-01	9.22E-01	9.98E-01	8.46E-01	9.79E-01
Cysteine	-0.08(0.17)	-0.11(0.25)	0.05(0.12)	-0.03(0.30)	0.13(0.21)	0.17(0.28)	9.08E-01	5.30E-01	5.47E-01	9.98E-01	7.66E-01	7.63E-01
Cysteine-glycine	0.03(0.23)	0.08(0.17)	-0.16(0.12)	0.05(0.29)	-0.19(0.26)	-0.23(0.21)	8.71E-01	4.78E-01	2.66E-01	9.98E-01	7.26E-01	5.22E-01
Cystine	-0.09(0.17)	-0.08(0.15)	-0.48(0.17)	0.01(0.22)	-0.39(0.23)	-0.40(0.23)	9.80E-01	9.81E-02	8.60E-02	9.98E-01	3.24E-01	3.21E-01
Dehydroabietic acid	-0.11(0.17)	0.22(0.33)	0.22(0.19)	0.33(0.37)	0.32(0.25)	0.00(0.38)	3.80E-01	2.10E-01	9.90E-01	9.98E-01	4.77E-01	9.94E-01
Deoxycholic acid	0.05(0.13)	0.01(0.22)	0.02(0.17)	-0.04(0.25)	-0.04(0.21)	0.01(0.27)	8.62E-01	8.60E-01	9.83E-01	9.98E-01	9.48E-01	9.94E-01

Dodecanol	0.01(0.13)	0.04(0.16)	0.49(0.17)	0.04(0.20)	0.49(0.21)	0.45(0.23)	8.53E-01	2.66E-02	5.61E-02	9.98E-01	1.76E-01	2.77E-01
Erythritol	-0.03(0.23)	0.21(0.21)	-0.11(0.12)	0.24(0.31)	-0.08(0.25)	-0.32(0.24)	4.44E-01	7.44E-01	1.83E-01	9.98E-01	9.21E-01	4.53E-01
Ethanolamine	0.08(0.16)	0.33(0.19)	0.22(0.13)	0.25(0.24)	0.14(0.21)	-0.11(0.23)	3.06E-01	5.07E-01	6.30E-01	9.98E-01	7.49E-01	8.02E-01
Fructose	0.17(0.31)	-0.19(0.27)	-0.13(0.13)	-0.35(0.40)	-0.29(0.34)	0.06(0.31)	3.76E-01	3.84E-01	8.47E-01	9.98E-01	6.83E-01	9.34E-01
Fucose	0.00(0.20)	0.07(0.18)	0.01(0.13)	0.07(0.27)	0.02(0.24)	-0.05(0.22)	7.93E-01	9.41E-01	8.09E-01	9.98E-01	9.74E-01	9.27E-01
Fumaric acid	0.07(0.13)	0.05(0.14)	0.75(0.18)	-0.02(0.19)	0.68(0.22)	0.69(0.23)	9.24E-01	2.74E-03	3.27E-03	9.98E-01	7.92E-02	8.75E-02
Galactonic acid	0.04(0.22)	-0.03(0.17)	-0.34(0.13)	-0.08(0.28)	-0.38(0.26)	-0.31(0.21)	7.86E-01	1.41E-01	1.56E-01	9.98E-01	3.78E-01	4.30E-01
Galactose	-0.05(0.16)	-0.14(0.17)	-0.23(0.14)	-0.09(0.23)	-0.18(0.21)	-0.10(0.22)	7.13E-01	3.93E-01	6.55E-01	9.98E-01	6.84E-01	8.12E-01
Gluconic acid	-0.03(0.24)	0.10(0.17)	-0.37(0.13)	0.12(0.30)	-0.35(0.28)	-0.47(0.22)	6.80E-01	2.13E-01	3.38E-02	9.98E-01	4.79E-01	2.34E-01
Glucose	0.09(0.15)	-0.20(0.26)	-0.24(0.13)	-0.29(0.30)	-0.33(0.20)	-0.04(0.29)	3.43E-01	1.01E-01	8.96E-01	9.98E-01	3.25E-01	9.63E-01
Glutamic acid	-0.09(0.14)	-0.20(0.17)	-0.47(0.14)	-0.11(0.22)	-0.39(0.19)	-0.28(0.21)	6.16E-01	4.63E-02	2.01E-01	9.98E-01	2.13E-01	4.66E-01
Glutamine	0.04(0.14)	-0.07(0.16)	0.29(0.20)	-0.12(0.20)	0.25(0.25)	0.36(0.26)	5.74E-01	3.22E-01	1.74E-01	9.98E-01	6.04E-01	4.42E-01
Glyceric acid	0.04(0.13)	0.10(0.17)	0.34(0.18)	0.06(0.21)	0.30(0.22)	0.24(0.25)	7.85E-01	1.81E-01	3.30E-01	9.98E-01	4.29E-01	5.81E-01
Glycerol	0.12(0.15)	0.09(0.15)	0.37(0.19)	-0.04(0.20)	0.24(0.23)	0.28(0.23)	8.49E-01	2.88E-01	2.21E-01	9.98E-01	5.80E-01	4.81E-01
Glycerol-alpha-phosphate	-0.10(0.15)	0.08(0.15)	-0.11(0.17)	0.18(0.22)	-0.01(0.23)	-0.18(0.23)	4.23E-01	9.81E-01	4.34E-01	9.98E-01	9.92E-01	6.76E-01
Glycine	0.08(0.14)	0.00(0.20)	0.23(0.14)	-0.08(0.25)	0.15(0.21)	0.23(0.24)	7.60E-01	4.74E-01	3.51E-01	9.98E-01	7.26E-01	6.03E-01
Glycolic acid	0.06(0.12)	0.18(0.22)	-0.24(0.22)	0.12(0.25)	-0.30(0.25)	-0.42(0.32)	6.43E-01	2.44E-01	2.01E-01	9.98E-01	5.24E-01	4.66E-01
Heptadecanoic acid	-0.01(0.15)	-0.06(0.18)	0.27(0.14)	-0.06(0.24)	0.28(0.21)	0.34(0.24)	8.14E-01	1.80E-01	1.60E-01	9.98E-01	4.29E-01	4.30E-01
Hexitol	-0.01(0.16)	-0.08(0.12)	0.26(0.24)	-0.07(0.20)	0.27(0.28)	0.34(0.27)	7.34E-01	3.39E-01	2.08E-01	9.98E-01	6.32E-01	4.70E-01
Hexuronic acid	-0.03(0.33)	0.02(0.24)	0.03(0.12)	0.05(0.41)	0.06(0.34)	0.01(0.27)	9.00E-01	8.57E-01	9.68E-01	9.98E-01	9.48E-01	9.94E-01
Hippuric acid	-0.16(0.31)	0.07(0.10)	0.13(0.28)	0.23(0.33)	0.29(0.41)	0.06(0.30)	4.89E-01	4.87E-01	8.50E-01	9.98E-01	7.31E-01	9.34E-01
Histidine	0.03(0.14)	-0.12(0.19)	-0.11(0.19)	-0.15(0.23)	-0.14(0.24)	0.01(0.27)	5.19E-01	5.63E-01	9.80E-01	9.98E-01	7.98E-01	9.94E-01
Hydrocinnamic acid	0.04(0.23)	-0.17(0.12)	0.62(0.37)	-0.22(0.26)	0.58(0.43)	0.80(0.38)	3.99E-01	1.81E-01	4.23E-02	9.98E-01	4.29E-01	2.41E-01
Hydroxylamine	0.02(0.14)	0.10(0.13)	0.83(0.19)	0.08(0.18)	0.81(0.23)	0.73(0.22)	6.58E-01	6.37E-04	1.70E-03	9.98E-01	3.43E-02	6.08E-02
Hydroxyproline dipeptide NIST	-0.12(0.13)	0.02(0.19)	-0.01(0.20)	0.14(0.23)	0.11(0.23)	-0.03(0.27)	5.34E-01	6.30E-01	9.14E-01	9.98E-01	8.45E-01	9.76E-01
Hypoxanthine	-0.10(0.19)	-0.06(0.14)	0.29(0.16)	0.05(0.24)	0.39(0.25)	0.34(0.22)	8.47E-01	1.27E-01	1.22E-01	9.98E-01	3.65E-01	3.78E-01
Indole-3-acetate	-0.13(0.16)	0.07(0.14)	0.04(0.42)	0.20(0.22)	0.18(0.45)	-0.02(0.44)	3.59E-01	6.95E-01	9.57E-01	9.98E-01	8.88E-01	9.93E-01
Indole-3-lactate	-0.12(0.17)	0.33(0.21)	0.05(0.12)	0.45(0.27)	0.17(0.21)	-0.28(0.24)	9.48E-02	4.15E-01	2.45E-01	9.98E-01	6.96E-01	5.11E-01

Isocitric acid	0.02(0.13)	0.10(0.15)	0.35(0.26)	0.09(0.20)	0.33(0.28)	0.24(0.29)	6.62E-01	2.45E-01	4.06E-01	9.98E-01	5.24E-01	6.59E-01
Isoleucine	-0.09(0.20)	0.19(0.15)	-0.09(0.17)	0.28(0.25)	0.00(0.26)	-0.28(0.22)	2.58E-01	9.92E-01	2.06E-01	9.98E-01	9.92E-01	4.69E-01
Isothreitol	-0.12(0.25)	0.18(0.22)	0.05(0.11)	0.30(0.33)	0.17(0.27)	-0.13(0.25)	3.68E-01	5.17E-01	6.05E-01	9.98E-01	7.55E-01	8.02E-01
Isothreonic acid	0.23(0.28)	0.21(0.21)	-0.08(0.11)	-0.02(0.35)	-0.31(0.31)	-0.30(0.24)	9.65E-01	3.11E-01	2.20E-01	9.98E-01	5.99E-01	4.81E-01
Ketohexose	0.00(0.09)	0.34(0.65)	-0.73(0.72)	0.34(0.66)	-0.73(0.73)	-1.07(0.97)	6.05E-01	3.20E-01	2.73E-01	9.98E-01	6.04E-01	5.22E-01
Kynurenine	-0.06(0.11)	0.14(0.28)	-0.02(0.23)	0.20(0.30)	0.04(0.25)	-0.16(0.37)	5.06E-01	8.66E-01	6.67E-01	9.98E-01	9.49E-01	8.17E-01
Lactic acid	0.02(0.21)	-0.03(0.24)	-0.29(0.11)	-0.05(0.33)	-0.31(0.24)	-0.26(0.26)	8.81E-01	2.03E-01	3.20E-01	9.98E-01	4.69E-01	5.75E-01
Lactulose	-0.07(0.36)	0.00(0.24)	-0.18(0.11)	0.08(0.43)	-0.10(0.38)	-0.18(0.27)	8.61E-01	7.85E-01	5.03E-01	9.98E-01	9.33E-01	7.26E-01
Lauric acid	0.03(0.19)	0.03(0.14)	0.36(0.20)	-0.00(0.24)	0.33(0.28)	0.34(0.24)	9.94E-01	2.32E-01	1.70E-01	9.99E-01	5.11E-01	4.42E-01
Leucine	-0.04(0.19)	0.17(0.15)	0.07(0.17)	0.21(0.23)	0.11(0.25)	-0.10(0.22)	3.64E-01	6.45E-01	6.55E-01	9.98E-01	8.47E-01	8.12E-01
Levoglucosan	-0.04(0.27)	0.02(0.15)	0.16(0.12)	0.07(0.31)	0.20(0.29)	0.14(0.20)	8.26E-01	4.90E-01	4.89E-01	9.98E-01	7.32E-01	7.17E-01
Linoleic acid	0.12(0.18)	-0.04(0.15)	0.10(0.17)	-0.16(0.23)	-0.02(0.24)	0.14(0.23)	4.85E-01	9.41E-01	5.38E-01	9.98E-01	9.74E-01	7.57E-01
Lysine	-0.07(0.15)	0.04(0.18)	-0.22(0.19)	0.11(0.24)	-0.15(0.24)	-0.26(0.26)	6.56E-01	5.26E-01	3.21E-01	9.98E-01	7.66E-01	5.75E-01
Lyxitol	0.01(0.16)	-0.12(0.19)	0.05(0.15)	-0.13(0.24)	0.04(0.21)	0.17(0.24)	5.95E-01	8.44E-01	4.73E-01	9.98E-01	9.48E-01	7.13E-01
Malic acid	0.08(0.37)	0.05(0.49)	0.08(0.10)	-0.03(0.61)	0.01(0.38)	0.04(0.50)	9.59E-01	9.87E-01	9.39E-01	9.98E-01	9.92E-01	9.84E-01
Maltose	-0.08(0.13)	0.10(0.19)	0.30(0.17)	0.18(0.22)	0.38(0.21)	0.20(0.25)	4.17E-01	7.67E-02	4.34E-01	9.98E-01	2.92E-01	6.76E-01
Maltotriose	-0.02(0.18)	0.17(0.13)	0.50(0.14)	0.19(0.21)	0.52(0.22)	0.33(0.19)	3.69E-01	2.06E-02	8.24E-02	9.98E-01	1.58E-01	3.20E-01
Mannitol	-0.09(0.27)	-1.13(0.42)	-0.03(0.10)	-1.04(0.49)	0.05(0.29)	1.09(0.43)	3.80E-02	8.55E-01	1.37E-02	9.98E-01	9.48E-01	1.53E-01
Mannose	-0.01(0.18)	-0.02(0.19)	-0.31(0.14)	-0.01(0.25)	-0.30(0.22)	-0.28(0.23)	9.57E-01	1.87E-01	2.27E-01	9.98E-01	4.37E-01	4.89E-01
Methanolphosphate	-0.03(0.13)	0.00(0.17)	-0.23(0.21)	0.03(0.21)	-0.20(0.24)	-0.23(0.27)	9.00E-01	4.11E-01	4.08E-01	9.98E-01	6.91E-01	6.61E-01
Methionine	-0.05(0.17)	-0.01(0.19)	0.19(0.38)	0.04(0.25)	0.24(0.42)	0.20(0.42)	8.88E-01	5.70E-01	6.36E-01	9.98E-01	7.99E-01	8.02E-01
Methionine sulfoxide	0.12(0.20)	0.02(0.15)	0.19(0.17)	-0.10(0.22)	0.06(0.26)	0.16(0.23)	6.53E-01	8.11E-01	4.72E-01	9.98E-01	9.44E-01	7.13E-01
Myo-inositol	0.05(0.21)	0.03(0.21)	-0.01(0.12)	-0.01(0.30)	-0.06(0.24)	-0.05(0.25)	9.72E-01	8.15E-01	8.49E-01	9.98E-01	9.44E-01	9.34E-01
Myristic acid	0.00(0.19)	0.02(0.13)	0.41(0.17)	0.02(0.23)	0.41(0.25)	0.38(0.21)	9.25E-01	1.10E-01	7.84E-02	9.98E-01	3.36E-01	3.08E-01
N-acetylglutamate	0.01(0.17)	0.09(0.14)	0.21(0.19)	0.08(0.21)	0.20(0.26)	0.12(0.24)	6.95E-01	4.40E-01	6.27E-01	9.98E-01	7.15E-01	8.02E-01
N-acetylglycine NIST	-0.01(0.15)	-0.04(0.15)	0.32(0.16)	-0.03(0.21)	0.33(0.22)	0.36(0.22)	8.99E-01	1.39E-01	1.10E-01	9.98E-01	3.78E-01	3.59E-01
N-acetylmannosamine	-0.10(0.22)	-0.06(0.12)	-0.18(0.22)	0.04(0.25)	-0.09(0.30)	-0.13(0.25)	8.75E-01	7.79E-01	6.13E-01	9.98E-01	9.33E-01	8.02E-01
N-acetylornithine	-0.03(0.25)	0.05(0.14)	0.03(0.18)	0.08(0.28)	0.06(0.30)	-0.02(0.22)	7.78E-01	8.47E-01	9.25E-01	9.98E-01	9.48E-01	9.79E-01
Naproxen	0.07(0.13)	0.56(1.11)	0.04(0.12)	0.49(1.11)	-0.03(0.18)	-0.52(1.12)	6.60E-01	8.76E-01	6.43E-01	9.98E-01	9.55E-01	8.05E-01

Nicotinic acid	0.02(0.12)	0.13(0.14)	0.93(0.23)	0.11(0.18)	0.90(0.26)	0.80(0.27)	5.50E-01	7.76E-04	4.07E-03	9.98E-01	3.66E-02	9.59E-02
N-methylalanine	0.06(0.14)	-0.15(0.16)	0.06(0.20)	-0.21(0.21)	0.00(0.24)	0.21(0.26)	3.38E-01	9.87E-01	4.17E-01	9.98E-01	9.92E-01	6.72E-01
Nonadecanoic acid	0.09(0.11)	-0.05(0.34)	0.22(0.20)	-0.14(0.35)	0.13(0.23)	0.27(0.39)	6.87E-01	5.70E-01	4.91E-01	9.98E-01	7.99E-01	7.17E-01
Oleic acid	0.10(0.21)	-0.06(0.14)	0.09(0.16)	-0.17(0.25)	-0.01(0.26)	0.16(0.21)	5.03E-01	9.67E-01	4.60E-01	9.98E-01	9.83E-01	7.06E-01
Ornithine	-0.05(0.17)	0.01(0.16)	0.11(0.15)	0.06(0.23)	0.17(0.22)	0.10(0.21)	7.91E-01	4.65E-01	6.30E-01	9.98E-01	7.24E-01	8.02E-01
Oxalic acid	0.03(0.15)	0.10(0.13)	0.64(0.25)	0.07(0.19)	0.61(0.28)	0.54(0.28)	7.09E-01	3.29E-02	5.79E-02	9.98E-01	1.94E-01	2.80E-01
Oxamic acid	0.00(0.17)	0.07(0.12)	0.45(0.22)	0.07(0.21)	0.45(0.27)	0.39(0.25)	7.48E-01	9.80E-02	1.25E-01	9.98E-01	3.24E-01	3.78E-01
Oxoproline	0.02(0.16)	0.20(0.15)	0.46(0.13)	0.18(0.21)	0.45(0.20)	0.26(0.20)	3.89E-01	3.12E-02	1.95E-01	9.98E-01	1.87E-01	4.66E-01
Palmitic acid	-0.01(0.16)	-0.03(0.17)	0.17(0.15)	-0.01(0.24)	0.18(0.22)	0.20(0.23)	9.56E-01	4.04E-01	4.05E-01	9.98E-01	6.89E-01	6.59E-01
Palmitoleic acid	0.05(0.28)	-0.06(0.13)	0.16(0.17)	-0.11(0.31)	0.11(0.33)	0.22(0.21)	7.24E-01	7.36E-01	3.05E-01	9.98E-01	9.21E-01	5.64E-01
Parabanic acid NIST	0.05(0.17)	0.06(0.12)	0.59(0.20)	0.01(0.20)	0.54(0.25)	0.53(0.23)	9.55E-01	3.57E-02	2.24E-02	9.98E-01	2.00E-01	1.92E-01
Pelargonic acid	-0.03(0.14)	-0.04(0.16)	0.58(0.16)	-0.01(0.21)	0.61(0.21)	0.62(0.22)	9.79E-01	4.37E-03	6.35E-03	9.98E-01	7.92E-02	1.22E-01
Pentadecanoic acid	-0.12(0.17)	-0.22(0.19)	-0.17(0.13)	-0.09(0.26)	-0.05(0.22)	0.04(0.23)	7.16E-01	8.16E-01	8.45E-01	9.98E-01	9.44E-01	9.34E-01
Phenylalanine	-0.09(0.16)	0.15(0.17)	0.27(0.15)	0.24(0.23)	0.36(0.22)	0.12(0.22)	3.05E-01	1.05E-01	5.80E-01	9.98E-01	3.27E-01	7.88E-01
Phenylethylamine	0.03(0.18)	0.05(0.16)	0.26(0.13)	0.02(0.24)	0.23(0.23)	0.21(0.21)	9.33E-01	3.04E-01	3.05E-01	9.98E-01	5.95E-01	5.64E-01
Phosphate	0.08(0.13)	0.04(0.15)	0.45(0.19)	-0.03(0.20)	0.38(0.23)	0.41(0.24)	8.69E-01	1.01E-01	8.68E-02	9.98E-01	3.25E-01	3.21E-01
Phosphoethanolamine	-0.02(0.13)	0.09(0.15)	0.67(0.17)	0.11(0.19)	0.69(0.20)	0.58(0.22)	5.51E-01	1.16E-03	1.04E-02	9.98E-01	4.84E-02	1.37E-01
Pinitol	0.02(0.22)	0.06(0.35)	0.98(0.43)	0.04(0.41)	0.96(0.48)	0.92(0.56)	9.28E-01	5.17E-02	1.05E-01	9.98E-01	2.26E-01	3.56E-01
Pipecolinic acid	-0.11(0.21)	0.01(0.18)	-0.01(0.12)	0.11(0.28)	0.10(0.24)	-0.01(0.22)	6.88E-01	6.85E-01	9.48E-01	9.98E-01	8.82E-01	9.90E-01
Proline	0.02(0.18)	-0.02(0.15)	0.19(0.17)	-0.04(0.23)	0.18(0.24)	0.21(0.22)	8.72E-01	4.74E-01	3.50E-01	9.98E-01	7.26E-01	6.03E-01
Pseudo uridine	-0.02(0.18)	0.15(0.17)	0.18(0.13)	0.16(0.25)	0.20(0.23)	0.03(0.22)	5.20E-01	3.90E-01	8.79E-01	9.98E-01	6.84E-01	9.50E-01
Pyrophosphate	-0.02(2.76)	-0.01(0.08)	9.58(2.31)	-0.00(2.77)	9.59(3.65)	9.59(2.31)	9.99E-01	1.08E-02	1.00E-04	9.99E-01	1.10E-01	1.26E-02
Pyrrole-2-carboxylic acid	0.00(0.14)	0.15(0.14)	0.63(0.21)	0.15(0.19)	0.63(0.25)	0.48(0.25)	4.45E-01	1.36E-02	5.65E-02	9.98E-01	1.22E-01	2.77E-01
Pyruvic acid	0.16(0.17)	0.13(0.16)	-0.20(0.14)	-0.03(0.23)	-0.36(0.22)	-0.33(0.22)	8.95E-01	1.15E-01	1.39E-01	9.98E-01	3.42E-01	4.04E-01
Quinic acid	-0.07(0.16)	0.02(0.13)	0.62(0.18)	0.08(0.21)	0.69(0.24)	0.61(0.23)	6.98E-01	5.36E-03	9.19E-03	9.98E-01	8.78E-02	1.37E-01
Ribonic acid	-0.13(0.38)	0.01(0.16)	0.17(0.16)	0.15(0.41)	0.30(0.41)	0.16(0.22)	7.20E-01	4.61E-01	4.82E-01	9.98E-01	7.24E-01	7.15E-01
Salicylic acid	-0.05(0.45)	0.02(0.10)	-0.45(1.41)	0.07(0.46)	-0.40(1.47)	-0.47(1.41)	8.86E-01	7.87E-01	7.43E-01	9.98E-01	9.33E-01	8.75E-01
Serine	0.12(0.14)	0.16(0.17)	0.30(0.16)	0.04(0.23)	0.18(0.21)	0.14(0.23)	8.48E-01	3.83E-01	5.50E-01	9.98E-01	6.83E-01	7.63E-01

Shikimic acid	0.05(0.14)	-0.26(0.12)	0.89(0.41)	-0.31(0.18)	0.84(0.45)	1.15(0.43)	8.84E-02	6.61E-02	1.05E-02	9.98E-01	2.77E-01	1.37E-01
Sorbitol	0.11(0.31)	0.71(3.29)	0.01(0.10)	0.60(3.30)	-0.10(0.33)	-0.70(3.29)	8.57E-01	7.55E-01	8.32E-01	9.98E-01	9.24E-01	9.34E-01
Stearic acid	-0.07(0.15)	-0.01(0.19)	0.24(0.15)	0.06(0.24)	0.30(0.21)	0.25(0.24)	8.16E-01	1.59E-01	3.09E-01	9.98E-01	4.02E-01	5.69E-01
Succinic acid	0.02(0.13)	0.29(0.21)	0.03(0.16)	0.27(0.25)	0.01(0.20)	-0.26(0.26)	2.77E-01	9.65E-01	3.20E-01	9.98E-01	9.83E-01	5.75E-01
Sucrose	-0.48(2.06)	0.10(0.21)	-0.01(0.10)	0.58(2.08)	0.47(2.06)	-0.11(0.23)	7.80E-01	8.21E-01	6.30E-01	9.98E-01	9.44E-01	8.02E-01
Tagatose	-0.11(0.31)	-0.09(0.31)	0.05(0.12)	0.02(0.44)	0.16(0.33)	0.14(0.33)	9.57E-01	6.27E-01	6.79E-01	9.98E-01	8.45E-01	8.21E-01
Taurine	0.01(0.21)	0.03(0.15)	-0.02(0.15)	0.02(0.25)	-0.03(0.25)	-0.05(0.21)	9.24E-01	9.21E-01	8.14E-01	9.98E-01	9.72E-01	9.27E-01
Threonic acid	0.04(0.23)	0.00(0.13)	0.19(0.18)	-0.04(0.26)	0.15(0.28)	0.19(0.22)	8.69E-01	6.05E-01	3.95E-01	9.98E-01	8.27E-01	6.55E-01
Threonine	0.07(0.15)	0.10(0.16)	0.35(0.17)	0.03(0.21)	0.28(0.23)	0.25(0.23)	9.04E-01	2.29E-01	2.87E-01	9.98E-01	5.11E-01	5.44E-01
Tocopherol alpha-	-0.08(0.16)	0.05(0.15)	-0.37(0.25)	0.13(0.21)	-0.29(0.28)	-0.42(0.29)	5.60E-01	3.04E-01	1.49E-01	9.98E-01	5.95E-01	4.26E-01
Tocopherol gamma-	0.04(0.17)	0.09(0.15)	0.29(0.20)	0.05(0.22)	0.25(0.25)	0.20(0.25)	8.14E-01	3.22E-01	4.21E-01	9.98E-01	6.04E-01	6.75E-01
Trans-4-hydroxyproline	-0.05(0.19)	-0.10(0.18)	0.08(0.13)	-0.05(0.27)	0.13(0.23)	0.18(0.23)	8.41E-01	5.91E-01	4.32E-01	9.98E-01	8.10E-01	6.76E-01
Tryptophan	-0.09(0.16)	-0.06(0.16)	-0.45(0.19)	0.03(0.22)	-0.36(0.24)	-0.39(0.25)	9.05E-01	1.39E-01	1.20E-01	9.98E-01	3.78E-01	3.78E-01
Tyrosine	-0.04(0.18)	0.08(0.19)	0.09(0.13)	0.12(0.26)	0.13(0.22)	0.01(0.23)	6.41E-01	5.74E-01	9.79E-01	9.98E-01	8.01E-01	9.94E-01
UDP GlcNAc	0.01(0.12)	-0.15(0.17)	0.53(0.19)	-0.16(0.21)	0.52(0.23)	0.68(0.26)	4.40E-01	2.37E-02	1.09E-02	9.98E-01	1.63E-01	1.37E-01
UDP-glucuronic acid	-0.12(0.50)	0.14(0.27)	0.29(0.09)	0.26(0.58)	0.40(0.51)	0.15(0.29)	6.56E-01	4.34E-01	6.11E-01	9.98E-01	7.11E-01	8.02E-01
Urea	0.01(0.20)	0.03(0.12)	0.22(0.18)	0.02(0.24)	0.21(0.27)	0.18(0.21)	9.20E-01	4.41E-01	3.96E-01	9.98E-01	7.15E-01	6.55E-01
Uric acid	-0.07(0.18)	0.16(0.20)	0.16(0.15)	0.24(0.26)	0.24(0.23)	0.00(0.25)	3.68E-01	3.05E-01	9.94E-01	9.98E-01	5.95E-01	9.94E-01
Uridine	-0.01(0.12)	0.26(0.22)	-0.14(0.25)	0.26(0.25)	-0.13(0.28)	-0.39(0.34)	2.95E-01	6.39E-01	2.45E-01	9.98E-01	8.46E-01	5.11E-01
Valine	-0.07(0.17)	0.18(0.15)	-0.03(0.18)	0.26(0.23)	0.04(0.25)	-0.22(0.23)	2.68E-01	8.71E-01	3.55E-01	9.98E-01	9.52E-01	6.03E-01
Xylitol	-0.03(0.15)	0.06(0.13)	0.59(0.22)	0.09(0.19)	0.62(0.26)	0.53(0.24)	6.43E-01	2.10E-02	3.42E-02	9.98E-01	1.58E-01	2.34E-01
Xylose	0.04(0.40)	0.27(0.20)	-0.10(0.16)	0.23(0.45)	-0.14(0.44)	-0.37(0.26)	6.13E-01	7.49E-01	1.62E-01	9.98E-01	9.23E-01	4.30E-01
**Unknown**												
87756	0.03(0.10)	0.10(0.15)	1.12(0.22)	0.06(0.18)	1.09(0.25)	1.02(0.27)	7.31E-01	3.99E-05	3.26E-04	9.98E-01	7.52E-03	1.83E-02
106634	0.04(0.11)	0.08(0.14)	1.30(0.30)	0.04(0.18)	1.26(0.31)	1.22(0.32)	8.24E-01	1.34E-04	3.40E-04	9.98E-01	1.27E-02	1.83E-02
109387	0.02(0.12)	0.09(0.17)	-0.70(0.13)	0.07(0.20)	-0.72(0.18)	-0.79(0.21)	7.14E-01	1.41E-04	3.36E-04	9.98E-01	1.27E-02	1.83E-02
135869	0.12(0.13)	-0.40(0.29)	-0.61(0.12)	-0.52(0.32)	-0.73(0.18)	-0.21(0.32)	1.10E-01	1.69E-04	5.18E-01	9.98E-01	1.27E-02	7.40E-01
14509	0.03(0.12)	0.10(0.14)	1.07(0.26)	0.07(0.18)	1.04(0.29)	0.97(0.30)	7.14E-01	5.82E-04	1.70E-03	9.98E-01	3.43E-02	6.08E-02

17651	-0.05(0.13)	-0.09(0.16)	0.61(0.18)	-0.04(0.22)	0.66(0.21)	0.69(0.24)	8.73E-01	2.46E-03	5.71E-03	9.98E-01	7.92E-02	1.20E-01
4976	0.03(0.10)	0.19(0.23)	0.90(0.26)	0.16(0.25)	0.87(0.28)	0.71(0.34)	5.10E-01	2.55E-03	4.10E-02	9.98E-01	7.92E-02	2.41E-01
5900	0.02(0.15)	0.14(0.12)	0.79(0.21)	0.12(0.18)	0.77(0.25)	0.65(0.24)	5.14E-01	2.65E-03	7.81E-03	9.98E-01	7.92E-02	1.28E-01
448	-0.01(0.13)	0.13(0.13)	0.83(0.25)	0.14(0.18)	0.84(0.28)	0.70(0.28)	4.41E-01	3.46E-03	1.39E-02	9.98E-01	7.92E-02	1.53E-01
134336	0.03(0.12)	0.21(0.19)	0.60(0.15)	0.17(0.22)	0.57(0.19)	0.39(0.24)	4.25E-01	3.69E-03	1.00E-01	9.98E-01	7.92E-02	3.50E-01
16550	0.03(0.12)	0.15(0.15)	0.91(0.27)	0.12(0.19)	0.88(0.29)	0.75(0.31)	5.21E-01	3.81E-03	1.62E-02	9.98E-01	7.92E-02	1.65E-01
17537	-0.05(0.15)	0.19(0.13)	0.65(0.18)	0.24(0.19)	0.70(0.23)	0.46(0.22)	2.14E-01	4.10E-03	4.26E-02	9.98E-01	7.92E-02	2.41E-01
145494	-0.06(0.16)	0.15(0.11)	0.81(0.25)	0.22(0.19)	0.88(0.30)	0.66(0.27)	2.70E-01	4.16E-03	1.80E-02	9.98E-01	7.92E-02	1.75E-01
64546	0.09(0.14)	0.03(0.26)	-0.46(0.12)	-0.07(0.30)	-0.56(0.19)	-0.49(0.28)	8.25E-01	4.17E-03	8.73E-02	9.98E-01	7.92E-02	3.21E-01
6646	0.14(0.15)	-0.48(0.34)	-0.40(0.12)	-0.62(0.37)	-0.55(0.19)	0.08(0.37)	9.85E-02	6.51E-03	8.36E-01	9.98E-01	9.44E-02	9.34E-01
13107	0.03(0.15)	0.15(0.13)	0.77(0.23)	0.13(0.19)	0.74(0.26)	0.61(0.26)	5.07E-01	6.52E-03	2.02E-02	9.98E-01	9.44E-02	1.81E-01
41689	0.00(0.12)	-0.02(0.29)	-0.59(0.18)	-0.03(0.31)	-0.59(0.21)	-0.56(0.34)	9.33E-01	6.98E-03	9.94E-02	9.98E-01	9.44E-02	3.50E-01
2039	-0.11(0.14)	-0.17(0.16)	0.51(0.17)	-0.06(0.21)	0.62(0.22)	0.68(0.22)	7.77E-01	7.01E-03	3.48E-03	9.98E-01	9.44E-02	8.75E-02
135610	0.25(0.16)	-0.11(0.23)	-0.31(0.13)	-0.36(0.28)	-0.56(0.20)	-0.20(0.26)	2.04E-01	7.28E-03	4.44E-01	9.98E-01	9.47E-02	6.89E-01
120562	0.01(0.15)	0.16(0.13)	0.66(0.20)	0.15(0.19)	0.65(0.24)	0.50(0.23)	4.48E-01	8.86E-03	3.38E-02	9.98E-01	1.04E-01	2.34E-01
7718	0.01(0.10)	-0.09(0.24)	1.06(0.38)	-0.10(0.26)	1.04(0.39)	1.15(0.45)	6.86E-01	9.90E-03	1.27E-02	9.98E-01	1.10E-01	1.53E-01
473	-0.04(0.16)	0.11(0.12)	0.71(0.24)	0.15(0.20)	0.75(0.28)	0.60(0.26)	4.47E-01	1.04E-02	2.74E-02	9.98E-01	1.10E-01	2.20E-01
132379	-0.10(0.16)	0.09(0.16)	0.49(0.16)	0.19(0.22)	0.59(0.22)	0.40(0.23)	3.92E-01	1.06E-02	8.41E-02	9.98E-01	1.10E-01	3.20E-01
479	0.01(0.16)	0.15(0.13)	0.64(0.19)	0.14(0.20)	0.63(0.24)	0.49(0.23)	4.79E-01	1.06E-02	3.54E-02	9.98E-01	1.10E-01	2.34E-01
107960	0.00(0.17)	0.06(0.12)	0.72(0.22)	0.07(0.20)	0.72(0.28)	0.65(0.25)	7.34E-01	1.12E-02	1.03E-02	9.98E-01	1.11E-01	1.37E-01
17263	-0.03(0.14)	0.17(0.15)	0.72(0.26)	0.19(0.20)	0.74(0.29)	0.55(0.30)	3.31E-01	1.17E-02	6.93E-02	9.98E-01	1.11E-01	2.95E-01
41683	0.15(0.15)	-0.16(0.35)	-0.36(0.13)	-0.32(0.38)	-0.51(0.20)	-0.20(0.37)	4.05E-01	1.28E-02	6.01E-01	9.98E-01	1.18E-01	8.02E-01
121890	-0.03(0.09)	0.05(0.59)	1.40(0.57)	0.08(0.59)	1.43(0.58)	1.35(0.83)	8.91E-01	1.55E-02	1.08E-01	9.98E-01	1.36E-01	3.59E-01
3258	0.00(0.11)	0.17(0.19)	0.68(0.25)	0.17(0.22)	0.68(0.27)	0.50(0.31)	4.32E-01	1.61E-02	1.08E-01	9.98E-01	1.38E-01	3.59E-01
1872	0.04(0.13)	0.14(0.18)	0.51(0.15)	0.09(0.21)	0.47(0.19)	0.38(0.22)	6.72E-01	1.77E-02	9.55E-02	9.98E-01	1.48E-01	3.40E-01
47	0.11(0.15)	0.01(0.16)	0.56(0.13)	-0.10(0.21)	0.46(0.19)	0.56(0.20)	6.29E-01	1.94E-02	6.79E-03	9.98E-01	1.56E-01	1.22E-01
490	-0.01(0.15)	0.07(0.13)	0.64(0.23)	0.08(0.19)	0.65(0.27)	0.57(0.26)	6.61E-01	1.94E-02	3.44E-02	9.98E-01	1.56E-01	2.34E-01
5346	-0.07(0.13)	0.11(0.19)	0.41(0.15)	0.18(0.23)	0.47(0.20)	0.29(0.24)	4.29E-01	2.14E-02	2.19E-01	9.98E-01	1.58E-01	4.81E-01
1878	0.03(0.13)	0.14(0.16)	0.53(0.18)	0.12(0.20)	0.51(0.22)	0.39(0.24)	5.56E-01	2.34E-02	1.05E-01	9.98E-01	1.63E-01	3.56E-01

4937	-0.05(0.14)	0.11(0.14)	0.63(0.27)	0.16(0.20)	0.69(0.30)	0.53(0.31)	4.29E-01	2.35E-02	8.86E-02	9.98E-01	1.63E-01	3.21E-01
6864	-0.01(0.17)	0.09(0.12)	0.64(0.24)	0.10(0.20)	0.65(0.29)	0.54(0.27)	6.09E-01	2.66E-02	4.72E-02	9.98E-01	1.76E-01	2.48E-01
1700	-0.15(0.28)	0.06(0.10)	1.19(0.52)	0.21(0.30)	1.34(0.59)	1.13(0.53)	4.81E-01	2.73E-02	3.78E-02	9.98E-01	1.76E-01	2.38E-01
4943	0.00(0.16)	0.07(0.12)	0.63(0.24)	0.08(0.19)	0.64(0.28)	0.56(0.26)	6.92E-01	2.85E-02	3.88E-02	9.98E-01	1.79E-01	2.38E-01
117141	0.04(0.15)	0.04(0.16)	0.50(0.13)	-0.00(0.22)	0.46(0.21)	0.45(0.20)	9.96E-01	3.03E-02	2.68E-02	9.99E-01	1.87E-01	2.20E-01
9392	-0.01(0.15)	-0.06(0.18)	-0.53(0.18)	-0.05(0.22)	-0.52(0.24)	-0.47(0.26)	8.26E-01	3.07E-02	7.10E-02	9.98E-01	1.87E-01	2.97E-01
1996	-0.01(0.17)	0.10(0.14)	0.48(0.15)	0.11(0.22)	0.48(0.22)	0.38(0.20)	6.26E-01	3.51E-02	6.95E-02	9.98E-01	2.00E-01	2.95E-01
3286	-0.01(0.15)	0.11(0.13)	0.71(0.32)	0.12(0.19)	0.73(0.34)	0.60(0.34)	5.19E-01	3.66E-02	7.74E-02	9.98E-01	2.00E-01	3.08E-01
87769	-0.08(0.14)	0.11(0.14)	0.48(0.22)	0.19(0.20)	0.56(0.26)	0.38(0.26)	3.47E-01	3.75E-02	1.57E-01	9.98E-01	2.00E-01	4.30E-01
4936	-0.05(0.14)	0.14(0.15)	1.97(0.95)	0.19(0.20)	2.01(0.95)	1.83(0.95)	3.61E-01	3.82E-02	5.88E-02	9.98E-01	2.00E-01	2.80E-01
5288	0.07(0.16)	0.11(0.12)	0.65(0.23)	0.03(0.20)	0.57(0.27)	0.54(0.25)	8.66E-01	3.86E-02	3.75E-02	9.98E-01	2.00E-01	2.38E-01
122151	0.01(0.15)	0.14(0.15)	0.52(0.20)	0.13(0.20)	0.51(0.24)	0.37(0.24)	5.10E-01	3.90E-02	1.27E-01	9.98E-01	2.00E-01	3.79E-01
131620	0.10(0.14)	-0.10(0.13)	0.56(0.17)	-0.19(0.20)	0.46(0.22)	0.65(0.21)	3.31E-01	3.90E-02	3.34E-03	9.98E-01	2.00E-01	8.75E-02
62	0.07(0.15)	-0.08(0.16)	0.49(0.12)	-0.15(0.22)	0.42(0.20)	0.57(0.20)	4.99E-01	3.97E-02	6.71E-03	9.98E-01	2.00E-01	1.22E-01
41691	0.04(0.17)	0.09(0.12)	0.57(0.19)	0.05(0.21)	0.53(0.25)	0.48(0.22)	8.03E-01	4.00E-02	3.49E-02	9.98E-01	2.00E-01	2.34E-01
41697	0.00(0.17)	0.09(0.12)	0.59(0.24)	0.09(0.20)	0.59(0.28)	0.50(0.27)	6.57E-01	4.02E-02	6.39E-02	9.98E-01	2.00E-01	2.87E-01
120526	0.02(0.15)	0.14(0.15)	0.52(0.20)	0.12(0.20)	0.50(0.24)	0.38(0.24)	5.48E-01	4.04E-02	1.25E-01	9.98E-01	2.00E-01	3.78E-01
135260	0.03(0.14)	0.17(0.18)	0.44(0.14)	0.14(0.22)	0.41(0.20)	0.27(0.22)	5.20E-01	4.09E-02	2.36E-01	9.98E-01	2.00E-01	5.03E-01
93947	-0.12(0.23)	0.05(0.11)	0.48(0.18)	0.17(0.25)	0.60(0.29)	0.43(0.21)	5.02E-01	4.24E-02	4.52E-02	9.98E-01	2.05E-01	2.47E-01
5482	0.01(0.19)	0.09(0.11)	0.65(0.27)	0.08(0.21)	0.65(0.31)	0.56(0.28)	6.94E-01	4.30E-02	5.30E-02	9.98E-01	2.05E-01	2.66E-01
3029	-0.04(0.09)	0.15(0.49)	-2.03(0.98)	0.18(0.50)	-2.00(0.99)	-2.18(1.09)	7.19E-01	4.69E-02	4.90E-02	9.98E-01	2.13E-01	2.53E-01
139467	0.04(0.19)	0.08(0.12)	0.58(0.21)	0.05(0.22)	0.55(0.27)	0.50(0.24)	8.32E-01	4.90E-02	4.23E-02	9.98E-01	2.20E-01	2.41E-01
46292	0.00(0.19)	0.05(0.13)	0.53(0.20)	0.05(0.23)	0.53(0.26)	0.47(0.23)	8.12E-01	5.00E-02	4.59E-02	9.98E-01	2.22E-01	2.47E-01
3232	-0.06(0.14)	0.05(0.17)	0.34(0.16)	0.12(0.21)	0.41(0.21)	0.29(0.22)	5.92E-01	5.37E-02	1.96E-01	9.98E-01	2.33E-01	4.66E-01
4932	0.07(0.17)	-0.12(0.14)	-0.35(0.14)	-0.19(0.22)	-0.42(0.23)	-0.23(0.20)	3.98E-01	6.57E-02	2.56E-01	9.98E-01	2.77E-01	5.17E-01
93385	0.01(0.20)	0.09(0.14)	0.45(0.14)	0.08(0.24)	0.44(0.24)	0.36(0.20)	7.35E-01	6.81E-02	7.51E-02	9.98E-01	2.81E-01	3.08E-01
6579	-0.01(0.12)	0.12(0.16)	0.77(0.41)	0.13(0.19)	0.78(0.42)	0.65(0.44)	4.93E-01	6.87E-02	1.41E-01	9.98E-01	2.81E-01	4.05E-01
146957	0.08(0.15)	-0.06(0.13)	0.50(0.17)	-0.15(0.20)	0.41(0.22)	0.56(0.21)	4.60E-01	6.93E-02	9.60E-03	9.98E-01	2.81E-01	1.37E-01
112487	0.02(0.14)	0.26(0.19)	0.39(0.13)	0.24(0.24)	0.37(0.20)	0.13(0.23)	3.22E-01	7.02E-02	5.78E-01	9.98E-01	2.81E-01	7.88E-01

16561	0.04(0.15)	0.13(0.13)	0.60(0.28)	0.08(0.19)	0.56(0.31)	0.48(0.31)	6.62E-01	7.09E-02	1.24E-01	9.98E-01	2.81E-01	3.78E-01
42357	-0.03(0.11)	0.07(0.22)	0.52(0.28)	0.11(0.24)	0.55(0.30)	0.44(0.36)	6.52E-01	7.25E-02	2.20E-01	9.98E-01	2.85E-01	4.81E-01
17453	-0.05(0.13)	0.15(0.18)	0.33(0.17)	0.20(0.22)	0.38(0.21)	0.18(0.25)	3.60E-01	7.57E-02	4.71E-01	9.98E-01	2.92E-01	7.13E-01
4863	-0.06(0.13)	-0.01(0.23)	0.29(0.14)	0.05(0.27)	0.35(0.19)	0.30(0.27)	8.53E-01	7.64E-02	2.69E-01	9.98E-01	2.92E-01	5.22E-01
1709	0.00(0.14)	0.02(0.18)	0.36(0.16)	0.03(0.23)	0.37(0.20)	0.34(0.24)	9.05E-01	7.78E-02	1.56E-01	9.98E-01	2.92E-01	4.30E-01
4927	-0.05(0.13)	0.19(0.18)	-2.80(1.56)	0.24(0.22)	-2.75(1.56)	-3.00(1.56)	2.72E-01	8.28E-02	5.94E-02	9.98E-01	2.99E-01	2.80E-01
4945	-0.07(0.16)	0.02(0.13)	0.36(0.19)	0.08(0.21)	0.42(0.24)	0.34(0.23)	6.84E-01	8.37E-02	1.53E-01	9.98E-01	2.99E-01	4.29E-01
130396	-0.02(0.18)	0.03(0.13)	0.41(0.18)	0.05(0.21)	0.43(0.25)	0.38(0.22)	8.05E-01	8.40E-02	8.76E-02	9.98E-01	2.99E-01	3.21E-01
61	-0.08(0.18)	0.28(0.25)	0.29(0.11)	0.36(0.31)	0.37(0.21)	0.01(0.28)	2.54E-01	8.53E-02	9.65E-01	9.98E-01	2.99E-01	9.94E-01
4942	-0.08(0.17)	0.07(0.12)	0.44(0.25)	0.15(0.20)	0.51(0.29)	0.36(0.27)	4.57E-01	8.56E-02	1.89E-01	9.98E-01	2.99E-01	4.63E-01
134	0.07(0.12)	-0.04(0.19)	-0.36(0.22)	-0.11(0.22)	-0.43(0.25)	-0.32(0.28)	6.17E-01	8.77E-02	2.64E-01	9.98E-01	3.03E-01	5.22E-01
592	0.02(0.20)	0.13(0.14)	0.44(0.14)	0.11(0.24)	0.42(0.24)	0.31(0.20)	6.37E-01	8.94E-02	1.35E-01	9.98E-01	3.05E-01	4.00E-01
88583	0.02(0.14)	-0.22(0.16)	0.37(0.15)	-0.24(0.21)	0.35(0.20)	0.59(0.22)	2.61E-01	8.98E-02	9.36E-03	9.98E-01	3.05E-01	1.37E-01
18488	-0.03(0.18)	0.11(0.12)	0.41(0.19)	0.14(0.21)	0.43(0.25)	0.29(0.22)	5.09E-01	9.08E-02	1.96E-01	9.98E-01	3.06E-01	4.66E-01
453	0.02(0.14)	0.13(0.15)	-0.42(0.22)	0.12(0.21)	-0.43(0.26)	-0.55(0.27)	5.68E-01	1.03E-01	4.29E-02	9.98E-01	3.27E-01	2.41E-01
6538	-0.15(0.15)	-0.01(0.17)	0.22(0.17)	0.14(0.22)	0.37(0.22)	0.23(0.24)	5.39E-01	1.04E-01	3.38E-01	9.98E-01	3.27E-01	5.91E-01
132414	-0.04(0.18)	0.06(0.12)	0.46(0.26)	0.10(0.21)	0.50(0.31)	0.40(0.28)	6.29E-01	1.08E-01	1.62E-01	9.98E-01	3.35E-01	4.30E-01
98027	0.08(0.14)	0.02(0.23)	0.74(0.38)	-0.06(0.28)	0.66(0.41)	0.72(0.45)	8.22E-01	1.14E-01	1.11E-01	9.98E-01	3.42E-01	3.59E-01
4981	-0.13(0.27)	0.08(0.11)	0.49(0.28)	0.20(0.29)	0.61(0.38)	0.41(0.30)	4.83E-01	1.14E-01	1.75E-01	9.98E-01	3.42E-01	4.42E-01
1875	0.03(0.15)	0.20(0.13)	0.42(0.20)	0.17(0.20)	0.39(0.25)	0.22(0.24)	3.85E-01	1.15E-01	3.55E-01	9.98E-01	3.42E-01	6.03E-01
3329	0.02(0.13)	0.08(0.15)	0.61(0.36)	0.06(0.20)	0.59(0.37)	0.53(0.39)	7.50E-01	1.16E-01	1.77E-01	9.98E-01	3.42E-01	4.44E-01
7053	0.00(0.15)	-0.24(0.18)	0.35(0.17)	-0.24(0.23)	0.35(0.23)	0.59(0.25)	2.96E-01	1.28E-01	2.12E-02	9.98E-01	3.66E-01	1.86E-01
110	0.04(0.25)	0.10(0.15)	0.49(0.16)	0.07(0.29)	0.45(0.29)	0.39(0.22)	8.18E-01	1.31E-01	7.85E-02	9.98E-01	3.68E-01	3.08E-01
106936	-0.02(0.17)	0.09(0.14)	0.35(0.18)	0.11(0.21)	0.37(0.24)	0.26(0.22)	6.09E-01	1.32E-01	2.52E-01	9.98E-01	3.68E-01	5.17E-01
1029	0.14(0.20)	-0.05(0.14)	0.50(0.13)	-0.19(0.23)	0.36(0.23)	0.54(0.19)	4.30E-01	1.33E-01	4.82E-03	9.98E-01	3.68E-01	1.07E-01
31357	0.02(0.12)	0.28(0.21)	-0.30(0.17)	0.26(0.24)	-0.31(0.21)	-0.58(0.27)	2.76E-01	1.34E-01	3.81E-02	9.98E-01	3.68E-01	2.38E-01
2061	0.16(0.17)	0.08(0.14)	0.48(0.13)	-0.08(0.22)	0.32(0.21)	0.40(0.20)	7.19E-01	1.41E-01	5.13E-02	9.98E-01	3.78E-01	2.61E-01
5930	0.01(0.13)	0.02(0.19)	0.49(0.30)	0.01(0.23)	0.48(0.33)	0.47(0.36)	9.62E-01	1.48E-01	2.00E-01	9.98E-01	3.91E-01	4.66E-01
307	-0.03(0.16)	0.13(0.14)	0.32(0.18)	0.16(0.21)	0.35(0.24)	0.19(0.24)	4.46E-01	1.52E-01	4.31E-01	9.98E-01	3.97E-01	6.76E-01

135889	0.08(0.20)	-0.07(0.19)	-5.33(3.72)	-0.15(0.27)	-5.41(3.75)	-5.26(3.73)	5.84E-01	1.54E-01	1.63E-01	9.98E-01	3.97E-01	4.30E-01
32247	-0.04(0.18)	0.14(0.15)	0.29(0.15)	0.18(0.23)	0.33(0.23)	0.15(0.21)	4.34E-01	1.56E-01	4.63E-01	9.98E-01	3.97E-01	7.06E-01
4985	-0.13(0.32)	0.08(0.16)	0.38(0.18)	0.21(0.35)	0.52(0.36)	0.31(0.24)	5.51E-01	1.60E-01	2.03E-01	9.98E-01	4.02E-01	4.66E-01
6526	-0.02(0.25)	0.08(0.11)	0.47(0.26)	0.10(0.26)	0.49(0.35)	0.39(0.28)	7.17E-01	1.65E-01	1.71E-01	9.98E-01	4.11E-01	4.42E-01
41699	-0.03(0.19)	0.12(0.13)	0.35(0.20)	0.15(0.22)	0.38(0.27)	0.22(0.23)	4.99E-01	1.66E-01	3.42E-01	9.98E-01	4.12E-01	5.94E-01
4983	-0.01(0.19)	0.04(0.12)	0.41(0.23)	0.04(0.22)	0.41(0.30)	0.37(0.27)	8.43E-01	1.69E-01	1.68E-01	9.98E-01	4.15E-01	4.41E-01
95550	0.01(0.15)	0.18(0.20)	0.29(0.14)	0.18(0.25)	0.28(0.20)	0.10(0.26)	4.91E-01	1.71E-01	6.87E-01	9.98E-01	4.19E-01	8.24E-01
4971	-0.01(0.15)	0.04(0.15)	0.32(0.19)	0.04(0.21)	0.32(0.24)	0.28(0.24)	8.45E-01	1.77E-01	2.37E-01	9.98E-01	4.29E-01	5.03E-01
4577	-0.02(0.17)	-0.07(0.15)	0.30(0.17)	-0.06(0.23)	0.32(0.23)	0.37(0.23)	8.10E-01	1.81E-01	1.03E-01	9.98E-01	4.29E-01	3.56E-01
87710	-0.01(0.13)	0.19(0.21)	-0.30(0.17)	0.20(0.25)	-0.30(0.22)	-0.49(0.28)	4.41E-01	1.82E-01	8.40E-02	9.98E-01	4.29E-01	3.20E-01
469	-0.07(0.16)	-0.19(0.15)	0.22(0.15)	-0.11(0.22)	0.29(0.22)	0.40(0.21)	6.13E-01	1.95E-01	6.36E-02	9.98E-01	4.53E-01	2.87E-01
6331	-0.04(0.10)	0.34(0.39)	0.57(0.47)	0.38(0.40)	0.61(0.48)	0.23(0.60)	3.43E-01	2.09E-01	7.01E-01	9.98E-01	4.77E-01	8.39E-01
4925	0.03(0.16)	0.15(0.13)	0.35(0.22)	0.12(0.21)	0.33(0.27)	0.20(0.25)	5.47E-01	2.31E-01	4.28E-01	9.98E-01	5.11E-01	6.76E-01
103102	0.11(0.16)	0.16(0.16)	0.35(0.14)	0.05(0.22)	0.25(0.21)	0.19(0.21)	8.09E-01	2.35E-01	3.59E-01	9.98E-01	5.16E-01	6.07E-01
91	-0.01(0.19)	0.01(0.15)	0.27(0.14)	0.02(0.24)	0.29(0.24)	0.26(0.20)	9.27E-01	2.40E-01	1.94E-01	9.98E-01	5.23E-01	4.66E-01
443	0.07(0.17)	-0.09(0.14)	0.35(0.15)	-0.16(0.22)	0.27(0.23)	0.44(0.21)	4.61E-01	2.42E-01	3.91E-02	9.98E-01	5.24E-01	2.38E-01
41708	0.02(0.22)	0.09(0.12)	0.36(0.21)	0.07(0.24)	0.33(0.29)	0.27(0.24)	7.87E-01	2.58E-01	2.69E-01	9.98E-01	5.50E-01	5.22E-01
2900	0.07(0.16)	-0.06(0.16)	0.36(0.20)	-0.13(0.23)	0.29(0.25)	0.41(0.26)	5.88E-01	2.60E-01	1.13E-01	9.98E-01	5.52E-01	3.60E-01
5483	0.01(0.15)	0.09(0.14)	0.34(0.25)	0.08(0.20)	0.32(0.29)	0.25(0.28)	6.90E-01	2.62E-01	3.91E-01	9.98E-01	5.52E-01	6.53E-01
1064	0.01(0.12)	0.14(0.22)	-0.51(0.45)	0.13(0.25)	-0.52(0.46)	-0.65(0.50)	5.92E-01	2.69E-01	2.03E-01	9.98E-01	5.63E-01	4.66E-01
39	-0.03(0.14)	0.18(0.17)	0.23(0.20)	0.21(0.22)	0.26(0.24)	0.05(0.26)	3.48E-01	2.72E-01	8.44E-01	9.98E-01	5.63E-01	9.34E-01
657	-0.03(0.18)	0.02(0.13)	0.25(0.18)	0.05(0.22)	0.28(0.26)	0.23(0.22)	8.29E-01	2.75E-01	2.89E-01	9.98E-01	5.66E-01	5.44E-01
121002	-0.06(0.15)	0.02(0.16)	-0.30(0.17)	0.08(0.23)	-0.24(0.22)	-0.32(0.24)	7.12E-01	2.78E-01	1.80E-01	9.98E-01	5.70E-01	4.50E-01
8607	-0.07(0.19)	-0.04(0.23)	-0.31(0.11)	0.03(0.29)	-0.24(0.22)	-0.27(0.26)	9.24E-01	2.80E-01	3.02E-01	9.98E-01	5.70E-01	5.64E-01
26918	-0.03(0.09)	0.56(1.18)	8.99(8.32)	0.59(1.18)	9.02(8.32)	8.43(8.37)	6.19E-01	2.82E-01	3.18E-01	9.98E-01	5.73E-01	5.75E-01
91421	0.01(0.15)	0.11(0.18)	-0.25(0.19)	0.10(0.24)	-0.26(0.24)	-0.36(0.25)	6.72E-01	2.89E-01	1.63E-01	9.98E-01	5.80E-01	4.30E-01
119023	0.02(0.29)	0.00(0.15)	0.34(0.12)	-0.02(0.33)	0.32(0.31)	0.34(0.19)	9.52E-01	2.98E-01	6.93E-02	9.98E-01	5.94E-01	2.95E-01
2011	0.00(0.16)	0.13(0.17)	0.22(0.15)	0.13(0.22)	0.22(0.22)	0.10(0.23)	5.64E-01	3.04E-01	6.80E-01	9.98E-01	5.95E-01	8.21E-01
132976	-0.04(0.16)	0.05(0.18)	0.18(0.14)	0.08(0.24)	0.22(0.21)	0.14(0.22)	7.25E-01	3.06E-01	5.48E-01	9.98E-01	5.95E-01	7.63E-01

4533	-0.05(0.24)	0.03(0.20)	-0.32(0.12)	0.08(0.31)	-0.27(0.26)	-0.35(0.23)	8.05E-01	3.11E-01	1.39E-01	9.98E-01	5.99E-01	4.04E-01
5419	-0.01(0.37)	0.09(0.14)	0.39(0.13)	0.10(0.39)	0.40(0.39)	0.30(0.19)	7.96E-01	3.14E-01	1.23E-01	9.98E-01	6.00E-01	3.78E-01
135619	0.03(0.20)	0.17(0.19)	0.27(0.13)	0.14(0.29)	0.24(0.25)	0.10(0.22)	6.37E-01	3.44E-01	6.60E-01	9.98E-01	6.36E-01	8.13E-01
8270	0.04(0.15)	0.04(0.24)	-0.16(0.14)	-0.00(0.28)	-0.20(0.21)	-0.20(0.27)	9.95E-01	3.44E-01	4.76E-01	9.99E-01	6.36E-01	7.13E-01
126397	0.04(0.13)	0.04(0.15)	0.31(0.26)	-0.00(0.20)	0.27(0.28)	0.26(0.29)	9.94E-01	3.49E-01	3.70E-01	9.99E-01	6.36E-01	6.23E-01
3465	0.05(0.13)	-0.05(0.19)	-0.17(0.19)	-0.10(0.23)	-0.22(0.23)	-0.12(0.26)	6.73E-01	3.49E-01	6.39E-01	9.98E-01	6.36E-01	8.03E-01
6580	0.06(0.15)	0.13(0.14)	0.33(0.25)	0.07(0.20)	0.27(0.28)	0.20(0.28)	7.18E-01	3.49E-01	4.92E-01	9.98E-01	6.36E-01	7.17E-01
4941	-0.01(0.17)	0.05(0.14)	0.28(0.28)	0.06(0.21)	0.30(0.32)	0.24(0.31)	7.81E-01	3.57E-01	4.47E-01	9.98E-01	6.47E-01	6.90E-01
119066	0.01(0.15)	-0.09(0.20)	0.20(0.15)	-0.10(0.26)	0.19(0.21)	0.29(0.26)	7.05E-01	3.75E-01	2.70E-01	9.98E-01	6.76E-01	5.22E-01
160543	0.01(0.28)	0.20(0.18)	-0.26(0.13)	0.19(0.32)	-0.27(0.30)	-0.45(0.22)	5.61E-01	3.80E-01	4.57E-02	9.98E-01	6.82E-01	2.47E-01
106629	0.01(0.14)	0.11(0.15)	0.24(0.23)	0.10(0.21)	0.23(0.27)	0.13(0.27)	6.34E-01	3.93E-01	6.33E-01	9.98E-01	6.84E-01	8.02E-01
62433	0.08(0.15)	0.14(0.16)	0.27(0.15)	0.06(0.22)	0.19(0.22)	0.12(0.22)	7.78E-01	3.97E-01	5.82E-01	9.98E-01	6.86E-01	7.88E-01
16788	0.11(0.26)	0.01(0.17)	0.35(0.11)	-0.10(0.30)	0.24(0.28)	0.34(0.20)	7.35E-01	4.01E-01	9.07E-02	9.98E-01	6.89E-01	3.26E-01
159824	0.02(0.17)	-0.04(0.15)	-0.17(0.15)	-0.07(0.23)	-0.20(0.23)	-0.13(0.22)	7.82E-01	4.02E-01	5.48E-01	9.98E-01	6.89E-01	7.63E-01
117171	0.01(0.14)	-0.02(0.20)	0.18(0.16)	-0.02(0.24)	0.18(0.21)	0.20(0.25)	9.19E-01	4.06E-01	4.33E-01	9.98E-01	6.89E-01	6.76E-01
17664	0.01(0.17)	0.06(0.13)	0.22(0.18)	0.05(0.22)	0.21(0.25)	0.16(0.22)	8.20E-01	4.09E-01	4.77E-01	9.98E-01	6.91E-01	7.13E-01
17068	0.15(0.14)	0.00(0.16)	0.33(0.17)	-0.15(0.22)	0.17(0.21)	0.33(0.23)	4.82E-01	4.21E-01	1.58E-01	9.98E-01	7.02E-01	4.30E-01
137	-0.04(0.17)	0.12(0.17)	-0.22(0.15)	0.16(0.24)	-0.18(0.22)	-0.34(0.23)	4.94E-01	4.29E-01	1.39E-01	9.98E-01	7.11E-01	4.04E-01
4975	0.00(0.13)	0.15(0.18)	-0.17(0.16)	0.15(0.22)	-0.17(0.21)	-0.31(0.24)	5.18E-01	4.30E-01	2.05E-01	9.98E-01	7.11E-01	4.68E-01
4609	-0.25(0.66)	0.04(0.18)	0.29(0.18)	0.28(0.68)	0.54(0.68)	0.25(0.25)	6.79E-01	4.33E-01	3.16E-01	9.98E-01	7.11E-01	5.75E-01
4922	-0.08(0.45)	0.03(0.15)	0.29(0.17)	0.11(0.47)	0.37(0.47)	0.26(0.22)	8.18E-01	4.42E-01	2.52E-01	9.98E-01	7.15E-01	5.17E-01
6115	-0.10(0.14)	0.10(0.16)	-0.30(0.23)	0.20(0.21)	-0.21(0.27)	-0.40(0.28)	3.43E-01	4.44E-01	1.51E-01	9.98E-01	7.15E-01	4.29E-01
4986	-0.09(0.23)	0.05(0.16)	-0.30(0.14)	0.14(0.28)	-0.20(0.27)	-0.35(0.21)	6.03E-01	4.50E-01	1.10E-01	9.98E-01	7.21E-01	3.59E-01
4929	0.00(0.27)	0.10(0.14)	-0.23(0.15)	0.10(0.30)	-0.23(0.31)	-0.33(0.21)	7.40E-01	4.56E-01	1.14E-01	9.98E-01	7.24E-01	3.62E-01
100253	0.10(0.23)	0.04(0.12)	0.31(0.17)	-0.05(0.26)	0.21(0.28)	0.26(0.21)	8.37E-01	4.62E-01	2.17E-01	9.98E-01	7.24E-01	4.81E-01
1725	-0.03(0.14)	-0.16(0.19)	0.13(0.17)	-0.13(0.23)	0.16(0.22)	0.29(0.25)	5.76E-01	4.63E-01	2.56E-01	9.98E-01	7.24E-01	5.17E-01
4546	0.10(0.34)	0.05(0.20)	-0.17(0.13)	-0.05(0.40)	-0.27(0.37)	-0.22(0.24)	9.05E-01	4.64E-01	3.55E-01	9.98E-01	7.24E-01	6.03E-01
6066	0.08(0.17)	-0.15(0.16)	0.24(0.14)	-0.23(0.24)	0.16(0.23)	0.39(0.21)	3.39E-01	4.76E-01	6.85E-02	9.98E-01	7.26E-01	2.95E-01
4871	-0.05(0.12)	0.22(0.32)	-0.19(0.16)	0.28(0.33)	-0.14(0.20)	-0.42(0.35)	4.11E-01	4.79E-01	2.39E-01	9.98E-01	7.26E-01	5.03E-01

4926	0.02(0.60)	-0.03(0.15)	-0.41(0.13)	-0.05(0.61)	-0.43(0.61)	-0.38(0.20)	9.33E-01	4.82E-01	6.36E-02	9.98E-01	7.26E-01	2.87E-01
47170	-0.04(0.17)	0.11(0.16)	0.12(0.16)	0.15(0.23)	0.16(0.23)	0.01(0.22)	5.26E-01	4.99E-01	9.65E-01	9.98E-01	7.44E-01	9.94E-01
4887	-0.02(0.18)	0.10(0.17)	-0.18(0.15)	0.12(0.25)	-0.16(0.24)	-0.28(0.23)	6.34E-01	5.08E-01	2.27E-01	9.98E-01	7.49E-01	4.89E-01
160	-0.02(0.24)	-0.26(0.17)	0.16(0.16)	-0.23(0.28)	0.18(0.28)	0.42(0.22)	4.10E-01	5.10E-01	6.72E-02	9.98E-01	7.49E-01	2.95E-01
153	-0.02(0.15)	-0.04(0.18)	0.12(0.16)	-0.02(0.24)	0.14(0.22)	0.16(0.25)	9.40E-01	5.11E-01	5.11E-01	9.98E-01	7.49E-01	7.35E-01
21666	0.01(0.17)	0.04(0.15)	0.15(0.15)	0.03(0.23)	0.14(0.23)	0.11(0.21)	8.91E-01	5.30E-01	6.02E-01	9.98E-01	7.66E-01	8.02E-01
109423	-0.05(0.11)	-1.09(1.10)	0.08(0.18)	-1.04(1.10)	0.13(0.21)	1.17(1.10)	3.49E-01	5.49E-01	2.94E-01	9.98E-01	7.90E-01	5.51E-01
68	0.05(0.15)	0.11(0.19)	0.17(0.14)	0.06(0.24)	0.12(0.21)	0.06(0.24)	8.08E-01	5.59E-01	7.93E-01	9.98E-01	7.98E-01	9.14E-01
257	0.03(0.18)	-0.05(0.14)	-0.11(0.17)	-0.08(0.22)	-0.14(0.25)	-0.07(0.22)	7.36E-01	5.59E-01	7.54E-01	9.98E-01	7.98E-01	8.82E-01
4956	-0.06(0.17)	0.09(0.13)	-0.24(0.24)	0.15(0.21)	-0.17(0.30)	-0.33(0.27)	4.79E-01	5.62E-01	2.36E-01	9.98E-01	7.98E-01	5.03E-01
87720	0.03(0.18)	0.14(0.14)	0.17(0.16)	0.11(0.23)	0.14(0.24)	0.03(0.21)	6.42E-01	5.69E-01	8.78E-01	9.98E-01	7.99E-01	9.50E-01
31559	-0.23(0.24)	0.21(0.67)	-0.08(0.10)	0.44(0.70)	0.15(0.26)	-0.29(0.67)	5.36E-01	5.79E-01	6.66E-01	9.98E-01	8.05E-01	8.17E-01
17253	-0.27(0.53)	0.05(0.19)	0.04(0.15)	0.31(0.56)	0.31(0.56)	-0.01(0.24)	5.79E-01	5.83E-01	9.82E-01	9.98E-01	8.06E-01	9.94E-01
26916	-0.03(0.09)	0.83(1.18)	6.77(12.35)	0.86(1.18)	6.8(12.35)	5.94(12.38)	4.69E-01	5.84E-01	6.33E-01	9.98E-01	8.06E-01	8.02E-01
1148	0.06(0.28)	-0.15(0.15)	0.21(0.13)	-0.20(0.32)	0.16(0.31)	0.36(0.20)	5.24E-01	6.10E-01	7.19E-02	9.98E-01	8.30E-01	2.98E-01
41808	-0.07(0.13)	-0.01(0.18)	-0.19(0.21)	0.06(0.23)	-0.12(0.24)	-0.18(0.28)	7.83E-01	6.16E-01	5.16E-01	9.98E-01	8.36E-01	7.40E-01
7403	0.28(1.33)	0.00(0.14)	0.94(0.20)	-0.27(1.34)	0.66(1.35)	0.94(0.24)	8.38E-01	6.24E-01	2.11E-04	9.98E-01	8.43E-01	1.83E-02
7440	0.05(1.61)	-0.04(0.12)	0.82(0.15)	-0.10(1.61)	0.76(1.62)	0.86(0.19)	9.52E-01	6.38E-01	3.58E-05	9.98E-01	8.46E-01	1.26E-02
4938	0.02(0.19)	0.12(0.13)	0.19(0.33)	0.09(0.22)	0.17(0.36)	0.08(0.35)	6.82E-01	6.42E-01	8.25E-01	9.98E-01	8.46E-01	9.33E-01
2001	0.03(0.19)	0.04(0.15)	0.15(0.16)	0.01(0.24)	0.12(0.25)	0.11(0.21)	9.73E-01	6.42E-01	6.14E-01	9.98E-01	8.46E-01	8.02E-01
2806	-0.76(1.59)	0.03(0.17)	-0.05(0.16)	0.78(1.61)	0.71(1.60)	-0.08(0.23)	6.28E-01	6.60E-01	7.45E-01	9.98E-01	8.64E-01	8.75E-01
145492	-0.05(0.23)	0.09(0.16)	0.07(0.15)	0.14(0.28)	0.12(0.27)	-0.02(0.21)	6.21E-01	6.62E-01	9.30E-01	9.98E-01	8.64E-01	9.79E-01
1981	-0.05(0.11)	0.14(0.37)	0.04(0.19)	0.19(0.39)	0.09(0.22)	-0.10(0.42)	6.18E-01	6.83E-01	8.05E-01	9.98E-01	8.82E-01	9.25E-01
573	-0.02(0.13)	0.21(0.26)	-0.10(0.17)	0.23(0.29)	-0.08(0.21)	-0.31(0.31)	4.39E-01	6.93E-01	3.22E-01	9.98E-01	8.88E-01	5.75E-01
5085	0.14(0.32)	-0.04(0.21)	0.00(0.13)	-0.17(0.39)	-0.13(0.34)	0.04(0.25)	6.58E-01	7.02E-01	8.72E-01	9.98E-01	8.94E-01	9.50E-01
133943	-0.03(0.33)	0.08(0.16)	0.09(0.15)	0.11(0.36)	0.12(0.35)	0.01(0.22)	7.57E-01	7.29E-01	9.59E-01	9.98E-01	9.21E-01	9.93E-01
54	-0.09(0.16)	0.07(0.14)	-0.19(0.23)	0.16(0.20)	-0.09(0.27)	-0.26(0.26)	4.28E-01	7.35E-01	3.38E-01	9.98E-01	9.21E-01	5.91E-01
4928	-0.16(1.00)	0.03(0.15)	0.16(0.19)	0.19(1.01)	0.32(1.01)	0.13(0.24)	8.54E-01	7.52E-01	5.83E-01	9.98E-01	9.23E-01	7.88E-01
892	-0.01(0.19)	0.11(0.12)	-0.11(0.29)	0.11(0.22)	-0.11(0.34)	-0.22(0.32)	6.19E-01	7.60E-01	4.92E-01	9.98E-01	9.24E-01	7.17E-01

20903	0.05(0.31)	-0.01(0.12)	0.16(0.20)	-0.06(0.33)	0.11(0.35)	0.17(0.23)	8.58E-01	7.61E-01	4.61E-01	9.98E-01	9.24E-01	7.06E-01
4898	-0.01(0.14)	0.05(0.20)	0.06(0.16)	0.06(0.24)	0.06(0.21)	0.00(0.25)	8.09E-01	7.62E-01	9.85E-01	9.98E-01	9.24E-01	9.94E-01
9320	-0.02(0.15)	0.08(0.22)	0.04(0.14)	0.10(0.27)	0.06(0.21)	-0.04(0.26)	7.18E-01	7.72E-01	8.87E-01	9.98E-01	9.33E-01	9.55E-01
1912	0.02(0.14)	-0.04(0.17)	0.08(0.18)	-0.05(0.22)	0.06(0.23)	0.12(0.24)	8.07E-01	7.79E-01	6.28E-01	9.98E-01	9.33E-01	8.02E-01
2936	0.04(0.20)	-0.11(0.20)	-0.03(0.13)	-0.15(0.28)	-0.07(0.24)	0.08(0.24)	5.94E-01	7.82E-01	7.20E-01	9.98E-01	9.33E-01	8.57E-01
34075	0.07(0.16)	-0.29(0.15)	0.13(0.16)	-0.35(0.22)	0.06(0.22)	0.42(0.22)	1.14E-01	7.84E-01	6.18E-02	9.98E-01	9.33E-01	2.87E-01
119129	0.99(3.36)	0.06(0.16)	0.11(0.11)	-0.93(3.37)	-0.89(3.36)	0.04(0.19)	7.83E-01	7.93E-01	8.19E-01	9.98E-01	9.37E-01	9.30E-01
127277	-0.03(0.14)	-0.24(0.15)	-0.27(1.07)	-0.21(0.20)	-0.25(1.07)	-0.04(1.08)	2.99E-01	8.17E-01	9.73E-01	9.98E-01	9.44E-01	9.94E-01
1704	-0.14(0.19)	0.05(0.13)	-0.19(0.17)	0.18(0.23)	-0.06(0.25)	-0.24(0.21)	4.24E-01	8.18E-01	2.59E-01	9.98E-01	9.44E-01	5.17E-01
139436	0.03(0.48)	0.15(0.24)	-0.08(0.12)	0.12(0.53)	-0.11(0.49)	-0.23(0.27)	8.25E-01	8.22E-01	3.99E-01	9.98E-01	9.44E-01	6.57E-01
111162	-0.44(3.88)	0.00(0.16)	0.41(0.13)	0.43(3.87)	0.84(3.87)	0.41(0.20)	9.12E-01	8.28E-01	4.73E-02	9.98E-01	9.46E-01	2.48E-01
4600	0.00(0.21)	-0.09(0.25)	0.05(0.12)	-0.09(0.33)	0.05(0.23)	0.14(0.28)	7.83E-01	8.33E-01	6.14E-01	9.98E-01	9.48E-01	8.02E-01
7402	-0.04(0.81)	0.08(0.17)	0.13(0.19)	0.12(0.82)	0.17(0.83)	0.05(0.25)	8.86E-01	8.42E-01	8.52E-01	9.98E-01	9.48E-01	9.34E-01
1941	-0.01(0.16)	-0.13(0.15)	-0.06(0.17)	-0.11(0.22)	-0.05(0.23)	0.07(0.23)	6.09E-01	8.45E-01	7.71E-01	9.98E-01	9.48E-01	8.97E-01
139478	-0.35(1.66)	-0.02(0.16)	-0.02(0.22)	0.32(1.67)	0.33(1.68)	0.00(0.27)	8.47E-01	8.46E-01	9.90E-01	9.98E-01	9.48E-01	9.94E-01
6353	-0.17(0.24)	-0.04(0.13)	-0.12(0.19)	0.13(0.27)	0.05(0.29)	-0.08(0.22)	6.16E-01	8.52E-01	7.21E-01	9.98E-01	9.48E-01	8.57E-01
160463	0.05(0.17)	-0.06(0.14)	0.01(0.16)	-0.11(0.22)	-0.04(0.24)	0.07(0.21)	6.11E-01	8.60E-01	7.37E-01	9.98E-01	9.48E-01	8.71E-01
119167	0.11(0.21)	0.07(0.21)	0.07(0.12)	-0.04(0.31)	-0.04(0.25)	0.00(0.24)	9.01E-01	8.86E-01	9.91E-01	9.98E-01	9.60E-01	9.94E-01
915	0.86(2.21)	-0.01(0.16)	0.55(0.16)	-0.86(2.21)	-0.31(2.21)	0.56(0.22)	6.98E-01	8.90E-01	1.42E-02	9.98E-01	9.61E-01	1.53E-01
4933	0.00(0.11)	0.00(0.26)	0.03(0.22)	-0.00(0.29)	0.03(0.25)	0.03(0.33)	9.98E-01	9.00E-01	9.24E-01	9.99E-01	9.63E-01	9.79E-01
41682	0.05(0.14)	0.19(0.23)	0.07(0.15)	0.14(0.28)	0.03(0.21)	-0.11(0.27)	6.14E-01	9.03E-01	6.70E-01	9.98E-01	9.63E-01	8.18E-01
160464	0.23(2.25)	0.00(0.18)	-0.04(0.15)	-0.23(2.25)	-0.27(2.25)	-0.04(0.23)	9.18E-01	9.04E-01	8.76E-01	9.98E-01	9.63E-01	9.50E-01
6802	0.01(0.17)	0.13(0.13)	-0.03(0.32)	0.12(0.21)	-0.04(0.36)	-0.16(0.34)	5.61E-01	9.18E-01	6.44E-01	9.98E-01	9.72E-01	8.05E-01
64551	-0.08(0.19)	0.06(0.15)	-0.06(0.16)	0.14(0.24)	0.02(0.25)	-0.12(0.22)	5.52E-01	9.34E-01	5.68E-01	9.98E-01	9.74E-01	7.81E-01
1675	0.01(0.11)	0.53(0.76)	-0.01(0.16)	0.52(0.77)	-0.02(0.20)	-0.54(0.78)	5.02E-01	9.37E-01	4.92E-01	9.98E-01	9.74E-01	7.17E-01
4543	0.02(0.30)	0.03(0.15)	0.04(0.16)	0.01(0.34)	0.02(0.33)	0.01(0.22)	9.71E-01	9.42E-01	9.56E-01	9.98E-01	9.74E-01	9.93E-01
16792	0.03(0.13)	-0.29(0.19)	0.01(0.19)	-0.32(0.23)	-0.02(0.24)	0.30(0.27)	1.75E-01	9.43E-01	2.77E-01	9.98E-01	9.74E-01	5.27E-01
4948	0.00(0.20)	0.09(0.12)	0.02(0.23)	0.10(0.23)	0.02(0.31)	-0.07(0.26)	6.79E-01	9.45E-01	7.74E-01	9.98E-01	9.74E-01	8.98E-01
160542	-0.09(1.24)	0.17(0.26)	-0.01(0.11)	0.26(1.24)	0.08(1.24)	-0.18(0.28)	8.34E-01	9.48E-01	5.29E-01	9.98E-01	9.74E-01	7.48E-01

Regression coefficient β* (standard error (SE)) denotes the association between baseline level of each metabolite and change in HbA1c in each intervention group (IMI, BAND or RYGB). β† (SE) denotes the association between baseline level of each metabolite and change in HbA1c induced by different types of weight-loss interventions (BAND vs. IMI, RYGB vs. IMI, and RYGB vs. BAND), and was obtained from the interaction term (metabolite × types of intervention) in a linear regression model. In this model, % change in HbA1c (follow-up value minus baseline value, divided by baseline value) was the dependent variable, baseline level of each metabolite, intervention groups (1=IMI, 2=BAND, 3=RYGB, with IMI being the reference), and an interaction term (metabolite × types of intervention) were the independent variables, adjusting for baseline age, sex, BMI as well as weight loss. Abbreviations: IMI: Intensive medical intervention. BAND: Laparoscopic adjustable banding. RYGB: Roux-en-Y gastric bypass surgery.

Table S3. Differential association between changes in metabolites and change in FPG before and 1-yr after intervention

	β*(SE)			β†(SE)			p-value			q-value		
**Metabolites**	**IMI**	**BAND**	**RYGB**	**BAND *vs.* IMI**	**RYGB *vs.* IMI**	**RYGB *vs.* BAND**	**BAND *vs.* IMI**	**RYGB vs. IMI**	**RYGB *vs.* BAND**	**BAND *vs.* IMI**	**RYGB *vs.* IMI**	**RYGB *vs.* BAND**
**Known**												
Gluconic acid	-0.02(0.14)	-0.22(0.13)	2.95(0.47)	-0.21(0.19)	2.97(0.49)	3.17(0.49)	2.90E-01	1.02E-07	1.80E-08	9.96E-01	1.92E-05	3.40E-06
Fructose	-0.37(0.37)	-0.03(0.11)	3.91(0.71)	0.34(0.38)	4.28(0.81)	3.94(0.72)	3.80E-01	1.74E-06	8.88E-07	9.96E-01	2.19E-04	1.12E-04
Xylose	-0.03(0.12)	-0.39(0.35)	2.15(0.47)	-0.36(0.37)	2.17(0.48)	2.54(0.59)	3.29E-01	2.87E-05	5.92E-05	9.96E-01	1.98E-03	3.19E-03
Valine	0.01(0.15)	-0.01(0.24)	-0.97(0.17)	-0.03(0.28)	-0.98(0.22)	-0.95(0.29)	9.28E-01	4.80E-05	1.72E-03	9.96E-01	2.58E-03	2.70E-02
Glycine	-0.09(0.16)	-0.54(0.36)	-0.99(0.15)	-0.44(0.39)	-0.90(0.22)	-0.46(0.38)	2.62E-01	1.07E-04	2.37E-01	9.96E-01	3.71E-03	5.28E-01
Leucine	0.01(0.15)	-0.15(0.22)	-0.95(0.18)	-0.16(0.26)	-0.96(0.23)	-0.80(0.28)	5.49E-01	1.08E-04	6.56E-03	9.96E-01	3.71E-03	6.19E-02
Benzoic acid	-0.12(0.14)	-0.33(0.29)	-2.65(0.60)	-0.21(0.32)	-2.53(0.62)	-2.32(0.66)	5.11E-01	1.18E-04	8.42E-04	9.96E-01	3.71E-03	1.98E-02
Proline	0.03(0.27)	-0.01(0.12)	-2.50(0.56)	-0.04(0.30)	-2.53(0.62)	-2.49(0.57)	8.90E-01	1.29E-04	4.93E-05	9.96E-01	3.74E-03	3.10E-03
Dodecanol	-0.10(0.16)	-0.20(0.19)	-0.94(0.13)	-0.10(0.24)	-0.84(0.21)	-0.74(0.23)	6.78E-01	1.52E-04	2.27E-03	9.96E-01	4.10E-03	2.91E-02
Hypoxanthine	-0.01(0.12)	-0.08(0.37)	-1.33(0.31)	-0.07(0.39)	-1.32(0.33)	-1.25(0.48)	8.54E-01	1.97E-04	1.17E-02	9.96E-01	4.94E-03	1.03E-01
Caprylic acid	-0.06(0.17)	-0.06(0.21)	-0.90(0.17)	-0.00(0.28)	-0.85(0.24)	-0.84(0.27)	9.92E-01	8.53E-04	2.32E-03	9.96E-01	1.89E-02	2.91E-02
1-monostearin	-0.02(0.20)	0.14(0.14)	-1.00(0.21)	0.15(0.25)	-0.98(0.29)	-1.14(0.26)	5.39E-01	1.31E-03	3.86E-05	9.96E-01	2.37E-02	2.91E-03
Threonine	-0.15(0.19)	-0.14(0.18)	-0.93(0.13)	0.01(0.26)	-0.78(0.24)	-0.79(0.22)	9.74E-01	1.45E-03	6.76E-04	9.96E-01	2.37E-02	1.81E-02
Indole-3-lactate	0.05(0.18)	0.13(0.22)	-0.77(0.18)	0.09(0.28)	-0.81(0.25)	-0.90(0.28)	7.56E-01	1.94E-03	2.16E-03	9.96E-01	2.37E-02	2.91E-02
Aminomalonate	0.02(0.13)	-0.49(0.51)	-0.98(0.29)	-0.51(0.52)	-1.01(0.31)	-0.50(0.57)	3.31E-01	1.97E-03	3.82E-01	9.96E-01	2.37E-02	6.45E-01
Pelargonic acid	-0.06(0.16)	0.18(0.26)	-0.88(0.19)	0.24(0.30)	-0.82(0.25)	-1.06(0.32)	4.39E-01	2.01E-03	1.48E-03	9.96E-01	2.37E-02	2.66E-02
Pyrophosphate	0.08(0.12)	0.81(0.99)	-1.86(0.60)	0.73(1.00)	-1.94(0.62)	-2.68(1.17)	4.66E-01	2.55E-03	2.52E-02	9.96E-01	2.91E-02	1.66E-01
UDP-glucuronic acid	-0.04(0.21)	0.21(0.15)	0.84(0.19)	0.25(0.26)	0.88(0.28)	0.62(0.24)	3.32E-01	2.72E-03	1.34E-02	9.96E-01	3.01E-02	1.07E-01
Allantoin	-0.22(0.16)	-0.37(0.28)	-2.56(0.77)	-0.15(0.32)	-2.34(0.79)	-2.19(0.82)	6.48E-01	4.12E-03	9.30E-03	9.96E-01	4.39E-02	8.35E-02
3-hydroxybutyric acid	-0.04(0.18)	0.01(0.15)	1.25(0.40)	0.06(0.24)	1.29(0.44)	1.23(0.42)	8.14E-01	4.60E-03	4.65E-03	9.96E-01	4.56E-02	4.87E-02
Creatinine	-0.01(0.12)	0.03(1.00)	-1.80(0.62)	0.04(1.00)	-1.79(0.63)	-1.82(1.15)	9.71E-01	6.14E-03	1.19E-01	9.96E-01	5.65E-02	3.64E-01
Glucose	0.36(0.17)	0.57(0.16)	0.91(0.08)	0.21(0.23)	0.54(0.19)	0.34(0.18)	3.85E-01	6.42E-03	6.79E-02	9.96E-01	5.76E-02	2.78E-01

Hydrocinnamic acid	0.09(0.17)	-0.09(0.25)	-1.64(0.62)	-0.18(0.30)	-1.74(0.64)	-1.56(0.66)	5.43E-01	8.31E-03	2.12E-02	9.96E-01	6.59E-02	1.48E-01
Phenylalanine	0.01(0.25)	-0.04(0.19)	-0.74(0.13)	-0.05(0.31)	-0.75(0.28)	-0.70(0.23)	8.82E-01	1.07E-02	3.00E-03	9.96E-01	7.79E-02	3.33E-02
Isoleucine	0.03(0.20)	-0.08(0.21)	-0.67(0.16)	-0.11(0.29)	-0.70(0.27)	-0.58(0.27)	7.00E-01	1.07E-02	3.25E-02	9.96E-01	7.79E-02	1.83E-01
Cholesterol	-0.21(0.32)	-0.16(0.12)	-1.33(0.33)	0.05(0.34)	-1.11(0.45)	-1.17(0.36)	8.78E-01	1.64E-02	1.67E-03	9.96E-01	1.09E-01	2.70E-02
Tagatose	-0.07(0.12)	0.89(0.76)	2.27(0.94)	0.95(0.76)	2.34(0.95)	1.39(1.20)	2.15E-01	1.70E-02	2.53E-01	9.96E-01	1.09E-01	5.53E-01
Tyrosine	0.02(0.22)	0.16(0.20)	-0.64(0.17)	0.13(0.30)	-0.66(0.28)	-0.79(0.26)	6.56E-01	2.05E-02	3.37E-03	9.96E-01	1.29E-01	3.63E-02
UDP GlcNAc	-0.13(0.15)	0.11(0.24)	-0.70(0.22)	0.24(0.29)	-0.57(0.26)	-0.81(0.33)	4.04E-01	3.34E-02	1.67E-02	9.96E-01	2.00E-01	1.24E-01
3,6-anhydro-D-galactose	0.11(0.16)	0.43(0.27)	0.69(0.21)	0.32(0.31)	0.58(0.27)	0.26(0.34)	3.15E-01	3.47E-02	4.49E-01	9.96E-01	2.05E-01	6.93E-01
Alanine	0.12(0.24)	-0.15(0.25)	-0.46(0.15)	-0.27(0.34)	-0.58(0.28)	-0.31(0.29)	4.31E-01	4.17E-02	2.90E-01	9.96E-01	2.22E-01	5.67E-01
Shikimic acid	0.13(0.19)	0.15(0.20)	-0.50(0.23)	0.02(0.27)	-0.63(0.30)	-0.65(0.30)	9.37E-01	4.18E-02	3.24E-02	9.96E-01	2.22E-01	1.83E-01
Glutamine	0.04(0.28)	0.01(0.16)	-0.68(0.21)	-0.03(0.31)	-0.72(0.36)	-0.69(0.27)	9.15E-01	4.73E-02	1.26E-02	9.96E-01	2.43E-01	1.07E-01
Behenic acid	-0.15(0.22)	0.12(0.16)	-0.71(0.19)	0.26(0.28)	-0.56(0.29)	-0.83(0.25)	3.45E-01	5.26E-02	1.46E-03	9.96E-01	2.57E-01	2.66E-02
Kynurenine	-0.15(0.38)	0.03(0.12)	-1.15(0.35)	0.18(0.40)	-1.01(0.51)	-1.19(0.37)	6.53E-01	5.40E-02	2.22E-03	9.96E-01	2.60E-01	2.91E-02
Methionine sulfoxide	0.09(0.25)	-0.05(0.19)	-0.51(0.18)	-0.14(0.30)	-0.59(0.31)	-0.46(0.26)	6.55E-01	5.93E-02	8.47E-02	9.96E-01	2.76E-01	3.02E-01
2-hydroxypyrazinyl-2-propenoic acid ethyl ester NIST	-0.12(0.19)	-0.07(0.24)	-0.61(0.19)	0.05(0.31)	-0.50(0.27)	-0.55(0.31)	8.74E-01	6.49E-02	8.00E-02	9.96E-01	2.89E-01	2.96E-01
Glyceric acid	0.06(0.12)	-0.31(1.35)	-1.89(1.04)	-0.38(1.36)	-1.96(1.04)	-1.58(1.73)	7.83E-01	6.53E-02	3.62E-01	9.96E-01	2.89E-01	6.44E-01
Trans-4-hydroxyproline	-0.06(0.15)	-0.29(0.30)	-0.61(0.25)	-0.23(0.33)	-0.54(0.29)	-0.32(0.39)	4.93E-01	6.75E-02	4.24E-01	9.96E-01	2.96E-01	6.71E-01
Beta-alanine	0.05(0.16)	-0.05(0.31)	-0.42(0.20)	-0.10(0.35)	-0.47(0.25)	-0.37(0.37)	7.79E-01	6.93E-02	3.15E-01	9.96E-01	2.97E-01	5.88E-01
Conduritol-beta-expoxide	0.04(0.18)	-0.04(0.23)	-0.45(0.20)	-0.08(0.29)	-0.49(0.27)	-0.41(0.30)	7.89E-01	7.18E-02	1.81E-01	9.96E-01	3.04E-01	4.68E-01
Hexuronic acid	-0.12(0.23)	0.01(0.18)	0.53(0.27)	0.12(0.29)	0.64(0.36)	0.52(0.33)	6.81E-01	7.51E-02	1.18E-01	9.96E-01	3.11E-01	3.64E-01
Methionine	0.10(0.48)	0.06(0.19)	-0.80(0.18)	-0.04(0.50)	-0.91(0.51)	-0.87(0.26)	9.36E-01	8.05E-02	1.61E-03	9.96E-01	3.16E-01	2.70E-02
Oxoproline	-0.29(0.22)	-0.31(0.18)	-0.74(0.15)	-0.02(0.28)	-0.45(0.26)	-0.43(0.23)	9.54E-01	9.50E-02	6.99E-02	9.96E-01	3.43E-01	2.81E-01
Sorbitol	0.62(1.39)	0.13(0.11)	3.50(1.00)	-0.49(1.40)	2.88(1.70)	3.37(1.01)	7.26E-01	9.54E-02	1.34E-03	9.96E-01	3.43E-01	2.65E-02
3-aminoisobutyric acid	-0.04(0.29)	-0.22(0.26)	-0.59(0.16)	-0.18(0.39)	-0.55(0.33)	-0.38(0.30)	6.55E-01	9.76E-02	2.11E-01	9.96E-01	3.47E-01	5.14E-01
Nicotinic acid	0.34(0.39)	-1.24(1.59)	-0.33(0.12)	-1.58(1.64)	-0.67(0.40)	0.91(1.60)	3.39E-01	1.01E-01	5.72E-01	9.96E-01	3.48E-01	8.05E-01

Arachidic acid	0.37(0.43)	-0.03(0.22)	-0.36(0.14)	-0.41(0.48)	-0.73(0.45)	-0.32(0.26)	3.98E-01	1.07E-01	2.20E-01	9.96E-01	3.56E-01	5.16E-01
Lauric acid	0.05(0.19)	-0.20(0.18)	-0.60(0.35)	-0.25(0.27)	-0.65(0.40)	-0.41(0.39)	3.59E-01	1.08E-01	3.06E-01	9.96E-01	3.56E-01	5.85E-01
Pentadecanoic acid	0.24(0.23)	-0.21(0.26)	-0.22(0.16)	-0.45(0.35)	-0.46(0.28)	-0.01(0.31)	2.06E-01	1.09E-01	9.66E-01	9.96E-01	3.56E-01	9.93E-01
Hippuric acid	-0.01(0.14)	0.12(0.34)	0.47(0.26)	0.13(0.38)	0.49(0.30)	0.35(0.42)	7.27E-01	1.15E-01	4.00E-01	9.96E-01	3.67E-01	6.53E-01
Cysteine	-0.04(0.19)	0.20(0.29)	-0.42(0.18)	0.24(0.34)	-0.39(0.26)	-0.62(0.33)	4.84E-01	1.38E-01	6.62E-02	9.96E-01	4.20E-01	2.78E-01
2-hydroxyglutaric acid	0.10(0.17)	-0.24(0.37)	-0.27(0.19)	-0.34(0.40)	-0.37(0.25)	-0.03(0.43)	3.97E-01	1.47E-01	9.45E-01	9.96E-01	4.27E-01	9.87E-01
Dehydroabietic acid	-0.18(0.33)	-0.04(0.13)	-0.90(0.38)	0.14(0.35)	-0.73(0.50)	-0.86(0.40)	6.98E-01	1.55E-01	3.36E-02	9.96E-01	4.40E-01	1.86E-01
Lactulose	0.00(0.14)	0.40(0.31)	0.58(0.38)	0.40(0.34)	0.58(0.41)	0.18(0.49)	2.47E-01	1.56E-01	7.07E-01	9.96E-01	4.40E-01	8.98E-01
Parabanic acid NIST	-0.01(0.16)	0.15(0.22)	-0.51(0.31)	0.16(0.27)	-0.50(0.35)	-0.66(0.38)	5.49E-01	1.61E-01	9.02E-02	9.96E-01	4.40E-01	3.13E-01
N-acetylornithine	0.03(0.25)	-0.31(0.17)	0.67(0.38)	-0.34(0.30)	0.64(0.45)	0.98(0.42)	2.60E-01	1.63E-01	2.28E-02	9.96E-01	4.40E-01	1.56E-01
Butane-2,3-diol NIST	0.10(0.21)	0.42(0.29)	-0.32(0.22)	0.32(0.36)	-0.43(0.30)	-0.74(0.36)	3.85E-01	1.65E-01	4.55E-02	9.96E-01	4.40E-01	2.23E-01
Tocopherol alpha-	0.10(0.32)	-0.18(0.15)	-0.49(0.29)	-0.28(0.36)	-0.59(0.43)	-0.31(0.33)	4.37E-01	1.76E-01	3.45E-01	9.96E-01	4.56E-01	6.25E-01
Urea	-0.20(0.17)	-0.03(0.17)	-0.83(0.43)	0.17(0.24)	-0.63(0.47)	-0.80(0.47)	4.83E-01	1.78E-01	9.08E-02	9.96E-01	4.56E-01	3.13E-01
Maltotriose	0.05(0.29)	0.18(0.16)	-0.42(0.19)	0.13(0.33)	-0.47(0.35)	-0.60(0.25)	6.89E-01	1.81E-01	2.12E-02	9.96E-01	4.56E-01	1.48E-01
1-monopalmitin	-0.13(0.29)	0.04(0.13)	-0.87(0.46)	0.18(0.32)	-0.74(0.55)	-0.92(0.48)	5.83E-01	1.85E-01	6.12E-02	9.96E-01	4.56E-01	2.68E-01
2-piperidinobenzonitrile NIST	-0.13(0.21)	-0.10(0.35)	0.24(0.17)	0.03(0.41)	0.37(0.28)	0.33(0.39)	9.35E-01	1.89E-01	3.96E-01	9.96E-01	4.56E-01	6.53E-01
Hexitol	0.09(0.15)	0.12(0.23)	0.58(0.34)	0.03(0.28)	0.49(0.37)	0.45(0.41)	9.02E-01	1.96E-01	2.79E-01	9.96E-01	4.66E-01	5.61E-01
Salicylic acid	-0.09(0.13)	-0.06(0.51)	-0.60(0.37)	0.03(0.52)	-0.51(0.39)	-0.54(0.62)	9.49E-01	1.97E-01	3.88E-01	9.96E-01	4.66E-01	6.45E-01
Pseudo uridine	-0.11(0.25)	0.03(0.28)	-0.50(0.18)	0.14(0.38)	-0.39(0.30)	-0.53(0.33)	7.12E-01	2.02E-01	1.13E-01	9.96E-01	4.71E-01	3.58E-01
Xylitol	-0.75(0.41)	-0.27(0.70)	-0.22(0.13)	0.48(0.81)	0.53(0.43)	0.05(0.71)	5.56E-01	2.28E-01	9.49E-01	9.96E-01	5.17E-01	9.87E-01
Glutamic acid	0.11(0.19)	0.14(0.22)	-0.27(0.26)	0.03(0.29)	-0.38(0.33)	-0.41(0.35)	9.22E-01	2.50E-01	2.43E-01	9.96E-01	5.54E-01	5.39E-01
2,4-diaminobutyric acid	-0.05(0.18)	0.08(0.27)	0.25(0.21)	0.13(0.32)	0.30(0.26)	0.17(0.34)	6.85E-01	2.63E-01	6.31E-01	9.96E-01	5.76E-01	8.36E-01
4-hydroxyphenylacetic acid	0.07(0.41)	0.31(0.41)	-0.40(0.13)	0.24(0.58)	-0.47(0.42)	-0.71(0.43)	6.83E-01	2.68E-01	1.04E-01	9.96E-01	5.83E-01	3.37E-01
Nonadecanoic acid	0.00(0.33)	0.31(0.16)	-0.41(0.19)	0.31(0.37)	-0.40(0.38)	-0.71(0.25)	4.00E-01	2.92E-01	5.76E-03	9.96E-01	6.21E-01	5.71E-02

Mannose	-0.17(0.42)	0.30(0.28)	0.30(0.13)	0.47(0.50)	0.47(0.44)	0.00(0.31)	3.54E-01	2.93E-01	9.92E-01	9.96E-01	6.21E-01	9.98E-01
Quinic acid	-0.14(0.20)	0.04(0.34)	0.13(0.18)	0.18(0.39)	0.27(0.27)	0.09(0.39)	6.44E-01	3.09E-01	8.16E-01	9.96E-01	6.48E-01	9.41E-01
Pyrrole-2-carboxylic acid	-0.20(0.21)	-0.08(0.24)	0.09(0.20)	0.13(0.31)	0.29(0.29)	0.16(0.31)	6.85E-01	3.13E-01	6.00E-01	9.96E-01	6.51E-01	8.29E-01
2-hydroxyvaleric acid	0.00(0.22)	0.21(0.27)	-0.39(0.33)	0.21(0.35)	-0.40(0.40)	-0.61(0.42)	5.53E-01	3.28E-01	1.51E-01	9.96E-01	6.67E-01	4.24E-01
Phosphate	-0.30(0.24)	-0.10(0.24)	-0.58(0.17)	0.20(0.33)	-0.28(0.28)	-0.48(0.29)	5.50E-01	3.29E-01	9.93E-02	9.96E-01	6.67E-01	3.34E-01
Glycolic acid	-0.04(0.19)	-0.07(0.21)	0.24(0.21)	-0.03(0.29)	0.28(0.28)	0.31(0.30)	9.24E-01	3.31E-01	3.18E-01	9.96E-01	6.67E-01	5.90E-01
3-(1-pyrazolyl)-L-alanine	-0.23(0.46)	0.04(0.12)	-0.82(0.37)	0.27(0.47)	-0.59(0.60)	-0.86(0.39)	5.78E-01	3.31E-01	3.24E-02	9.96E-01	6.67E-01	1.83E-01
Adenosine-5-monophosphate	-0.03(0.35)	0.33(0.25)	-0.39(0.14)	0.35(0.42)	-0.37(0.38)	-0.72(0.28)	4.09E-01	3.35E-01	1.36E-02	9.96E-01	6.67E-01	1.07E-01
1,5-anhydroglucitol	-5.70(5.46)	-1.38(0.63)	-0.50(0.11)	4.33(5.52)	5.20(5.46)	0.88(0.64)	4.36E-01	3.44E-01	1.75E-01	9.96E-01	6.74E-01	4.63E-01
Hydroxylamine	-0.31(0.27)	-0.11(0.18)	-0.01(0.20)	0.20(0.33)	0.30(0.34)	0.11(0.26)	5.44E-01	3.68E-01	6.88E-01	9.96E-01	6.94E-01	8.82E-01
Isocitric acid	-0.21(0.25)	-0.15(0.23)	0.07(0.19)	0.06(0.34)	0.28(0.31)	0.22(0.29)	8.58E-01	3.70E-01	4.55E-01	9.96E-01	6.94E-01	6.97E-01
Pipecolinic acid	0.19(0.23)	0.11(0.23)	-0.09(0.22)	-0.07(0.32)	-0.28(0.31)	-0.21(0.32)	8.23E-01	3.79E-01	5.18E-01	9.96E-01	7.06E-01	7.60E-01
Asparagine	-0.22(0.28)	-0.14(0.22)	-0.49(0.15)	0.08(0.34)	-0.27(0.31)	-0.36(0.26)	8.10E-01	3.84E-01	1.78E-01	9.96E-01	7.06E-01	4.63E-01
Cellobiose minor	-0.16(0.60)	0.02(0.14)	-0.77(0.40)	0.18(0.61)	-0.62(0.71)	-0.80(0.43)	7.68E-01	3.85E-01	6.71E-02	9.96E-01	7.06E-01	2.78E-01
Glycerol-alpha-phosphate	0.01(0.19)	0.00(0.21)	-0.24(0.22)	-0.01(0.29)	-0.25(0.29)	-0.25(0.31)	9.86E-01	3.91E-01	4.28E-01	9.96E-01	7.08E-01	6.76E-01
Maltose	-0.24(0.82)	-0.02(0.13)	-1.22(0.81)	0.21(0.83)	-0.98(1.16)	-1.19(0.83)	8.01E-01	4.02E-01	1.54E-01	9.96E-01	7.17E-01	4.25E-01
Oleic acid	-0.04(0.21)	-0.17(0.34)	0.17(0.16)	-0.13(0.40)	0.21(0.27)	0.34(0.37)	7.39E-01	4.31E-01	3.57E-01	9.96E-01	7.49E-01	6.40E-01
Levoglucosan	-0.08(0.33)	0.10(0.18)	0.22(0.18)	0.18(0.37)	0.30(0.38)	0.12(0.26)	6.28E-01	4.31E-01	6.40E-01	9.96E-01	7.49E-01	8.36E-01
2-deoxyisotetronic acid NIST	-0.02(0.22)	-0.02(0.20)	0.22(0.21)	-0.00(0.31)	0.24(0.30)	0.24(0.29)	9.89E-01	4.38E-01	4.07E-01	9.96E-01	7.52E-01	6.59E-01
Mannitol	-0.04(0.15)	0.23(0.23)	0.35(0.48)	0.26(0.27)	0.39(0.50)	0.13(0.53)	3.39E-01	4.42E-01	8.12E-01	9.96E-01	7.52E-01	9.41E-01
Naproxen	-0.27(0.59)	0.04(0.12)	3.14(4.50)	0.31(0.60)	3.42(4.55)	3.11(4.50)	6.08E-01	4.55E-01	4.92E-01	9.96E-01	7.64E-01	7.33E-01
N-acetylglycine NIST	-0.14(0.43)	0.13(0.25)	-0.48(0.16)	0.27(0.49)	-0.35(0.47)	-0.61(0.29)	5.91E-01	4.60E-01	3.80E-02	9.96E-01	7.64E-01	2.05E-01
Linoleic acid	-0.07(0.27)	-0.22(0.31)	0.16(0.15)	-0.15(0.42)	0.22(0.31)	0.38(0.33)	7.19E-01	4.76E-01	2.66E-01	9.96E-01	7.75E-01	5.53E-01
Ornithine	-0.26(0.31)	-0.24(0.23)	-0.50(0.18)	0.01(0.37)	-0.25(0.35)	-0.26(0.29)	9.71E-01	4.81E-01	3.71E-01	9.96E-01	7.75E-01	6.45E-01
Capric acid	-0.05(0.21)	-0.06(0.25)	-0.29(0.28)	-0.01(0.33)	-0.24(0.35)	-0.23(0.37)	9.75E-01	4.93E-01	5.41E-01	9.96E-01	7.86E-01	7.79E-01
N-acetylglutamate	-0.36(0.58)	0.07(0.13)	0.10(0.33)	0.43(0.59)	0.46(0.68)	0.03(0.35)	4.67E-01	4.94E-01	9.33E-01	9.96E-01	7.86E-01	9.87E-01

Galactose	-0.02(0.33)	0.04(0.19)	0.22(0.17)	0.05(0.38)	0.24(0.37)	0.19(0.25)	8.88E-01	5.19E-01	4.63E-01	9.96E-01	8.06E-01	7.04E-01
Stearic acid	-0.07(0.25)	0.00(0.16)	-0.33(0.33)	0.08(0.30)	-0.26(0.42)	-0.34(0.37)	7.93E-01	5.36E-01	3.57E-01	9.96E-01	8.20E-01	6.40E-01
Phosphoethanolamine	-0.30(0.26)	-0.18(0.18)	-0.49(0.18)	0.12(0.31)	-0.19(0.31)	-0.31(0.25)	6.93E-01	5.48E-01	2.18E-01	9.96E-01	8.30E-01	5.16E-01
Serine	-0.66(0.25)	-0.31(0.25)	-0.82(0.16)	0.35(0.33)	-0.16(0.28)	-0.51(0.28)	3.01E-01	5.64E-01	7.48E-02	9.96E-01	8.37E-01	2.94E-01
Uridine	-0.04(0.21)	-0.18(0.20)	-0.20(0.21)	-0.14(0.29)	-0.16(0.30)	-0.02(0.29)	6.24E-01	5.88E-01	9.50E-01	9.96E-01	8.56E-01	9.87E-01
Fumaric acid	-0.05(0.14)	-0.24(0.47)	0.15(0.34)	-0.19(0.49)	0.20(0.37)	0.39(0.58)	6.92E-01	5.95E-01	4.99E-01	9.96E-01	8.57E-01	7.40E-01
Heptadecanoic acid	-0.03(0.21)	0.03(0.17)	-0.23(0.33)	0.06(0.27)	-0.20(0.39)	-0.26(0.37)	8.30E-01	6.06E-01	4.83E-01	9.96E-01	8.69E-01	7.23E-01
Deoxycholic acid	0.01(0.38)	-0.12(0.40)	0.22(0.14)	-0.13(0.55)	0.21(0.41)	0.33(0.43)	8.22E-01	6.10E-01	4.38E-01	9.96E-01	8.71E-01	6.88E-01
N-acetylmannosamine	-0.05(0.29)	0.01(0.14)	0.21(0.45)	0.06(0.33)	0.27(0.53)	0.21(0.47)	8.57E-01	6.19E-01	6.60E-01	9.96E-01	8.78E-01	8.55E-01
Cystine	-0.02(0.47)	0.06(0.14)	0.24(0.27)	0.08(0.49)	0.27(0.54)	0.19(0.30)	8.75E-01	6.22E-01	5.34E-01	9.96E-01	8.78E-01	7.75E-01
Lysine	-0.03(0.48)	0.01(0.19)	-0.28(0.19)	0.04(0.51)	-0.25(0.51)	-0.29(0.26)	9.37E-01	6.29E-01	2.75E-01	9.96E-01	8.79E-01	5.60E-01
Myo-inositol	0.00(0.36)	-0.10(0.22)	-0.19(0.17)	-0.10(0.42)	-0.18(0.39)	-0.09(0.28)	8.18E-01	6.36E-01	7.62E-01	9.96E-01	8.79E-01	9.23E-01
Galactonic acid	0.16(0.45)	-0.02(0.16)	0.37(0.18)	-0.18(0.47)	0.22(0.48)	0.39(0.24)	7.07E-01	6.55E-01	1.03E-01	9.96E-01	8.94E-01	3.37E-01
Erythritol	-0.06(0.78)	0.25(0.67)	0.29(0.12)	0.31(1.03)	0.35(0.79)	0.04(0.68)	7.66E-01	6.61E-01	9.54E-01	9.96E-01	8.94E-01	9.87E-01
Pinitol	-0.45(0.57)	-0.25(0.19)	-0.72(0.21)	0.20(0.60)	-0.26(0.60)	-0.46(0.28)	7.38E-01	6.66E-01	1.07E-01	9.96E-01	8.94E-01	3.45E-01
Sucrose	-0.11(0.21)	-0.08(0.15)	-0.43(0.72)	0.03(0.26)	-0.32(0.75)	-0.35(0.74)	9.22E-01	6.68E-01	6.38E-01	9.96E-01	8.94E-01	8.36E-01
Myristic acid	-0.09(0.19)	-0.08(0.19)	-0.23(0.25)	0.02(0.27)	-0.13(0.31)	-0.15(0.32)	9.45E-01	6.72E-01	6.32E-01	9.96E-01	8.94E-01	8.36E-01
Aconitic acid	0.10(0.39)	-0.43(0.34)	0.28(0.14)	-0.53(0.52)	0.17(0.41)	0.71(0.37)	3.09E-01	6.74E-01	5.88E-02	9.96E-01	8.94E-01	2.63E-01
2-hydroxybutanoic acid	0.35(0.43)	0.22(0.29)	0.16(0.14)	-0.13(0.54)	-0.19(0.45)	-0.05(0.32)	8.02E-01	6.77E-01	8.67E-01	9.96E-01	8.94E-01	9.61E-01
Acetaminophen	-0.28(0.51)	0.32(0.48)	-0.06(0.13)	0.61(0.71)	0.22(0.53)	-0.39(0.50)	3.94E-01	6.77E-01	4.42E-01	9.96E-01	8.94E-01	6.89E-01
Phenylethylamine	0.11(0.27)	0.01(0.24)	0.24(0.17)	-0.10(0.36)	0.13(0.31)	0.23(0.29)	7.87E-01	6.80E-01	4.42E-01	9.96E-01	8.94E-01	6.89E-01
Citric acid	-0.37(0.29)	-0.16(0.24)	-0.23(0.19)	0.21(0.38)	0.14(0.35)	-0.07(0.30)	5.84E-01	6.95E-01	8.08E-01	9.96E-01	9.02E-01	9.41E-01
Palmitoleic acid	0.00(0.17)	-0.01(0.36)	0.10(0.19)	-0.01(0.40)	0.10(0.26)	0.11(0.40)	9.87E-01	6.98E-01	7.93E-01	9.96E-01	9.02E-01	9.37E-01
Arachidonic acid	-0.17(0.42)	0.43(0.47)	0.00(0.13)	0.60(0.62)	0.17(0.45)	-0.43(0.49)	3.36E-01	7.02E-01	3.81E-01	9.96E-01	9.02E-01	6.45E-01
2,3-dihydroxybutanoic acid NIST	-0.16(0.23)	-0.02(0.24)	-0.27(0.18)	0.15(0.32)	-0.11(0.28)	-0.25(0.29)	6.49E-01	7.10E-01	3.82E-01	9.96E-01	9.02E-01	6.45E-01
Aspartic acid	-0.21(0.23)	-0.01(0.28)	-0.31(0.18)	0.19(0.36)	-0.10(0.29)	-0.30(0.34)	5.87E-01	7.23E-01	3.80E-01	9.96E-01	9.02E-01	6.45E-01

Palmitic acid	-0.12(0.22)	0.01(0.18)	0.00(0.27)	0.14(0.28)	0.12(0.35)	-0.02(0.33)	6.27E-01	7.31E-01	9.58E-01	9.96E-01	9.02E-01	9.87E-01
Indole-3-acetate	-0.07(0.47)	-0.24(0.34)	0.09(0.15)	-0.16(0.59)	0.17(0.49)	0.33(0.38)	7.81E-01	7.37E-01	3.85E-01	9.96E-01	9.02E-01	6.45E-01
Ketohexose	0.03(0.15)	-0.05(0.24)	0.16(0.35)	-0.08(0.28)	0.13(0.38)	0.20(0.42)	7.87E-01	7.38E-01	6.26E-01	9.96E-01	9.02E-01	8.36E-01
Isothreonic acid	0.08(0.21)	-0.29(0.28)	0.17(0.18)	-0.36(0.34)	0.09(0.28)	0.45(0.34)	2.95E-01	7.48E-01	1.86E-01	9.96E-01	9.02E-01	4.78E-01
Isothreitol	-0.14(0.28)	-0.80(0.50)	-0.24(0.14)	-0.66(0.57)	-0.10(0.31)	0.57(0.52)	2.45E-01	7.54E-01	2.82E-01	9.96E-01	9.02E-01	5.61E-01
Allantoic acid	-0.10(0.50)	0.22(0.20)	-0.27(0.24)	0.31(0.54)	-0.17(0.55)	-0.49(0.31)	5.63E-01	7.57E-01	1.17E-01	9.96E-01	9.02E-01	3.64E-01
Tryptophan	0.06(1.65)	0.02(0.12)	-0.57(1.17)	-0.04(1.65)	-0.63(2.04)	-0.59(1.18)	9.82E-01	7.59E-01	6.18E-01	9.96E-01	9.02E-01	8.36E-01
Cysteine-glycine	-0.10(0.38)	-0.08(0.19)	0.02(0.19)	0.02(0.42)	0.12(0.42)	0.10(0.27)	9.66E-01	7.69E-01	6.99E-01	9.96E-01	9.02E-01	8.93E-01
Ethanolamine	-0.15(0.39)	-0.42(0.41)	-0.04(0.14)	-0.27(0.56)	0.11(0.42)	0.38(0.43)	6.32E-01	7.89E-01	3.83E-01	9.96E-01	9.09E-01	6.45E-01
Fucose	0.33(0.86)	0.02(0.13)	0.09(0.31)	-0.31(0.88)	-0.24(0.92)	0.07(0.34)	7.22E-01	7.91E-01	8.40E-01	9.96E-01	9.09E-01	9.51E-01
Lyxitol	-0.06(0.15)	0.02(0.97)	0.02(0.25)	0.07(0.99)	0.07(0.28)	0.00(1.00)	9.42E-01	7.92E-01	9.98E-01	9.96E-01	9.09E-01	9.98E-01
Uric acid	0.14(0.43)	0.09(0.24)	0.26(0.16)	-0.04(0.50)	0.12(0.46)	0.16(0.29)	9.33E-01	7.93E-01	5.76E-01	9.96E-01	9.09E-01	8.07E-01
Oxamic acid	-0.02(0.12)	1.60(3.66)	-1.01(3.80)	1.62(3.66)	-0.98(3.80)	-2.60(5.41)	6.61E-01	7.96E-01	6.32E-01	9.96E-01	9.10E-01	8.36E-01
Tocopherol gamma-	-0.12(0.27)	-0.18(0.15)	-0.29(0.64)	-0.06(0.31)	-0.17(0.70)	-0.11(0.65)	8.52E-01	8.06E-01	8.62E-01	9.96E-01	9.12E-01	9.61E-01
Malic acid	-0.31(0.23)	0.11(0.19)	-0.38(0.20)	0.42(0.31)	-0.07(0.30)	-0.49(0.29)	1.76E-01	8.10E-01	9.20E-02	9.96E-01	9.14E-01	3.13E-01
2-deoxytetronic acid	-0.23(0.30)	-0.25(0.25)	-0.31(0.15)	-0.01(0.39)	-0.08(0.34)	-0.06(0.29)	9.72E-01	8.18E-01	8.25E-01	9.96E-01	9.15E-01	9.49E-01
Arabitol	-0.22(0.22)	0.18(0.31)	-0.17(0.16)	0.40(0.39)	0.05(0.28)	-0.35(0.34)	3.10E-01	8.56E-01	3.15E-01	9.96E-01	9.43E-01	5.88E-01
Methanolphosphate	-0.07(0.33)	-0.02(0.24)	-0.13(0.15)	0.04(0.40)	-0.06(0.36)	-0.10(0.29)	9.19E-01	8.69E-01	7.24E-01	9.96E-01	9.46E-01	9.01E-01
N-methylalanine	-0.10(0.37)	0.11(0.22)	-0.04(0.16)	0.21(0.44)	0.07(0.41)	-0.14(0.27)	6.32E-01	8.70E-01	5.99E-01	9.96E-01	9.46E-01	8.29E-01
Hydroxyproline dipeptide NIST	-0.47(0.73)	-0.52(0.46)	-0.37(0.13)	-0.05(0.87)	0.10(0.74)	0.16(0.49)	9.50E-01	8.89E-01	7.45E-01	9.96E-01	9.53E-01	9.10E-01
Glycerol	0.03(0.21)	-0.07(0.21)	0.07(0.20)	-0.10(0.30)	0.03(0.29)	0.14(0.29)	7.27E-01	9.11E-01	6.37E-01	9.96E-01	9.73E-01	8.36E-01
Ribonic acid	0.00(0.20)	-0.10(0.33)	0.03(0.30)	-0.10(0.38)	0.03(0.36)	0.13(0.44)	7.92E-01	9.37E-01	7.67E-01	9.96E-01	9.80E-01	9.23E-01
Alloxanoic acid NIST	-0.13(0.15)	-0.06(0.37)	-0.16(0.50)	0.07(0.40)	-0.04(0.51)	-0.11(0.62)	8.61E-01	9.44E-01	8.65E-01	9.96E-01	9.83E-01	9.61E-01
Succinic acid	-0.11(0.26)	-0.04(0.26)	-0.09(0.22)	0.07(0.37)	0.02(0.34)	-0.05(0.34)	8.50E-01	9.51E-01	8.86E-01	9.96E-01	9.86E-01	9.68E-01
Oxalic acid	-0.14(0.22)	0.18(0.20)	-0.15(0.21)	0.32(0.30)	-0.01(0.30)	-0.33(0.29)	2.95E-01	9.76E-01	2.68E-01	9.96E-01	9.99E-01	5.56E-01
Pyruvic acid	-0.10(0.28)	-0.06(0.19)	-0.10(0.18)	0.04(0.34)	-0.01(0.33)	-0.05(0.26)	9.10E-01	9.80E-01	8.60E-01	9.96E-01	9.99E-01	9.61E-01
Lactic acid	0.00(0.16)	-0.22(0.33)	-0.01(0.28)	-0.22(0.37)	-0.01(0.32)	0.21(0.44)	5.56E-01	9.85E-01	6.27E-01	9.96E-01	1.00E+0	8.36E-01
Threonic acid	-0.09(0.26)	-0.05(0.20)	-0.09(0.21)	0.04(0.34)	0.00(0.32)	-0.04(0.29)	9.11E-01	9.92E-01	8.90E-01	9.96E-01	1.00E+0	9.69E-01

Histidine	-0.21(0.95)	0.05(0.17)	-0.20(0.21)	0.26(0.96)	0.01(0.96)	-0.25(0.27)	7.89E-01	9.96E-01	3.58E-01	9.96E-01	1.00E+0	6.40E-01
Taurine	0.02(0.86)	-0.54(0.70)	0.03(0.13)	-0.56(1.09)	0.00(0.86)	0.56(0.70)	6.10E-01	9.98E-01	4.23E-01	9.96E-01	1.00E+0	6.71E-01
**Unknown**												
135869	0.11(0.10)	0.21(0.14)	2.07(0.15)	0.10(0.17)	1.96(0.19)	1.86(0.21)	5.44E-01	1.56E-15	1.36E-12	9.96E-01	5.86E-13	5.14E-10
7403	-0.05(0.27)	-0.14(0.25)	-5.80(1.18)	-0.09(0.37)	-5.76(1.24)	-5.66(1.20)	7.99E-01	1.67E-05	1.35E-05	9.96E-01	1.57E-03	1.27E-03
8270	0.06(0.14)	0.16(0.17)	1.30(0.24)	0.10(0.22)	1.24(0.28)	1.14(0.30)	6.39E-01	3.16E-05	3.71E-04	9.96E-01	1.98E-03	1.17E-02
21666	0.13(0.12)	0.10(0.28)	-1.38(0.33)	-0.03(0.31)	-1.51(0.35)	-1.48(0.43)	9.29E-01	6.27E-05	1.10E-03	9.96E-01	2.67E-03	2.30E-02
95550	-0.06(0.16)	0.09(0.20)	-0.98(0.14)	0.15(0.25)	-0.91(0.21)	-1.07(0.25)	5.52E-01	6.37E-05	6.80E-05	9.96E-01	2.67E-03	3.21E-03
41683	0.13(0.15)	0.85(0.33)	0.95(0.15)	0.72(0.36)	0.81(0.21)	0.10(0.37)	5.00E-02	2.11E-04	7.90E-01	9.96E-01	4.97E-03	9.36E-01
1148	0.05(0.16)	0.16(0.16)	-1.99(0.57)	0.10(0.23)	-2.04(0.59)	-2.15(0.59)	6.47E-01	9.95E-04	5.19E-04	9.96E-01	2.08E-02	1.50E-02
64546	0.43(0.14)	0.54(0.15)	0.96(0.07)	0.11(0.20)	0.53(0.16)	0.42(0.16)	5.87E-01	1.37E-03	1.29E-02	9.96E-01	2.37E-02	1.07E-01
7440	-0.24(0.14)	-0.52(0.30)	-7.47(2.15)	-0.28(0.32)	-7.24(2.16)	-6.95(2.18)	3.78E-01	1.37E-03	2.18E-03	9.96E-01	2.37E-02	2.91E-02
6646	0.21(0.20)	0.52(0.17)	0.92(0.08)	0.30(0.27)	0.71(0.21)	0.40(0.19)	2.64E-01	1.62E-03	4.19E-02	9.96E-01	2.37E-02	2.14E-01
915	-0.07(0.17)	-0.20(0.48)	-7.38(2.23)	-0.13(0.51)	-7.31(2.23)	-7.18(2.28)	8.00E-01	1.67E-03	2.44E-03	9.96E-01	2.37E-02	2.96E-02
9320	0.12(0.12)	-0.63(0.34)	-0.92(0.29)	-0.75(0.37)	-1.04(0.32)	-0.29(0.45)	4.38E-02	1.72E-03	5.21E-01	9.96E-01	2.37E-02	7.62E-01
5419	-0.08(0.18)	-0.02(0.21)	-1.33(0.34)	0.06(0.28)	-1.25(0.38)	-1.31(0.40)	8.16E-01	1.81E-03	1.92E-03	9.96E-01	2.37E-02	2.89E-02
4546	0.30(0.16)	0.58(0.27)	1.58(0.36)	0.28(0.31)	1.27(0.39)	0.99(0.45)	3.75E-01	1.89E-03	2.90E-02	9.96E-01	2.37E-02	1.80E-01
1941	-0.18(0.18)	0.10(0.20)	-0.91(0.14)	0.29(0.27)	-0.73(0.23)	-1.02(0.24)	2.88E-01	1.97E-03	9.92E-05	9.96E-01	2.37E-02	4.16E-03
1029	-0.21(0.18)	0.01(0.20)	-0.98(0.17)	0.21(0.25)	-0.77(0.24)	-0.99(0.25)	4.02E-01	1.99E-03	2.12E-04	9.96E-01	2.37E-02	7.99E-03
257	0.04(0.14)	-0.27(0.38)	-0.75(0.23)	-0.31(0.40)	-0.79(0.27)	-0.48(0.45)	4.47E-01	4.19E-03	2.85E-01	9.96E-01	4.39E-02	5.61E-01
4943	-0.09(0.20)	0.25(0.22)	0.65(0.15)	0.35(0.29)	0.74(0.25)	0.39(0.26)	2.44E-01	4.52E-03	1.34E-01	9.96E-01	4.56E-02	3.95E-01
132379	-0.07(0.23)	-0.07(0.20)	-0.87(0.16)	-0.00(0.30)	-0.80(0.28)	-0.80(0.25)	9.91E-01	5.64E-03	2.54E-03	9.96E-01	5.46E-02	2.99E-02
1700	0.01(0.16)	-0.18(0.23)	-0.92(0.28)	-0.19(0.28)	-0.93(0.33)	-0.74(0.37)	4.96E-01	5.91E-03	4.99E-02	9.96E-01	5.57E-02	2.36E-01
20903	-0.06(0.19)	0.14(0.20)	-0.87(0.23)	0.20(0.27)	-0.81(0.29)	-1.01(0.29)	4.64E-01	7.21E-03	9.62E-04	9.96E-01	6.23E-02	2.13E-02
16550	-0.18(0.17)	-0.39(0.23)	-0.81(0.15)	-0.21(0.29)	-0.63(0.23)	-0.42(0.27)	4.73E-01	7.27E-03	1.31E-01	9.96E-01	6.23E-02	3.89E-01
4985	0.07(0.13)	-1.88(1.83)	-13.52(4.94)	-1.96(1.84)	-13.59(4.95)	-11.63(5.25)	2.90E-01	7.77E-03	3.04E-02	9.96E-01	6.51E-02	1.83E-01
106629	0.04(0.14)	-0.74(0.90)	-0.84(0.29)	-0.79(0.92)	-0.88(0.32)	-0.10(0.93)	3.94E-01	8.34E-03	9.17E-01	9.96E-01	6.59E-02	9.82E-01
5346	-0.03(0.13)	-0.23(0.35)	-0.85(0.27)	-0.21(0.37)	-0.82(0.30)	-0.62(0.44)	5.79E-01	8.39E-03	1.66E-01	9.96E-01	6.59E-02	4.46E-01
4887	0.06(0.21)	-0.12(0.15)	-1.47(0.53)	-0.18(0.26)	-1.53(0.57)	-1.35(0.55)	4.95E-01	9.50E-03	1.62E-02	9.96E-01	7.31E-02	1.24E-01
135260	-0.08(0.15)	-0.47(0.23)	-0.79(0.22)	-0.39(0.28)	-0.72(0.27)	-0.33(0.32)	1.63E-01	9.77E-03	3.14E-01	9.96E-01	7.37E-02	5.88E-01

1912	0.02(0.26)	0.05(0.13)	-1.01(0.32)	0.03(0.29)	-1.03(0.41)	-1.07(0.35)	9.04E-01	1.36E-02	2.98E-03	9.96E-01	9.66E-02	3.33E-02
6353	-0.09(0.14)	0.04(0.36)	-0.97(0.32)	0.13(0.39)	-0.88(0.35)	-1.01(0.48)	7.37E-01	1.40E-02	3.81E-02	9.96E-01	9.76E-02	2.05E-01
4929	0.01(0.19)	-0.12(0.16)	-1.45(0.56)	-0.14(0.25)	-1.46(0.59)	-1.32(0.57)	5.72E-01	1.58E-02	2.35E-02	9.96E-01	1.08E-01	1.58E-01
41682	-0.14(0.16)	0.10(0.20)	0.72(0.31)	0.25(0.25)	0.87(0.35)	0.62(0.37)	3.36E-01	1.66E-02	1.01E-01	9.96E-01	1.09E-01	3.37E-01
5483	0.05(0.20)	-0.10(0.19)	-0.65(0.21)	-0.15(0.27)	-0.70(0.29)	-0.55(0.29)	5.93E-01	1.68E-02	5.77E-02	9.96E-01	1.09E-01	2.62E-01
5900	-0.08(0.16)	-0.03(0.23)	-0.74(0.23)	0.06(0.28)	-0.65(0.28)	-0.71(0.32)	8.44E-01	2.13E-02	3.12E-02	9.96E-01	1.32E-01	1.83E-01
131620	-0.29(0.22)	0.12(0.21)	-0.83(0.12)	0.40(0.30)	-0.54(0.25)	-0.94(0.24)	1.82E-01	3.26E-02	2.47E-04	9.96E-01	1.98E-01	8.45E-03
62433	-0.10(0.17)	-0.28(0.23)	-0.66(0.20)	-0.19(0.28)	-0.56(0.26)	-0.38(0.29)	5.10E-01	3.58E-02	1.97E-01	9.96E-01	2.08E-01	4.96E-01
17537	0.03(0.14)	-0.03(0.43)	-0.67(0.29)	-0.06(0.44)	-0.70(0.33)	-0.64(0.52)	8.89E-01	3.71E-02	2.27E-01	9.96E-01	2.12E-01	5.16E-01
473	-0.13(0.15)	0.23(0.78)	0.46(0.26)	0.37(0.80)	0.59(0.28)	0.22(0.82)	6.45E-01	3.82E-02	7.87E-01	9.96E-01	2.15E-01	9.36E-01
2061	-0.16(0.18)	-0.09(0.25)	-0.71(0.18)	0.08(0.30)	-0.54(0.26)	-0.62(0.31)	7.98E-01	3.97E-02	4.64E-02	9.96E-01	2.19E-01	2.24E-01
4945	-0.05(0.14)	-0.07(0.32)	-0.76(0.31)	-0.02(0.35)	-0.71(0.34)	-0.69(0.44)	9.54E-01	4.00E-02	1.24E-01	9.96E-01	2.19E-01	3.75E-01
132414	0.04(0.72)	-0.06(0.12)	2.25(0.79)	-0.10(0.72)	2.21(1.07)	2.31(0.80)	8.91E-01	4.31E-02	5.45E-03	9.96E-01	2.26E-01	5.56E-02
132976	0.24(0.23)	0.05(0.29)	-0.31(0.15)	-0.19(0.37)	-0.55(0.27)	-0.36(0.33)	6.05E-01	4.77E-02	2.74E-01	9.96E-01	2.43E-01	5.60E-01
2001	0.11(0.13)	0.35(0.70)	-0.87(0.47)	0.24(0.71)	-0.97(0.49)	-1.22(0.85)	7.33E-01	5.04E-02	1.56E-01	9.96E-01	2.53E-01	4.25E-01
117171	-0.08(0.20)	0.21(0.23)	-0.59(0.17)	0.29(0.31)	-0.51(0.26)	-0.80(0.29)	3.52E-01	5.23E-02	7.09E-03	9.96E-01	2.57E-01	6.52E-02
91	-0.08(0.21)	-0.04(0.17)	-0.70(0.24)	0.03(0.27)	-0.62(0.32)	-0.66(0.29)	9.01E-01	5.46E-02	2.76E-02	9.96E-01	2.60E-01	1.80E-01
41689	-0.16(0.28)	0.02(0.23)	0.45(0.17)	0.18(0.36)	0.61(0.32)	0.43(0.28)	6.20E-01	5.85E-02	1.25E-01	9.96E-01	2.76E-01	3.75E-01
453	0.22(0.79)	-0.02(0.12)	-1.77(0.68)	-0.24(0.80)	-1.99(1.05)	-1.75(0.69)	7.67E-01	6.24E-02	1.33E-02	9.96E-01	2.87E-01	1.07E-01
54	0.06(0.21)	0.00(0.19)	-0.49(0.20)	-0.06(0.28)	-0.55(0.29)	-0.49(0.27)	8.20E-01	6.42E-02	7.97E-02	9.96E-01	2.89E-01	2.96E-01
17664	-0.05(0.37)	0.02(0.14)	0.88(0.32)	0.07(0.40)	0.93(0.50)	0.86(0.35)	8.55E-01	6.84E-02	1.66E-02	9.96E-01	2.96E-01	1.24E-01
87756	-0.12(0.20)	-0.13(0.26)	-0.59(0.16)	-0.01(0.33)	-0.47(0.26)	-0.46(0.31)	9.77E-01	7.26E-02	1.39E-01	9.96E-01	3.04E-01	4.04E-01
657	0.00(0.33)	0.14(0.15)	0.70(0.21)	0.14(0.36)	0.70(0.39)	0.56(0.25)	6.97E-01	7.73E-02	2.90E-02	9.96E-01	3.16E-01	1.80E-01
139467	-0.14(0.17)	0.46(0.21)	1.03(0.65)	0.59(0.26)	1.17(0.66)	0.58(0.68)	2.64E-02	7.99E-02	3.98E-01	9.96E-01	3.16E-01	6.53E-01
159824	0.08(0.19)	0.01(0.18)	-0.52(0.27)	-0.07(0.25)	-0.60(0.34)	-0.53(0.34)	7.82E-01	8.01E-02	1.24E-01	9.96E-01	3.16E-01	3.75E-01
17651	0.00(0.19)	-0.17(0.26)	0.44(0.17)	-0.17(0.34)	0.44(0.25)	0.61(0.32)	6.31E-01	8.31E-02	6.21E-02	9.96E-01	3.23E-01	2.69E-01
4927	-0.16(0.14)	0.09(0.28)	-0.82(0.35)	0.25(0.32)	-0.66(0.38)	-0.91(0.46)	4.43E-01	8.63E-02	5.51E-02	9.96E-01	3.32E-01	2.57E-01
106634	-0.12(0.17)	-0.15(0.27)	0.35(0.21)	-0.03(0.32)	0.47(0.27)	0.50(0.34)	9.22E-01	8.79E-02	1.50E-01	9.96E-01	3.35E-01	4.24E-01
126397	-0.03(0.21)	-0.07(0.28)	-0.50(0.17)	-0.04(0.33)	-0.47(0.27)	-0.43(0.32)	9.04E-01	8.93E-02	1.90E-01	9.96E-01	3.37E-01	4.80E-01
592	0.07(0.18)	-0.25(0.41)	-0.38(0.20)	-0.32(0.45)	-0.45(0.27)	-0.14(0.46)	4.83E-01	9.43E-02	7.69E-01	9.96E-01	3.43E-01	9.23E-01

135610	0.07(0.24)	0.67(0.42)	0.51(0.13)	0.60(0.49)	0.44(0.26)	-0.16(0.44)	2.20E-01	9.50E-02	7.12E-01	9.96E-01	3.43E-01	8.98E-01
16792	0.11(0.23)	0.32(0.18)	0.61(0.19)	0.21(0.30)	0.50(0.30)	0.30(0.26)	4.90E-01	9.54E-02	2.63E-01	9.96E-01	3.43E-01	5.53E-01
1725	0.00(0.16)	-0.01(0.24)	-0.56(0.30)	-0.01(0.28)	-0.56(0.34)	-0.55(0.38)	9.70E-01	9.90E-02	1.52E-01	9.96E-01	3.48E-01	4.24E-01
4981	-0.08(0.16)	0.02(0.26)	0.61(0.38)	0.10(0.30)	0.69(0.41)	0.59(0.46)	7.30E-01	1.00E-01	2.07E-01	9.96E-01	3.48E-01	5.14E-01
3329	-0.10(0.24)	-0.10(0.20)	-0.64(0.22)	-0.00(0.31)	-0.54(0.32)	-0.54(0.30)	9.96E-01	1.03E-01	7.65E-02	9.96E-01	3.53E-01	2.96E-01
6526	-0.07(0.16)	0.21(0.26)	0.43(0.27)	0.29(0.31)	0.50(0.31)	0.22(0.38)	3.53E-01	1.07E-01	5.69E-01	9.96E-01	3.56E-01	8.05E-01
6538	0.08(0.29)	0.08(0.18)	-0.52(0.23)	-0.00(0.34)	-0.60(0.37)	-0.59(0.28)	9.92E-01	1.08E-01	4.10E-02	9.96E-01	3.56E-01	2.12E-01
26916	0.01(0.26)	-0.07(0.18)	-0.52(0.20)	-0.08(0.33)	-0.54(0.34)	-0.46(0.27)	8.07E-01	1.15E-01	9.16E-02	9.96E-01	3.67E-01	3.13E-01
41708	-0.20(0.23)	0.18(0.22)	0.26(0.17)	0.38(0.32)	0.46(0.29)	0.08(0.28)	2.34E-01	1.16E-01	7.76E-01	9.96E-01	3.67E-01	9.28E-01
121890	-0.04(0.17)	0.18(0.20)	-0.59(0.31)	0.23(0.26)	-0.55(0.34)	-0.77(0.37)	3.94E-01	1.16E-01	4.07E-02	9.96E-01	3.67E-01	2.12E-01
6115	-0.16(0.54)	0.15(0.14)	0.77(0.25)	0.31(0.56)	0.93(0.61)	0.63(0.28)	5.86E-01	1.29E-01	2.92E-02	9.96E-01	4.04E-01	1.80E-01
32247	0.03(0.22)	-0.07(0.28)	-0.39(0.17)	-0.10(0.36)	-0.42(0.27)	-0.32(0.32)	7.87E-01	1.30E-01	3.22E-01	9.96E-01	4.05E-01	5.90E-01
119167	0.18(0.20)	0.35(0.21)	0.58(0.16)	0.17(0.29)	0.40(0.26)	0.23(0.27)	5.69E-01	1.33E-01	4.01E-01	9.96E-01	4.12E-01	6.53E-01
121002	0.02(0.26)	-0.01(0.24)	-0.44(0.16)	-0.03(0.35)	-0.46(0.30)	-0.42(0.29)	9.22E-01	1.38E-01	1.54E-01	9.96E-01	4.20E-01	4.25E-01
6802	0.02(0.26)	0.06(0.29)	0.48(0.14)	0.03(0.39)	0.45(0.30)	0.42(0.33)	9.31E-01	1.39E-01	2.12E-01	9.96E-01	4.20E-01	5.14E-01
2806	0.02(0.15)	-0.78(0.94)	-1.01(0.68)	-0.80(0.95)	-1.03(0.69)	-0.23(1.18)	4.01E-01	1.42E-01	8.48E-01	9.96E-01	4.24E-01	9.54E-01
13107	-0.12(0.29)	-0.33(0.16)	-0.67(0.22)	-0.21(0.33)	-0.55(0.37)	-0.34(0.27)	5.28E-01	1.44E-01	2.20E-01	9.96E-01	4.27E-01	5.16E-01
3029	-0.06(0.24)	-0.07(0.35)	0.36(0.15)	-0.01(0.42)	0.42(0.28)	0.43(0.38)	9.78E-01	1.45E-01	2.60E-01	9.96E-01	4.27E-01	5.53E-01
134336	-0.20(0.18)	-0.21(0.31)	-0.55(0.16)	-0.01(0.36)	-0.35(0.24)	-0.34(0.35)	9.83E-01	1.47E-01	3.23E-01	9.96E-01	4.27E-01	5.90E-01
892	-0.49(0.75)	-0.07(0.12)	0.77(0.47)	0.42(0.76)	1.26(0.87)	0.84(0.48)	5.81E-01	1.49E-01	8.45E-02	9.96E-01	4.30E-01	3.02E-01
135619	0.05(0.14)	-0.09(0.31)	0.64(0.39)	-0.14(0.34)	0.60(0.42)	0.74(0.50)	6.78E-01	1.58E-01	1.45E-01	9.96E-01	4.40E-01	4.16E-01
62	-0.14(0.25)	-0.04(0.25)	-0.56(0.14)	0.11(0.36)	-0.42(0.29)	-0.53(0.28)	7.66E-01	1.59E-01	6.64E-02	9.96E-01	4.40E-01	2.78E-01
5990	0.02(0.22)	-0.06(0.24)	-0.38(0.17)	-0.08(0.33)	-0.40(0.28)	-0.32(0.30)	8.06E-01	1.60E-01	2.86E-01	9.96E-01	4.40E-01	5.61E-01
41697	-0.16(0.20)	0.08(0.20)	0.25(0.22)	0.24(0.29)	0.42(0.29)	0.17(0.30)	3.98E-01	1.60E-01	5.68E-01	9.96E-01	4.40E-01	8.05E-01
1875	-0.13(0.20)	-0.35(0.15)	-0.61(0.28)	-0.21(0.25)	-0.48(0.34)	-0.27(0.32)	3.97E-01	1.64E-01	4.02E-01	9.96E-01	4.40E-01	6.53E-01
133943	-0.15(0.22)	0.04(0.16)	-0.68(0.31)	0.20(0.27)	-0.53(0.38)	-0.73(0.35)	4.69E-01	1.66E-01	4.38E-02	9.96E-01	4.40E-01	2.20E-01
7402	0.06(0.20)	-0.15(0.24)	-1.21(0.91)	-0.21(0.30)	-1.28(0.91)	-1.07(0.93)	4.87E-01	1.67E-01	2.56E-01	9.96E-01	4.41E-01	5.53E-01
160543	-0.15(0.25)	0.02(0.15)	-0.68(0.29)	0.17(0.29)	-0.53(0.38)	-0.70(0.33)	5.71E-01	1.71E-01	3.94E-02	9.96E-01	4.48E-01	2.09E-01
46292	-0.24(0.23)	-0.18(0.20)	-0.65(0.17)	0.07(0.31)	-0.40(0.29)	-0.47(0.26)	8.27E-01	1.75E-01	7.88E-02	9.96E-01	4.56E-01	2.96E-01
4543	-0.06(0.15)	0.04(0.20)	-0.72(0.47)	0.10(0.25)	-0.67(0.49)	-0.76(0.51)	7.03E-01	1.80E-01	1.37E-01	9.96E-01	4.56E-01	4.01E-01

88583	-0.05(0.19)	-0.05(0.24)	-0.42(0.20)	-0.00(0.30)	-0.37(0.27)	-0.37(0.30)	9.90E-01	1.81E-01	2.29E-01	9.96E-01	4.56E-01	5.16E-01
3465	-0.11(0.19)	0.16(0.28)	0.28(0.22)	0.26(0.34)	0.38(0.28)	0.12(0.35)	4.39E-01	1.84E-01	7.40E-01	9.96E-01	4.56E-01	9.10E-01
4942	0.11(0.45)	0.03(0.13)	0.95(0.44)	-0.07(0.47)	0.84(0.63)	0.92(0.46)	8.74E-01	1.84E-01	5.02E-02	9.96E-01	4.56E-01	2.36E-01
87769	-0.07(0.14)	-0.08(0.43)	-0.48(0.28)	-0.02(0.45)	-0.42(0.31)	-0.40(0.53)	9.70E-01	1.90E-01	4.50E-01	9.96E-01	4.56E-01	6.93E-01
39	-0.42(0.27)	-0.16(0.36)	-0.02(0.16)	0.26(0.45)	0.40(0.31)	0.15(0.40)	5.67E-01	1.90E-01	7.15E-01	9.96E-01	4.56E-01	8.98E-01
68	-0.11(0.20)	0.03(0.25)	-0.45(0.17)	0.14(0.33)	-0.34(0.26)	-0.48(0.30)	6.63E-01	1.98E-01	1.14E-01	9.96E-01	4.66E-01	3.58E-01
6066	-0.23(0.29)	0.03(0.19)	-0.64(0.14)	0.26(0.34)	-0.41(0.32)	-0.67(0.23)	4.51E-01	1.99E-01	6.00E-03	9.96E-01	4.66E-01	5.80E-02
87720	-0.12(0.20)	-0.17(0.19)	-0.49(0.22)	-0.05(0.28)	-0.37(0.30)	-0.32(0.29)	8.55E-01	2.22E-01	2.72E-01	9.96E-01	5.10E-01	5.60E-01
1872	-0.14(0.21)	-0.20(0.29)	0.19(0.18)	-0.06(0.35)	0.33(0.27)	0.40(0.35)	8.61E-01	2.25E-01	2.55E-01	9.96E-01	5.13E-01	5.53E-01
41808	-0.13(0.18)	0.03(0.28)	-0.49(0.24)	0.16(0.33)	-0.36(0.31)	-0.52(0.37)	6.24E-01	2.42E-01	1.67E-01	9.96E-01	5.47E-01	4.46E-01
1981	-0.07(0.17)	-0.07(0.37)	-0.36(0.18)	-0.00(0.41)	-0.29(0.25)	-0.29(0.41)	9.95E-01	2.47E-01	4.78E-01	9.96E-01	5.54E-01	7.21E-01
127277	0.09(0.22)	0.40(0.28)	-0.26(0.21)	0.31(0.36)	-0.36(0.31)	-0.67(0.35)	3.88E-01	2.49E-01	5.93E-02	9.96E-01	5.54E-01	2.63E-01
3232	-0.05(0.60)	0.04(0.13)	-0.83(0.34)	0.09(0.61)	-0.78(0.70)	-0.86(0.37)	8.89E-01	2.69E-01	2.12E-02	9.96E-01	5.83E-01	1.48E-01
1709	-0.17(0.29)	0.43(0.22)	-0.51(0.14)	0.60(0.37)	-0.34(0.31)	-0.94(0.26)	1.06E-01	2.81E-01	7.20E-04	9.96E-01	6.05E-01	1.81E-02
42357	0.03(0.15)	0.13(0.26)	-0.28(0.25)	0.10(0.30)	-0.31(0.30)	-0.42(0.37)	7.37E-01	2.93E-01	2.59E-01	9.96E-01	6.21E-01	5.53E-01
134	-0.32(0.53)	0.00(0.16)	0.27(0.22)	0.32(0.56)	0.59(0.58)	0.27(0.27)	5.68E-01	3.09E-01	3.20E-01	9.96E-01	6.48E-01	5.90E-01
98027	0.22(0.54)	0.19(0.18)	-0.38(0.26)	-0.03(0.57)	-0.61(0.60)	-0.58(0.31)	9.60E-01	3.14E-01	7.01E-02	9.96E-01	6.51E-01	2.81E-01
443	-0.09(0.27)	0.06(0.16)	-0.42(0.22)	0.15(0.31)	-0.33(0.34)	-0.48(0.27)	6.32E-01	3.32E-01	8.36E-02	9.96E-01	6.67E-01	3.02E-01
17263	0.02(0.18)	0.26(0.30)	-0.25(0.22)	0.24(0.35)	-0.27(0.28)	-0.51(0.37)	5.01E-01	3.35E-01	1.78E-01	9.96E-01	6.67E-01	4.63E-01
2011	0.05(0.16)	-0.48(0.38)	-0.22(0.24)	-0.53(0.41)	-0.27(0.29)	0.26(0.45)	2.02E-01	3.43E-01	5.71E-01	9.96E-01	6.74E-01	8.05E-01
4600	-0.18(0.23)	0.08(0.20)	-0.49(0.27)	0.26(0.31)	-0.31(0.33)	-0.57(0.34)	4.14E-01	3.45E-01	1.03E-01	9.96E-01	6.74E-01	3.37E-01
106936	-0.20(0.19)	-0.01(0.24)	0.07(0.22)	0.19(0.30)	0.27(0.29)	0.08(0.32)	5.17E-01	3.45E-01	8.01E-01	9.96E-01	6.74E-01	9.38E-01
130396	-0.60(1.14)	0.01(0.12)	-2.04(0.99)	0.61(1.14)	-1.45(1.53)	-2.05(1.00)	5.97E-01	3.47E-01	4.43E-02	9.96E-01	6.75E-01	2.20E-01
117141	-0.09(0.29)	0.45(0.57)	-0.38(0.13)	0.54(0.65)	-0.29(0.31)	-0.83(0.58)	4.08E-01	3.52E-01	1.59E-01	9.96E-01	6.80E-01	4.30E-01
17068	-0.21(0.31)	0.13(0.31)	-0.52(0.12)	0.34(0.44)	-0.31(0.33)	-0.65(0.34)	4.38E-01	3.54E-01	5.69E-02	9.96E-01	6.80E-01	2.62E-01
4926	0.00(0.14)	0.15(0.51)	0.28(0.27)	0.15(0.53)	0.28(0.31)	0.14(0.59)	7.82E-01	3.57E-01	8.17E-01	9.96E-01	6.82E-01	9.41E-01
7718	0.02(0.19)	0.00(0.19)	-0.26(0.24)	-0.01(0.27)	-0.28(0.30)	-0.27(0.31)	9.64E-01	3.62E-01	3.87E-01	9.96E-01	6.89E-01	6.45E-01
5288	-0.58(0.54)	0.13(0.46)	-0.08(0.13)	0.72(0.73)	0.50(0.56)	-0.22(0.48)	3.32E-01	3.70E-01	6.54E-01	9.96E-01	6.94E-01	8.50E-01
146957	-0.27(0.24)	-0.15(0.23)	-0.52(0.16)	0.12(0.33)	-0.25(0.28)	-0.37(0.28)	7.20E-01	3.84E-01	1.88E-01	9.96E-01	7.06E-01	4.80E-01
4937	-0.07(0.23)	0.00(0.29)	-0.33(0.18)	0.07(0.37)	-0.26(0.29)	-0.33(0.35)	8.41E-01	3.86E-01	3.44E-01	9.96E-01	7.06E-01	6.25E-01

5930	-0.04(0.21)	-0.31(0.29)	-0.27(0.16)	-0.27(0.36)	-0.23(0.27)	0.04(0.34)	4.63E-01	3.90E-01	9.17E-01	9.96E-01	7.08E-01	9.82E-01
6580	0.00(0.33)	0.00(0.15)	0.35(0.24)	-0.00(0.36)	0.35(0.40)	0.35(0.28)	9.94E-01	3.93E-01	2.14E-01	9.96E-01	7.08E-01	5.14E-01
3286	-1.35(1.92)	0.00(3.00)	0.30(0.12)	1.35(3.55)	1.65(1.92)	0.30(3.00)	7.05E-01	3.95E-01	9.21E-01	9.96E-01	7.10E-01	9.83E-01
100253	-0.06(0.16)	0.02(0.29)	0.19(0.25)	0.07(0.33)	0.25(0.29)	0.17(0.37)	8.24E-01	4.07E-01	6.41E-01	9.96E-01	7.24E-01	8.36E-01
3258	-0.07(0.18)	0.05(0.24)	-0.29(0.21)	0.12(0.30)	-0.22(0.28)	-0.34(0.32)	6.96E-01	4.17E-01	2.81E-01	9.96E-01	7.38E-01	5.61E-01
119066	-0.34(0.27)	-0.05(0.20)	-0.04(0.27)	0.29(0.33)	0.30(0.38)	0.01(0.33)	3.82E-01	4.28E-01	9.78E-01	9.96E-01	7.49E-01	9.94E-01
4933	0.12(0.27)	0.04(0.16)	-0.18(0.26)	-0.08(0.31)	-0.30(0.38)	-0.22(0.31)	7.98E-01	4.29E-01	4.76E-01	9.96E-01	7.49E-01	7.21E-01
145494	0.21(0.19)	0.01(0.18)	0.48(0.30)	-0.20(0.27)	0.27(0.35)	0.47(0.34)	4.61E-01	4.38E-01	1.74E-01	9.96E-01	7.52E-01	4.63E-01
120562	-0.07(0.22)	0.04(0.18)	-0.32(0.24)	0.11(0.29)	-0.25(0.33)	-0.36(0.30)	7.17E-01	4.43E-01	2.29E-01	9.96E-01	7.52E-01	5.16E-01
2900	-0.16(0.27)	0.52(0.44)	0.14(0.28)	0.68(0.53)	0.30(0.39)	-0.38(0.51)	2.03E-01	4.44E-01	4.62E-01	9.96E-01	7.52E-01	7.04E-01
93947	0.12(0.30)	0.07(0.16)	-0.17(0.21)	-0.05(0.34)	-0.28(0.37)	-0.24(0.27)	8.91E-01	4.45E-01	3.79E-01	9.96E-01	7.52E-01	6.45E-01
307	-0.01(0.19)	0.40(0.27)	-0.21(0.19)	0.41(0.33)	-0.20(0.26)	-0.60(0.34)	2.21E-01	4.59E-01	8.01E-02	9.96E-01	7.64E-01	2.96E-01
17253	-0.04(0.26)	-0.15(0.20)	-0.30(0.23)	-0.11(0.32)	-0.26(0.35)	-0.15(0.30)	7.38E-01	4.60E-01	6.14E-01	9.96E-01	7.64E-01	8.36E-01
6864	-0.29(0.30)	0.08(0.24)	-0.03(0.20)	0.38(0.38)	0.26(0.35)	-0.12(0.31)	3.21E-01	4.65E-01	7.09E-01	9.96E-01	7.68E-01	8.98E-01
47	-0.34(0.23)	-0.02(0.40)	-0.53(0.14)	0.32(0.45)	-0.19(0.26)	-0.51(0.42)	4.75E-01	4.67E-01	2.27E-01	9.96E-01	7.69E-01	5.16E-01
5085	0.02(0.13)	0.00(0.64)	0.48(0.63)	-0.03(0.65)	0.46(0.64)	0.48(0.90)	9.67E-01	4.78E-01	5.91E-01	9.96E-01	7.75E-01	8.23E-01
2039	0.01(0.27)	0.37(0.49)	-0.21(0.14)	0.36(0.57)	-0.22(0.32)	-0.58(0.50)	5.34E-01	4.79E-01	2.48E-01	9.96E-01	7.75E-01	5.46E-01
160542	-0.20(0.15)	0.11(0.32)	-0.40(0.24)	0.31(0.35)	-0.20(0.28)	-0.51(0.39)	3.76E-01	4.82E-01	1.99E-01	9.96E-01	7.75E-01	4.97E-01
4577	-0.64(0.64)	-0.29(0.39)	-0.19(0.13)	0.36(0.75)	0.46(0.65)	0.10(0.41)	6.38E-01	4.83E-01	8.01E-01	9.96E-01	7.75E-01	9.38E-01
4936	0.46(1.54)	-0.04(0.33)	-0.61(0.38)	-0.50(1.57)	-1.08(1.58)	-0.57(0.50)	7.49E-01	4.99E-01	2.57E-01	9.96E-01	7.90E-01	5.53E-01
4609	-0.03(0.24)	-0.10(0.54)	-0.25(0.23)	-0.07(0.59)	-0.22(0.33)	-0.15(0.58)	9.07E-01	5.01E-01	7.95E-01	9.96E-01	7.91E-01	9.37E-01
4986	0.12(0.33)	0.02(0.18)	-0.13(0.19)	-0.11(0.38)	-0.25(0.38)	-0.15(0.26)	7.77E-01	5.07E-01	5.72E-01	9.96E-01	7.95E-01	8.05E-01
2936	-0.44(0.57)	-0.13(0.44)	-0.06(0.13)	0.31(0.72)	0.38(0.58)	0.08(0.45)	6.71E-01	5.14E-01	8.69E-01	9.96E-01	8.00E-01	9.61E-01
87710	0.40(0.83)	-0.02(0.16)	-0.15(0.23)	-0.42(0.84)	-0.55(0.86)	-0.13(0.28)	6.23E-01	5.25E-01	6.41E-01	9.96E-01	8.10E-01	8.36E-01
160	0.02(0.16)	0.39(0.43)	-0.30(0.50)	0.37(0.45)	-0.32(0.52)	-0.69(0.65)	4.14E-01	5.38E-01	2.93E-01	9.96E-01	8.20E-01	5.69E-01
160463	0.12(0.23)	0.25(0.23)	-0.06(0.17)	0.14(0.32)	-0.18(0.29)	-0.31(0.29)	6.72E-01	5.40E-01	2.84E-01	9.96E-01	8.20E-01	5.61E-01
160313	-0.03(0.20)	-0.68(0.62)	0.13(0.17)	-0.66(0.65)	0.16(0.27)	0.81(0.64)	3.20E-01	5.56E-01	2.11E-01	9.96E-01	8.37E-01	5.14E-01
112487	-0.27(0.36)	0.09(0.31)	-0.50(0.12)	0.36(0.49)	-0.23(0.38)	-0.59(0.34)	4.65E-01	5.58E-01	8.49E-02	9.96E-01	8.37E-01	3.02E-01
4863	-0.03(0.23)	0.51(0.41)	-0.19(0.15)	0.54(0.46)	-0.16(0.28)	-0.70(0.44)	2.47E-01	5.60E-01	1.13E-01	9.96E-01	8.37E-01	3.58E-01
17453	-0.47(0.63)	0.02(0.13)	-0.94(0.51)	0.49(0.64)	-0.47(0.81)	-0.96(0.53)	4.44E-01	5.65E-01	7.28E-02	9.96E-01	8.37E-01	2.89E-01

4898	-0.27(0.25)	0.05(0.21)	-0.44(0.16)	0.32(0.33)	-0.17(0.29)	-0.49(0.27)	3.41E-01	5.66E-01	7.74E-02	9.96E-01	8.37E-01	2.96E-01
93385	-0.17(0.19)	0.21(0.28)	-0.35(0.26)	0.38(0.33)	-0.18(0.32)	-0.56(0.38)	2.61E-01	5.80E-01	1.46E-01	9.96E-01	8.55E-01	4.16E-01
145492	-0.85(0.81)	0.00(0.13)	-0.38(0.29)	0.85(0.82)	0.47(0.86)	-0.38(0.31)	3.02E-01	5.86E-01	2.29E-01	9.96E-01	8.56E-01	5.16E-01
21513	-0.03(0.14)	0.20(0.34)	0.20(0.39)	0.23(0.36)	0.23(0.42)	0.00(0.53)	5.28E-01	5.86E-01	9.96E-01	9.96E-01	8.56E-01	9.98E-01
8607	0.09(0.20)	0.17(0.20)	-0.07(0.23)	0.08(0.28)	-0.16(0.30)	-0.24(0.31)	7.81E-01	5.93E-01	4.46E-01	9.96E-01	8.57E-01	6.93E-01
119129	-0.03(0.16)	0.02(0.21)	-0.27(0.43)	0.05(0.27)	-0.25(0.46)	-0.29(0.48)	8.58E-01	5.94E-01	5.41E-01	9.96E-01	8.57E-01	7.79E-01
110	-0.19(0.35)	-0.39(0.41)	0.00(0.15)	-0.20(0.53)	0.19(0.38)	0.39(0.43)	7.05E-01	6.21E-01	3.67E-01	9.96E-01	8.78E-01	6.45E-01
4976	0.00(0.15)	-0.03(0.28)	-0.17(0.32)	-0.04(0.32)	-0.18(0.36)	-0.14(0.43)	9.11E-01	6.26E-01	7.44E-01	9.96E-01	8.79E-01	9.10E-01
64551	-0.13(0.20)	-0.18(0.25)	0.00(0.18)	-0.05(0.32)	0.13(0.27)	0.18(0.31)	8.69E-01	6.30E-01	5.52E-01	9.96E-01	8.79E-01	7.91E-01
1996	-0.38(0.28)	0.17(0.58)	-0.24(0.14)	0.56(0.63)	0.14(0.31)	-0.41(0.58)	3.82E-01	6.39E-01	4.81E-01	9.96E-01	8.79E-01	7.23E-01
109423	-0.19(0.25)	-0.30(0.14)	-0.38(0.35)	-0.11(0.28)	-0.19(0.43)	-0.08(0.38)	6.96E-01	6.57E-01	8.32E-01	9.96E-01	8.94E-01	9.51E-01
6331	-0.22(0.27)	0.21(0.24)	-0.36(0.19)	0.43(0.37)	-0.14(0.33)	-0.57(0.31)	2.42E-01	6.66E-01	6.74E-02	9.96E-01	8.94E-01	2.78E-01
14509	-0.15(0.21)	-0.16(0.31)	-0.27(0.19)	-0.01(0.38)	-0.12(0.28)	-0.10(0.37)	9.75E-01	6.79E-01	7.78E-01	9.96E-01	8.94E-01	9.28E-01
107960	-0.14(0.39)	0.02(0.27)	-0.31(0.15)	0.15(0.47)	-0.17(0.42)	-0.33(0.32)	7.44E-01	6.79E-01	3.07E-01	9.96E-01	8.94E-01	5.85E-01
153	-0.39(0.46)	0.21(0.35)	-0.21(0.14)	0.60(0.59)	0.19(0.47)	-0.41(0.38)	3.14E-01	6.93E-01	2.80E-01	9.96E-01	9.02E-01	5.61E-01
91421	-0.21(0.34)	-0.10(0.20)	-0.06(0.18)	0.12(0.39)	0.15(0.39)	0.04(0.27)	7.65E-01	6.97E-01	8.94E-01	9.96E-01	9.02E-01	9.69E-01
31357	-0.11(0.21)	-0.03(0.17)	-0.24(0.26)	0.08(0.27)	-0.13(0.33)	-0.21(0.31)	7.61E-01	7.09E-01	5.04E-01	9.96E-01	9.02E-01	7.42E-01
119023	0.23(0.51)	0.04(0.13)	-0.03(0.55)	-0.19(0.53)	-0.26(0.73)	-0.07(0.57)	7.12E-01	7.23E-01	9.08E-01	9.96E-01	9.02E-01	9.78E-01
4922	0.00(0.26)	-0.30(0.25)	0.20(0.52)	-0.30(0.36)	0.20(0.58)	0.50(0.57)	4.13E-01	7.31E-01	3.87E-01	9.96E-01	9.02E-01	6.45E-01
4941	-0.08(0.29)	0.15(0.23)	-0.20(0.22)	0.23(0.37)	-0.12(0.36)	-0.35(0.31)	5.32E-01	7.39E-01	2.65E-01	9.96E-01	9.02E-01	5.53E-01
135889	1.44(3.50)	0.28(0.22)	0.26(0.28)	-1.16(3.51)	-1.18(3.54)	-0.03(0.35)	7.43E-01	7.39E-01	9.43E-01	9.96E-01	9.02E-01	9.87E-01
4925	-0.07(0.23)	0.12(0.21)	0.04(0.22)	0.19(0.32)	0.10(0.32)	-0.09(0.30)	5.44E-01	7.43E-01	7.68E-01	9.96E-01	9.02E-01	9.23E-01
31559	0.17(0.44)	0.00(0.18)	0.01(0.18)	-0.17(0.47)	-0.16(0.48)	0.02(0.25)	7.18E-01	7.43E-01	9.51E-01	9.96E-01	9.02E-01	9.87E-01
1878	-0.27(0.22)	-0.18(0.18)	-0.37(0.21)	0.09(0.28)	-0.10(0.31)	-0.19(0.28)	7.52E-01	7.45E-01	5.04E-01	9.96E-01	9.02E-01	7.42E-01
4983	-0.20(0.37)	0.16(0.24)	-0.07(0.16)	0.36(0.45)	0.13(0.40)	-0.23(0.29)	4.21E-01	7.47E-01	4.20E-01	9.96E-01	9.02E-01	6.71E-01
41699	-0.19(0.32)	0.07(0.43)	-0.08(0.14)	0.26(0.55)	0.11(0.35)	-0.15(0.45)	6.41E-01	7.51E-01	7.50E-01	9.96E-01	9.02E-01	9.12E-01
5482	-0.20(0.25)	-0.02(0.17)	-0.09(0.27)	0.18(0.30)	0.11(0.35)	-0.07(0.32)	5.66E-01	7.52E-01	8.37E-01	9.96E-01	9.02E-01	9.51E-01
4975	0.16(0.24)	0.06(0.21)	0.06(0.20)	-0.10(0.32)	-0.10(0.31)	0.00(0.28)	7.61E-01	7.54E-01	9.98E-01	9.96E-01	9.02E-01	9.98E-01
16788	0.00(0.17)	0.24(0.22)	0.10(0.26)	0.24(0.28)	0.10(0.32)	-0.14(0.34)	3.91E-01	7.55E-01	6.69E-01	9.96E-01	9.02E-01	8.63E-01
6579	-0.19(0.23)	0.01(0.23)	-0.10(0.18)	0.20(0.33)	0.09(0.30)	-0.11(0.29)	5.55E-01	7.61E-01	7.20E-01	9.96E-01	9.02E-01	8.98E-01

1675	-0.08(0.42)	0.74(0.55)	0.05(0.12)	0.82(0.70)	0.13(0.44)	-0.69(0.57)	2.46E-01	7.63E-01	2.27E-01	9.96E-01	9.02E-01	5.16E-01
7053	0.07(0.28)	0.02(0.33)	-0.02(0.17)	-0.05(0.43)	-0.10(0.33)	-0.05(0.37)	9.02E-01	7.66E-01	9.04E-01	9.96E-01	9.02E-01	9.77E-01
9392	0.00(0.28)	-0.01(0.18)	0.10(0.24)	-0.00(0.33)	0.11(0.37)	0.11(0.30)	9.95E-01	7.70E-01	7.17E-01	9.96E-01	9.02E-01	8.98E-01
4971	-0.08(0.20)	0.03(0.20)	0.01(0.24)	0.11(0.29)	0.09(0.32)	-0.02(0.31)	7.05E-01	7.87E-01	9.39E-01	9.96E-01	9.09E-01	9.87E-01
122151	-0.04(0.35)	0.15(0.26)	-0.15(0.16)	0.20(0.44)	-0.10(0.38)	-0.30(0.30)	6.56E-01	7.89E-01	3.15E-01	9.96E-01	9.09E-01	5.88E-01
120526	-0.14(0.34)	0.35(0.30)	-0.23(0.15)	0.48(0.45)	-0.10(0.37)	-0.58(0.34)	2.87E-01	7.90E-01	8.74E-02	9.96E-01	9.09E-01	3.08E-01
448	-0.03(0.26)	-0.12(0.21)	-0.11(0.21)	-0.10(0.33)	-0.09(0.34)	0.01(0.31)	7.71E-01	8.00E-01	9.74E-01	9.96E-01	9.11E-01	9.94E-01
103102	-0.37(0.35)	-0.69(0.45)	-0.28(0.13)	-0.32(0.56)	0.09(0.37)	0.41(0.46)	5.75E-01	8.02E-01	3.70E-01	9.96E-01	9.11E-01	6.45E-01
139436	-0.15(0.19)	-0.10(0.21)	-0.23(0.29)	0.05(0.28)	-0.08(0.33)	-0.13(0.35)	8.60E-01	8.13E-01	7.19E-01	9.96E-01	9.15E-01	8.98E-01
469	-0.08(0.25)	0.17(0.27)	-0.15(0.16)	0.25(0.37)	-0.07(0.29)	-0.32(0.31)	4.95E-01	8.15E-01	3.03E-01	9.96E-01	9.15E-01	5.82E-01
47170	-0.18(0.49)	-0.06(0.15)	-0.29(0.22)	0.13(0.51)	-0.11(0.54)	-0.24(0.26)	8.07E-01	8.39E-01	3.72E-01	9.96E-01	9.33E-01	6.45E-01
34075	-0.13(0.55)	0.17(0.17)	-0.24(0.29)	0.29(0.58)	-0.11(0.62)	-0.41(0.33)	6.14E-01	8.56E-01	2.30E-01	9.96E-01	9.43E-01	5.16E-01
573	-0.01(0.28)	-0.07(0.24)	-0.07(0.17)	-0.05(0.37)	-0.06(0.33)	-0.01(0.30)	8.87E-01	8.58E-01	9.83E-01	9.96E-01	9.43E-01	9.96E-01
4938	-0.34(1.96)	0.34(0.99)	0.01(0.12)	0.69(2.24)	0.35(1.97)	-0.33(1.00)	7.60E-01	8.58E-01	7.39E-01	9.96E-01	9.43E-01	9.10E-01
16561	-0.05(0.27)	-0.06(0.26)	0.00(0.17)	-0.01(0.38)	0.05(0.32)	0.06(0.30)	9.84E-01	8.64E-01	8.38E-01	9.96E-01	9.46E-01	9.51E-01
4932	0.42(1.26)	-0.02(0.15)	0.21(0.23)	-0.44(1.28)	-0.21(1.29)	0.22(0.27)	7.34E-01	8.70E-01	4.10E-01	9.96E-01	9.46E-01	6.61E-01
490	-0.02(0.20)	-0.11(0.61)	0.01(0.16)	-0.08(0.64)	0.04(0.25)	0.12(0.63)	8.97E-01	8.81E-01	8.48E-01	9.96E-01	9.53E-01	9.54E-01
1064	0.06(0.24)	0.21(0.48)	0.10(0.14)	0.14(0.54)	0.04(0.28)	-0.10(0.51)	7.91E-01	8.86E-01	8.39E-01	9.96E-01	9.53E-01	9.51E-01
4871	-0.04(0.30)	0.02(0.28)	0.01(0.15)	0.06(0.40)	0.05(0.33)	-0.02(0.31)	8.76E-01	8.88E-01	9.57E-01	9.96E-01	9.53E-01	9.87E-01
4948	0.11(0.19)	0.43(0.57)	0.13(0.16)	0.32(0.60)	0.03(0.25)	-0.30(0.59)	5.92E-01	9.17E-01	6.15E-01	9.96E-01	9.77E-01	8.36E-01
1704	-0.05(0.17)	-0.26(0.29)	-0.01(0.37)	-0.21(0.34)	0.04(0.41)	0.25(0.45)	5.38E-01	9.23E-01	5.85E-01	9.96E-01	9.78E-01	8.17E-01
109387	0.06(0.36)	0.01(0.39)	0.03(0.14)	-0.05(0.52)	-0.04(0.38)	0.02(0.41)	9.20E-01	9.23E-01	9.69E-01	9.96E-01	9.78E-01	9.93E-01
111162	0.01(0.18)	-0.29(0.49)	-0.07(0.79)	-0.30(0.52)	-0.08(0.81)	0.22(0.93)	5.63E-01	9.26E-01	8.11E-01	9.96E-01	9.78E-01	9.41E-01
139478	-0.08(0.28)	-0.10(0.15)	0.11(2.10)	-0.02(0.32)	0.18(2.13)	0.21(2.11)	9.45E-01	9.32E-01	9.23E-01	9.96E-01	9.79E-01	9.83E-01
4533	-0.07(0.57)	0.03(0.16)	-0.02(0.22)	0.10(0.59)	0.05(0.61)	-0.05(0.28)	8.63E-01	9.33E-01	8.57E-01	9.96E-01	9.79E-01	9.61E-01
160464	-0.05(0.23)	-0.07(0.19)	-0.08(0.23)	-0.02(0.30)	-0.03(0.33)	-0.01(0.30)	9.55E-01	9.39E-01	9.78E-01	9.96E-01	9.80E-01	9.94E-01
137	0.07(0.20)	-0.09(0.28)	0.09(0.19)	-0.16(0.35)	0.02(0.27)	0.18(0.35)	6.48E-01	9.52E-01	6.10E-01	9.96E-01	9.86E-01	8.36E-01
479	-0.29(0.34)	0.03(0.16)	-0.31(0.21)	0.32(0.38)	-0.02(0.40)	-0.34(0.27)	4.06E-01	9.60E-01	2.14E-01	9.96E-01	9.92E-01	5.14E-01
61	-0.40(0.46)	-0.16(0.30)	-0.42(0.14)	0.25(0.55)	-0.02(0.47)	-0.26(0.33)	6.54E-01	9.73E-01	4.22E-01	9.96E-01	9.99E-01	6.71E-01
26918	-0.12(0.23)	0.01(0.19)	-0.13(0.21)	0.13(0.30)	-0.01(0.32)	-0.14(0.28)	6.65E-01	9.73E-01	6.22E-01	9.96E-01	9.99E-01	8.36E-01

18488	0.00(0.24)	-0.05(0.21)	-0.01(0.19)	-0.05(0.32)	-0.01(0.31)	0.04(0.28)	8.78E-01	9.79E-01	8.84E-01	9.96E-01	9.99E-01	9.68E-01
41691	-0.30(0.61)	-0.31(0.38)	-0.31(0.14)	-0.00(0.71)	-0.01(0.63)	-0.01(0.40)	9.94E-01	9.87E-01	9.90E-01	9.96E-01	1.00E+0	9.98E-01
4928	-0.07(0.27)	-0.30(0.28)	-0.06(1.59)	-0.23(0.39)	0.01(1.60)	0.24(1.61)	5.53E-01	9.97E-01	8.85E-01	9.96E-01	1.00E+0	9.68E-01
4956	-0.07(0.66)	-0.02(0.13)	-0.07(0.32)	0.05(0.67)	0.00(0.73)	-0.05(0.34)	9.40E-01	1.00E+0	8.84E-01	9.96E-01	1.00E+0	9.68E-01

Regression coefficient β* (standard error (SE)) denotes the association between % change in metabolites and % change in PFG in each intervention group (IMI, BAND or RYGB). β† (SE) denotes the association between % change in metabolites and % change in FPG induced by different types of weight-loss interventions (BAND vs. IMI, RYGB vs. IMI, and RYGB vs. BAND), and was obtained from the interaction term (% change in metabolites × types of intervention) in a linear regression model. In the model, the dependent variable was % change in FPG, and % change in each metabolite, intervention groups (RYGB, BAND, IMI), and their interactions (% change in each metabolite × types of intervention) were the independent variables, adjusting for weight loss, sex, baseline age and BMI.

Table S4. Differential association between changes in metabolites and change in HbA1c before and 1-yr after intervention

	β*(SE)			β† (SE)			p-value			q-value		
**Metabolites**	**IMI**	**BAND**	**RYGB**	**BAND *vs.* IMI**	**RYGB *vs.* IMI**	**RYGB *vs.* BAND**	**BAND *vs.* IMI**	**RYGB vs. IMI**	**RYGB *vs.* BAND**	**BAND *vs.* IMI**	**RYGB *vs.* IMI**	**RYGB *vs.* BAND**
**Known**												
Allantoin	0.01(0.12)	-0.06(0.21)	-2.28(0.58)	-0.07(0.24)	-2.29(0.60)	-2.22(0.62)	7.84E-01	2.87E-04	6.56E-04	9.95E-01	2.81E-02	4.79E-02
Aminomalonate	-0.05(0.09)	-0.22(0.37)	-0.86(0.21)	-0.17(0.38)	-0.82(0.23)	-0.65(0.42)	6.62E-01	7.72E-04	1.28E-01	9.95E-01	5.82E-02	4.91E-01
1-monostearin	0.16(0.16)	0.13(0.11)	-0.62(0.17)	-0.02(0.20)	-0.77(0.23)	-0.75(0.20)	9.06E-01	1.44E-03	4.74E-04	9.95E-01	9.02E-02	4.47E-02
Fumaric acid	-0.03(0.10)	0.07(0.33)	-0.83(0.24)	0.09(0.34)	-0.80(0.26)	-0.89(0.40)	7.89E-01	2.90E-03	3.03E-02	9.95E-01	1.21E-01	2.57E-01
Pyrophosphate	0.01(0.09)	0.32(0.76)	-1.39(0.46)	0.32(0.77)	-1.40(0.47)	-1.71(0.90)	6.83E-01	4.47E-03	6.01E-02	9.95E-01	1.53E-01	3.33E-01
Shikimic acid	0.00(0.13)	0.07(0.14)	-0.63(0.17)	0.07(0.19)	-0.63(0.22)	-0.70(0.21)	7.25E-01	4.73E-03	1.57E-03	9.95E-01	1.53E-01	6.59E-02
Oxoproline	-0.05(0.16)	-0.30(0.13)	-0.61(0.11)	-0.25(0.20)	-0.56(0.19)	-0.31(0.17)	2.30E-01	5.15E-03	7.31E-02	9.95E-01	1.53E-01	3.67E-01
Parabanic acid NIST	-0.03(0.11)	0.10(0.16)	-0.75(0.22)	0.13(0.19)	-0.72(0.25)	-0.86(0.27)	4.85E-01	5.56E-03	2.69E-03	9.95E-01	1.53E-01	7.72E-02
Tocopherol gamma-	-0.02(0.19)	-0.16(0.10)	-1.42(0.45)	-0.14(0.22)	-1.40(0.49)	-1.26(0.46)	5.37E-01	6.16E-03	8.18E-03	9.95E-01	1.53E-01	1.29E-01
Lactic acid	0.09(0.11)	-0.22(0.23)	0.71(0.19)	-0.31(0.25)	0.63(0.22)	0.94(0.30)	2.26E-01	6.40E-03	2.63E-03	9.95E-01	1.53E-01	7.72E-02
Phosphoethanolamine	0.04(0.19)	0.07(0.13)	-0.56(0.13)	0.03(0.22)	-0.60(0.22)	-0.63(0.18)	8.81E-01	9.06E-03	7.63E-04	9.95E-01	1.71E-01	4.79E-02
2-hydroxyglutaric acid	-0.05(0.12)	-0.28(0.26)	-0.51(0.13)	-0.23(0.28)	-0.46(0.18)	-0.23(0.30)	4.12E-01	1.14E-02	4.42E-01	9.95E-01	1.81E-01	7.54E-01
Glycerol	-0.04(0.15)	-0.15(0.15)	0.47(0.14)	-0.11(0.21)	0.52(0.20)	0.62(0.20)	6.05E-01	1.29E-02	2.85E-03	9.95E-01	1.87E-01	7.72E-02
Indole-3-lactate	0.11(0.14)	-0.14(0.18)	-0.39(0.14)	-0.25(0.23)	-0.50(0.20)	-0.25(0.23)	2.69E-01	1.72E-02	2.87E-01	9.95E-01	2.24E-01	6.29E-01
Maltotriose	0.07(0.21)	0.00(0.12)	-0.53(0.14)	-0.07(0.24)	-0.60(0.25)	-0.53(0.18)	7.77E-01	1.79E-02	4.71E-03	9.95E-01	2.24E-01	9.87E-02
Glycine	-0.08(0.14)	-0.35(0.32)	-0.55(0.13)	-0.27(0.34)	-0.46(0.19)	-0.19(0.33)	4.31E-01	1.82E-02	5.71E-01	9.95E-01	2.24E-01	8.41E-01
Pelargonic acid	-0.01(0.13)	0.07(0.21)	-0.51(0.16)	0.08(0.25)	-0.50(0.21)	-0.58(0.26)	7.33E-01	1.91E-02	2.78E-02	9.95E-01	2.24E-01	2.57E-01
Lactulose	0.02(0.11)	0.25(0.23)	0.69(0.28)	0.23(0.25)	0.67(0.30)	0.45(0.36)	3.73E-01	2.93E-02	2.25E-01	9.95E-01	3.08E-01	5.84E-01
Pentadecanoic acid	0.10(0.17)	0.09(0.19)	-0.36(0.12)	-0.00(0.26)	-0.46(0.21)	-0.46(0.23)	9.89E-01	3.03E-02	4.78E-02	9.96E-01	3.08E-01	2.88E-01
Xylose	-0.01(0.10)	-0.15(0.30)	0.89(0.40)	-0.14(0.31)	0.90(0.41)	1.04(0.50)	6.66E-01	3.11E-02	4.21E-02	9.95E-01	3.08E-01	2.78E-01
UDP GlcNAc	0.01(0.12)	0.06(0.19)	-0.41(0.17)	0.06(0.23)	-0.41(0.21)	-0.47(0.26)	8.05E-01	4.96E-02	7.65E-02	9.95E-01	4.45E-01	3.73E-01
3-aminoisobutyric acid	0.12(0.23)	-0.13(0.21)	-0.38(0.12)	-0.24(0.31)	-0.50(0.26)	-0.25(0.23)	4.28E-01	5.76E-02	2.82E-01	9.95E-01	4.92E-01	6.22E-01
1-monopalmitin	-0.02(0.22)	0.03(0.10)	-0.81(0.35)	0.05(0.24)	-0.79(0.42)	-0.84(0.36)	8.39E-01	6.13E-02	2.34E-02	9.95E-01	4.92E-01	2.54E-01
Mannitol	-0.01(0.11)	0.10(0.17)	0.67(0.36)	0.11(0.20)	0.69(0.38)	0.57(0.40)	5.80E-01	7.17E-02	1.51E-01	9.95E-01	5.20E-01	5.15E-01

Sorbitol	-0.22(1.10)	0.05(0.09)	2.22(0.79)	0.27(1.10)	2.44(1.34)	2.16(0.79)	8.04E-01	7.41E-02	8.19E-03	9.95E-01	5.22E-01	1.29E-01
Benzoic acid	0.05(0.12)	-0.17(0.25)	-0.91(0.51)	-0.22(0.28)	-0.96(0.53)	-0.74(0.57)	4.32E-01	7.47E-02	1.97E-01	9.95E-01	5.22E-01	5.49E-01
Proline	0.08(0.23)	0.01(0.10)	-0.86(0.48)	-0.06(0.25)	-0.94(0.53)	-0.88(0.49)	8.02E-01	7.98E-02	7.61E-02	9.95E-01	5.37E-01	3.73E-01
Oleic acid	-0.04(0.15)	-0.13(0.25)	0.29(0.12)	-0.09(0.29)	0.34(0.19)	0.43(0.27)	7.60E-01	8.80E-02	1.21E-01	9.95E-01	5.44E-01	4.84E-01
3-hydroxybutyric acid	0.01(0.15)	-0.01(0.12)	0.61(0.31)	-0.02(0.19)	0.60(0.35)	0.62(0.33)	9.37E-01	8.97E-02	6.97E-02	9.95E-01	5.44E-01	3.67E-01
Urea	-0.06(0.13)	-0.06(0.13)	-0.66(0.33)	-0.01(0.19)	-0.61(0.36)	-0.60(0.36)	9.73E-01	9.28E-02	9.82E-02	9.95E-01	5.44E-01	4.31E-01
Pyruvic acid	-0.14(0.21)	-0.03(0.14)	0.28(0.13)	0.11(0.25)	0.42(0.25)	0.31(0.19)	6.71E-01	9.39E-02	1.13E-01	9.95E-01	5.44E-01	4.65E-01
Aspartic acid	-0.02(0.17)	0.02(0.21)	-0.38(0.13)	0.04(0.26)	-0.36(0.21)	-0.40(0.25)	8.73E-01	1.00E-01	1.13E-01	9.95E-01	5.44E-01	4.65E-01
Glutamine	0.14(0.22)	0.04(0.13)	-0.33(0.17)	-0.10(0.25)	-0.46(0.28)	-0.36(0.21)	6.81E-01	1.07E-01	9.48E-02	9.95E-01	5.44E-01	4.30E-01
Butane-2,3-diol NIST	-0.01(0.16)	0.23(0.22)	-0.38(0.17)	0.24(0.27)	-0.37(0.23)	-0.61(0.27)	3.91E-01	1.09E-01	3.04E-02	9.95E-01	5.44E-01	2.57E-01
2-hydroxypyrazinyl-2-propenoic acid ethyl ester NIST	-0.13(0.14)	0.09(0.19)	-0.45(0.14)	0.22(0.23)	-0.33(0.20)	-0.54(0.23)	3.55E-01	1.13E-01	2.36E-02	9.95E-01	5.44E-01	2.54E-01
Maltose	0.49(0.63)	0.03(0.10)	-0.92(0.62)	-0.46(0.63)	-1.41(0.88)	-0.95(0.63)	4.68E-01	1.14E-01	1.36E-01	9.95E-01	5.44E-01	4.93E-01
Fructose	0.12(0.33)	0.05(0.10)	1.28(0.64)	-0.07(0.34)	1.16(0.73)	1.23(0.65)	8.38E-01	1.16E-01	6.23E-02	9.95E-01	5.44E-01	3.40E-01
Creatinine	0.05(0.09)	0.41(0.79)	-0.73(0.49)	0.36(0.79)	-0.78(0.50)	-1.14(0.91)	6.53E-01	1.24E-01	2.18E-01	9.95E-01	5.44E-01	5.73E-01
3-(1-pyrazolyl)-L-alanine	-0.08(0.34)	0.00(0.09)	-0.79(0.28)	0.08(0.35)	-0.70(0.45)	-0.78(0.29)	8.19E-01	1.24E-01	9.39E-03	9.95E-01	5.44E-01	1.42E-01
Isothreonic acid	-0.13(0.16)	-0.20(0.21)	0.19(0.14)	-0.08(0.26)	0.32(0.21)	0.40(0.26)	7.65E-01	1.34E-01	1.26E-01	9.95E-01	5.60E-01	4.91E-01
Allantoic acid	0.23(0.37)	0.15(0.15)	-0.38(0.18)	-0.08(0.40)	-0.61(0.41)	-0.53(0.23)	8.39E-01	1.39E-01	2.17E-02	9.95E-01	5.64E-01	2.54E-01
Palmitic acid	-0.01(0.16)	0.05(0.13)	0.36(0.20)	0.06(0.21)	0.38(0.26)	0.32(0.24)	7.67E-01	1.52E-01	1.99E-01	9.95E-01	6.01E-01	5.49E-01
Glutamic acid	0.07(0.14)	0.16(0.16)	0.41(0.19)	0.09(0.21)	0.34(0.24)	0.25(0.25)	6.75E-01	1.61E-01	3.28E-01	9.95E-01	6.19E-01	6.63E-01
Adenosine-5-monophosphate	0.06(0.26)	-0.01(0.19)	-0.33(0.11)	-0.07(0.32)	-0.40(0.29)	-0.33(0.22)	8.29E-01	1.73E-01	1.36E-01	9.95E-01	6.20E-01	4.93E-01
Kynurenine	-0.04(0.30)	0.06(0.10)	-0.59(0.28)	0.09(0.32)	-0.56(0.41)	-0.65(0.30)	7.69E-01	1.77E-01	3.17E-02	9.95E-01	6.20E-01	2.57E-01
Capric acid	0.00(0.15)	0.13(0.19)	-0.35(0.21)	0.13(0.25)	-0.35(0.26)	-0.49(0.28)	5.92E-01	1.82E-01	8.84E-02	9.95E-01	6.20E-01	4.11E-01
Hypoxanthine	0.05(0.10)	0.16(0.31)	-0.33(0.26)	0.11(0.33)	-0.38(0.28)	-0.49(0.41)	7.44E-01	1.83E-01	2.35E-01	9.95E-01	6.20E-01	5.89E-01
Salicylic acid	-0.03(0.10)	0.20(0.39)	0.36(0.28)	0.24(0.40)	0.40(0.30)	0.16(0.48)	5.53E-01	1.89E-01	7.41E-01	9.95E-01	6.24E-01	9.11E-01
Palmitoleic acid	-0.04(0.13)	-0.03(0.27)	0.21(0.15)	0.02(0.30)	0.25(0.19)	0.23(0.30)	9.58E-01	1.98E-01	4.43E-01	9.95E-01	6.37E-01	7.54E-01
Uric acid	0.13(0.32)	-0.10(0.18)	-0.31(0.12)	-0.23(0.37)	-0.44(0.34)	-0.21(0.21)	5.36E-01	1.99E-01	3.22E-01	9.95E-01	6.37E-01	6.63E-01
Ribonic acid	0.01(0.15)	-0.02(0.24)	0.37(0.23)	-0.03(0.29)	0.35(0.27)	0.38(0.33)	9.10E-01	2.03E-01	2.46E-01	9.95E-01	6.37E-01	6.07E-01
Caprylic acid	-0.10(0.15)	-0.05(0.19)	-0.37(0.15)	0.06(0.24)	-0.27(0.21)	-0.33(0.23)	8.17E-01	2.06E-01	1.65E-01	9.95E-01	6.37E-01	5.34E-01

Lauric acid	0.05(0.15)	-0.03(0.14)	-0.33(0.27)	-0.09(0.21)	-0.39(0.31)	-0.30(0.31)	6.73E-01	2.16E-01	3.29E-01	9.95E-01	6.46E-01	6.63E-01
2,4-diaminobutyric acid	0.05(0.13)	0.19(0.21)	-0.19(0.16)	0.14(0.25)	-0.24(0.20)	-0.38(0.26)	5.84E-01	2.28E-01	1.50E-01	9.95E-01	6.76E-01	5.15E-01
Dodecanol	-0.10(0.15)	0.01(0.18)	-0.33(0.13)	0.10(0.24)	-0.23(0.20)	-0.34(0.23)	6.70E-01	2.53E-01	1.41E-01	9.95E-01	7.02E-01	4.96E-01
Glucose	-0.11(0.22)	0.47(0.20)	0.17(0.11)	0.58(0.30)	0.28(0.24)	-0.30(0.23)	5.33E-02	2.55E-01	1.93E-01	9.95E-01	7.02E-01	5.49E-01
Hydroxyproline dipeptide NIST	0.41(0.55)	-0.78(0.35)	-0.22(0.10)	-1.19(0.66)	-0.64(0.56)	0.55(0.37)	7.44E-02	2.58E-01	1.35E-01	9.95E-01	7.06E-01	4.93E-01
Glycolic acid	0.05(0.15)	0.06(0.16)	0.29(0.16)	0.01(0.22)	0.24(0.21)	0.23(0.23)	9.68E-01	2.71E-01	3.22E-01	9.95E-01	7.19E-01	6.63E-01
Cysteine	-0.03(0.15)	0.05(0.23)	-0.25(0.14)	0.08(0.26)	-0.22(0.20)	-0.29(0.26)	7.68E-01	2.87E-01	2.64E-01	9.95E-01	7.22E-01	6.19E-01
N-acetylmannosamine	0.14(0.22)	0.02(0.10)	0.56(0.33)	-0.11(0.24)	0.42(0.39)	0.54(0.35)	6.41E-01	2.87E-01	1.27E-01	9.95E-01	7.22E-01	4.91E-01
Phenylalanine	-0.13(0.22)	0.00(0.16)	-0.39(0.11)	0.13(0.27)	-0.26(0.24)	-0.39(0.19)	6.26E-01	2.88E-01	4.81E-02	9.95E-01	7.22E-01	2.88E-01
Gluconic acid	0.04(0.13)	-0.31(0.12)	0.53(0.44)	-0.35(0.18)	0.48(0.46)	0.83(0.45)	5.48E-02	2.92E-01	7.11E-02	9.95E-01	7.22E-01	3.67E-01
Ketohexose	0.01(0.11)	0.09(0.18)	0.31(0.26)	0.08(0.21)	0.30(0.29)	0.23(0.32)	7.09E-01	2.94E-01	4.77E-01	9.95E-01	7.22E-01	7.65E-01
Hydrocinnamic acid	0.03(0.13)	0.15(0.20)	-0.50(0.49)	0.12(0.24)	-0.53(0.51)	-0.65(0.52)	6.20E-01	2.95E-01	2.17E-01	9.95E-01	7.22E-01	5.73E-01
Tocopherol alpha-	-0.10(0.25)	-0.18(0.11)	0.24(0.22)	-0.08(0.27)	0.34(0.33)	0.42(0.25)	7.75E-01	3.04E-01	9.61E-02	9.95E-01	7.22E-01	4.31E-01
Threonine	-0.20(0.17)	0.15(0.17)	-0.42(0.12)	0.35(0.24)	-0.22(0.22)	-0.57(0.20)	1.44E-01	3.22E-01	7.05E-03	9.95E-01	7.28E-01	1.29E-01
Myo-inositol	-0.14(0.27)	-0.03(0.17)	0.15(0.13)	0.12(0.32)	0.29(0.29)	0.18(0.21)	7.20E-01	3.22E-01	4.13E-01	9.95E-01	7.28E-01	7.35E-01
Arachidic acid	0.08(0.33)	-0.03(0.17)	-0.25(0.11)	-0.11(0.37)	-0.33(0.34)	-0.22(0.20)	7.69E-01	3.42E-01	2.77E-01	9.95E-01	7.49E-01	6.19E-01
Methionine	0.10(0.40)	0.17(0.16)	-0.31(0.15)	0.08(0.42)	-0.40(0.43)	-0.48(0.22)	8.53E-01	3.45E-01	3.12E-02	9.95E-01	7.49E-01	2.57E-01
Aconitic acid	-0.24(0.30)	-0.43(0.27)	0.05(0.11)	-0.19(0.40)	0.29(0.32)	0.49(0.28)	6.38E-01	3.60E-01	9.33E-02	9.95E-01	7.71E-01	4.29E-01
Indole-3-acetate	0.15(0.34)	-0.38(0.25)	-0.18(0.11)	-0.53(0.43)	-0.33(0.36)	0.20(0.28)	2.20E-01	3.64E-01	4.66E-01	9.95E-01	7.76E-01	7.54E-01
N-acetylornithine	0.03(0.20)	-0.12(0.14)	-0.29(0.30)	-0.15(0.24)	-0.32(0.36)	-0.17(0.33)	5.32E-01	3.72E-01	6.11E-01	9.95E-01	7.82E-01	8.56E-01
Naproxen	-0.17(0.45)	0.04(0.09)	2.88(3.42)	0.21(0.46)	3.05(3.45)	2.85(3.42)	6.53E-01	3.80E-01	4.08E-01	9.95E-01	7.89E-01	7.35E-01
Nonadecanoic acid	-0.19(0.24)	0.14(0.12)	-0.44(0.14)	0.33(0.27)	-0.25(0.28)	-0.58(0.19)	2.32E-01	3.84E-01	2.87E-03	9.95E-01	7.92E-01	7.72E-02
Myristic acid	0.02(0.15)	-0.01(0.14)	0.22(0.19)	-0.03(0.21)	0.20(0.24)	0.23(0.24)	8.92E-01	3.97E-01	3.41E-01	9.95E-01	8.02E-01	6.79E-01
2-piperidinobenzonitrile NIST	0.04(0.16)	0.05(0.27)	0.21(0.13)	0.01(0.31)	0.18(0.21)	0.16(0.30)	9.64E-01	3.98E-01	5.81E-01	9.95E-01	8.02E-01	8.41E-01
1,5-anhydroglucitol	-3.35(3.35)	-1.38(0.39)	-0.52(0.07)	1.96(3.39)	2.82(3.35)	0.86(0.39)	5.64E-01	4.03E-01	3.26E-02	9.95E-01	8.02E-01	2.57E-01
4-hydroxyphenylacetic acid	-0.04(0.31)	0.36(0.31)	-0.30(0.10)	0.39(0.44)	-0.27(0.32)	-0.66(0.32)	3.70E-01	4.09E-01	4.66E-02	9.95E-01	8.02E-01	2.88E-01

2-hydroxyvaleric acid	-0.04(0.16)	0.23(0.20)	0.21(0.25)	0.26(0.27)	0.25(0.31)	-0.01(0.32)	3.28E-01	4.12E-01	9.70E-01	9.95E-01	8.02E-01	9.97E-01
Sucrose	0.01(0.16)	0.00(0.12)	0.48(0.55)	-0.01(0.20)	0.47(0.57)	0.48(0.56)	9.64E-01	4.13E-01	3.97E-01	9.95E-01	8.02E-01	7.33E-01
Cholesterol	-0.11(0.26)	-0.14(0.10)	-0.42(0.28)	-0.03(0.29)	-0.31(0.38)	-0.27(0.30)	9.10E-01	4.19E-01	3.59E-01	9.95E-01	8.02E-01	6.96E-01
Behenic acid	-0.02(0.18)	0.19(0.13)	-0.21(0.15)	0.21(0.23)	-0.19(0.24)	-0.40(0.21)	3.69E-01	4.20E-01	5.67E-02	9.95E-01	8.02E-01	3.21E-01
Nicotinic acid	-0.08(0.29)	-0.55(1.18)	-0.32(0.09)	-0.47(1.21)	-0.24(0.30)	0.23(1.18)	7.01E-01	4.24E-01	8.48E-01	9.95E-01	8.02E-01	9.54E-01
Hydroxylamine	0.06(0.21)	-0.07(0.13)	-0.14(0.15)	-0.13(0.25)	-0.21(0.26)	-0.07(0.20)	5.95E-01	4.26E-01	7.19E-01	9.95E-01	8.02E-01	9.11E-01
Hippuric acid	0.00(0.11)	-0.10(0.27)	-0.18(0.20)	-0.11(0.29)	-0.19(0.24)	-0.08(0.32)	7.11E-01	4.35E-01	8.14E-01	9.95E-01	8.12E-01	9.41E-01
Citric acid	-0.01(0.22)	-0.15(0.18)	-0.21(0.15)	-0.15(0.29)	-0.21(0.26)	-0.06(0.23)	6.15E-01	4.40E-01	7.94E-01	9.95E-01	8.14E-01	9.35E-01
Conduritol-beta-expoxide	-0.09(0.14)	-0.01(0.18)	-0.25(0.15)	0.08(0.23)	-0.16(0.21)	-0.24(0.23)	7.26E-01	4.43E-01	3.10E-01	9.95E-01	8.14E-01	6.60E-01
Glycerol-alpha-phosphate	0.12(0.14)	0.05(0.16)	0.28(0.16)	-0.06(0.22)	0.17(0.22)	0.23(0.23)	7.77E-01	4.48E-01	3.27E-01	9.95E-01	8.20E-01	6.63E-01
Leucine	-0.02(0.14)	-0.18(0.20)	-0.18(0.16)	-0.15(0.24)	-0.16(0.21)	0.00(0.26)	5.24E-01	4.57E-01	9.89E-01	9.95E-01	8.20E-01	9.97E-01
Cystine	0.24(0.34)	0.04(0.10)	0.52(0.19)	-0.19(0.35)	0.29(0.39)	0.48(0.22)	5.90E-01	4.62E-01	3.18E-02	9.95E-01	8.20E-01	2.57E-01
Glyceric acid	-0.03(0.09)	0.69(1.05)	-0.63(0.80)	0.73(1.05)	-0.60(0.81)	-1.32(1.34)	4.93E-01	4.64E-01	3.27E-01	9.95E-01	8.20E-01	6.63E-01
Erythritol	0.32(0.61)	-0.08(0.53)	-0.12(0.09)	-0.40(0.81)	-0.44(0.62)	-0.05(0.54)	6.26E-01	4.77E-01	9.33E-01	9.95E-01	8.29E-01	9.97E-01
N-acetylglycine NIST	-0.10(0.33)	0.17(0.19)	-0.34(0.12)	0.27(0.37)	-0.25(0.36)	-0.51(0.22)	4.78E-01	4.90E-01	2.27E-02	9.95E-01	8.36E-01	2.54E-01
Heptadecanoic acid	-0.02(0.16)	0.05(0.13)	-0.21(0.25)	0.07(0.20)	-0.19(0.29)	-0.26(0.28)	7.24E-01	5.16E-01	3.45E-01	9.95E-01	8.56E-01	6.80E-01
Tyrosine	-0.06(0.18)	0.15(0.17)	-0.20(0.14)	0.21(0.25)	-0.14(0.23)	-0.35(0.22)	4.01E-01	5.43E-01	1.08E-01	9.95E-01	8.69E-01	4.53E-01
Linoleic acid	-0.05(0.21)	-0.12(0.24)	0.10(0.11)	-0.08(0.32)	0.14(0.24)	0.22(0.26)	8.16E-01	5.46E-01	3.92E-01	9.95E-01	8.69E-01	7.32E-01
Threonic acid	-0.20(0.19)	0.13(0.15)	-0.34(0.15)	0.33(0.24)	-0.14(0.23)	-0.47(0.21)	1.87E-01	5.47E-01	3.33E-02	9.95E-01	8.69E-01	2.57E-01
Isocitric acid	-0.03(0.19)	-0.16(0.18)	0.11(0.14)	-0.13(0.26)	0.14(0.24)	0.27(0.22)	6.24E-01	5.48E-01	2.29E-01	9.95E-01	8.69E-01	5.84E-01
Tagatose	-0.02(0.10)	0.79(0.60)	0.42(0.75)	0.81(0.60)	0.44(0.75)	-0.37(0.95)	1.83E-01	5.63E-01	6.98E-01	9.95E-01	8.69E-01	9.07E-01
Pseudo uridine	-0.04(0.20)	-0.19(0.22)	-0.18(0.14)	-0.15(0.30)	-0.14(0.24)	0.01(0.26)	6.27E-01	5.66E-01	9.67E-01	9.95E-01	8.69E-01	9.97E-01
Serine	-0.31(0.22)	-0.19(0.21)	-0.45(0.13)	0.12(0.28)	-0.14(0.24)	-0.26(0.24)	6.60E-01	5.67E-01	2.76E-01	9.95E-01	8.69E-01	6.19E-01
Galactose	0.05(0.25)	-0.02(0.14)	-0.11(0.13)	-0.08(0.29)	-0.16(0.28)	-0.08(0.19)	7.89E-01	5.67E-01	6.62E-01	9.95E-01	8.69E-01	8.85E-01
Hexitol	-0.03(0.12)	0.14(0.18)	0.13(0.26)	0.17(0.22)	0.16(0.29)	-0.01(0.32)	4.37E-01	5.83E-01	9.79E-01	9.95E-01	8.69E-01	9.97E-01
Galactonic acid	0.04(0.34)	0.02(0.13)	0.24(0.14)	-0.02(0.36)	0.20(0.37)	0.22(0.18)	9.58E-01	5.83E-01	2.29E-01	9.95E-01	8.69E-01	5.84E-01
Phosphate	-0.15(0.19)	-0.11(0.19)	-0.28(0.14)	0.04(0.26)	-0.13(0.23)	-0.17(0.23)	8.67E-01	5.86E-01	4.62E-01	9.95E-01	8.69E-01	7.54E-01
Pipecolinic acid	-0.04(0.18)	0.08(0.17)	0.09(0.17)	0.12(0.25)	0.13(0.24)	0.01(0.24)	6.34E-01	5.90E-01	9.62E-01	9.95E-01	8.69E-01	9.97E-01
Oxamic acid	-0.02(0.09)	3.87(2.74)	-1.55(2.85)	3.89(2.75)	-1.53(2.85)	-5.42(4.05)	1.62E-01	5.92E-01	1.86E-01	9.95E-01	8.69E-01	5.49E-01

Ethanolamine	-0.22(0.30)	-0.05(0.31)	-0.05(0.11)	0.17(0.43)	0.17(0.32)	0.00(0.33)	6.95E-01	5.99E-01	9.98E-01	9.95E-01	8.74E-01	9.99E-01
Oxalic acid	-0.01(0.17)	0.17(0.15)	-0.13(0.16)	0.18(0.23)	-0.12(0.23)	-0.30(0.22)	4.35E-01	6.00E-01	1.83E-01	9.95E-01	8.74E-01	5.49E-01
Dehydroabietic acid	-0.21(0.26)	-0.11(0.10)	0.00(0.30)	0.09(0.28)	0.21(0.40)	0.11(0.31)	7.37E-01	6.05E-01	7.18E-01	9.95E-01	8.74E-01	9.11E-01
Methionine sulfoxide	-0.12(0.20)	0.07(0.15)	-0.24(0.14)	0.20(0.24)	-0.12(0.24)	-0.31(0.21)	4.12E-01	6.36E-01	1.35E-01	9.95E-01	8.87E-01	4.93E-01
Phenylethylamine	0.07(0.21)	-0.08(0.19)	-0.03(0.13)	-0.15(0.28)	-0.11(0.24)	0.04(0.23)	5.89E-01	6.52E-01	8.51E-01	9.95E-01	9.01E-01	9.54E-01
N-methylalanine	-0.09(0.28)	0.06(0.17)	0.05(0.12)	0.15(0.33)	0.14(0.31)	-0.01(0.21)	6.59E-01	6.62E-01	9.60E-01	9.95E-01	9.04E-01	9.97E-01
Stearic acid	-0.04(0.19)	0.02(0.12)	-0.18(0.25)	0.07(0.23)	-0.13(0.32)	-0.20(0.28)	7.69E-01	6.76E-01	4.73E-01	9.95E-01	9.04E-01	7.63E-01
Cellobiose minor	0.08(0.46)	-0.05(0.11)	-0.15(0.32)	-0.13(0.47)	-0.23(0.55)	-0.10(0.33)	7.79E-01	6.79E-01	7.75E-01	9.95E-01	9.04E-01	9.34E-01
Histidine	0.53(0.72)	0.05(0.13)	0.23(0.16)	-0.48(0.72)	-0.30(0.73)	0.18(0.20)	5.05E-01	6.83E-01	3.70E-01	9.95E-01	9.04E-01	7.07E-01
Fucose	0.12(0.65)	0.05(0.10)	-0.15(0.24)	-0.07(0.66)	-0.27(0.70)	-0.20(0.25)	9.13E-01	6.96E-01	4.33E-01	9.95E-01	9.04E-01	7.51E-01
Beta-alanine	-0.03(0.13)	0.12(0.24)	-0.11(0.15)	0.15(0.27)	-0.08(0.20)	-0.23(0.29)	5.79E-01	6.99E-01	4.28E-01	9.95E-01	9.04E-01	7.47E-01
Ornithine	-0.05(0.25)	-0.06(0.18)	-0.15(0.15)	-0.01(0.30)	-0.10(0.28)	-0.09(0.23)	9.71E-01	7.10E-01	6.88E-01	9.95E-01	9.04E-01	9.06E-01
2-deoxyisotetronic acid NIST	0.09(0.17)	-0.14(0.15)	0.17(0.16)	-0.23(0.23)	0.08(0.23)	0.31(0.22)	3.23E-01	7.16E-01	1.56E-01	9.95E-01	9.05E-01	5.22E-01
Arachidonic acid	-0.08(0.32)	-0.08(0.36)	0.04(0.10)	-0.01(0.47)	0.12(0.34)	0.13(0.37)	9.89E-01	7.21E-01	7.30E-01	9.96E-01	9.06E-01	9.11E-01
Deoxycholic acid	-0.12(0.30)	0.10(0.31)	-0.01(0.10)	0.22(0.43)	0.11(0.31)	-0.11(0.33)	6.08E-01	7.25E-01	7.40E-01	9.95E-01	9.08E-01	9.11E-01
Mannose	0.02(0.32)	0.38(0.22)	0.12(0.10)	0.37(0.39)	0.11(0.34)	-0.26(0.24)	3.49E-01	7.51E-01	2.91E-01	9.95E-01	9.26E-01	6.35E-01
Hexuronic acid	-0.03(0.18)	0.03(0.14)	0.06(0.21)	0.06(0.23)	0.09(0.28)	0.03(0.26)	8.01E-01	7.52E-01	9.07E-01	9.95E-01	9.26E-01	9.84E-01
Succinic acid	0.01(0.20)	0.00(0.20)	0.09(0.17)	-0.01(0.28)	0.08(0.26)	0.09(0.26)	9.63E-01	7.58E-01	7.17E-01	9.95E-01	9.30E-01	9.11E-01
3,6-anhydro-D-galactose	0.05(0.13)	0.15(0.22)	0.12(0.18)	0.10(0.26)	0.06(0.22)	-0.03(0.28)	7.13E-01	7.72E-01	9.11E-01	9.95E-01	9.41E-01	9.84E-01
2,3-dihydroxybutanoic acid NIST	-0.07(0.18)	0.03(0.18)	-0.01(0.14)	0.10(0.25)	0.06(0.22)	-0.04(0.22)	6.90E-01	7.74E-01	8.73E-01	9.95E-01	9.41E-01	9.62E-01
UDP-glucuronic acid	0.00(0.18)	0.06(0.13)	0.07(0.17)	0.06(0.23)	0.07(0.25)	0.01(0.22)	8.10E-01	7.81E-01	9.49E-01	9.95E-01	9.46E-01	9.97E-01
Pinitol	-0.08(0.47)	-0.01(0.16)	-0.22(0.17)	0.07(0.50)	-0.13(0.50)	-0.21(0.24)	8.87E-01	7.89E-01	3.87E-01	9.95E-01	9.49E-01	7.27E-01
Valine	-0.01(0.14)	-0.16(0.22)	-0.06(0.16)	-0.15(0.26)	-0.06(0.21)	0.10(0.27)	5.61E-01	7.90E-01	7.22E-01	9.95E-01	9.49E-01	9.11E-01
Lyxitol	-0.06(0.11)	-0.13(0.74)	0.00(0.19)	-0.07(0.75)	0.05(0.21)	0.13(0.76)	9.22E-01	8.00E-01	8.67E-01	9.95E-01	9.56E-01	9.62E-01
Quinic acid	0.09(0.15)	-0.11(0.26)	0.04(0.14)	-0.19(0.30)	-0.05(0.20)	0.14(0.30)	5.19E-01	8.01E-01	6.32E-01	9.95E-01	9.56E-01	8.69E-01
Isoleucine	0.03(0.17)	-0.08(0.18)	-0.02(0.14)	-0.12(0.25)	-0.05(0.23)	0.06(0.23)	6.38E-01	8.11E-01	7.86E-01	9.95E-01	9.62E-01	9.34E-01
Lysine	0.28(0.36)	0.06(0.14)	0.19(0.14)	-0.22(0.39)	-0.09(0.39)	0.13(0.20)	5.78E-01	8.22E-01	5.21E-01	9.95E-01	9.65E-01	7.99E-01
Xylitol	-0.11(0.33)	0.04(0.56)	-0.04(0.10)	0.14(0.65)	0.07(0.34)	-0.07(0.56)	8.23E-01	8.39E-01	8.95E-01	9.95E-01	9.72E-01	9.77E-01

N-acetylglutamate	-0.09(0.44)	0.03(0.10)	0.01(0.25)	0.12(0.45)	0.10(0.52)	-0.02(0.27)	7.97E-01	8.50E-01	9.43E-01	9.95E-01	9.72E-01	9.97E-01
Malic acid	-0.01(0.18)	-0.06(0.15)	0.03(0.16)	-0.05(0.24)	0.04(0.24)	0.09(0.23)	8.37E-01	8.51E-01	6.77E-01	9.95E-01	9.72E-01	9.02E-01
2-hydroxybutanoic acid	0.13(0.33)	0.14(0.22)	0.06(0.10)	0.01(0.41)	-0.06(0.35)	-0.08(0.25)	9.76E-01	8.57E-01	7.62E-01	9.95E-01	9.72E-01	9.24E-01
Cysteine-glycine	0.23(0.28)	0.02(0.14)	0.28(0.14)	-0.22(0.31)	0.05(0.31)	0.27(0.20)	4.91E-01	8.71E-01	1.85E-01	9.95E-01	9.75E-01	5.49E-01
Pyrrole-2-carboxylic acid	0.00(0.16)	-0.07(0.18)	0.03(0.16)	-0.06(0.24)	0.03(0.22)	0.10(0.24)	7.93E-01	8.77E-01	6.85E-01	9.95E-01	9.76E-01	9.06E-01
Uridine	-0.09(0.16)	-0.15(0.15)	-0.06(0.16)	-0.06(0.22)	0.03(0.23)	0.09(0.22)	7.99E-01	8.89E-01	6.89E-01	9.95E-01	9.80E-01	9.06E-01
Alanine	-0.02(0.19)	-0.18(0.21)	-0.05(0.12)	-0.15(0.28)	-0.03(0.23)	0.13(0.24)	5.83E-01	8.99E-01	5.99E-01	9.95E-01	9.83E-01	8.42E-01
Asparagine	-0.22(0.23)	0.09(0.17)	-0.19(0.12)	0.31(0.27)	0.03(0.25)	-0.28(0.21)	2.55E-01	9.06E-01	1.78E-01	9.95E-01	9.83E-01	5.45E-01
Levoglucosan	-0.07(0.25)	0.06(0.13)	-0.10(0.14)	0.13(0.29)	-0.03(0.29)	-0.17(0.20)	6.48E-01	9.10E-01	4.05E-01	9.95E-01	9.83E-01	7.35E-01
Tryptophan	0.75(1.24)	0.04(0.09)	0.89(0.88)	-0.71(1.25)	0.13(1.54)	0.84(0.89)	5.70E-01	9.31E-01	3.46E-01	9.95E-01	9.86E-01	6.80E-01
Isothreitol	-0.02(0.22)	-0.38(0.39)	0.00(0.11)	-0.36(0.45)	0.02(0.25)	0.38(0.41)	4.25E-01	9.36E-01	3.60E-01	9.95E-01	9.86E-01	6.96E-01
Arabitol	-0.01(0.17)	-0.05(0.24)	-0.02(0.13)	-0.04(0.30)	-0.01(0.22)	0.03(0.27)	8.91E-01	9.57E-01	9.11E-01	9.95E-01	9.90E-01	9.84E-01
Trans-4-hydroxyproline	0.07(0.12)	-0.30(0.24)	0.07(0.20)	-0.37(0.26)	-0.01(0.23)	0.36(0.31)	1.61E-01	9.69E-01	2.46E-01	9.95E-01	9.90E-01	6.07E-01
Acetaminophen	0.01(0.39)	-0.23(0.37)	-0.01(0.10)	-0.23(0.54)	-0.02(0.40)	0.22(0.38)	6.68E-01	9.70E-01	5.70E-01	9.95E-01	9.90E-01	8.41E-01
Taurine	0.11(0.64)	-0.50(0.52)	0.13(0.10)	-0.60(0.81)	0.02(0.65)	0.63(0.52)	4.62E-01	9.70E-01	2.32E-01	9.95E-01	9.90E-01	5.87E-01
Methanolphosphate	0.09(0.25)	0.11(0.18)	0.10(0.11)	0.02(0.31)	0.01(0.27)	-0.01(0.22)	9.51E-01	9.78E-01	9.58E-01	9.95E-01	9.90E-01	9.97E-01
2-deoxytetronic acid	0.02(0.24)	-0.15(0.20)	0.02(0.12)	-0.16(0.31)	0.01(0.27)	0.17(0.23)	5.99E-01	9.80E-01	4.61E-01	9.95E-01	9.90E-01	7.54E-01
Alloxanoic acid NIST	0.01(0.11)	0.00(0.28)	0.00(0.38)	-0.01(0.30)	0.00(0.39)	0.01(0.48)	9.69E-01	9.94E-01	9.85E-01	9.95E-01	9.97E-01	9.97E-01
**Unknown**												
915	-0.11(0.12)	0.00(0.34)	-7.56(1.57)	0.11(0.36)	-7.45(1.57)	-7.56(1.60)	7.56E-01	1.19E-05	1.32E-05	9.95E-01	4.47E-03	4.99E-03
7440	0.07(0.10)	-0.22(0.23)	-6.55(1.66)	-0.30(0.25)	-6.62(1.67)	-6.32(1.68)	2.34E-01	1.84E-04	3.60E-04	9.95E-01	2.81E-02	4.47E-02
7403	0.11(0.22)	0.04(0.20)	-3.70(0.95)	-0.07(0.30)	-3.81(0.99)	-3.74(0.97)	8.17E-01	2.98E-04	2.60E-04	9.95E-01	2.81E-02	4.47E-02
5419	-0.05(0.14)	0.05(0.16)	-0.98(0.26)	0.10(0.21)	-0.93(0.29)	-1.03(0.31)	6.32E-01	2.43E-03	1.52E-03	9.95E-01	1.15E-01	6.59E-02
135869	-0.01(0.14)	0.28(0.19)	0.71(0.21)	0.29(0.23)	0.73(0.26)	0.44(0.29)	2.18E-01	6.51E-03	1.39E-01	9.95E-01	1.53E-01	4.95E-01
1148	0.00(0.12)	0.08(0.13)	-1.30(0.44)	0.08(0.18)	-1.30(0.46)	-1.38(0.46)	6.38E-01	6.90E-03	3.88E-03	9.95E-01	1.53E-01	9.56E-02
132379	0.08(0.18)	0.14(0.16)	-0.54(0.13)	0.05(0.25)	-0.62(0.23)	-0.68(0.21)	8.30E-01	7.69E-03	1.78E-03	9.95E-01	1.61E-01	6.70E-02
5900	0.03(0.12)	-0.05(0.18)	-0.55(0.17)	-0.07(0.21)	-0.57(0.21)	-0.50(0.25)	7.43E-01	8.72E-03	4.54E-02	9.95E-01	1.71E-01	2.88E-01
93385	0.03(0.14)	0.07(0.20)	-0.59(0.19)	0.04(0.24)	-0.62(0.23)	-0.67(0.28)	8.54E-01	1.03E-02	1.85E-02	9.95E-01	1.81E-01	2.24E-01
100253	0.00(0.11)	0.04(0.20)	0.55(0.18)	0.04(0.23)	0.55(0.21)	0.51(0.26)	8.77E-01	1.14E-02	5.64E-02	9.95E-01	1.81E-01	3.21E-01

135260	0.02(0.12)	-0.34(0.18)	-0.53(0.17)	-0.36(0.22)	-0.55(0.21)	-0.19(0.25)	9.99E-02	1.15E-02	4.61E-01	9.95E-01	1.81E-01	7.54E-01
121890	0.08(0.12)	0.05(0.15)	-0.58(0.23)	-0.03(0.20)	-0.66(0.26)	-0.63(0.28)	8.81E-01	1.25E-02	2.60E-02	9.95E-01	1.87E-01	2.57E-01
42357	0.01(0.11)	0.01(0.19)	-0.51(0.18)	0.01(0.22)	-0.52(0.22)	-0.53(0.27)	9.79E-01	1.88E-02	5.24E-02	9.95E-01	2.24E-01	3.08E-01
17651	0.08(0.15)	0.14(0.20)	-0.37(0.13)	0.07(0.26)	-0.45(0.19)	-0.52(0.24)	7.89E-01	1.98E-02	3.41E-02	9.95E-01	2.26E-01	2.57E-01
592	-0.02(0.13)	-0.07(0.30)	-0.46(0.14)	-0.05(0.33)	-0.44(0.20)	-0.39(0.34)	8.73E-01	2.61E-02	2.48E-01	9.95E-01	2.90E-01	6.07E-01
4863	0.08(0.17)	0.08(0.29)	-0.36(0.11)	-0.00(0.33)	-0.44(0.20)	-0.44(0.31)	9.95E-01	3.10E-02	1.67E-01	9.98E-01	3.08E-01	5.34E-01
127277	-0.05(0.16)	0.22(0.20)	-0.50(0.15)	0.27(0.26)	-0.45(0.22)	-0.72(0.25)	2.99E-01	4.23E-02	5.02E-03	9.95E-01	4.00E-01	9.96E-02
1941	0.10(0.16)	0.23(0.19)	-0.33(0.13)	0.13(0.25)	-0.43(0.21)	-0.56(0.23)	5.99E-01	4.25E-02	1.53E-02	9.95E-01	4.00E-01	2.03E-01
13107	-0.01(0.22)	-0.30(0.12)	-0.56(0.16)	-0.29(0.25)	-0.55(0.27)	-0.26(0.20)	2.39E-01	4.79E-02	2.00E-01	9.95E-01	4.40E-01	5.49E-01
1700	-0.02(0.13)	0.10(0.18)	-0.51(0.22)	0.11(0.22)	-0.50(0.26)	-0.61(0.29)	6.14E-01	5.92E-02	4.03E-02	9.95E-01	4.92E-01	2.71E-01
17537	0.08(0.10)	-0.17(0.33)	-0.40(0.23)	-0.25(0.34)	-0.48(0.25)	-0.23(0.40)	4.59E-01	6.12E-02	5.74E-01	9.95E-01	4.92E-01	8.41E-01
1875	0.05(0.15)	-0.30(0.12)	-0.44(0.21)	-0.35(0.19)	-0.49(0.26)	-0.14(0.24)	7.01E-02	6.31E-02	5.64E-01	9.95E-01	4.92E-01	8.41E-01
1704	0.04(0.12)	0.07(0.21)	0.61(0.27)	0.03(0.25)	0.57(0.30)	0.54(0.33)	9.14E-01	6.36E-02	1.07E-01	9.95E-01	4.92E-01	4.53E-01
134336	-0.07(0.14)	0.06(0.24)	-0.42(0.12)	0.14(0.28)	-0.35(0.18)	-0.48(0.26)	6.25E-01	6.40E-02	7.29E-02	9.95E-01	4.92E-01	3.67E-01
448	0.05(0.19)	-0.12(0.15)	-0.40(0.15)	-0.17(0.24)	-0.46(0.24)	-0.29(0.22)	4.81E-01	6.61E-02	1.96E-01	9.95E-01	4.98E-01	5.49E-01
2061	-0.06(0.14)	-0.14(0.20)	-0.44(0.15)	-0.08(0.24)	-0.38(0.21)	-0.30(0.24)	7.29E-01	6.93E-02	2.27E-01	9.95E-01	5.12E-01	5.84E-01
41689	-0.07(0.21)	0.21(0.18)	0.36(0.13)	0.29(0.27)	0.43(0.24)	0.14(0.21)	2.90E-01	7.64E-02	5.00E-01	9.95E-01	5.23E-01	7.82E-01
16788	-0.03(0.13)	0.08(0.16)	-0.44(0.19)	0.12(0.21)	-0.41(0.23)	-0.53(0.25)	5.80E-01	8.25E-02	3.84E-02	9.95E-01	5.44E-01	2.65E-01
9392	0.14(0.19)	0.00(0.12)	0.58(0.17)	-0.14(0.23)	0.44(0.26)	0.58(0.21)	5.50E-01	9.14E-02	8.09E-03	9.95E-01	5.44E-01	1.29E-01
119023	0.05(0.37)	0.05(0.10)	-0.87(0.40)	-0.00(0.39)	-0.92(0.54)	-0.92(0.42)	9.98E-01	9.26E-02	3.09E-02	9.98E-01	5.44E-01	2.57E-01
64546	-0.08(0.21)	0.34(0.22)	0.32(0.10)	0.42(0.30)	0.40(0.24)	-0.02(0.24)	1.63E-01	9.36E-02	9.40E-01	9.95E-01	5.44E-01	9.97E-01
46292	-0.04(0.19)	-0.08(0.16)	-0.43(0.14)	-0.04(0.24)	-0.39(0.23)	-0.35(0.21)	8.62E-01	9.70E-02	9.94E-02	9.95E-01	5.44E-01	4.31E-01
5346	0.06(0.11)	0.05(0.28)	-0.34(0.22)	-0.01(0.30)	-0.40(0.24)	-0.39(0.35)	9.73E-01	1.04E-01	2.71E-01	9.95E-01	5.44E-01	6.19E-01
3029	0.01(0.18)	0.05(0.26)	0.36(0.11)	0.03(0.31)	0.34(0.21)	0.31(0.28)	9.15E-01	1.04E-01	2.69E-01	9.95E-01	5.44E-01	6.19E-01
2806	-0.06(0.11)	-0.41(0.71)	-0.92(0.51)	-0.35(0.72)	-0.86(0.52)	-0.51(0.89)	6.30E-01	1.06E-01	5.67E-01	9.95E-01	5.44E-01	8.41E-01
98027	0.03(0.38)	0.01(0.13)	-0.65(0.18)	-0.03(0.41)	-0.69(0.43)	-0.66(0.22)	9.51E-01	1.11E-01	4.24E-03	9.95E-01	5.44E-01	9.56E-02
4937	0.17(0.17)	0.14(0.22)	-0.19(0.14)	-0.03(0.28)	-0.36(0.22)	-0.33(0.26)	9.11E-01	1.13E-01	2.19E-01	9.95E-01	5.44E-01	5.73E-01
6646	-0.14(0.25)	0.38(0.21)	0.29(0.10)	0.52(0.34)	0.43(0.27)	-0.09(0.24)	1.25E-01	1.15E-01	6.97E-01	9.95E-01	5.44E-01	9.07E-01
4948	-0.03(0.14)	0.40(0.42)	0.26(0.12)	0.43(0.44)	0.29(0.18)	-0.14(0.43)	3.31E-01	1.15E-01	7.40E-01	9.95E-01	5.44E-01	9.11E-01
3465	-0.06(0.14)	0.22(0.21)	0.28(0.16)	0.27(0.25)	0.34(0.21)	0.07(0.26)	2.87E-01	1.17E-01	8.06E-01	9.95E-01	5.44E-01	9.41E-01

4942	0.28(0.34)	0.00(0.10)	-0.48(0.34)	-0.29(0.36)	-0.77(0.49)	-0.48(0.36)	4.27E-01	1.18E-01	1.80E-01	9.95E-01	5.44E-01	5.47E-01
4945	0.01(0.11)	0.12(0.25)	-0.41(0.24)	0.11(0.27)	-0.42(0.26)	-0.53(0.35)	6.92E-01	1.21E-01	1.34E-01	9.95E-01	5.44E-01	4.93E-01
61	0.03(0.31)	-0.01(0.21)	-0.48(0.09)	-0.03(0.38)	-0.51(0.33)	-0.47(0.22)	9.28E-01	1.23E-01	3.87E-02	9.95E-01	5.44E-01	2.65E-01
6331	0.06(0.20)	0.09(0.18)	-0.33(0.14)	0.03(0.28)	-0.39(0.25)	-0.41(0.23)	9.25E-01	1.23E-01	8.25E-02	9.95E-01	5.44E-01	3.89E-01
17453	0.10(0.47)	0.01(0.10)	-0.85(0.38)	-0.09(0.48)	-0.95(0.61)	-0.86(0.40)	8.53E-01	1.24E-01	3.40E-02	9.95E-01	5.44E-01	2.57E-01
91	-0.08(0.16)	0.05(0.13)	-0.46(0.19)	0.13(0.21)	-0.38(0.25)	-0.52(0.22)	5.15E-01	1.26E-01	2.50E-02	9.95E-01	5.44E-01	2.57E-01
473	-0.06(0.12)	0.06(0.61)	0.27(0.20)	0.12(0.61)	0.33(0.22)	0.21(0.64)	8.48E-01	1.28E-01	7.37E-01	9.95E-01	5.47E-01	9.11E-01
41683	-0.05(0.14)	0.40(0.32)	0.26(0.15)	0.45(0.35)	0.31(0.20)	-0.14(0.35)	2.02E-01	1.32E-01	6.91E-01	9.95E-01	5.59E-01	9.06E-01
95550	-0.10(0.15)	0.13(0.19)	-0.40(0.13)	0.23(0.24)	-0.30(0.20)	-0.54(0.23)	3.30E-01	1.35E-01	2.54E-02	9.95E-01	5.60E-01	2.57E-01
47	-0.06(0.18)	0.07(0.32)	-0.36(0.11)	0.13(0.35)	-0.30(0.20)	-0.43(0.33)	7.19E-01	1.39E-01	1.96E-01	9.95E-01	5.64E-01	5.49E-01
135610	-0.24(0.20)	0.47(0.35)	0.09(0.11)	0.70(0.41)	0.32(0.22)	-0.38(0.37)	9.17E-02	1.48E-01	3.05E-01	9.95E-01	5.93E-01	6.55E-01
111162	0.00(0.14)	-0.29(0.36)	-0.88(0.59)	-0.29(0.39)	-0.88(0.61)	-0.59(0.70)	4.54E-01	1.53E-01	4.02E-01	9.95E-01	6.01E-01	7.35E-01
4926	0.07(0.10)	0.15(0.38)	0.39(0.20)	0.08(0.39)	0.32(0.23)	0.24(0.43)	8.31E-01	1.60E-01	5.86E-01	9.95E-01	6.19E-01	8.41E-01
1029	-0.03(0.16)	0.13(0.18)	-0.34(0.15)	0.16(0.23)	-0.31(0.22)	-0.47(0.23)	4.98E-01	1.63E-01	4.58E-02	9.95E-01	6.19E-01	2.88E-01
17263	0.00(0.13)	0.22(0.22)	-0.30(0.17)	0.22(0.26)	-0.30(0.21)	-0.52(0.28)	4.08E-01	1.64E-01	7.12E-02	9.95E-01	6.19E-01	3.67E-01
117141	-0.03(0.21)	0.25(0.42)	-0.35(0.09)	0.28(0.48)	-0.32(0.23)	-0.60(0.43)	5.53E-01	1.69E-01	1.64E-01	9.95E-01	6.20E-01	5.34E-01
2001	0.00(0.10)	0.47(0.54)	0.52(0.36)	0.48(0.55)	0.52(0.38)	0.04(0.65)	3.89E-01	1.73E-01	9.49E-01	9.95E-01	6.20E-01	9.97E-01
5483	0.00(0.16)	-0.01(0.15)	-0.31(0.17)	-0.01(0.22)	-0.31(0.23)	-0.30(0.23)	9.56E-01	1.76E-01	1.95E-01	9.95E-01	6.20E-01	5.49E-01
31357	-0.07(0.16)	-0.05(0.13)	0.28(0.20)	0.02(0.20)	0.34(0.25)	0.32(0.23)	9.19E-01	1.77E-01	1.71E-01	9.95E-01	6.20E-01	5.38E-01
8270	0.06(0.12)	0.04(0.16)	0.40(0.22)	-0.02(0.20)	0.34(0.25)	0.36(0.27)	9.14E-01	1.78E-01	1.89E-01	9.95E-01	6.20E-01	5.49E-01
3258	0.08(0.14)	0.07(0.18)	-0.20(0.16)	-0.01(0.23)	-0.28(0.21)	-0.27(0.24)	9.50E-01	1.84E-01	2.73E-01	9.95E-01	6.20E-01	6.19E-01
3232	0.44(0.47)	-0.01(0.10)	-0.30(0.27)	-0.45(0.48)	-0.73(0.55)	-0.28(0.29)	3.50E-01	1.85E-01	3.27E-01	9.95E-01	6.20E-01	6.63E-01
257	-0.07(0.11)	-0.31(0.30)	-0.35(0.18)	-0.24(0.32)	-0.27(0.21)	-0.03(0.35)	4.55E-01	1.98E-01	9.23E-01	9.95E-01	6.37E-01	9.94E-01
62	-0.05(0.20)	0.06(0.20)	-0.35(0.11)	0.11(0.28)	-0.30(0.23)	-0.41(0.22)	6.92E-01	2.04E-01	6.96E-02	9.95E-01	6.37E-01	3.67E-01
1872	0.05(0.16)	0.15(0.22)	-0.21(0.13)	0.10(0.27)	-0.26(0.21)	-0.36(0.26)	7.12E-01	2.04E-01	1.68E-01	9.95E-01	6.37E-01	5.34E-01
41808	-0.08(0.14)	0.27(0.21)	0.22(0.19)	0.35(0.26)	0.30(0.23)	-0.05(0.29)	1.76E-01	2.05E-01	8.64E-01	9.95E-01	6.37E-01	9.62E-01
7718	-0.10(0.14)	0.18(0.14)	-0.38(0.17)	0.28(0.20)	-0.28(0.22)	-0.56(0.22)	1.63E-01	2.12E-01	1.56E-02	9.95E-01	6.42E-01	2.03E-01
6580	-0.15(0.25)	0.06(0.12)	0.24(0.18)	0.20(0.27)	0.38(0.30)	0.18(0.21)	4.54E-01	2.12E-01	3.96E-01	9.95E-01	6.42E-01	7.33E-01
120562	0.05(0.17)	-0.01(0.14)	-0.26(0.18)	-0.06(0.22)	-0.32(0.25)	-0.25(0.23)	7.76E-01	2.13E-01	2.66E-01	9.95E-01	6.42E-01	6.19E-01
4929	-0.09(0.15)	-0.04(0.13)	0.48(0.44)	0.04(0.19)	0.57(0.47)	0.52(0.45)	8.22E-01	2.30E-01	2.52E-01	9.95E-01	6.76E-01	6.11E-01

16550	-0.08(0.15)	-0.24(0.21)	-0.33(0.13)	-0.15(0.26)	-0.24(0.20)	-0.09(0.24)	5.53E-01	2.35E-01	7.21E-01	9.95E-01	6.84E-01	9.11E-01
131620	-0.09(0.20)	0.26(0.20)	-0.36(0.11)	0.35(0.28)	-0.27(0.23)	-0.62(0.22)	2.14E-01	2.36E-01	7.40E-03	9.95E-01	6.84E-01	1.29E-01
453	-0.38(0.63)	-0.01(0.09)	0.60(0.54)	0.37(0.63)	0.98(0.83)	0.61(0.54)	5.56E-01	2.40E-01	2.68E-01	9.95E-01	6.87E-01	6.19E-01
14509	-0.08(0.16)	0.08(0.23)	-0.33(0.14)	0.16(0.28)	-0.25(0.21)	-0.41(0.27)	5.66E-01	2.41E-01	1.39E-01	9.95E-01	6.87E-01	4.95E-01
4985	0.01(0.10)	-0.86(1.47)	-4.61(3.96)	-0.87(1.47)	-4.62(3.96)	-3.75(4.21)	5.56E-01	2.47E-01	3.76E-01	9.95E-01	6.99E-01	7.12E-01
5085	-0.02(0.10)	0.17(0.48)	-0.58(0.48)	0.19(0.49)	-0.56(0.48)	-0.76(0.68)	6.96E-01	2.49E-01	2.69E-01	9.95E-01	6.99E-01	6.19E-01
7053	0.17(0.21)	0.00(0.25)	-0.11(0.13)	-0.17(0.32)	-0.28(0.25)	-0.11(0.28)	5.92E-01	2.53E-01	6.92E-01	9.95E-01	7.02E-01	9.06E-01
16792	0.09(0.19)	0.11(0.15)	-0.19(0.15)	0.03(0.25)	-0.28(0.25)	-0.30(0.22)	9.19E-01	2.63E-01	1.69E-01	9.95E-01	7.13E-01	5.34E-01
134	0.28(0.35)	0.11(0.10)	0.70(0.14)	-0.17(0.36)	0.42(0.38)	0.59(0.18)	6.43E-01	2.68E-01	1.38E-03	9.95E-01	7.19E-01	6.59E-02
1709	0.10(0.24)	0.36(0.18)	-0.18(0.11)	0.25(0.30)	-0.29(0.26)	-0.54(0.22)	4.00E-01	2.69E-01	1.52E-02	9.95E-01	7.19E-01	2.03E-01
135889	-2.22(2.54)	0.07(0.16)	0.59(0.20)	2.28(2.55)	2.81(2.57)	0.53(0.26)	3.74E-01	2.78E-01	4.28E-02	9.95E-01	7.22E-01	2.78E-01
490	-0.01(0.15)	0.11(0.45)	-0.21(0.12)	0.12(0.47)	-0.20(0.19)	-0.32(0.47)	8.07E-01	2.79E-01	4.95E-01	9.95E-01	7.22E-01	7.82E-01
106634	0.01(0.13)	-0.03(0.21)	-0.21(0.16)	-0.04(0.25)	-0.23(0.21)	-0.19(0.27)	8.67E-01	2.81E-01	4.86E-01	9.95E-01	7.22E-01	7.73E-01
41708	-0.03(0.18)	0.06(0.17)	0.20(0.13)	0.09(0.24)	0.23(0.22)	0.14(0.22)	7.03E-01	2.97E-01	5.18E-01	9.95E-01	7.22E-01	7.99E-01
160	0.04(0.12)	0.31(0.33)	-0.37(0.38)	0.27(0.34)	-0.41(0.39)	-0.68(0.49)	4.36E-01	2.98E-01	1.74E-01	9.95E-01	7.22E-01	5.42E-01
32247	0.00(0.17)	-0.15(0.21)	-0.22(0.13)	-0.16(0.28)	-0.22(0.21)	-0.07(0.25)	5.75E-01	2.98E-01	7.93E-01	9.95E-01	7.22E-01	9.35E-01
4933	0.16(0.20)	0.07(0.12)	-0.14(0.20)	-0.10(0.24)	-0.30(0.29)	-0.20(0.23)	6.81E-01	2.98E-01	3.88E-01	9.95E-01	7.22E-01	7.27E-01
21666	-0.01(0.10)	0.29(0.24)	0.31(0.28)	0.30(0.26)	0.31(0.30)	0.01(0.37)	2.57E-01	3.00E-01	9.75E-01	9.95E-01	7.22E-01	9.97E-01
21513	0.05(0.10)	0.29(0.26)	0.38(0.29)	0.23(0.27)	0.32(0.31)	0.09(0.39)	3.90E-01	3.01E-01	8.22E-01	9.95E-01	7.22E-01	9.41E-01
2039	0.16(0.21)	0.23(0.37)	-0.09(0.11)	0.07(0.44)	-0.25(0.24)	-0.32(0.38)	8.74E-01	3.04E-01	4.06E-01	9.95E-01	7.22E-01	7.35E-01
6066	0.05(0.24)	0.13(0.16)	-0.23(0.12)	0.08(0.29)	-0.28(0.27)	-0.36(0.20)	7.77E-01	3.07E-01	7.74E-02	9.95E-01	7.23E-01	3.73E-01
9320	0.03(0.10)	-0.25(0.28)	0.29(0.24)	-0.28(0.30)	0.27(0.26)	0.54(0.38)	3.61E-01	3.16E-01	1.52E-01	9.95E-01	7.28E-01	5.15E-01
26918	0.04(0.17)	0.01(0.14)	0.28(0.16)	-0.03(0.22)	0.24(0.24)	0.27(0.21)	8.77E-01	3.16E-01	1.94E-01	9.95E-01	7.28E-01	5.49E-01
6802	0.00(0.21)	-0.10(0.23)	0.25(0.11)	-0.10(0.31)	0.24(0.24)	0.34(0.26)	7.49E-01	3.18E-01	2.02E-01	9.95E-01	7.28E-01	5.52E-01
93947	0.00(0.23)	0.06(0.12)	-0.27(0.16)	0.06(0.25)	-0.28(0.27)	-0.33(0.20)	8.27E-01	3.20E-01	1.01E-01	9.95E-01	7.28E-01	4.32E-01
130396	0.26(0.88)	0.06(0.09)	-0.93(0.77)	-0.20(0.89)	-1.19(1.19)	-0.98(0.78)	8.20E-01	3.21E-01	2.11E-01	9.95E-01	7.28E-01	5.71E-01
2900	-0.18(0.20)	0.38(0.33)	0.11(0.21)	0.57(0.40)	0.29(0.30)	-0.27(0.39)	1.65E-01	3.30E-01	4.85E-01	9.95E-01	7.40E-01	7.73E-01
6864	0.05(0.22)	0.21(0.18)	-0.21(0.15)	0.16(0.28)	-0.26(0.26)	-0.42(0.23)	5.77E-01	3.32E-01	7.81E-02	9.95E-01	7.41E-01	3.73E-01
112487	-0.03(0.29)	-0.18(0.25)	-0.32(0.10)	-0.15(0.39)	-0.29(0.30)	-0.14(0.26)	7.01E-01	3.40E-01	5.92E-01	9.95E-01	7.49E-01	8.42E-01
1981	0.12(0.14)	0.01(0.29)	-0.07(0.14)	-0.11(0.32)	-0.19(0.19)	-0.07(0.32)	7.26E-01	3.43E-01	8.21E-01	9.95E-01	7.49E-01	9.41E-01

17068	-0.25(0.22)	0.13(0.22)	-0.47(0.08)	0.38(0.31)	-0.22(0.23)	-0.60(0.24)	2.33E-01	3.46E-01	1.46E-02	9.95E-01	7.49E-01	2.03E-01
120526	-0.05(0.25)	0.24(0.22)	-0.30(0.11)	0.28(0.33)	-0.26(0.27)	-0.54(0.25)	3.99E-01	3.48E-01	3.25E-02	9.95E-01	7.49E-01	2.57E-01
122151	-0.02(0.26)	0.05(0.19)	-0.27(0.12)	0.07(0.32)	-0.25(0.28)	-0.32(0.22)	8.32E-01	3.71E-01	1.42E-01	9.95E-01	7.82E-01	4.96E-01
6115	0.09(0.42)	0.05(0.11)	0.51(0.19)	-0.04(0.44)	0.42(0.47)	0.46(0.22)	9.26E-01	3.73E-01	3.83E-02	9.95E-01	7.82E-01	2.65E-01
160543	0.15(0.20)	0.06(0.12)	-0.12(0.23)	-0.09(0.23)	-0.27(0.30)	-0.18(0.26)	7.03E-01	3.81E-01	4.98E-01	9.95E-01	7.89E-01	7.82E-01
6538	0.26(0.22)	0.13(0.14)	0.02(0.18)	-0.13(0.26)	-0.24(0.29)	-0.12(0.22)	6.36E-01	3.97E-01	5.98E-01	9.95E-01	8.02E-01	8.42E-01
20903	-0.10(0.16)	0.10(0.16)	-0.30(0.19)	0.20(0.22)	-0.20(0.24)	-0.41(0.24)	3.69E-01	4.03E-01	9.83E-02	9.95E-01	8.02E-01	4.31E-01
109423	0.03(0.19)	-0.30(0.10)	0.30(0.26)	-0.33(0.21)	0.26(0.32)	0.59(0.28)	1.22E-01	4.13E-01	3.75E-02	9.95E-01	8.02E-01	2.65E-01
117171	-0.08(0.17)	0.20(0.19)	-0.25(0.13)	0.28(0.25)	-0.17(0.21)	-0.45(0.23)	2.72E-01	4.14E-01	5.71E-02	9.95E-01	8.02E-01	3.21E-01
1064	0.01(0.18)	0.18(0.36)	0.18(0.11)	0.17(0.40)	0.17(0.21)	0.00(0.38)	6.81E-01	4.19E-01	9.96E-01	9.95E-01	8.02E-01	9.99E-01
107960	0.10(0.30)	0.14(0.21)	-0.17(0.11)	0.04(0.36)	-0.26(0.32)	-0.31(0.25)	9.07E-01	4.19E-01	2.19E-01	9.95E-01	8.02E-01	5.73E-01
126397	-0.11(0.16)	-0.05(0.22)	-0.28(0.13)	0.06(0.26)	-0.17(0.21)	-0.23(0.25)	8.27E-01	4.27E-01	3.72E-01	9.95E-01	8.02E-01	7.07E-01
4871	-0.09(0.22)	0.04(0.21)	0.11(0.11)	0.13(0.30)	0.20(0.25)	0.07(0.24)	6.81E-01	4.28E-01	7.61E-01	9.95E-01	8.02E-01	9.24E-01
160542	0.12(0.12)	0.13(0.25)	-0.05(0.19)	0.01(0.28)	-0.17(0.21)	-0.18(0.31)	9.63E-01	4.42E-01	5.61E-01	9.95E-01	8.14E-01	8.41E-01
4983	-0.08(0.27)	0.20(0.18)	0.14(0.12)	0.29(0.33)	0.23(0.30)	-0.06(0.21)	3.96E-01	4.51E-01	7.82E-01	9.95E-01	8.20E-01	9.34E-01
4976	-0.02(0.11)	0.13(0.21)	-0.22(0.25)	0.15(0.24)	-0.20(0.27)	-0.35(0.32)	5.37E-01	4.54E-01	2.78E-01	9.95E-01	8.20E-01	6.19E-01
121002	-0.11(0.21)	0.11(0.19)	0.07(0.13)	0.21(0.28)	0.18(0.24)	-0.03(0.23)	4.56E-01	4.60E-01	8.96E-01	9.95E-01	8.20E-01	9.77E-01
479	-0.01(0.26)	0.00(0.12)	-0.24(0.16)	0.01(0.29)	-0.22(0.30)	-0.24(0.20)	9.67E-01	4.62E-01	2.53E-01	9.95E-01	8.20E-01	6.11E-01
87769	0.05(0.11)	-0.11(0.33)	-0.13(0.22)	-0.16(0.35)	-0.18(0.24)	-0.02(0.41)	6.55E-01	4.65E-01	9.57E-01	9.95E-01	8.20E-01	9.97E-01
145494	0.00(0.14)	-0.11(0.14)	-0.20(0.23)	-0.10(0.21)	-0.20(0.27)	-0.09(0.27)	6.16E-01	4.70E-01	7.27E-01	9.95E-01	8.24E-01	9.11E-01
3329	-0.06(0.19)	0.02(0.16)	-0.25(0.18)	0.09(0.25)	-0.19(0.26)	-0.27(0.24)	7.33E-01	4.75E-01	2.60E-01	9.95E-01	8.29E-01	6.19E-01
18488	0.06(0.18)	0.09(0.16)	-0.11(0.14)	0.03(0.24)	-0.17(0.23)	-0.20(0.21)	8.99E-01	4.82E-01	3.59E-01	9.95E-01	8.33E-01	6.96E-01
5930	-0.11(0.16)	-0.13(0.22)	-0.25(0.12)	-0.03(0.27)	-0.14(0.20)	-0.12(0.25)	9.24E-01	4.85E-01	6.49E-01	9.95E-01	8.33E-01	8.80E-01
4981	0.03(0.12)	0.07(0.20)	-0.20(0.30)	0.04(0.23)	-0.22(0.32)	-0.26(0.36)	8.65E-01	4.86E-01	4.61E-01	9.95E-01	8.33E-01	7.54E-01
573	-0.04(0.21)	-0.02(0.18)	0.13(0.13)	0.03(0.28)	0.17(0.25)	0.15(0.23)	9.25E-01	4.94E-01	5.27E-01	9.95E-01	8.39E-01	7.99E-01
5288	-0.26(0.42)	0.09(0.35)	0.03(0.10)	0.34(0.56)	0.29(0.43)	-0.05(0.37)	5.46E-01	4.98E-01	8.90E-01	9.95E-01	8.39E-01	9.76E-01
4922	-0.14(0.20)	-0.12(0.19)	-0.44(0.39)	0.02(0.27)	-0.30(0.44)	-0.32(0.44)	9.39E-01	5.00E-01	4.63E-01	9.95E-01	8.39E-01	7.54E-01
133943	0.14(0.18)	0.09(0.12)	-0.05(0.24)	-0.05(0.21)	-0.20(0.29)	-0.15(0.28)	8.15E-01	5.01E-01	5.92E-01	9.95E-01	8.39E-01	8.42E-01
469	0.15(0.19)	0.17(0.20)	0.00(0.12)	0.03(0.28)	-0.15(0.22)	-0.17(0.23)	9.24E-01	5.15E-01	4.63E-01	9.95E-01	8.56E-01	7.54E-01
91421	-0.11(0.26)	0.01(0.15)	0.08(0.13)	0.11(0.30)	0.19(0.30)	0.08(0.21)	7.03E-01	5.22E-01	7.05E-01	9.95E-01	8.62E-01	9.11E-01

87756	-0.11(0.16)	-0.08(0.21)	-0.24(0.13)	0.03(0.26)	-0.13(0.21)	-0.16(0.25)	9.09E-01	5.41E-01	5.28E-01	9.95E-01	8.69E-01	7.99E-01
4546	0.02(0.14)	0.02(0.24)	0.23(0.33)	-0.00(0.28)	0.22(0.35)	0.21(0.40)	9.91E-01	5.43E-01	5.97E-01	9.96E-01	8.69E-01	8.42E-01
892	0.08(0.58)	-0.04(0.10)	0.49(0.36)	-0.12(0.58)	0.41(0.67)	0.53(0.37)	8.33E-01	5.43E-01	1.58E-01	9.95E-01	8.69E-01	5.22E-01
109387	0.07(0.26)	-0.01(0.28)	0.24(0.10)	-0.08(0.38)	0.16(0.28)	0.24(0.30)	8.40E-01	5.53E-01	4.19E-01	9.95E-01	8.69E-01	7.41E-01
119066	0.02(0.20)	0.02(0.15)	0.19(0.20)	0.01(0.25)	0.17(0.29)	0.16(0.25)	9.79E-01	5.63E-01	5.24E-01	9.95E-01	8.69E-01	7.99E-01
31559	-0.01(0.33)	0.01(0.13)	-0.21(0.13)	0.01(0.35)	-0.21(0.36)	-0.22(0.19)	9.70E-01	5.63E-01	2.40E-01	9.95E-01	8.69E-01	6.00E-01
4928	-0.06(0.20)	-0.09(0.21)	-0.77(1.21)	-0.03(0.29)	-0.70(1.23)	-0.68(1.23)	9.31E-01	5.68E-01	5.84E-01	9.95E-01	8.69E-01	8.41E-01
26916	0.11(0.21)	-0.18(0.14)	-0.04(0.16)	-0.29(0.26)	-0.15(0.26)	0.14(0.21)	2.65E-01	5.68E-01	5.10E-01	9.95E-01	8.69E-01	7.94E-01
8607	-0.04(0.15)	0.08(0.15)	-0.17(0.18)	0.12(0.22)	-0.13(0.23)	-0.24(0.24)	5.84E-01	5.79E-01	3.06E-01	9.95E-01	8.69E-01	6.55E-01
62433	-0.08(0.14)	-0.15(0.18)	-0.20(0.16)	-0.08(0.23)	-0.12(0.21)	-0.04(0.24)	7.43E-01	5.79E-01	8.52E-01	9.95E-01	8.69E-01	9.54E-01
135619	0.04(0.11)	-0.17(0.24)	0.22(0.30)	-0.20(0.26)	0.18(0.32)	0.38(0.38)	4.37E-01	5.80E-01	3.24E-01	9.95E-01	8.69E-01	6.63E-01
4898	-0.10(0.19)	0.10(0.17)	-0.23(0.13)	0.20(0.26)	-0.13(0.23)	-0.33(0.21)	4.44E-01	5.84E-01	1.30E-01	9.95E-01	8.69E-01	4.93E-01
4577	0.19(0.49)	-0.29(0.30)	-0.09(0.10)	-0.48(0.58)	-0.27(0.50)	0.20(0.32)	4.14E-01	5.89E-01	5.23E-01	9.95E-01	8.69E-01	7.99E-01
41682	0.04(0.13)	0.05(0.16)	0.19(0.25)	0.01(0.20)	0.15(0.28)	0.14(0.30)	9.59E-01	5.91E-01	6.36E-01	9.95E-01	8.69E-01	8.69E-01
1725	0.05(0.12)	0.18(0.18)	0.19(0.24)	0.13(0.21)	0.14(0.26)	0.01(0.29)	5.32E-01	5.93E-01	9.83E-01	9.95E-01	8.69E-01	9.97E-01
68	-0.11(0.16)	0.01(0.20)	-0.21(0.13)	0.12(0.26)	-0.10(0.20)	-0.23(0.24)	6.38E-01	6.09E-01	3.40E-01	9.95E-01	8.77E-01	6.79E-01
39	-0.12(0.20)	0.01(0.27)	-0.23(0.12)	0.14(0.34)	-0.11(0.23)	-0.25(0.30)	6.88E-01	6.29E-01	4.10E-01	9.95E-01	8.87E-01	7.35E-01
7402	0.13(0.16)	0.02(0.18)	-0.22(0.70)	-0.10(0.23)	-0.34(0.70)	-0.24(0.72)	6.64E-01	6.30E-01	7.40E-01	9.95E-01	8.87E-01	9.11E-01
4941	0.00(0.22)	0.18(0.17)	-0.13(0.17)	0.18(0.28)	-0.13(0.28)	-0.31(0.24)	5.20E-01	6.30E-01	1.91E-01	9.95E-01	8.87E-01	5.49E-01
6579	-0.11(0.18)	0.08(0.18)	0.00(0.14)	0.18(0.25)	0.11(0.23)	-0.07(0.22)	4.70E-01	6.31E-01	7.42E-01	9.95E-01	8.87E-01	9.11E-01
4943	0.03(0.17)	0.10(0.19)	-0.07(0.13)	0.07(0.25)	-0.10(0.22)	-0.17(0.22)	7.96E-01	6.33E-01	4.51E-01	9.95E-01	8.87E-01	7.54E-01
4543	0.00(0.12)	0.06(0.16)	0.18(0.36)	0.06(0.20)	0.18(0.38)	0.13(0.39)	7.78E-01	6.36E-01	7.50E-01	9.95E-01	8.87E-01	9.16E-01
41697	-0.04(0.15)	0.13(0.16)	0.06(0.17)	0.17(0.22)	0.11(0.23)	-0.06(0.23)	4.44E-01	6.37E-01	7.90E-01	9.95E-01	8.87E-01	9.35E-01
1878	-0.11(0.17)	-0.05(0.14)	-0.22(0.16)	0.06(0.22)	-0.11(0.24)	-0.17(0.22)	7.80E-01	6.52E-01	4.44E-01	9.95E-01	9.01E-01	7.54E-01
160313	0.02(0.16)	-0.30(0.48)	0.11(0.13)	-0.32(0.50)	0.09(0.20)	0.41(0.49)	5.23E-01	6.64E-01	4.08E-01	9.95E-01	9.04E-01	7.35E-01
47170	0.03(0.37)	0.01(0.11)	0.21(0.17)	-0.02(0.39)	0.17(0.41)	0.20(0.20)	9.50E-01	6.73E-01	3.25E-01	9.95E-01	9.04E-01	6.63E-01
159824	-0.11(0.15)	0.11(0.14)	0.00(0.21)	0.22(0.20)	0.11(0.26)	-0.12(0.26)	2.59E-01	6.77E-01	6.58E-01	9.95E-01	9.04E-01	8.85E-01
54	-0.08(0.17)	-0.07(0.15)	-0.18(0.16)	0.01(0.22)	-0.10(0.23)	-0.10(0.21)	9.72E-01	6.77E-01	6.30E-01	9.95E-01	9.04E-01	8.69E-01
87710	0.31(0.63)	0.03(0.12)	0.04(0.18)	-0.28(0.64)	-0.27(0.65)	0.01(0.21)	6.66E-01	6.78E-01	9.73E-01	9.95E-01	9.04E-01	9.97E-01
132414	0.25(0.58)	0.04(0.10)	-0.10(0.64)	-0.22(0.58)	-0.36(0.86)	-0.14(0.65)	7.13E-01	6.79E-01	8.25E-01	9.95E-01	9.04E-01	9.43E-01

16561	-0.05(0.21)	-0.14(0.20)	0.05(0.13)	-0.09(0.29)	0.10(0.24)	0.19(0.23)	7.57E-01	6.87E-01	4.21E-01	9.95E-01	9.04E-01	7.41E-01
6353	0.02(0.11)	0.21(0.29)	-0.09(0.26)	0.19(0.31)	-0.11(0.28)	-0.30(0.39)	5.43E-01	6.98E-01	4.39E-01	9.95E-01	9.04E-01	7.54E-01
5990	0.07(0.17)	-0.02(0.19)	-0.02(0.14)	-0.09(0.26)	-0.09(0.22)	0.00(0.23)	7.40E-01	6.99E-01	9.99E-01	9.95E-01	9.04E-01	9.99E-01
4600	-0.14(0.18)	0.06(0.15)	-0.24(0.21)	0.19(0.24)	-0.10(0.25)	-0.29(0.26)	4.20E-01	7.02E-01	2.75E-01	9.95E-01	9.04E-01	6.19E-01
2936	0.00(0.42)	0.19(0.33)	0.17(0.10)	0.19(0.54)	0.17(0.44)	-0.02(0.34)	7.29E-01	7.03E-01	9.52E-01	9.95E-01	9.04E-01	9.97E-01
132976	0.05(0.18)	0.18(0.23)	-0.03(0.12)	0.13(0.29)	-0.08(0.21)	-0.21(0.26)	6.55E-01	7.05E-01	4.12E-01	9.95E-01	9.04E-01	7.35E-01
4925	-0.06(0.18)	0.01(0.16)	-0.15(0.16)	0.06(0.24)	-0.09(0.24)	-0.15(0.23)	7.89E-01	7.09E-01	4.98E-01	9.95E-01	9.04E-01	7.82E-01
87720	-0.15(0.15)	-0.13(0.15)	-0.23(0.17)	0.01(0.21)	-0.08(0.23)	-0.10(0.22)	9.51E-01	7.14E-01	6.60E-01	9.95E-01	9.05E-01	8.85E-01
4932	-0.12(0.95)	0.01(0.11)	0.24(0.17)	0.12(0.97)	0.35(0.97)	0.23(0.20)	8.98E-01	7.20E-01	2.71E-01	9.95E-01	9.06E-01	6.19E-01
119129	0.04(0.12)	0.17(0.16)	0.15(0.32)	0.13(0.20)	0.12(0.35)	-0.02(0.36)	5.11E-01	7.40E-01	9.64E-01	9.95E-01	9.20E-01	9.97E-01
1996	-0.05(0.22)	0.05(0.45)	-0.12(0.11)	0.09(0.49)	-0.08(0.24)	-0.17(0.46)	8.49E-01	7.52E-01	7.09E-01	9.95E-01	9.26E-01	9.11E-01
64551	0.06(0.16)	-0.06(0.19)	0.12(0.13)	-0.12(0.24)	0.06(0.20)	0.18(0.23)	6.14E-01	7.73E-01	4.34E-01	9.95E-01	9.41E-01	7.51E-01
4938	-0.39(1.49)	0.10(0.75)	0.03(0.09)	0.48(1.70)	0.41(1.50)	-0.07(0.76)	7.79E-01	7.83E-01	9.30E-01	9.95E-01	9.46E-01	9.97E-01
160464	0.09(0.17)	0.08(0.15)	0.15(0.17)	-0.01(0.23)	0.06(0.25)	0.07(0.23)	9.65E-01	8.11E-01	7.65E-01	9.95E-01	9.62E-01	9.25E-01
139436	0.12(0.14)	0.03(0.16)	0.18(0.22)	-0.10(0.21)	0.06(0.25)	0.15(0.27)	6.51E-01	8.22E-01	5.70E-01	9.95E-01	9.65E-01	8.41E-01
34075	0.08(0.42)	0.17(0.13)	-0.02(0.22)	0.09(0.44)	-0.10(0.47)	-0.19(0.25)	8.41E-01	8.24E-01	4.51E-01	9.95E-01	9.65E-01	7.54E-01
1675	0.00(0.32)	0.14(0.42)	0.07(0.10)	0.14(0.54)	0.07(0.34)	-0.07(0.44)	7.93E-01	8.27E-01	8.76E-01	9.95E-01	9.65E-01	9.62E-01
119167	0.07(0.17)	0.07(0.18)	0.03(0.14)	-0.00(0.25)	-0.04(0.22)	-0.05(0.23)	9.89E-01	8.46E-01	8.41E-01	9.96E-01	9.72E-01	9.52E-01
2011	-0.04(0.12)	-0.43(0.29)	0.00(0.18)	-0.39(0.31)	0.04(0.22)	0.43(0.34)	2.19E-01	8.49E-01	2.13E-01	9.95E-01	9.72E-01	5.73E-01
160463	0.07(0.17)	0.16(0.18)	0.11(0.13)	0.09(0.24)	0.04(0.22)	-0.05(0.22)	7.06E-01	8.56E-01	8.11E-01	9.95E-01	9.72E-01	9.41E-01
103102	-0.01(0.28)	0.21(0.36)	-0.07(0.11)	0.22(0.45)	-0.05(0.30)	-0.27(0.36)	6.28E-01	8.56E-01	4.55E-01	9.95E-01	9.72E-01	7.54E-01
139478	0.07(0.22)	0.04(0.12)	0.36(1.60)	-0.03(0.24)	0.29(1.62)	0.32(1.61)	8.90E-01	8.59E-01	8.40E-01	9.95E-01	9.72E-01	9.52E-01
145492	-0.04(0.62)	-0.10(0.10)	0.07(0.22)	-0.06(0.63)	0.11(0.66)	0.18(0.24)	9.20E-01	8.63E-01	4.65E-01	9.95E-01	9.74E-01	7.54E-01
4971	-0.01(0.15)	0.08(0.15)	-0.05(0.19)	0.09(0.22)	-0.04(0.24)	-0.13(0.24)	6.68E-01	8.69E-01	5.77E-01	9.95E-01	9.75E-01	8.41E-01
4927	0.06(0.11)	0.03(0.23)	0.11(0.28)	-0.03(0.25)	0.05(0.30)	0.08(0.37)	9.01E-01	8.71E-01	8.28E-01	9.95E-01	9.75E-01	9.43E-01
153	0.00(0.35)	0.37(0.27)	0.05(0.11)	0.37(0.45)	0.06(0.36)	-0.32(0.29)	4.14E-01	8.78E-01	2.79E-01	9.95E-01	9.76E-01	6.19E-01
4936	-0.55(1.18)	0.16(0.25)	-0.37(0.29)	0.71(1.20)	0.18(1.21)	-0.52(0.38)	5.58E-01	8.81E-01	1.76E-01	9.95E-01	9.77E-01	5.45E-01
110	-0.03(0.27)	-0.15(0.31)	0.01(0.11)	-0.12(0.41)	0.04(0.29)	0.16(0.33)	7.68E-01	8.88E-01	6.25E-01	9.95E-01	9.80E-01	8.69E-01
4887	-0.07(0.17)	-0.06(0.12)	-0.13(0.43)	0.01(0.21)	-0.06(0.46)	-0.07(0.44)	9.54E-01	8.97E-01	8.71E-01	9.95E-01	9.83E-01	9.62E-01
657	0.01(0.27)	0.05(0.12)	-0.03(0.17)	0.04(0.30)	-0.04(0.32)	-0.08(0.21)	8.96E-01	9.04E-01	7.07E-01	9.95E-01	9.83E-01	9.11E-01

5482	0.02(0.19)	0.06(0.13)	0.05(0.20)	0.05(0.23)	0.03(0.26)	-0.01(0.24)	8.46E-01	9.07E-01	9.53E-01	9.95E-01	9.83E-01	9.97E-01
137	0.00(0.15)	0.09(0.22)	0.02(0.14)	0.10(0.27)	0.02(0.21)	-0.07(0.26)	7.21E-01	9.09E-01	7.86E-01	9.95E-01	9.83E-01	9.34E-01
106629	-0.04(0.11)	0.62(0.73)	-0.07(0.23)	0.66(0.74)	-0.03(0.26)	-0.68(0.75)	3.76E-01	9.18E-01	3.63E-01	9.95E-01	9.86E-01	6.99E-01
1912	-0.09(0.21)	0.02(0.11)	-0.12(0.26)	0.10(0.23)	-0.03(0.33)	-0.14(0.28)	6.65E-01	9.20E-01	6.34E-01	9.95E-01	9.86E-01	8.69E-01
3286	-0.30(1.51)	0.42(2.35)	-0.15(0.10)	0.72(2.78)	0.15(1.51)	-0.57(2.35)	7.97E-01	9.21E-01	8.10E-01	9.95E-01	9.86E-01	9.41E-01
17664	-0.06(0.30)	0.04(0.11)	-0.03(0.26)	0.10(0.32)	0.04(0.40)	-0.07(0.28)	7.48E-01	9.26E-01	8.18E-01	9.95E-01	9.86E-01	9.41E-01
88583	0.07(0.15)	0.18(0.19)	0.05(0.15)	0.11(0.24)	-0.02(0.21)	-0.13(0.24)	6.31E-01	9.30E-01	5.78E-01	9.95E-01	9.86E-01	8.41E-01
307	-0.05(0.15)	0.06(0.21)	-0.07(0.15)	0.11(0.26)	-0.02(0.21)	-0.12(0.26)	6.81E-01	9.34E-01	6.43E-01	9.95E-01	9.86E-01	8.75E-01
106936	0.04(0.15)	0.03(0.18)	0.03(0.17)	-0.01(0.23)	-0.02(0.22)	0.00(0.24)	9.53E-01	9.36E-01	9.86E-01	9.95E-01	9.86E-01	9.97E-01
139467	-0.08(0.13)	0.13(0.17)	-0.05(0.52)	0.22(0.21)	0.04(0.53)	-0.18(0.54)	3.07E-01	9.43E-01	7.45E-01	9.95E-01	9.90E-01	9.12E-01
443	-0.12(0.21)	0.10(0.13)	-0.10(0.17)	0.22(0.24)	0.02(0.26)	-0.20(0.21)	3.65E-01	9.50E-01	3.42E-01	9.95E-01	9.90E-01	6.79E-01
6526	0.00(0.12)	0.16(0.20)	0.02(0.21)	0.16(0.24)	0.01(0.24)	-0.15(0.30)	5.04E-01	9.52E-01	6.22E-01	9.95E-01	9.90E-01	8.69E-01
4975	0.04(0.18)	-0.09(0.16)	0.05(0.15)	-0.13(0.24)	0.01(0.24)	0.14(0.21)	6.04E-01	9.68E-01	5.24E-01	9.95E-01	9.90E-01	7.99E-01
41691	-0.25(0.47)	-0.10(0.29)	-0.24(0.10)	0.16(0.55)	0.02(0.48)	-0.14(0.31)	7.75E-01	9.70E-01	6.54E-01	9.95E-01	9.90E-01	8.84E-01
146957	-0.15(0.20)	0.08(0.19)	-0.16(0.13)	0.23(0.27)	-0.01(0.23)	-0.23(0.23)	4.08E-01	9.71E-01	3.04E-01	9.95E-01	9.90E-01	6.55E-01
4956	-0.06(0.50)	-0.04(0.10)	-0.04(0.24)	0.02(0.51)	0.02(0.55)	-0.01(0.26)	9.62E-01	9.75E-01	9.77E-01	9.95E-01	9.90E-01	9.97E-01
4533	0.33(0.42)	0.03(0.12)	0.34(0.16)	-0.31(0.43)	0.01(0.45)	0.32(0.20)	4.80E-01	9.79E-01	1.22E-01	9.95E-01	9.90E-01	4.84E-01
17253	0.08(0.20)	-0.05(0.15)	0.07(0.17)	-0.13(0.25)	-0.01(0.27)	0.13(0.23)	5.92E-01	9.79E-01	5.85E-01	9.95E-01	9.90E-01	8.41E-01
41699	0.04(0.24)	0.09(0.33)	0.04(0.11)	0.05(0.42)	-0.01(0.27)	-0.06(0.35)	9.05E-01	9.84E-01	8.73E-01	9.95E-01	9.92E-01	9.62E-01
4609	0.07(0.18)	0.18(0.41)	0.08(0.17)	0.11(0.45)	0.00(0.25)	-0.11(0.45)	8.06E-01	9.87E-01	8.11E-01	9.95E-01	9.92E-01	9.41E-01
4986	0.07(0.25)	0.03(0.14)	0.07(0.14)	-0.04(0.29)	0.00(0.29)	0.04(0.20)	8.81E-01	9.97E-01	8.20E-01	9.95E-01	9.97E-01	9.41E-01

Regression coefficient β* (standard error (SE)) denotes the association between % change in metabolites and % change in HbA1c in each intervention group (IMI, BAND or RYGB). β† (SE) denotes the association between % change in metabolites and % change in HbA1c induced by different types of weight-loss interventions (BAND vs. IMI, RYGB vs. IMI, and RYGB vs. BAND), and was obtained from the interaction term (% change in metabolites × types of intervention) in a linear regression model. In the model, the dependent variable was % change in HbA1c, and % change in each metabolite, intervention groups (RYGB, BAND, IMI), and their interactions (% change in each metabolite × types of intervention) were the independent variables, adjusting for weight loss, sex, baseline age and BMI.

Table S5. Metabolite modules identified by WGCNA among participants who received RYGB (n=25)

Metabolite	Module
Valine	Blue
Urea	Blue
UDP GlcNac	Blue
Tyrosine	Blue
Trans-4-Hydroxyproline	Blue
Threonine	Blue
Serine	Blue
Pyrophosphate	Blue
Pseudo Uridine	Blue
Proline	Blue
Phosphate	Blue
Phenylalanine	Blue
Pelargonic Acid	Blue
Oxoproline	Blue
Ornithine	Blue
Myo-Inositol	Blue
Methionine	Blue
Methanolphosphate	Blue
Lyxitol	Blue
Leucine	Blue
Isothreitol	Blue
Isoleucine	Blue
Indole-3-Lactate	Blue
Glycine	Blue
Glycerol-Alpha-Phosphate	Blue
Galactose	Blue
Dodecanol	Blue
Dehydroabietic Acid	Blue
Conduritol-Beta-Expoxide	Blue
Cholesterol	Blue
Caprylic Acid	Blue
Asparagine	Blue
Aminomalonate	Blue
Alanine	Blue
Acetaminophen	Blue
4-Hydroxyphenylacetic Acid	Blue
2-Hydroxyvaleric Acid	Blue
131620	Blue
103102	Blue
54	Blue
106629	Blue
257	Blue
3258	Blue
1148	Blue

17068	Blue
4945	Blue
1941	Blue
121890	Blue
4577	Blue
1981	Blue
1996	Blue
20903	Blue
46292	Blue
87756	Blue
135260	Blue
16550	Blue
2061	Blue
14509	Blue
146957	Blue
132379	Blue
47	Blue
62	Blue
1029	Blue
117171	Blue
95550	Blue
112487	Blue
62433	Blue
1709	Blue
443	Blue
6066	Blue
2936	Blue
7718	Blue
145492	Blue
88583	Blue
Succinic Acid	Brown
Pyrrole-2-Carboxylic Acid	Brown
Parabanic Acid Nist	Brown
Oxamic Acid	Brown
Oxalic Acid	Brown
Indole-3-Acetate	Brown
Ethanolamine	Brown
Alloxanoic Acid Nist	Brown
2,3-Dihydroxybutanoic Acid Nist	Brown
2-Piperidinobenzonitrile Nist	Brown
2-Hydroxypyrazinyl-2-Propenoic Acid Ethyl Ester Nist	Brown
2-Hydroxyglutaric Acid	Brown
4938	Brown
4925	Brown
5483	Brown
130396	Brown
4942	Brown

4943	Brown
4941	Brown
17537	Brown
3329	Brown
93947	Brown
6526	Brown
6580	Brown
41697	Brown
126397	Brown
6579	Brown
17263	Brown
13107	Brown
4971	Brown
4981	Brown
6864	Brown
4898	Brown
21513	Brown
6331	Brown
39	Brown
4976	Brown
41699	Brown
5482	Brown
4937	Brown
16561	Brown
120526	Brown
479	Brown
122151	Brown
87769	Brown
110	Brown
1872	Brown
490	Brown
120562	Brown
1878	Brown
132414	Brown
Uridine	Grey
Uric Acid	Grey
Udp-Glucuronic Acid	Grey
Tryptophan	Grey
Threonic Acid	Grey
Isothreonic Acid	Grey
Taurine	Grey
Sucrose	Grey
Shikimic Acid	Grey
Salicylic Acid	Grey
Quinic Acid	Grey
Pyruvic Acid	Grey
Pipecolinic Acid	Grey

Pinitol	Grey
Phosphoethanolamine	Grey
Phenylethylamine	Grey
Pentadecanoic Acid	Grey
Palmitoleic Acid	Grey
Palmitic Acid	Grey
Oleic Acid	Grey
Naproxen	Grey
N-Methylalanine	Grey
Myristic Acid	Grey
Methionine Sulfoxide	Grey
Mannitol	Grey
Malic Acid	Grey
Lysine	Grey
Linoleic Acid	Grey
Levoglucosan	Grey
Lactic Acid	Grey
Kynurenine	Grey
Isocitric Acid	Grey
Hypoxanthine	Grey
Hydroxyproline Dipeptide Nist	Grey
Hydroxylamine	Grey
Histidine	Grey
Hippuric Acid	Grey
Hexitol	Grey
Heptadecanoic Acid	Grey
Glycolic Acid	Grey
Glycerol	Grey
Glyceric Acid	Grey
Glutamine	Grey
Glutamic Acid	Grey
Fumaric Acid	Grey
Fucose	Grey
Erythritol	Grey
Deoxycholic Acid	Grey
Cystine	Grey
Cysteine-Glycine	Grey
Cysteine	Grey
Citric Acid	Grey
Arachidonic Acid	Grey
Arabitol	Grey
Allantoic Acid	Grey
3-Hydroxybutyric Acid	Grey
3-(1-Pyrazolyl)-L-Alanine	Grey
2,4-Diaminobutyric Acid	Grey
2-Hydroxybutanoic Acid	Grey
2-Deoxyisotetronic Acid Nist	Grey

2-Deoxytetronic Acid	Grey
1,5-Anhydroglucitol	Grey
1-Monostearin	Grey
1-Monopalmitin	Grey
5288	Grey
4926	Grey
4927	Grey
109423	Grey
134	Grey
4871	Grey
31559	Grey
26918	Grey
26916	Grey
6115	Grey
1675	Grey
87710	Grey
4863	Grey
4936	Grey
1064	Grey
98027	Grey
4533	Grey
448	Grey
4887	Grey
892	Grey
61	Grey
42357	Grey
4929	Grey
6802	Grey
5930	Grey
592	Grey
32247	Grey
6353	Grey
31357	Grey
18488	Grey
3029	Grey
139467	Grey
137	Grey
106634	Grey
41682	Grey
6538	Grey
7053	Grey
109387	Grey
119023	Grey
17664	Grey
145494	Grey
119066	Grey
4986	Grey

106936	Grey
573	Grey
41691	Grey
4543	Grey
121002	Grey
2039	Grey
657	Grey
473	Grey
41808	Grey
5900	Grey
1875	Grey
1704	Grey
91	Grey
47170	Grey
5990	Grey
17651	Grey
91421	Grey
160463	Grey
159824	Grey
117141	Grey
9392	Grey
107960	Grey
2900	Grey
87720	Grey
34075	Grey
469	Grey
Xylose	Turquoise
Xylitol	Turquoise
Tagatose	Turquoise
Stearic Acid	Turquoise
Sorbitol	Turquoise
Ribonic Acid	Turquoise
Nonadecanoic Acid	Turquoise
N-Acetylornithine	Turquoise
N-Acetylmannosamine	Turquoise
N-Acetylglycine Nist	Turquoise
Mannose	Turquoise
Lactulose	Turquoise
Ketohexose	Turquoise
Hydrocinnamic Acid	Turquoise
Hexuronic Acid	Turquoise
Glucose	Turquoise
Gluconic Acid	Turquoise
Galactonic Acid	Turquoise
Fructose	Turquoise
Creatinine	Turquoise
Butane-2,3-Diol NIST	Turquoise

Benzoic Acid	Turquoise
Behenic Acid	Turquoise
Arachidic Acid	Turquoise
Allantoin	Turquoise
Aconitic Acid	Turquoise
3,6-Anhydro-D-Galactose	Turquoise
3-Aminoisobutyric Acid	Turquoise
6646	Turquoise
4928	Turquoise
4922	Turquoise
4985	Turquoise
135889	Turquoise
160464	Turquoise
2001	Turquoise
7402	Turquoise
160542	Turquoise
7403	Turquoise
915	Turquoise
160313	Turquoise
8270	Turquoise
16792	Turquoise
4609	Turquoise
7440	Turquoise
111162	Turquoise
2806	Turquoise
133943	Turquoise
5085	Turquoise
132976	Turquoise
4546	Turquoise
139436	Turquoise
16788	Turquoise
119167	Turquoise
160543	Turquoise
160	Turquoise
135619	Turquoise
1725	Turquoise
4600	Turquoise
9320	Turquoise
4956	Turquoise
17253	Turquoise
135610	Turquoise
41683	Turquoise
41689	Turquoise
64551	Turquoise
127277	Turquoise
64546	Turquoise
139478	Turquoise

1912	Turquoise
21666	Turquoise
100253	Turquoise
3465	Turquoise
135869	Turquoise
119129	Turquoise
4975	Turquoise
8607	Turquoise
Tocopherol Gamma-	Yellow
Tocopherol Alpha-	Yellow
Nicotinic Acid	Yellow
N-Acetylglutamate	Yellow
Maltotriose	Yellow
Maltose	Yellow
Lauric Acid	Yellow
Cellobiose Minor	Yellow
Capric Acid	Yellow
Beta-Alanine	Yellow
Aspartic Acid	Yellow
Adenosine-5-Monophosphate	Yellow
4933	Yellow
41708	Yellow
307	Yellow
4932	Yellow
68	Yellow
4983	Yellow
5419	Yellow
3286	Yellow
93385	Yellow
2011	Yellow
3232	Yellow
4948	Yellow
453	Yellow
153	Yellow
1700	Yellow
5346	Yellow
134336	Yellow
17453	Yellow

Table S6. Metabolite modules identified by WGCNA among participants who received IMI (n=25)

Metabolite	Module
Xylose	Blue
Tryptophan	Blue
Threonine	Blue
Threonic Acid	Blue
Isothreonic Acid	Blue
Tagatose	Blue
Serine	Blue
Ribonic Acid	Blue
Pyrophosphate	Blue
Pseudo Uridine	Blue
Pinitol	Blue
Oxoproline	Blue
N-Acetylornithine	Blue
N-Acetylglutamate	Blue
Myo-Inositol	Blue
Methionine	Blue
Methanolphosphate	Blue
Mannose	Blue
Lysine	Blue
Lauric Acid	Blue
Hydroxyproline Dipeptide NIST	Blue
Hydrocinnamic Acid	Blue
Histidine	Blue
Hexuronic Acid	Blue
Hexitol	Blue
Glutamine	Blue
Glucose	Blue
Gluconic Acid	Blue
Galactonic Acid	Blue
Fumaric Acid	Blue
Fructose	Blue
Cystine	Blue
Cysteine-Glycine	Blue
Conduritol-Beta-Expoxide	Blue
Capric Acid	Blue
Butane-2,3-Diol NIST	Blue
Benzoic Acid	Blue
Allantoin	Blue
Allantoic Acid	Blue
3,6-Anhydro-D-Galactose	Blue
2-Hydroxyvaleric Acid	Blue
2-Hydroxyglutaric Acid	Blue
1-Monopalmitin	Blue
6646	Blue

4928	Blue
4922	Blue
54	Blue
4926	Blue
4985	Blue
135889	Blue
4927	Blue
160464	Blue
6115	Blue
7402	Blue
307	Blue
4932	Blue
87710	Blue
7403	Blue
4936	Blue
915	Blue
160313	Blue
4983	Blue
4609	Blue
98027	Blue
106629	Blue
4533	Blue
7440	Blue
111162	Blue
2806	Blue
5419	Blue
5085	Blue
4887	Blue
61	Blue
4546	Blue
4929	Blue
139436	Blue
592	Blue
2011	Blue
139467	Blue
4600	Blue
4945	Blue
7053	Blue
121890	Blue
17253	Blue
4986	Blue
135610	Blue
41683	Blue
46292	Blue
127277	Blue
64546	Blue
135260	Blue

110	Blue
139478	Blue
145492	Blue
159824	Blue
9392	Blue
3465	Blue
135869	Blue
Xylitol	Grey
Valine	Grey
Uridine	Grey
Uric Acid	Grey
Urea	Grey
Udp-Glucuronic Acid	Grey
Tyrosine	Grey
Tocopherol Alpha-	Grey
Taurine	Grey
Sucrose	Grey
Stearic Acid	Grey
Sorbitol	Grey
Salicylic Acid	Grey
Quinic Acid	Grey
Phosphate	Grey
Phenylethylamine	Grey
Phenylalanine	Grey
Pentadecanoic Acid	Grey
Palmitoleic Acid	Grey
Palmitic Acid	Grey
Ornithine	Grey
Oleic Acid	Grey
Nonadecanoic Acid	Grey
Naproxen	Grey
N-Methylalanine	Grey
N-Acetylglycine NIST	Grey
Myristic Acid	Grey
Methionine Sulfoxide	Grey
Mannitol	Grey
Maltose	Grey
Lyxitol	Grey
Linoleic Acid	Grey
Levoglucosan	Grey
Leucine	Grey
Lactulose	Grey
Kynurenine	Grey
Ketohexose	Grey
Isothreitol	Grey
Isoleucine	Grey
Isocitric Acid	Grey

Indole-3-Lactate	Grey
Indole-3-Acetate	Grey
Hypoxanthine	Grey
Hippuric Acid	Grey
Heptadecanoic Acid	Grey
Glycolic Acid	Grey
Glycerol-Alpha-Phosphate	Grey
Glycerol	Grey
Glyceric Acid	Grey
Glutamic Acid	Grey
Galactose	Grey
Fucose	Grey
Ethanolamine	Grey
Erythritol	Grey
Deoxycholic Acid	Grey
Cysteine	Grey
Citric Acid	Grey
Cellobiose Minor	Grey
Beta-Alanine	Grey
Behenic Acid	Grey
Asparagine	Grey
Aspartic Acid	Grey
Arachidonic Acid	Grey
Arachidic Acid	Grey
Arabitol	Grey
Aminomalonate	Grey
Adenosine-5-Monophosphate	Grey
Aconitic Acid	Grey
4-Hydroxyphenylacetic Acid	Grey
3-Hydroxybutyric Acid	Grey
3-(1-Pyrazolyl)-L-Alanine	Grey
2-Hydroxybutanoic Acid	Grey
2-Deoxyisotetronic Acid NIST	Grey
1,5-Anhydroglucitol	Grey
1-Monostearin	Grey
109423	Grey
134	Grey
4871	Grey
2001	Grey
31559	Grey
26918	Grey
26916	Grey
1675	Grey
160542	Grey
4863	Grey
68	Grey
1064	Grey

8270	Grey
16792	Grey
257	Grey
132976	Grey
892	Grey
42357	Grey
16788	Grey
5930	Grey
119167	Grey
1148	Grey
6353	Grey
31357	Grey
160	Grey
3232	Grey
135619	Grey
1725	Grey
109387	Grey
119023	Grey
119066	Grey
9320	Grey
4956	Grey
573	Grey
453	Grey
41689	Grey
20903	Grey
64551	Grey
4543	Grey
121002	Grey
153	Grey
2039	Grey
41808	Grey
21666	Grey
117171	Grey
95550	Grey
62433	Grey
443	Grey
5990	Grey
17651	Grey
7718	Grey
17453	Grey
100253	Grey
91421	Grey
160463	Grey
88583	Grey
107960	Grey
2900	Grey
87720	Grey

119129	Grey
4975	Grey
34075	Grey
8607	Grey
469	Grey
Udp Glcnac	Turquoise
Trans-4-Hydroxyproline	Turquoise
Tocopherol Gamma-	Turquoise
Succinic Acid	Turquoise
Shikimic Acid	Turquoise
Pyruvic Acid	Turquoise
Pyrrole-2-Carboxylic Acid	Turquoise
Proline	Turquoise
Pipecolinic Acid	Turquoise
Phosphoethanolamine	Turquoise
Pelargonic Acid	Turquoise
Parabanic Acid NIST	Turquoise
Oxamic Acid	Turquoise
Oxalic Acid	Turquoise
Nicotinic Acid	Turquoise
N-Acetylmannosamine	Turquoise
Maltotriose	Turquoise
Malic Acid	Turquoise
Lactic Acid	Turquoise
Hydroxylamine	Turquoise
Glycine	Turquoise
Dodecanol	Turquoise
Dehydroabietic Acid	Turquoise
Creatinine	Turquoise
Cholesterol	Turquoise
Caprylic Acid	Turquoise
Alloxanoic Acid NIST	Turquoise
Alanine	Turquoise
Acetaminophen	Turquoise
3-Aminoisobutyric Acid	Turquoise
2,4-Diaminobutyric Acid	Turquoise
2,3-Dihydroxybutanoic Acid NIST	Turquoise
2-Piperidinobenzonitrile NIST	Turquoise
2-Hydroxypyrazinyl-2-Propenoic Acid Ethyl Ester NIST	Turquoise
2-Deoxytetronic Acid	Turquoise
131620	Turquoise
103102	Turquoise
5288	Turquoise
4938	Turquoise
4933	Turquoise
4925	Turquoise
41708	Turquoise

5483	Turquoise
130396	Turquoise
4942	Turquoise
4943	Turquoise
4941	Turquoise
17537	Turquoise
3329	Turquoise
133943	Turquoise
448	Turquoise
93947	Turquoise
3286	Turquoise
6526	Turquoise
93385	Turquoise
6802	Turquoise
6580	Turquoise
41697	Turquoise
3258	Turquoise
126397	Turquoise
6579	Turquoise
17263	Turquoise
32247	Turquoise
160543	Turquoise
13107	Turquoise
18488	Turquoise
3029	Turquoise
4971	Turquoise
137	Turquoise
17068	Turquoise
106634	Turquoise
4981	Turquoise
6864	Turquoise
4898	Turquoise
21513	Turquoise
41682	Turquoise
6538	Turquoise
4948	Turquoise
6331	Turquoise
39	Turquoise
4976	Turquoise
1941	Turquoise
41699	Turquoise
17664	Turquoise
145494	Turquoise
4577	Turquoise
1981	Turquoise
106936	Turquoise
5482	Turquoise

1996	Turquoise
4937	Turquoise
16561	Turquoise
120526	Turquoise
41691	Turquoise
479	Turquoise
122151	Turquoise
87756	Turquoise
87769	Turquoise
1700	Turquoise
657	Turquoise
473	Turquoise
5900	Turquoise
16550	Turquoise
1872	Turquoise
490	Turquoise
120562	Turquoise
2061	Turquoise
14509	Turquoise
1875	Turquoise
146957	Turquoise
1878	Turquoise
1912	Turquoise
132379	Turquoise
5346	Turquoise
47	Turquoise
62	Turquoise
1029	Turquoise
1704	Turquoise
134336	Turquoise
112487	Turquoise
1709	Turquoise
91	Turquoise
47170	Turquoise
6066	Turquoise
2936	Turquoise
117141	Turquoise
132414	Turquoise

Table S7. Table S5. Metabolite modules identified by WGCNA among participants who received BAND (n=25)

Metabolite	Module
Xylose	Blue
Uric Acid	Blue
Urea	Blue
Tocopherol Alpha	Blue
Threonic Acid	Blue
Ribonic Acid	Blue
Pseudo Uridine	Blue
Phosphoethanolamine	Blue
N-Acetylornithine	Blue
Methionine	Blue
Methanolphosphate	Blue
Lauric Acid	Blue
Hydroxylamine	Blue
Hydrocinnamic Acid	Blue
Glycolic Acid	Blue
Glyceric Acid	Blue
Gluconic Acid	Blue
Galactonic Acid	Blue
Fructose	Blue
Erythritol	Blue
Dodecanol	Blue
Dehydroabietic Acid	Blue
Cholesterol	Blue
Capric Acid	Blue
Butane-2,3-Diol NIST	Blue
Benzoic Acid	Blue
Allantoin	Blue
Allantoic Acid	Blue
3,6-Anhydro-D-Galactose	Blue
2-Hydroxyvaleric Acid	Blue
2-Deoxytetronic Acid	Blue
4928	Blue
4922	Blue
54	Blue
4985	Blue
135889	Blue
4927	Blue
160464	Blue
7402	Blue
160542	Blue
87710	Blue
7403	Blue
4936	Blue

915	Blue
160313	Blue
130396	Blue
8270	Blue
16792	Blue
4609	Blue
98027	Blue
7440	Blue
111162	Blue
2806	Blue
133943	Blue
5085	Blue
4887	Blue
4546	Blue
4929	Blue
139436	Blue
16788	Blue
119167	Blue
160543	Blue
6353	Blue
137	Blue
4945	Blue
7053	Blue
121890	Blue
17253	Blue
4986	Blue
4577	Blue
64551	Blue
46292	Blue
127277	Blue
4543	Blue
110	Blue
16550	Blue
139478	Blue
14509	Blue
5346	Blue
1704	Blue
62433	Blue
145492	Blue
135869	Blue
119129	Blue
8607	Blue
Valine	Grey
Uridine	Grey
UDP -Glucuronic Acid	Grey
UDP GlcNac	Grey
Tyrosine	Grey

Trans-4-Hydroxyproline	Grey
Tocopherol Gamma-	Grey
Threonine	Grey
Tagatose	Grey
Sucrose	Grey
Sorbitol	Grey
Shikimic Acid	Grey
Serine	Grey
Salicylic Acid	Grey
Quinic Acid	Grey
Pyruvic Acid	Grey
Pyrophosphate	Grey
Pipecolinic Acid	Grey
Pinitol	Grey
Phosphate	Grey
Phenylalanine	Grey
Pentadecanoic Acid	Grey
Ornithine	Grey
Nonadecanoic Acid	Grey
Naproxen	Grey
N-Methylalanine	Grey
N-Acetylglycine NIST	Grey
Myo-Inositol	Grey
Mannose	Grey
Mannitol	Grey
Maltotriose	Grey
Maltose	Grey
Lyxitol	Grey
Levoglucosan	Grey
Leucine	Grey
Lactulose	Grey
Lactic Acid	Grey
Kynurenine	Grey
Ketohexose	Grey
Isoleucine	Grey
Isocitric Acid	Grey
Indole-3-Lactate	Grey
Indole-3-Acetate	Grey
Hypoxanthine	Grey
Hydroxyproline Dipeptide NIST	Grey
Hippuric Acid	Grey
Hexuronic Acid	Grey
Hexitol	Grey
Glycine	Grey
Glycerol-Alpha-Phosphate	Grey
Glutamic Acid	Grey
Glucose	Grey

Galactose	Grey
Fucose	Grey
Ethanolamine	Grey
Deoxycholic Acid	Grey
Cysteine	Grey
Conduritol-Beta-Expoxide	Grey
Citric Acid	Grey
Cellobiose Minor	Grey
Beta-Alanine	Grey
Behenic Acid	Grey
Aspartic Acid	Grey
Arachidic Acid	Grey
Alanine	Grey
Adenosine-5-Monophosphate	Grey
Aconitic Acid	Grey
Acetaminophen	Grey
4-Hydroxyphenylacetic Acid	Grey
3-Hydroxybutyric Acid	Grey
3-(1-Pyrazolyl)-L-Alanine	Grey
2,3-Dihydroxybutanoic Acid NIST	Grey
2-Hydroxypyrazinyl-2-Propenoic Acid Ethyl Ester NIST	Grey
2-Hydroxybutanoic Acid	Grey
2-Deoxyisotetronic Acid NIST	Grey
1-Monostearin	Grey
1-Monopalmitin	Grey
131620	Grey
103102	Grey
6646	Grey
109423	Grey
134	Grey
2001	Grey
31559	Grey
26918	Grey
26916	Grey
6115	Grey
1675	Grey
4863	Grey
1064	Grey
132976	Grey
892	Grey
42357	Grey
5930	Grey
32247	Grey
1148	Grey
3029	Grey
160	Grey
17068	Grey

3232	Grey
135619	Grey
1725	Grey
109387	Grey
9320	Grey
4956	Grey
1981	Grey
573	Grey
453	Grey
135610	Grey
4937	Grey
41683	Grey
41689	Grey
479	Grey
20903	Grey
87769	Grey
153	Grey
2039	Grey
64546	Grey
135260	Grey
1872	Grey
120562	Grey
2061	Grey
1875	Grey
1912	Grey
21666	Grey
132379	Grey
47	Grey
62	Grey
1029	Grey
134336	Grey
117171	Grey
112487	Grey
1709	Grey
443	Grey
6066	Grey
17651	Grey
2936	Grey
17453	Grey
100253	Grey
91421	Grey
160463	Grey
88583	Grey
117141	Grey
3465	Grey
107960	Grey
2900	Grey

87720	Grey
4975	Grey
34075	Grey
469	Grey
Xylitol	Turquoise
Tryptophan	Turquoise
Isothreonic Acid	Turquoise
Taurine	Turquoise
Succinic Acid	Turquoise
Stearic Acid	Turquoise
Pyrrole-2-Carboxylic Acid	Turquoise
Proline	Turquoise
Phenylethylamine	Turquoise
Pelargonic Acid	Turquoise
Parabanic Acid NIST	Turquoise
Palmitoleic Acid	Turquoise
Palmitic Acid	Turquoise
Oxoproline	Turquoise
Oxamic Acid	Turquoise
Oxalic Acid	Turquoise
Oleic Acid	Turquoise
Nicotinic Acid	Turquoise
N-Acetylmannosamine	Turquoise
N-Acetylglutamate	Turquoise
Myristic Acid	Turquoise
Methionine Sulfoxide	Turquoise
Malic Acid	Turquoise
Lysine	Turquoise
Linoleic Acid	Turquoise
Isothreitol	Turquoise
Histidine	Turquoise
Heptadecanoic Acid	Turquoise
Glycerol	Turquoise
Glutamine	Turquoise
Fumaric Acid	Turquoise
Cystine	Turquoise
Cysteine-Glycine	Turquoise
Creatinine	Turquoise
Caprylic Acid	Turquoise
Asparagine	Turquoise
Arachidonic Acid	Turquoise
Arabitol	Turquoise
Aminomalonate	Turquoise
Alloxanoic Acid NIST	Turquoise
3-Aminoisobutyric Acid	Turquoise
2,4-Diaminobutyric Acid	Turquoise
2-Piperidinobenzonitrile NIST	Turquoise

2-Hydroxyglutaric Acid	Turquoise
1,5-Anhydroglucitol	Turquoise
5288	Turquoise
4938	Turquoise
4933	Turquoise
4925	Turquoise
4926	Turquoise
4871	Turquoise
41708	Turquoise
307	Turquoise
4932	Turquoise
5483	Turquoise
68	Turquoise
4942	Turquoise
4943	Turquoise
4983	Turquoise
4941	Turquoise
106629	Turquoise
4533	Turquoise
17537	Turquoise
3329	Turquoise
448	Turquoise
5419	Turquoise
93947	Turquoise
257	Turquoise
3286	Turquoise
6526	Turquoise
61	Turquoise
93385	Turquoise
6802	Turquoise
6580	Turquoise
41697	Turquoise
3258	Turquoise
126397	Turquoise
6579	Turquoise
592	Turquoise
2011	Turquoise
17263	Turquoise
13107	Turquoise
31357	Turquoise
18488	Turquoise
139467	Turquoise
4971	Turquoise
106634	Turquoise
4981	Turquoise
6864	Turquoise
4600	Turquoise

4898	Turquoise
21513	Turquoise
41682	Turquoise
6538	Turquoise
4948	Turquoise
6331	Turquoise
39	Turquoise
119023	Turquoise
4976	Turquoise
1941	Turquoise
41699	Turquoise
17664	Turquoise
145494	Turquoise
119066	Turquoise
106936	Turquoise
5482	Turquoise
1996	Turquoise
16561	Turquoise
120526	Turquoise
41691	Turquoise
122151	Turquoise
87756	Turquoise
121002	Turquoise
1700	Turquoise
657	Turquoise
473	Turquoise
41808	Turquoise
5900	Turquoise
490	Turquoise
146957	Turquoise
1878	Turquoise
95550	Turquoise
91	Turquoise
47170	Turquoise
5990	Turquoise
7718	Turquoise
159824	Turquoise
9392	Turquoise
132414	Turquoise

Table S8. Module differentially connectivity analysis (MDC) (RYGB vs. IMI)

Module	Size	Connectivity	q value	Category
Brown	51	-0.73	0.976	Conserved
Turquoise	76	-19.8	0.341	Conserved
Blue	74	82.72	0.001	Gain of connectivity
Yellow	30	10.09	0.09	Conserved

Table S9. Module differentially connectivity analysis (MDC) (RYGB vs BAND)

Module	Size	Connectivity	q value	Category
Brown	51	11.46	0.72	Conserved
Turquoise	76	-41.92	0.048	Loss of connectivity
Blue	74	89.67	0.013	Gain of connectivity
Yellow	30	10.91	0.06	Conserved

Table S10. List of metabolites included in the enrichment analysis

Metabolites included in the blue module in RYGB (n=37)	Metabolites matched (n=34)	HMDB (n=34)	KEGG (n=34)
Valine	L-Valine	HMDB0000883	C00183
Urea	Urea	HMDB0000294	C00086
UDP GlcNac	Uridine diphosphate-N-acetylglucosamine	HMDB0000290	C00043
Tyrosine	L-Tyrosine	HMDB0000158	C00082
Trans-4-Hydroxyproline	4-Hydroxyproline	HMDB0000725	C01157
Threonine	L-Threonine	HMDB0000167	C00188
Serine	Serine	HMDB0062263	C00716
Pyrophosphate	Pyrophosphate	HMDB0000250	C00013
Pseudo Uridine	Pseudouridine	HMDB0000767	C02067
Proline	L-Proline	HMDB0000162	C00148
Phosphate	Phosphate	HMDB0001429	C00009
Phenylalanine	L-Phenylalanine	HMDB0000159	C00079
Pelargonic Acid	Pelargonic acid	HMDB0000847	C01601
Oxoproline	Pyroglutamic acid	HMDB0000267	C01879
Ornithine	Ornithine	HMDB0000214	C00077
Myo-Inositol	myo-Inositol	HMDB0000211	C00137
Methionine	L-Methionine	HMDB0000696	C00073
Methanolphosphate	NA	NA	NA
Lyxitol	L-Arabitol	HMDB0001851	C00532
Leucine	L-Leucine	HMDB0000687	C00123
Isothreitol	NA	NA	NA
Isoleucine	L-Isoleucine	HMDB0000172	C00407
Indole-3-Lactate	Indolelactic acid	HMDB0000671	C02043
Glycine	Glycine	HMDB0000123	C00037
Glycerol-3-Phosphate	Glycerol 3-phosphate	HMDB0000126	C00093
Galactose	D-Galactose	HMDB0000143	C00984
Dodecanol	Dodecanol	HMDB0011626	C02277
Dehydroabietic Acid	Dehydroabietic acid	HMDB0061925	C12078
Conduritol-Beta-Expoxide	NA	NA	NA
Cholesterol	Cholesterol	HMDB0000067	C00187
Caprylic Acid	Caprylic acid	HMDB0000482	C06423
Asparagine	L-Asparagine	HMDB0000168	C00152
Aminomalonate	Aminomalonic acid	HMDB0001147	C00872
Alanine	L-Alanine	HMDB0000161	C00041
Acetaminophen	Acetaminophen	HMDB0001859	C06804
4-Hydroxyphenylacetic Acid	p-Hydroxyphenylacetic acid	HMDB0000020	C00642
2-Hydroxyvaleric Acid	2-Hydroxyvaleric acid	HMDB0001863	NA

Table S11. List of background metabolites included in the enrichment analysis

Our background data	Matched	HMDB	KEGG
1,5-anhydroglucitol	1,5-Anhydrosorbitol	HMDB0002712	C07326
Glycerol-3-phosphate	Glycerol 3-phosphate	HMDB0000126	C00093
Valine	L-Valine	HMDB0000883	C00183
Isoleucine	L-Isoleucine	HMDB0000172	C00407
Shikimic acid	Shikimic acid	HMDB0003070	C00493
Fructose	D-Fructose	HMDB0000660	C02336
Quinic acid	Quinic acid	HMDB0003072	C00296
Pyrophosphate	Pyrophosphate	HMDB0000250	C00013
Galactonic acid	Galactonic acid	HMDB0000565	C00880
Leucine	L-Leucine	HMDB0000687	C00123
Cholesterol	Cholesterol	HMDB0000067	C00187
4-hydroxyphenylacetic acid	p-Hydroxyphenylacetic acid	HMDB0000020	C00642
Taurine	Taurine	HMDB0000251	C00245
Threonine	L-Threonine	HMDB0000167	C00188
Ornithine	Ornithine	HMDB0000214	C00077
Mannitol	Mannitol	HMDB0000765	C00392
Phenylalanine	L-Phenylalanine	HMDB0000159	C00079
Indole-3-acetate	Indoleacetic acid	HMDB0000197	C00954
Allantoin	Allantoin	HMDB0000462	C01551
Pipecolinic acid	Pipecolic acid	HMDB0000070	C00408
Oxoproline	Pyroglutamic acid	HMDB0000267	C01879
N-acetylmannosamine	N-Acetylmannosamine	HMDB0001129	C00645
Tyrosine	L-Tyrosine	HMDB0000158	C00082
Uric acid	Uric acid	HMDB0000289	C00366
Glucose	D-Glucose	HMDB0000122	C00221
Aspartic acid	L-Aspartic acid	HMDB0000191	C00049
Acetaminophen	Acetaminophen	HMDB0001859	C06804
Allantoic acid	Allantoic acid	HMDB0001209	C00499
Pyrrole-2-carboxylic acid	Pyrrole-2-carboxylic acid	HMDB0004230	C05942
Aminomalonate	Aminomalonic acid	HMDB0001147	C00872
Dodecanol	Dodecanol	HMDB0011626	C02277
Galactose	D-Galactose	HMDB0000143	C00984
Indole-3-lactate	Indolelactic acid	HMDB0000671	C02043
Kynurenine	L-Kynurenine	HMDB0000684	C00328
Phosphoethanolamine	O-Phosphoethanolamine	HMDB0000224	C00346
3-hydroxybutyric acid	3-Hydroxybutyric acid	HMDB0000357	C01089
Ethanolamine	Ethanolamine	HMDB0000149	C00189
Oxalic acid	Oxalic acid	HMDB0002329	C00209
Xylitol	D-Xylitol	HMDB0002917	C00379
N-acetylornithine	N-Acetylornithine	HMDB0003357	C00437
Gluconic acid	Gluconic acid	HMDB0000625	C00257
Trans-4-hydroxyproline	4-Hydroxyproline	HMDB0000725	C01157

Lactulose	Lactulose	HMDB0000740	C07064
Capric acid	Capric acid	HMDB0000511	C01571
Glycine	Glycine	HMDB0000123	C00037
Arachidonic acid	Arachidonic acid	HMDB0001043	C00219
Dehydroabietic acid	Dehydroabietic acid	HMDB0061925	C12078
Fucose	L-Fucose	HMDB0000174	C01019
Alanine	L-Alanine	HMDB0000161	C00041
Myristic acid	Myristic acid	HMDB0000806	C06424
UDP GlcNAc	Uridine diphosphate-N-acetylglucosamine	HMDB0000290	C00043
Hypoxanthine	Hypoxanthine	HMDB0000157	C00262
Phenylethylamine	Phenylethylamine	HMDB0012275	C05332
Phosphate	Phosphate	HMDB0001429	C00009
Urea	Urea	HMDB0000294	C00086
Lyxitol	L-Arabitol	HMDB0001851	C00532
Nicotinic acid	Nicotinic acid	HMDB0001488	C00253
Methionine	L-Methionine	HMDB0000696	C00073
Levoglucosan	Levoglucosan	HMDB0000640	NA
Lauric acid	Dodecanoic acid	HMDB0000638	C02679
Hippuric acid	Hippuric acid	HMDB0000714	C01586
Serine	Serine	HMDB0062263	C00716
Naproxen	Naproxen	HMDB0001923	C01517
Glycolic acid	Glycolic acid	HMDB0000115	C03547
Hydroxylamine	Hydroxylamine	HMDB0003338	C00192
Benzoic acid	Benzoic acid	HMDB0001870	C00539
2-hydroxybutanoic acid	2-Hydroxybutyric acid	HMDB0000008	C05984
Threonic acid	Threonic acid	HMDB0000943	C01620
2-deoxytetronic acid	(S)-3,4-Dihydroxybutyric acid	HMDB0000337	NA
Oleic acid	Oleic acid	HMDB0000207	C00712
Creatinine	Creatinine	HMDB0000562	C00791
2,4-diaminobutyric acid	2,4-Diaminobutyric acid	HMDB0002362	NA
Pinitol	D-Pinitol	HMDB0034219	C03844
Beta-alanine	Beta-Alanine	HMDB0000056	C00099
Succinic acid	Succinic acid	HMDB0000254	C00042
N-acetylglutamate	N-Acetylglutamic acid	HMDB0001138	C00624
Maltotriose	Maltotriose	HMDB0001262	C01835
N-methylalanine	N-Methylalanine	HMDB0094692	C02721
Adenosine-5-monophosphate	Adenosine monophosphate	HMDB0000045	C00020
2-hydroxyglutaric acid	4-Amino-5-aminomethyl-2-methylpyrimidine	NA	C20267
1-monostearin	Glycerol 1-octadecanoate	HMDB0031075	NA
Deoxycholic acid	Deoxycholic acid	HMDB0000626	C04483
Sorbitol	Sorbitol	HMDB0000247	C00794
Fumaric acid	Fumaric acid	HMDB0000134	C00122
Myo-inositol	myo-Inositol	HMDB0000211	C00137
Arabitol	D-Arabitol	HMDB0000568	C01904

Malic acid	Malic acid	HMDB0000744	C03668
Arachidic acid	Arachidic acid	HMDB0002212	C06425
Cysteine-glycine	Cysteinylglycine	HMDB0000078	C01419
Mannose	D-Mannose	HMDB0000169	C00936
Pyruvic acid	Pyruvic acid	HMDB0000243	C00022
Linoleic acid	Linoleic acid	HMDB0000673	C01595
Glyceric acid	Glyceric acid	HMDB0000139	C00258
UDP-glucuronic acid	Uridine diphosphate glucuronic acid	HMDB0000935	C00167
Heptadecanoic acid	Heptadecanoic acid	HMDB0002259	NA
Caprylic acid	Caprylic acid	HMDB0000482	C06423
Uridine	Uridine	HMDB0000296	C00299
Lysine	L-Lysine	HMDB0000182	C00047
Pentadecanoic acid	Pentadecanoic acid	HMDB0000826	C16537
Tagatose	D-Tagatose	HMDB0003418	C00795
Palmitic acid	Palmitic acid	HMDB0000220	C00249
Glutamine	L-Glutamine	HMDB0000641	C00064
Glycerol	Glycerol	HMDB0000131	C00116
Glutamic acid	L-Glutamic acid	HMDB0000148	C00025
Ribonic acid	Ribonic acid	HMDB0000867	C01685
Hydrocinnamic acid	Hydrocinnamic acid	HMDB0000764	C05629
Histidine	L-Histidine	HMDB0000177	C00135
Hexitol	Galactitol	HMDB0000107	C01697
Erythritol	Erythritol	HMDB0002994	C00503
Maltose	Maltose	HMDB0037138	C00897
3-aminoisobutyric acid	3-Aminoisobutanoic acid	HMDB0003911	C05145
Tryptophan	L-Tryptophan	HMDB0000929	C00078
1-monopalmitin	MG(16:0/0:0/0:0)	HMDB0011564	NA
Aconitic acid	cis-Aconitic acid	HMDB0000072	C00417
Pseudo uridine	Pseudouridine	HMDB0000767	C02067
Pelargonic acid	Pelargonic acid	HMDB0000847	C01601
Nonadecanoic acid	Nonadecanoic acid	HMDB0000772	C16535
Xylose	D-Xylose	HMDB0000098	C00181
Cystine	L-Cystine	HMDB0000192	C00491
Sucrose	Sucrose	HMDB0000258	C00089
Isocitric acid	Isocitric acid	HMDB0000193	C00311
2-hydroxyvaleric acid	2-Hydroxyvaleric acid	HMDB0001863	NA
Behenic acid	Behenic acid	HMDB0000944	C08281
Stearic acid	Stearic acid	HMDB0000827	C01530
Salicylic acid	Salicylic acid	HMDB0001895	C00805
Asparagine	L-Asparagine	HMDB0000168	C00152
Cysteine	L-Cysteine	HMDB0000574	C00097
Proline	L-Proline	HMDB0000162	C00148
Citric acid	Citric acid	HMDB0000094	C00158
Palmitoleic acid	Palmitoleic acid	HMDB0003229	C08362
Lactic acid	L-Lactic acid	HMDB0000190	C00186
Methionine sulfoxide	Methionine sulfoxide	HMDB0002005	C02989

Table S12. Pathway enrichment analysis for metabolites included in the blue module (RYGB vs BAND)

Pathway	Total	Expected	Hits	P-value*	FDR**	Impact
Aminoacyl-tRNA biosynthesis	18	5.111	11	0.001	0.057	0.000
Valine, leucine and isoleucine biosynthesis	4	1.136	4	0.005	0.149	0.000
Valine, leucine and isoleucine degradation	3	0.852	3	0.021	0.387	0.000
Phenylalanine, tyrosine and tryptophan biosynthesis	2	0.568	2	0.078	1.000	1.000
Arginine and proline metabolism	5	1.420	3	0.136	1.000	0.249

Total: total number of compounds in the pathway; the Hits is the actually matched number from the user uploaded data; the Impact is the pathway impact value calculated from pathway topology analysis. *P values are calculated from the enrichment analysis; ** FDR is the p value adjusted using false discovery rate;

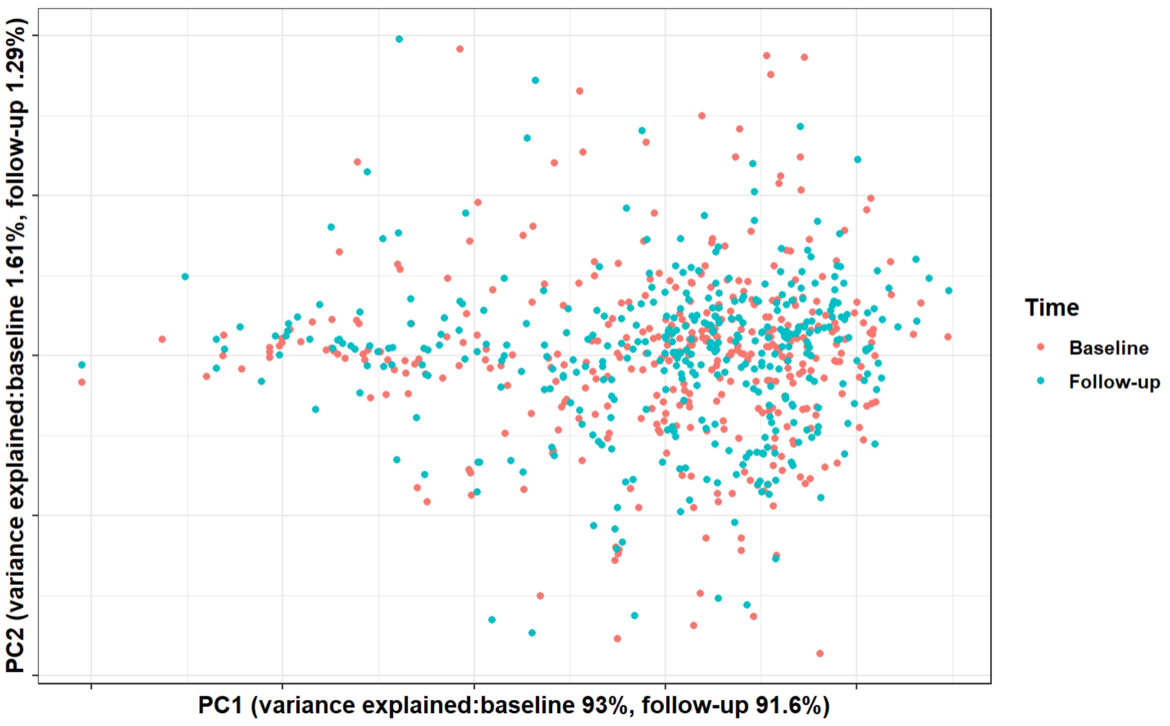

Figure S1. Principal component analysis shows that there are no clear batches in our metabolomic data. Each dot represents a metabolite measured at baseline (red) or 1-year follow-up (green). No clear batch was observed for metabolites measured at baseline and follow-up. PC1: principal component 1, PC2: principal component 2.

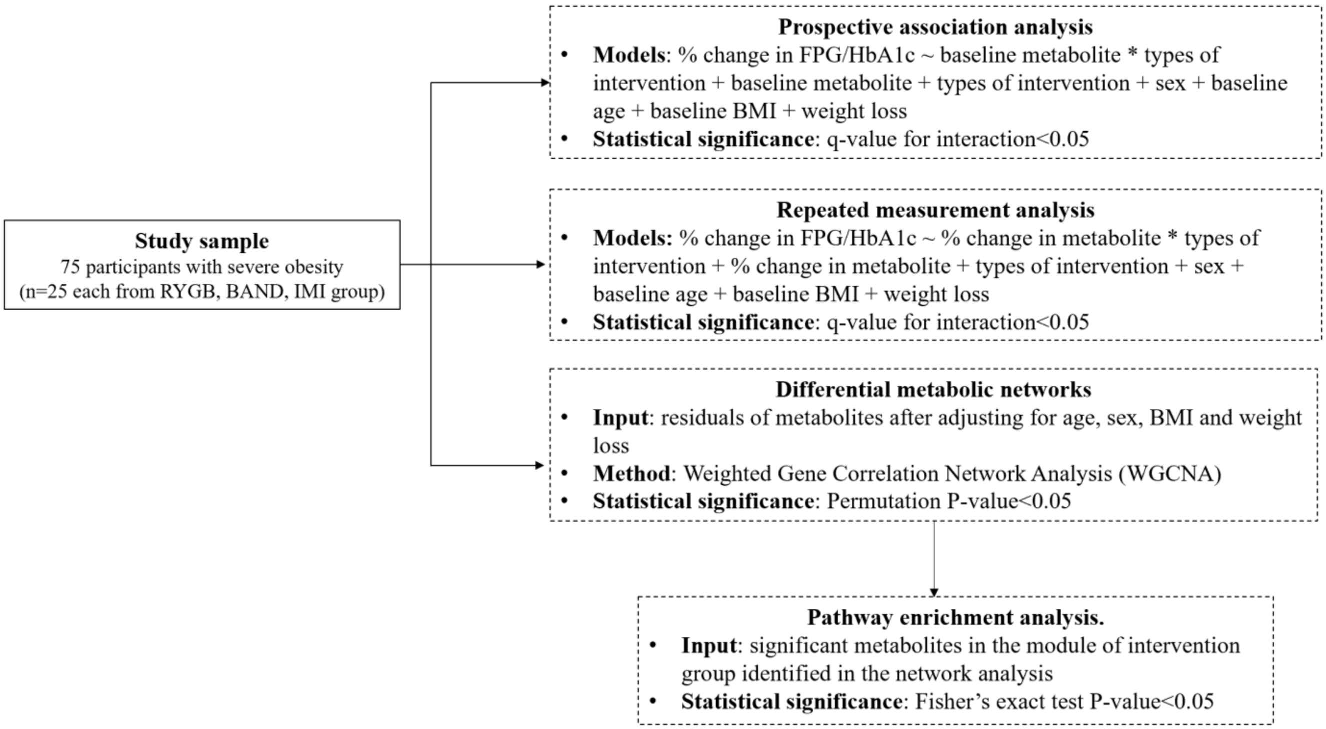

Figure S2. Statistical Analysis flowchart.
Abbreviations: RYGB, Roux-en-Y gastric bypass; BAND, adjustable gastric band; IMI, intensive medical intervention; FPG, fasting plasma glucose; HbA1c, hemoglobin A1c; FDR, false discovery rate;

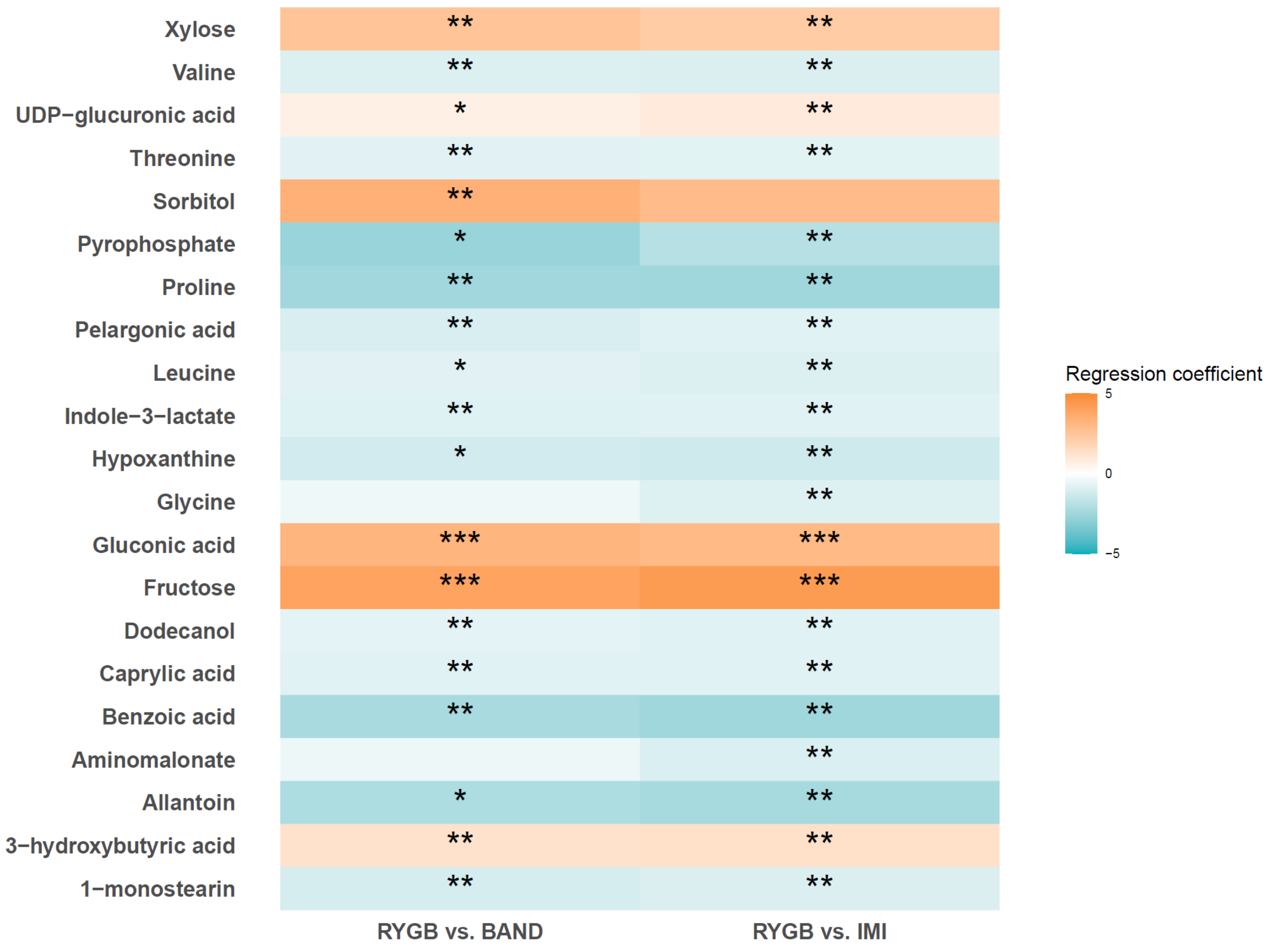

Figure S3. Heatmap showing the longitudinal association between changes in plasma metabolites and changes in fasting plasma glucose (FPG) in response to different weight-loss interventions. Each row represents a metabolite, and each column represents a different comparison group. Only significant metabolites with P<0.05 are shown here. Color codes are based on the regression coefficients obtained from the linear regression model, adjusting for weight loss, sex, baseline age, BMI. *P<0.05, **q<0.01, ***q<0.001

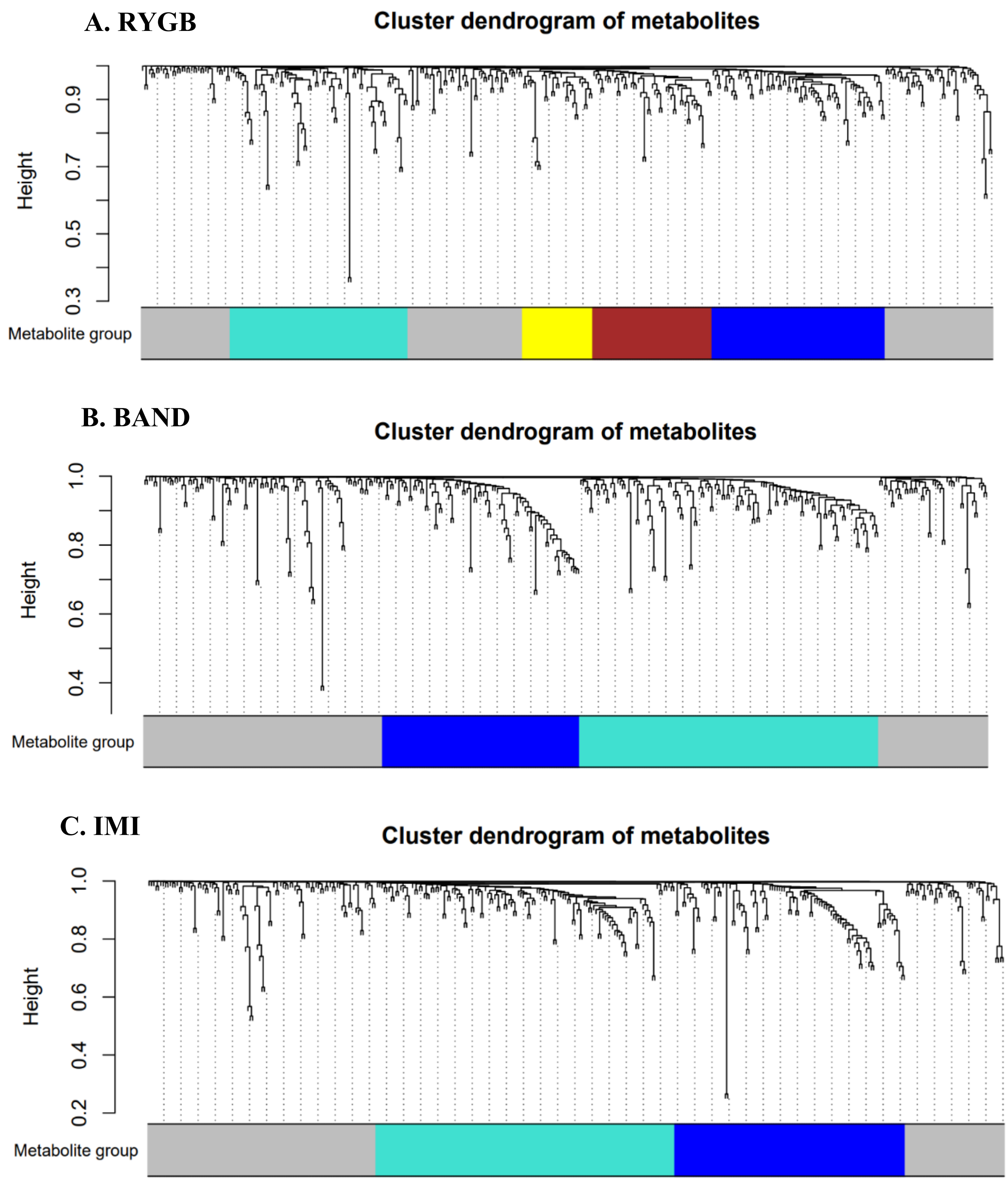

Figure S4. Membership of co-regulated metabolites modules identified by WGCNA in the RYGB group **(A)**, the BAND group **(B)** and the IMI group **(C)**. Co-regulation dendrogram was obtained by clustering the dissimilarity of metabolites based on topological overlap matrix distance. Highly co-regulated metabolites are represented by a unique color under the dendrogram, which is denoted by the module memberships. Grey color indicates that metabolites did not fall into any module.

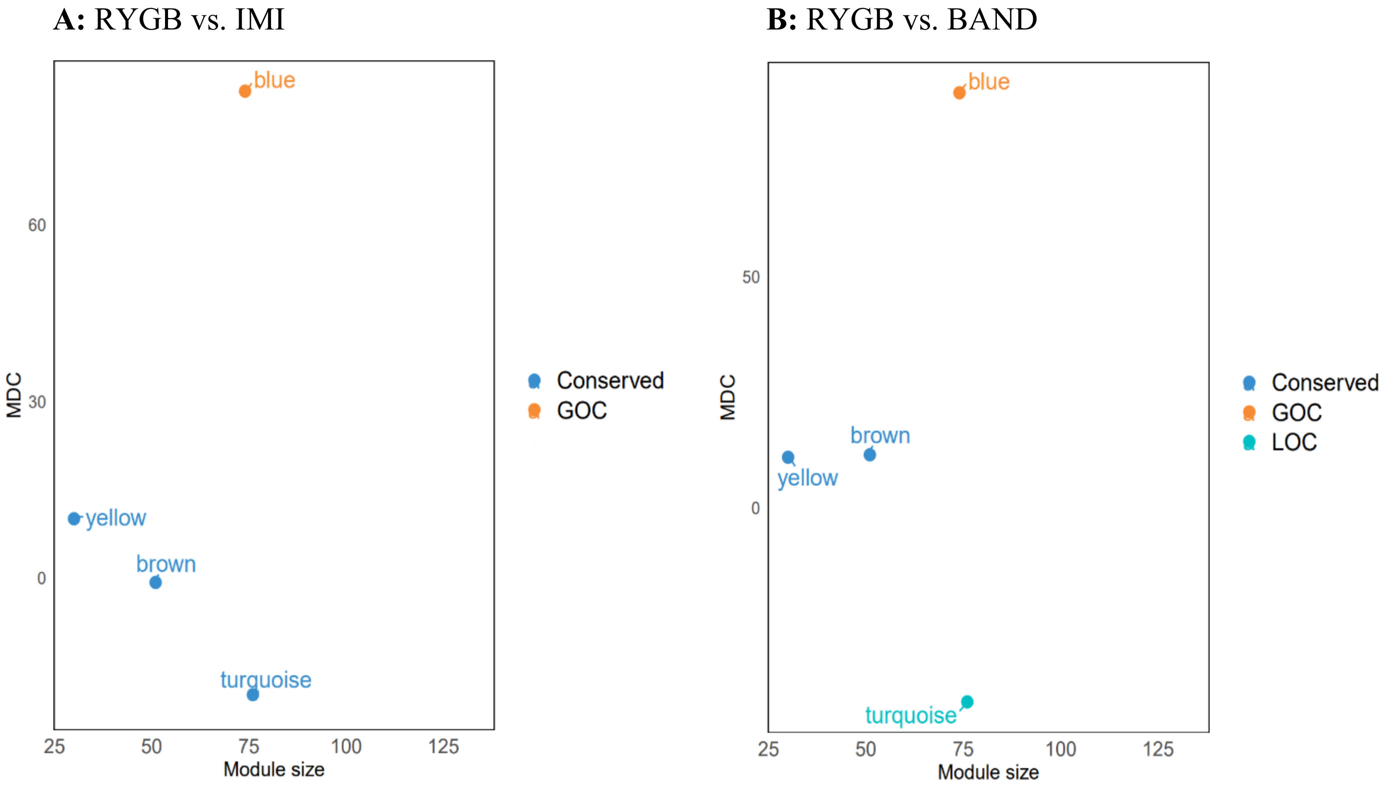

Figure S5. Differential metabolites networks (modules) associated with different types of weight-loss interventions. **A:** Compared to participants who underwent IMI, those who underwent RYGB exhibited a significant gain of connectivity (GOC) for metabolites in the blue module (MDC=82.7, P=0.001). **B:** Compared to participants who underwent BAND, those who underwent RYGB exhibited a significant gain of connectivity (GOC) for metabolites in the blue module (MDC=89.7, P=0.013) and a significant loss of connectivity (LOC) for the metabolites in the turquoise module (MDC=-15.94, P=0.011). Statistical significance of the modular differential connectivity (MDC) was determined by 1,000 permutations. Significance level was set to $P<0.05$. X-axis: module size (number of metabolites in a module). Y-axis: MDC.
